# Analysis of 44 Cases before the Landlord and Tenant Board Involving Bed Bug Infestations in Ontario, Canada: Focus on Adjudicator Decisions Based on Entomological/Pest Management Evidence and Accountability under the Residential Tenancy Act and Other Applicable Legislation

**DOI:** 10.3390/insects2030343

**Published:** 2011-07-19

**Authors:** Sam Bryks

**Affiliations:** Integrated Pest Management (IPM) Consultancy, 536 Rustic Road, Toronto, Ontario M6L 1X9, Canada; E-Mail: sbryks@gmail.com; Tel.: +1-416-248-8553; Cell: +1-647-708-5712

**Keywords:** landlord/tenant rights, conflict, accountability, blame, adjudication, mediation

## Abstract

The resurgence of bed bugs in major urban centres in North America has resulted in conflict between landlords and tenants. This is commonly focused on attribution of blame for source of infestation, on responsibility, on costs for preparation, treatment and losses, and for compensation as rent abatement and/or alternative temporary housing. In Ontario, Canada, these issues are often decided by adjudicators at the **Landlord and Tenant Board** hearing claims, counter-claims and defense by legal representation (lawyers and paralegals) as well as through mediation. Evidence in these hearings may include photographs, invoices for costs as well as testimony by tenants, landlords and “expert witnesses” who are most often pest control firms representing their landlord clients. A total of 44 **Landlord and Tenant Board** adjudicated cases available online were analyzed. The analysis included elements of the decisions such as adjudicator, claimant (landlord or tenant), basis of claim, review of evidence, amount of claim, amount awarded, and evaluation of the quality of the evidence. The results of the analysis of these findings are discussed. Recommendations for improvement of adjudicator decisions on the basis of knowledge of bed bug biology and Integrated Pest Management best practices are presented as well as the importance of education of tenants and landlords to a process of mutual trust, support and accountability.

## 1. Introduction

Landlord and tenant conflicts and rights in relation to responsibilities of each party in lease agreements and/or understandings are a familiar part of life considering that in many urban areas, the majority of homes are in multi-dwelling structures. For example, in New York City it is reported that 83% of housing stock is rental housing [1]. In Toronto, 56% of housing is multi-dwelling, and 11% of all housing is publically funded low-income housing [2]. Other urban centres with smaller populations experiencing landlord tenant conflict on bed bug issues such as Columbus, Ohio, also show similar high levels of multi-dwelling properties [3]. The term “landlord” derived from medieval England is linked to the Roman Empire and subsequently feudalism [4], but in the modern context it defines ownership of property that is rented for use. The landlord tenant conflict and the development of laws to address this have a rich history [5–7]. As society shifted from rural to urban habitation, multi-dwelling properties became much more common. Tenants of single family detached dwellings were expected to handle most aspects of property maintenance, however, with the shift to multi-dwelling units with common elements, the landlord became more accountable for maintenance [8]. Conflicts between landlords and tenants in terms of maintenance issues, and pest problems are very common. This also resulted in a political context not dissimilar to conflicts between labour and management in protection of the rights of the disadvantaged and the rights of corporations and of individual landlords as free enterprise, as exemplified by the major conflicts between tenant and landlord organizations in New York City in the 20th century [6]. Concerns about the rights of low income tenants living in slum conditions in Great Britain in the 1930's and the high incidence of bed bugs resulted in the famous study on bed bugs in slum tenements by the Ministry of Health published in 1934 [9]. One of the outcomes of this study was passage of the Public Health Act (1936) that included requirements for action to eliminate vermin, as well as providing temporary shelter to tenants if homes required gas fumigation [10].

The quality of multi-dwelling housing is often characterized in terms of quality of control of pests so that the terms “rat-infested” or “cockroach-infested” have been associated with poor quality and poor management of housing whether in the private or public sectors. Bed bugs are now on the top of the list in urgency as reflected in a survey published on the internet by Forbes showing the thirteen top infested cities in the U.S. based on number of service requests from data provided by the largest pest control firms [11]. Prior to the late twentieth century, bed bugs had been relegated to the status of “historical pests” as their occurrence was uncommon in North America, Europe, Australia [12] and in China [13]. This has been attributed to the effectiveness of modern pesticides starting with DDT but also due to improvements in general conditions in housing and likely also due to intolerance for this pest. Bed bug infestations had been significantly reduced in the U.K. before the advent of DDT [14,15]. This is considered to be due to the basic methodology of reducing clutter, careful inspections, heat treatment of possessions and available pesticide treatments as well as ongoing follow-up as was recommended in the 1934 Ministry of Health Report on the Bed-bug [9]. It was in no small part successful due to regulations and the fact that the landlord was the local government authority, and the enforcement was through the Public Health Act. The resurgence of bed bugs in the period from 1997 to the present has heightened the landlord/tenant conflict with much blame of each by the other [16].

Resolution of landlord/tenant conflicts in relation to bed bug infestations are addressed by various means including health department interventions, although some health departments considered bed bugs as a “nuisance pest” rather than a pest of public health concern and limited their involvement in addressing this issue [17]. Municipal property standard violations under by-laws are another means for complaints by tenants against landlords for bed bug problems [18]. Concurrently, landlord associations have launched defensive actions such as guidelines to enable landlords to reduce their liability by screening prospective tenants for risk as well as requiring tenants to sign statements designed to relieve the landlord of responsibility [19]. Legislators in various locations have responded to constituents of which tenants comprise a much larger group of voters than landlords by obligating landlords to disclose history of infestations in apartments to new tenants with documentation of treatment and verification that the units are “bed bug free” [20].

Although most landlord tenant regulation and bylaws as reflected in lease rental agreements do not speak to bed bugs specifically, the responsibilities of landlords and of tenants in these agreements are very similar across jurisdictions and nations [21]. Landlords are responsible for general maintenance and upkeep of homes, and to ensure that tenants have the “enjoyment” of the dwelling while tenants are expected to keep their homes in a reasonable condition, to report problems on a timely basis and co-operate as appropriate, to not interfere with other tenants and to pay their rent on a timely basis as defined in the lease rental agreement. Many regulations do specify that it is the responsibility of the landlord to keep a premise free of vermin.

The control of a pest is dependent on the behavior and actions of both the tenant and of the landlord. The tenant has accountability in terms of the upkeep of their home in a reasonable sanitary and housekeeping condition, and co-operation in treatment, and the landlord has a responsibility to all tenants to ensure that the site including the common elements is kept free of pests.

The resurgence of bed bugs and the lack of understanding of society in general of management of bed bugs in various types of multi-housing dwellings including apartments, homeless shelters, hotels, motels, and university residences created a crisis that has manifested in an increase in litigation and claims due to bed bugs.

In Ontario, Canada, Landlord tenant disputes are generally handled by an agency created for this purpose–the Landlord and Tenant Board (LTB). The purpose of the LTB is to streamline and facilitate resolutions ideally through a mediation/negotiation process and with the judgment of appointed adjudicators who hear claims and counter claims in cases in which mediation has failed. Not unlike other litigation processes, results of LTB decisions are based on presentation of claims with evidence and argument by lawyers or qualified paralegal agents. Some tenants qualify for legal aid, but many do not and may or may not be supported by a legal representative. The competence and the impartiality of the Adjudicator in reviewing evidence may also be a factor. The key participants in this process are not “expert” in pest management although “experts” may be called upon as witnesses in the dispute process. The purpose of this study was to review this process from available cases on the Internet. Each case comprised of the claims and defense and/or counterclaims of plaintiffs and the decision of the adjudicator. The initial motivation to undertake this study was the author's review of a small number of cases posted on the Ontario Non-Profit Housing Association website [22] in which it was found that some adjudicators' decisions were badly flawed on the basis of the presented facts against common sense and knowledge of bed bug biology and management of bed bug infestations.

The purpose of this study was not to evaluate legal processes as this is beyond the scope of this analysis. The legal aspects of cases relate specifically to provincial and municipal legislation under the **Ontario Residential Tenancies Act,** the **Health Protection and Promotion Act, Ontario,** and local municipal by-laws. These Acts and By-laws define responsibilities and requirements of landlords and tenants as well as of parameters by which the courts and boards render their decisions. The Acts do not generally relate specifically to the biology of one pest such as bed bugs, but function in a broader requirement relating to pests in general sometimes referred to as “vermin”.

## 2. Method of Investigation

Bed bug cases before the LTB (Appendix 1) were reviewed for predefined key elements and input into an Excel spreadsheet (Appendix 2) for analysis. The purpose of the creation of the spreadsheet was to evaluate the key elements of cases and of adjudicator decisions from plaintiff claims and/or defense or counter claims and to note any trends of resolution. The claims were documented in an Excel spreadsheet as follows: case number, date, adjudicator name, claimant (tenant and/or landlord), outcome (tenant and/or landlord), claim legal details, legal basis of award, cause, claim award details, evidence, expert witness details, missing items, evaluation of decision, appraisal of decision, score, rating of decision and expert witness type. The spreadsheet was ordered in a sequence that was parallel to the format of decisions for ease of entry.

### 2.1. Source of Data

Data was found by a search on the Canadian Legal Information Institute website [23] for cases in Ontario handled by the LTB with the key search words “bed bug”. This resulted in a total of 44 cases. All cases found including these key words were included in the study. Other terms were also used, but the term “bed bug” yielded the most results. Although there were cases in other provinces, the fact that these cases are handled primarily by the LTB in Ontario enabled more specific screening, as well as evaluation based on the specific Legislation of the Ontario Residential Tenancy Act. Further, this paper intended to evaluate the adjudication process for bed bug cases in Ontario to enable improvement of this process if necessary, as well as to provide insights to other jurisdictions. Review of cases in other jurisdictions with different practices may be undertaken at a future time. A brief review of how bed bug cases are handled in Canada in provinces [24] shows that there are similarities but also major differences between provinces in how these matters are addressed in conflict between landlords and tenants. The issue does focus on the respective responsibilities of landlords and tenants. There have been similar reviews of handling of bed bugs in jurisdictions in the U.S.

### 2.2. Parameters of Analysis

In order to evaluate these decisions in 44 cases, it was necessary to establish a qualitative evaluative process. Our approach followed the basic principles of Inductive Qualitative Analysis as outlined by Thomas [25] as follows:

1. to condense extensive and varied raw text data into a brief, summary format;
2. to establish clear links between the research objectives and the summary findings derived from the raw data and to ensure that these links are both transparent (able to be demonstrated to others) and defensible (justifiable given the objectives of the research); and
3. to develop a model or theory about the underlying structure of experiences or processes that are evident in the text data.

We evaluated the basis of adjudicator decisions in relation to the biology of bed bugs, and known factors of their spread, of detection, and of their control and prevention. As this study was not a field evaluation of specific cases, the evaluations were based on presented evidence documented in the cases, and on the decisions of adjudicators on the basis of this evidence. The evidence is described by most adjudicators within the summary of the case and includes a brief summary of presented evidence by claimant and defendant against claim as well as that of any “expert” witness. Outcomes of cases were analyzed simply in terms of outcomes for claimant or for defendant (sometimes as counter claims), or for both. The rating of the decisions was based on a number of evaluative factors. Six elements were selected for scoring. These were Cause, Detail Claim, Evidence, Expert Testimony, Missing, and Evaluation. Scoring was based on a simple pass, fail or neutral rating. (1 = pass, −1 = fail, 0 = neutral). The scores were tallied. A score of 0 or less was rated as a fail. This was directly related to how evidence and expert witness testimony impacted decisions, and if the decisions were reasonable based on the evidence. In some cases, outcomes were not particularly useful to advance control, but adjudicators are not pest management professionals and are limited within the legal framework although decisions can influence subsequent actions by landlords and/or tenants especially through mediation.

## 3. Results and Discussion

### 3.1. Results of Adjudicator Decisions

As shown in Table 1, the majority of cases were brought by tenants against landlords with much smaller number of cases brought by the landlord or in which counter claims were made by landlord or tenant. Decision outcomes in favour of tenant or of landlord were nearly evenly split. There were 9 cases in which awards were made to both tenants and landlords, mostly involving rent arrears owed to landlord. In two cases, actions were dropped. On this small sample, it appears that there was balance in adjudicator decisions not favouring landlords or tenants even though most claims were made by tenants.

### 3.2. Expert Witnesses

Expert witnesses were utilized in 19 cases (Table 2). In all but 4 cases the expert witness supported the landlord. The expert witness was the contractor's pest control firm representative in 10 cases, public health inspector in 3 cases, and unspecified in one case. It was noted in one case that a public health unit would not send an inspector deeming bed bugs to not be part of their responsibility. A family doctor's note was used as an expert source in one case, but adjudicator did not consider this as expert testimony though it was given consideration.

### 3.3. Evaluation of Decisions

LTB decisions are based on the assessment of the facts by adjudicators. The LTB's purpose is to offer fair judgments based on the Residential Tenancy Act and the Health Protection and Promotion Act of Ontario. The intention of the LTB is to enable tenants and landlords to protect their interests through enforcement of the provisions of these laws without the need to resort to costly processes in the courts. Tenants may also call upon Municipal Property Standards or Public Health units; however, they are encouraged to use the LTB due to the ability to facilitate decisions that are binding, or to enable mediation that is also enforced by the board. Most of the issues facing the Board involve the key elements of maintenance, of enjoyment of the premises, and of the responsibilities of landlords and of tenants to one another, and to other tenants in their lease agreements. Pest control issues are more complex than most other maintenance issues as successful control in an individual apartment and in the entire building hinges on actions of tenants, of landlords, as well as of pest control firms, health units, and other agencies. The Adjudicator with a long docket of cases is under pressure to render a fair decision in a relatively short timeframe, and to present this on the basis of a fair evaluation of the evidence in relation to the appropriate statutes.

It appears from these results that 71% of cases were well evaluated with reasonable decisions based on the entomological/pest management evidence, however, 12 of the decisions (27%) were adjudged not to pass on the factual bases as presented in the cases (Table 3). In case 54, the adjudicator considered that the landlord giving the tenant a few cans of spray and dust and offering another room in a rooming house was an adequate response although it was reported by other tenants that the problem existed in the site. This showed a remarkable ignorance of the difficulties of bed bug control and failure to hold the landlord accountable.

In another case (86704), the adjudicator considered that either the presence of infestation at some level as a pre-existing condition, or the failure of elimination were not sufficient grounds to terminate the lease as this was a common occurrence. In this case, the adjudicator considered that the unit was indeed infested prior to the tenant's move in. In spite of these findings the adjudicator stated in their analysis of the case that “Further, in my view, the steps taken by the Tenant were an over-reaction to a common and entirely resolvable problem. It was unnecessary for the Tenant to vacate the unit”. The landlord's agent indicated that all vacant units were fogged as a routine practice whether infested or not. This is an illegal action in the absence of infestation, and is considered a substandard practice by industry at large and Provincial Ministry of Environment Senior Scientist [26]. Other adjudicators in similar situations agreed that the tenant had the right to terminate the lease. In this circumstance, the outcomes depended on which adjudicator heard the case, rather than a consistent application of the statute. In addition, some of the facts presented by the landlord's expert witness (their pest control firm) were factually incorrect and obviously designed to prove that the tenant brought in the infestation rather than this being a pre-existing condition. This was noted in the case as follows:

“During cross-examination, Witness #3 gave a detailed explanation of the manner in which bedbug infestations occur. He advised that bedbugs do not exhibit the same travel patterns as cockroaches. Cockroaches will travel extensively throughout a building using electrical and plumbing conduits; however, bedbugs travel exclusively with a host and as a result are spread by people moving either themselves or their belongings from one lodging to another. Witness #3 was emphatic and unshakeable in his evidence that bedbugs do not generally move from one unit to another without a human carrier…”

Spread of bed bugs between units in a building is a well known fact [27]. Unfortunately, in this case, the credentials of this witness were strong in spite of the fact that the statements are patently false.

It appeared even in this failed adjudication that the adjudicator did try to give a fair decision; however, the adjudicator did not understand the facts or what should be considered an acceptable state for a tenant at moving into a unit. This was based on an interpretation of “habitability”. The adjudicator noted that “The ‘fit for habitation’ standard does not mean that the premises is literally impossible to live in; rather, the test will be whether the premises meets current standards of decent accommodation”. I think most would agree that the presence of bed bugs does not comprise “current standards of decent accommodation”. These considerations are beyond scope of this study, however, knowledge of the impact of bed bugs, difficulty of control, and the expectation of a tenant to take possession of an un-infested premises were absent in this case.

In another case (13390), the adjudicator supported landlord paying for accommodation for a pregnant tenant due to recommendation that they not be present in the unit after treatment for 24 h. This may set a precedent in future cases. In case 13393, the adjudicator adjudged the landlord's response to tenant concerns as “prompt, reasonable and professional” although there appeared to be chronic pest problems with mice, roaches and bed bugs. This was mitigated by the testimony that the tenant had not prepared the unit properly for treatment, however, the findings suggest that there were pre-existing infestation issues in the building. In addition, the female tenant was more than 8 months pregnant. This added concern and complexity and may have required accommodation. Some of the recommendations that the landlord made such as vacuuming for dead mice were not professional by any reasonable standard of pest management practices. The outcome of this case could have been different had the adjudicator been provided better evidence and facts, or had the tenant been represented by legal support.

## 4. Conclusions

Most of the adjudicators in these cases were able to render fair decisions in a situation of conflict in which the majority of the expert witnesses represented the interests of landlords. The outcomes of cases were balanced between awards to tenants and validation of landlord claims, which were mostly about rent arrears and/or preparation issues. Unfortunately, 27% of adjudicator decisions were not rated as highly and were considered failures on the basis of the evidence in relation to the biology and pest management of bed bugs. In a workshop on bed bug management through Integrated Pest Management (IPM) held in Toronto [28], two legal professionals respectively advocating for tenants or for landlords as their primary practice agreed that best resolutions between tenants and landlords should be between parties with support of mediators when needed. The LTB does provide an inexpensive means of resolving conflicts; however it is clear from this study that LTB adjudicators and similar positions in other jurisdictions would benefit from better knowledge of bed bug biology and best control and preventive practices. Further, as the majority of expert witnesses were pest control firms providing services to landlords, and tenants generally did not have availability of this resource, it may be worthwhile to have neutral expert witnesses evaluate evidence and provide their findings to LTB Adjudicators. Mediator decisions are not available, however, these may also benefit from pest management/entomological expertise.

As the cases focused on claims by specific tenants and landlords, there was little consideration given to the overall status in a site excepting evidence by the parties pertaining to the case, and in none of the cases was there any direction for follow-up at a housing site as this was out of scope of the LTB, nor were there any cases in which human rights in relation to accommodation were addressed. These are important issues which can influence abilities to control bed bugs. It is presumed that adjudicators will address such issues as in the case of a tenant who is unable to prepare for treatment and is under risk of eviction, so that the LTB can facilitate appropriate links to resources to assist in these cases, and that landlords will better understand reasonable accommodation in such instances. The Human Rights Code in Ontario requires reasonable accommodation for tenants who for reasons of health, or disability are unable to prepare. Pregnancy may fit into this category as well due to difficulty of preparation and health concerns. Landlords may need to act as facilitators in order that accommodation is provided.

This review emphasizes the importance of a societal response to the bed bug resurgence as noted by the Joint Statement of EPA/CDC on the bed bug resurgence [29]. Further, the actions of the Ontario Government in supporting education of the public and other stakeholders through funding of Health Units (2010) throughout the province will assist in addressing this issue [30]. This measure will not address the issue of education of adjudicators to ensure fair decisions, nor of the need for neutral expert witness review of difficult cases to ensure a fair evaluation. This is especially critical for low income tenants who may not qualify for legal aid resources. In the reported cases tenants were not supported by legal representatives. Finally, it appears that the LTB adjudicators do not address the wider issues in these cases, as the focus must be kept on the specifics in each case. This weakens longterm outcomes at the sites where problems may be chronic. A process to facilitate involvement of other agencies as an outcome of these hearings could be of great benefit to addressing the bed bug crisis. This could result in appropriate supports and measures for vulnerable tenants, as well as addressing problems of maintenance in assisting landlords through education and enforcement. This would support the IPM approach highlighted by the EPA/CDC joint statement as well as by other agencies in Canada and across the world. The success of a joint effort was well demonstrated by the efforts in the UK in the thirties, and can be repeated in our modern era with even better outcomes.

## Figures and Tables

**Table 1:** Adjudicator Decision Outcomes of Bed Bug Cases.

	**Adjudicator Decision Outcome Favouring**				
Claimant	**Tenant**	**Landlord**	**Both**	**None**
	
Tenant 30 (68%)	13	13	3	1
Landlord 10 (23%)	0	4	5	1
Both 4 (9%)	1	1	1	1

**Table 2:** Expert Witness Type and Alignment

**EXPERT WITNESS**	
**Representing/Type**	**Number**
Landlord Pest Control	10
Landlord Public Health	3
Tenant Pest Control	0
Tenant Public Health	3
Neutral (Unknown type)	1
Tenant Family Doctor	1
Public Health Refusal	1
None	25
TOTAL	44

**Table 3:** Adjudication Evaluation Scores.

**ADJUDICATION EVALUATION SCORES**		

**RATING**	**NUMBER**	**PERCENT**
Pass	31	71%
Fail	12	27%
Null	1	2%
	44	100%

## Supplementary Files

Supplementary File 1
