# Supplementary material for: Analysis of 44 Cases before the Landlord and Tenant Board Involving Bed Bug Infestations in Ontario, Canada: Focus on Adjudicator Decisions Based on Entomological/Pest Management Evidence and Accountability under the Residential Tenancy Act and Other Applicable Legislation
Source: Insects. 2011 Jul 19;2(3):343–53. doi: 10.3390/insects2030343 (PMC4553548; doi:10.3390/insects2030343)
Supplement: Supplementary File 1 [file insects-02-00343-s001.zip › supplementary-2/2007.EAT00054.pdf]

Landlord and Tenant Board
Ontario

Order under Section 31
Residential Tenancies Act, 2006

File Number: EAT-00054

In the matter of: [Address of Rental Unit]
Ottawa ON

Between: [Tenant] Tenant

and

Sharon Leon Landlord

[Tenant] (the 'Tenant') applied for an order determining that Sharon Leon (the 'Landlord') substantially interfered with the reasonable enjoyment of the rental unit or residential complex by the Tenant and withheld or deliberately interfered with the reasonable supply of a vital service, care service, or food that the Landlord is obligated to supply under the tenancy agreement.

This application was heard in Ottawa on March 20, 2007.

The Landlord's representative, Ms. Tammy Robinson and the Tenant attended the hearing.

Background and evidence:

<u>Substantial interference with reasonable enjoyment:</u>

At the hearing the Tenant testified that he moved into the rental unit on January 7, 2007. The rental unit is a ten foot by ten foot room in a large rooming house with several other units. He stated that Ontario Works paid his first month's rent of $420.00 directly to the Landlord.

The Tenant complained that on January 14, 2007, he discovered that he had bedbugs in his room. The Tenant advised the Health department, Ontario works and his Landlord of the problem. According to his conversation with a representative of the Health department, bedbugs are more of a nuisance than a health problem and as such, the representative mentioned that he was unable to help the Tenant with this matter. The Tenant was also dissatisfied with the Landlord's response and felt that he had been deceived into thinking that the problem would be solved. He claimed that the Landlord knew of the problem before he moved in and didn't advise him. The Tenant complained that the problem with the bedbugs has still not been solved and he claims that his belongings are still infested. The Tenant added that he has not chosen to move elsewhere because he does not want to bring the problem with him to a new location.

The Tenant, in support of his application, brought four tenants from the same rooming house to testify that they had bedbugs in their rooms as well. The Tenant and his witnesses complained of bites all over their bodies and willingly showed the results of the bedbug bites on their arms and

other visible areas of skin. The Tenant even brought a vial and a piece of tape containing small bugs that he claimed were found in his room.

The Tenant requested that he be paid $200.00 to fumigate his belongings.

The Landlord's representative testified that she responded promptly to the tenant's complaint. She stated that the Tenant was provided with a spray and a powder to counter the bugs, a bed to replace the one the he claimed was infested with bedbugs and money to wash his clothes. She added that the Tenant was even offered a different room in a different building, but had refused the offer.

The Landlord's representative, in her questioning of the Tenant's witnesses, was able to determine that, although they had visible signs of bites, some of them had not advised her of the problem. The Landlord's representative admitted that although she had spent over $1000.00 on a spray and powder for the bugs, the problem is still lingering. She added that she was working closely with the Health department to find a permanent solution but was told that a permanent solution would be difficult to reach unless both parties (tenants and landlord) worked closely in cooperation to eliminate the problem. She was further told that if measures were taken to eliminate the problem in one tenant's room, the success could be short lived if that same tenant visits or is visited by another tenant who has bedbugs and has not cooperated with the landlord to eradicate the problem.

<u>Interference with the reasonable supply of vital service:</u>

The Tenant claimed that there was no heat in his room for a period of 2 weeks. He had also complained to the Landlord that he had a problem with the window in his room and that the Landlord only did the repair at the end of February. He added that he had to be out of his room for a period of 5 days while the window was being replaced.

The Tenant wanted to be reimbursed in the amount of $165.00 for the 5 days that he was not using the room while the window in his room was being repaired and for the overpayment of rent for January 2007 as he had only taken possession on January 7, 2007.

The Landlord's representative testified that she was not made aware of the lack of heat in the Tenant's room, but that she responded promptly to the complaint of a defective window by offering to change the window as soon as she was made aware of the problem. She brought the maintenance worker as a witness to testify that he was willing to change the window shortly after the Tenant's complaint but that the Tenant preferred that the window be changed at the end of the month of February when he was planning to be out of town. The witness stated that it took 2 days for the window to be changed.

The Landlord's representative stated that Ontario Works paid the Tenant's rent directly and in full for January 2007. She further explained that any overpayment would go back to Ontario Works should they request it. She added that Ontario Works as a general rule requested that money not be reimbursed directly to the tenants when a direct payment to the Landlord is in effect.

Analysis and conclusions:

Based on the submissions of both parties, I find on the balance of probabilities that the Landlord responded promptly to the Tenant's complaint about bedbugs. She responded with the solutions that were within her control by providing the Tenant with the powder and spray that was available to counter the bedbugs, as well as providing the Tenant with a replacement bed and money to wash his clothes. The Landlord went so far as to offer alternative accommodations that the Tenant refused. In my view, it is reasonable to expect that both parties need to cooperate closely to deal with a problem of this nature and the Landlord has little control over the amount of cooperation provided by anyone but herself.

At the hearing, I gave directions to the Tenant to provide me with post hearing submissions. These submissions were to confirm that Ontario Works recognized that they had overpaid the Landlord for the January 2007 rent and were requesting reimbursement, or authorizing the Landlord to forward the reimbursement to the Tenant. This reimbursement would have been for the period of January 1, 2007 to January 6, 2007 before the Tenant had taken possession of the rental unit on January 7, 2007. The Tenant did not provide me this specific information.

I find on the balance of probabilities that the Landlord, upon the Tenant's request, chose to replace the Tenant's window during the Tenant's 2 day absence at the end of February 2007. In my view it would be unreasonable to compensate the Tenant for this period of time.

Determinations:

1. The Tenant failed to satisfy me that the Landlord seriously interfered with his reasonable enjoyment of the rental unit or interfered with the reasonable supply of a vital service (heat).

It is ordered that:

1. The Tenant's application is dismissed

March 29, 2007
Date Issued

John Nolan
Member, Landlord and Tenant Board

Eastern Region
4th floor, 255 Albert Street
Ottawa ON K1P 6A9

If you have any questions about this order, call 416-645-8080 or toll free at 1-888-332-3234.

Payment of the fine must be made to the Board by the deadline set out above. The fine can be paid by certified cheque, bank draft or money order made payable to the *Minister of Finance*. If paying in person, the debt can also be paid by cash, credit card or debit card.
