# Supplementary material for: Analysis of 44 Cases before the Landlord and Tenant Board Involving Bed Bug Infestations in Ontario, Canada: Focus on Adjudicator Decisions Based on Entomological/Pest Management Evidence and Accountability under the Residential Tenancy Act and Other Applicable Legislation
Source: Insects. 2011 Jul 19;2(3):343–53. doi: 10.3390/insects2030343 (PMC4553548; doi:10.3390/insects2030343)
Supplement: Supplementary File 1 [file insects-02-00343-s001.zip › supplementary-2/2008canlii01714.pdf]

Order under Section 31
Residential Tenancies Act, 2006

File Number: TST-01714

A. D. (the 'Tenant') applied for an order determining that C. R. (the 'Landlord') or the Landlord's superintendent or the Landlord's agent substantially interfered with the reasonable enjoyment of the rental unit or residential complex by the Tenant or by a member of her household.

This application was heard in Toronto on October 16, 2008. The Tenant; Mr. W. R. for the Landlord, and the Landlord's representative, Mr. G. P., attended the hearing.

The Tenant consulted Tenant Duty Counsel before the hearing.

The Tenant and Mr. R. ('Witness 1') testified at the hearing.

Determinations:
Generally:

1. The basis for the Tenant's application for substantial interference is that the unit was infested with bedbugs and that she never slept or resided there because the Landlord did not eradicate the infestation.

2. There was a written tenancy agreement (the 'Lease'). The Lease had a one year term, beginning on July 1, 2008.

3. The Tenant paid the Landlord $1,890.00 as the deposit for first and last month's rent.

4. The Tenant moved into the rental unit on July 5, 2008. That night, after she moved all her furniture and belongings into the unit, and was preparing to go to sleep, she noted bugs on her bed, which she identified as bedbugs. Given the totality of the evidence on this point, specifically, the Tenant's testimony that she saw bedbugs in the rental unit on the night of July 5, which testimony I accept, plus the Tenant's photographs of July 6, 2008, showing an insect the Landlord accepted was a bedbug, the evidence established that there were live bedbugs in the rental unit on the night of July 5, 2008.

5. The Tenant left the unit that night and slept at a friend's.

6. On July 6, she notified the Landlord about the bedbugs. On July 8, a technician from the pest control company which the Landlord had on contract treated the rental unit for bedbugs.

7. On July 8, the Tenant emailed Witness 1 and stated she was worried about spending '…a night…' in the unit, and would appreciate '…professional reassurance that every step has been taken to curb the situation' (Exhibit L2).

8. Exhibit L2 also contains Witness 1's information to the Tenant about her contractual responsibilities under the Lease, and the procedure and costs to sublet the unit or assign the Lease. Witness 1 combined the concepts of subletting and assignment, but the thrust of his information was to advise the Tenant about her potential liabilities, and how to minimize her liability for the balance of the rent for the Lease term.

9. The Tenant did not dispute that on July 8, 2008, because of the bedbugs, she thought about permanently vacating the rental unit. However, after learning more about these insects, she decided to rely on the Landlord's pest control contractor's bedbug treatment.

10. There was also no dispute that on July 29, 2008, the Landlord's contractor again treated the rental unit for bedbugs. This contractor's arrangement with the Landlord is to automatically do a follow-up, second treatment for bedbugs a few weeks after the first one, whether or not there is a request for the follow-up second treatment.

11. The Tenant stayed with friends for the entire balance of July, 2008. She did so because the Landlord's exterminator told her bedbugs might still be present for up to ten days after a treatment.

12. On August 6, 2008, the Tenant returned to the unit with the intention of residing there, as enough time should have elapsed after the July 29, 2008 treatment to rid the unit of bedbugs. That night, around midnight, she found live bedbugs in the unit, and left to stay with a friend.

13. On August 7, 2008, the Tenant gave the Landlord written notice that she no longer wished to rent *'… nor take possession of unit 2308. Effectively [sic] immediately, please begin to show the apartment to prospective tenants'* (the 'Tenant's Termination Notice').

14. In the Landlord's August 7, 2008 response to the Tenant's Termination Notice (Exhibit L2A), the Landlord again notified her of their mutual rights and obligations under the Lease and the Act. Exhibit L2A is a form letter the Landlord's system automatically generates on receipt of a tenant's termination notice, varied only by the particulars of the specific tenancy.

15. It is not relevant whether or not the Tenant received Exhibit L2A (she testified she did not). She did not dispute, and the evidence established, that from July 8, 2008, she knew what her liabilities and options were regarding her Lease obligations.

16. There was also no dispute that the Tenant never stayed overnight in the rental unit.

17. On August 12, 2008, the Tenant removed the last of her belongings from the rental unit.

18. The Landlord re-rented the unit effective August 17, 2008 (Exhibit L5).

19. At the hearing, the Landlord refunded $467.17 to the Tenant, representing daily rent from August 17, 2008 to August 31, 2008 plus interest on the last month's rent deposit.

Issue: Did the Tenant take possession of the rental unit?

20. I do not agree with either party regarding their intermittent statements and submissions that the Tenant never took possession of the rental unit. Notwithstanding that she never slept there, or lived there, she signed the Lease, paid a first and last month's rent deposit, and moved in on July 5, 2008 with, as she testified, 'everything' she '…needed to reside in the unit.'

21. Further, throughout the period between the Lease signing and at least August 12, 2008, when she moved the last of her belongings out of the rental unit, she was entitled to come and go from the unit at will, and the evidence established that she in fact did so.

22. There was no specific evidence from either party as to when the Tenant returned the keys to the Landlord. Witness 1's testimony that the Landlord did not move the Tenant's possession out of the rental unit on August 7 or thereafter, but left them there because Witness 1 felt that the Tenant would retrieve them, which she did on August 12.

23. The totality of the above evidence established that the Tenant was entitled in law to possession of the rental unit and in fact did have possession of the rental unit.

Issue: Did the tenancy terminate on August 8, 2008?

24. The Tenant also seeks an order that the tenancy was terminated on August 8, 2008. She is not entitled to that order, given her continuing obligations under the Lease contract until the unit was re-rented, and the fact that the unit was re-rented effective August 17, 2008.

25. The tenancy terminated on August 16, 2008.

Issue: Did the Landlord substantially interfere with the Tenant's reasonable enjoyment of the rental unit?

26. There were basically two time periods to take into consideration in determining this issue:

(a) July 1, 2008 to August 5, 2008

27. The Tenant is not entitled to a finding that the Landlord substantially interfered with her reasonable enjoyment during the period July 1, 2008 to and including July 4, 2008, because she did not move into the unit until July 5, 2008.

28. The first relevant period is July 5, 2008 to August 5, 2008, the latter date being the day before the Tenant went back to the unit with the intention of residing there.

29. The Tenant notified the Landlord promptly on July 6, 2008 about the bedbugs. The Landlord responded with a prompt bedbug treatment on July 8, 2008 and a reasonable second follow-up treatment on July 29, 2008.

30. The Tenant complied with all the required preparation of the unit and her furniture and clothing, as evidenced by Exhibit L4. If done properly, the preparation takes time, and this is an interference with a tenant, albeit necessary to give the pest extermination the best chance to take effect. The Tenant felt she could not sleep in the unit. Further, she kept

her clothing in plastic garbage bags rather than in closets, both as part of the preparation for the two treatments, which were three weeks apart.

31. The evidence established that during the period July 5, 2008 to August 5, 2008, the Landlord substantially interfered with the Tenant's reasonable enjoyment of the rental unit.

(b) August 6, 2008 to August 16, 2008

32. The other relevant period is from August 6, 2008, when the Tenant came back to the unit with the intention of residing there, to August 16, 2008, when the Tenant's liability for rent under the Lease ended.

33. I accept the Tenant's testimony that she found bedbugs on August 6, 2008 when she came back to the unit. The Tenant was straightforward and organized in her testimony, and she also was candid when she admitted that after she first found the bedbugs on July 5, she definitely thought about leaving the unit, but decided to rely on the potential for exterminating them. The evidence established that by August 6, 2008, the Landlord had not rid the unit of bedbugs. After again finding bedbugs that night, the Tenant left the unit, never to return to try to live there.

34. The continuing presence of the bedbugs, notwithstanding the two treatments by the Landlord, caused the Tenant again to be unable to sleep in the unit, to again have to sleep at other locations, and to be disrupted again as a consequence of the bedbugs.

35. Therefore, during the period August 6, 2008 to August 16, 2008, the Landlord also substantially interfered with the reasonable enjoyment of the rental unit or residential complex by the Tenant because the bedbugs were still in the unit. The Landlord had not succeeded in eradicating them.

Issue: Amount of Abatement of Rent

36. A tenant pays rent in exchange for a landlord supplying an interest in property (the right to exclusively occupy and reside inside a physical space) plus various other amenities and services which are either inside the physical space, or a part of it (a balcony), or used in common with others in the residential complex – for example, elevators. Subject to the parties' rights and obligations, those are the rights, goods and services that the parties have bargained for in the lease contract.

37. Abatement of rent is tied to the concept that if a tenant is paying 100% of the rent and not receiving all of the rights, goods and services for which the tenant is paying, an abatement should be granted proportionate to the difference between what the tenant is paying for and what the tenant is actually receiving.

38. In the period July 5 to August 5, 2008, the Tenant discovered the bedbugs, and decided that she could not sleep in the unit until the bedbugs were eradicated. Further, she decided not to be in the unit at all, but to live for this entire period with friends, while

occasionally returning to the rental unit – for example, to prepare it for the bedbug treatments.

39. Notwithstanding the information the Tenant received from the Landlord's pest control contractor that there could be bedbugs in evidence for up to ten days after a treatment, there was no evidence that the contractor's information included the stipulation or requirement that a tenant not reside in the unit at all during that time. The Tenant was of free to exercise that choice and not sleep or stay there, if she felt uncomfortable doing so, as she testified she did. However, that was her choice, and she cannot hold the Landlord 100% financially responsible for it.

40. As well, during this period, the Landlord did everything a landlord could reasonably do, to eliminate the bedbugs. The Landlord's actions were prompt, thorough and reasonable.

41. Nevertheless, the impact of the existence of bedbugs on the Tenant during this period was significant. She felt she could not sleep in the unit; she had to prepare the unit for the treatments, which she did thoroughly and completely; she had to dispose of certain items for fear of carrying the bedbugs or their eggs into her friends' homes; she had to store clothing in plastic garbage bags in an effort to contain the bugs; in summary, she was significantly disrupted in the use of the rental unit for the usual purposes.

42. Then, after she returned on August 6, 2008 and still found bedbugs, the foregoing consequences and disruption in the use of the rental unit continued.

43. The Tenant's rent for the unit was $945.00 per month, being $11,340.00 per year. The daily rent was $31.07 ($11,340.00 / 365 days).

44. For the period July 5 to August 5, 2008 (32 days), the rent was $994.24. Given the significant impact on the Tenant of the bedbug infestation, particularly as it relates to her ability to sleep in the unit, an abatement of twenty-five percent is reasonable. Twenty-five percent of $994.24 is $248.56.

45. The Landlord owes the Tenant $248.56, representing an abatement of rent on account of the Landlord's substantial interference with the Tenant's reasonable enjoyment of the rental unit for the period July 5 to and including August 5, 2008.

46. For the period August 6 to August 16, 2008 (11 days), the Tenant is entitled to a one-third abatement of the rent. The Landlord's efforts, although in good faith and reasonable and proper, had not succeeded in getting rid of the bedbugs. This made the disruption to her use of the unit more significant. A reasonable abatement for this period is one-third of the rent payable. The rent for eleven days is $341.77. One-third of the rent for that period is $112.78.

47. The Landlord owes the Tenant $361.34, representing the abatement of rent on account of the Landlord's substantial interference with the Tenant's reasonable enjoyment of the rental unit for all the usual purposes.

Issue: Compensation for repair and replacement costs of the Tenant's damaged, destroyed or disposed property

48. The Tenant's testimony was that she slept on a blow-up, 'camping'-type mattress in the unit. At another point, she testified that the bed she had in the unit was approximately four years old, and brought from home. In any event, she threw out the bed, after being told by pest control contractors that this was the best practice to forestall future bedbugs.

49. The Tenant included a portion of the projected $1,065.00 cost to buy a new bed (Exhibit T7) as part of her compensation request of $575.00 for her property that was damaged, destroyed or disposed of because of the bedbugs. She testified she bought a new mattress, but not the same one as she had in the unit. It was not clear if the new mattress was the one in Exhibit T7. The fact that the mattress she had in the unit was about four years old, and because she bought a different one to substitute for it was why she did not ask for the full replacement cost of the new mattress.

50. Given the internal inconsistency of her testimony regarding the type of bed she had in the unit, but recognizing that the Tenant had to discard a mattress, a reasonable amount towards the mattress' replacement cost is $100.00.

51. On July 7, 2008, the Tenant washed the items of clothing and linens that were washable. There was insufficient evidence to establish the cost of washing.

52. The Tenant threw out her duvet on the advice of the pest control contractors that this was a best practice in trying to prevent future bedbugs. She used a blanket for two months thereafter. On September 10, 2008, when in funds, she bought a replacement duvet for $146.89 (Exhibit T8).

53. On August 11, 2008, the Tenant took several items of clothing to a dry cleaner, at a cost of $151.31 (Exhibit T8). She had to wait until then because she did not have money to pay for the dry cleaning before that date.

54. The Tenant returned to the unit on August 6, 2008, with the intention of residing there. She found bedbugs, which meant that the Landlord had not succeeded in eradicating them, and which substantially interfered with her reasonable enjoyment of the rental unit. It is reasonable for the Landlord to pay for the dry cleaning of these items.

55. The Landlord owes the Tenant $100.00 towards her cost of a replacement mattress; $146.89 for her cost of a replacement duvet, plus $151.31 for her cost of dry cleaning the clothing in Exhibit T8.

56. The Landlord owes the Tenant is $398.20, representing compensation for the Tenant's reasonable costs of replacing her property which was destroyed or disposed of on account of the Landlord's substantial interference with the Tenant's reasonable enjoyment of the unit.

57. The total amount the Landlord owes the Tenant for an abatement of rent and the cost of replacement of property is $759.54.

It is ordered that:

1. The tenancy between the Landlord and the Tenant was terminated on August 16, 2008.

2. On or before February 1, 2009, the Landlord shall pay to the Tenant $361.34, representing the abatement of rent for the period during which the Landlord substantially interfered with the Tenant’s reasonable enjoyment of the rental unit for the usual purposes.

3. On or before February 1, 2009, the Landlord shall also pay the Tenant $398.20, representing compensation for the replacement cost of her property destroyed as a result of the Landlord’s substantial interference.

4. If the Landlord does not pay the Tenant the full amount of $759.54 owing by February 1, 2009, the Landlord shall also start to owe interest. This shall be simple interest calculated at 4.00% per annum, starting February 2, 2009 on the balance outstanding.

5. The Tenant has the right, at any time, to collect the full amount owing or any balance outstanding under this order.

January 21, 2009
Date Issued

Olga Luftig
Member, Landlord and Tenant Board

Toronto South Region
2nd Floor, 79 St. Clair Ave. E
Toronto ON M4T 1M6

If you have any questions about this order, call 416-645-8080 or toll free at 1-888-332-3234.
