# Supplementary material for: Analysis of 44 Cases before the Landlord and Tenant Board Involving Bed Bug Infestations in Ontario, Canada: Focus on Adjudicator Decisions Based on Entomological/Pest Management Evidence and Accountability under the Residential Tenancy Act and Other Applicable Legislation
Source: Insects. 2011 Jul 19;2(3):343–53. doi: 10.3390/insects2030343 (PMC4553548; doi:10.3390/insects2030343)
Supplement: Supplementary File 1 [file insects-02-00343-s001.zip › supplementary-2/2008canlii86704.pdf]

Landlord and Tenant Board

Order under Section 30
Residential Tenancies Act, 2006

File Number: TST-02298 and TST-02092

2008 CanLII 86704 (ON L.T.B.)

ORDER

FF (the 'Tenant') applied for an order determining that BFC (the 'Landlord') failed to meet the Landlord's maintenance obligations under the *Residential Tenancies Act, 2006* (the 'Act') or failed to comply with health, safety, housing or maintenance standards (T6 Application).
The Tenant also applied for an order determining that the Landlord seriously interfered with her reasonable enjoyment of the rental unit (T2 Application).

This application was heard in Toronto on June 9 and October 7, 2009. The Tenant was represented at the hearing by counsel, PW. The Landlord was represented during the proceeding by LW.

The Tenant gave viva voce evidence at the hearing as did two witnesses on her behalf. VR will be referred to Witness #1 and VC will be referred to throughout as Witness #2. The Tenant's evidence was concluded on June 9, 2009. The Landlord led evidence through DM (Witness #3) and CM (Witness #4). The evidence of the Landlord's witnesses was heard on October 7, 2009.

The Claim:

The essence of the Tenant's applications was that when she assumed occupation of her rental unit it was infested with bedbugs. The Tenant claims that she was unable to continue her occupation of the unit and that she occupied the unit for less than two weeks. She requested early termination of the tenancy together with the return of her first and last month's rent deposit. The Tenant also made claims for compensation for items that she alleged were disposed of as a result of the infestation.

The Landlord denied the validity of both the Tenant's T2 and T6 claims. The Landlord denied the fact of the infestation; however, in the alternative, the Landlord's position was that if there was an infestation of bedbugs they took all reasonable, timely and necessary steps to rid the unit of the infestation.

The Evidence:

The Tenant moved into the rental unit on October 1, 2008 with her two and one-half year old daughter following a separation from her husband. The Tenant executed a written lease which stipulated a rental term of six (6) months. The Tenant testified that she noticed itchy bumps on her skin within days of moving in. She treated what she believed to be a skin irritation with Benadryl. This over-the-counter medication did not relieve her symptoms and so the Tenant attended at her family doctor. He prescribed some medication and referred her to a specialist. The specialist reported to the Tenant's primary care physician by letter dated October 10, 2008

(Exhibit 2). The Specialist's "Impression" was that the Tenant had suffered an "Arthropod Assault".

The Tenant testified that after being told by the specialist of his diagnosis she put white sheets on her bed, and that she was able to clearly the bed bugs as a result. The Tenant stated that she was so disgusted that she threw out her bedding and asked her estranged husband to take her daughter until she could sort the problem out.

The Tenant stated that she went to the building office on "Monday" and that she was told to attend the Landlord's head office. She stated that she did so and showed "Josephine" her bites and a video of the bugs.

The Tenant stated that she was offered alternate accommodation at King and Dufferin but that when she went to see this building she determined it was not suitable. She returned to the Landlord's office to advise that she wanted to terminate the tenancy. The Tenant testified that a secretary in the office, not Josephine, stated that it was probably she, the Tenant, who had brought the bedbugs into the building, if indeed they existed. Clearly, the Tenant was very offended by this suggestion as she presented a letter from her previous landlord stating that there had never been any problems with bedbugs in the Tenant's former unit, either during or after the termination of her tenancy (Exhibit 3).

The Tenant completed a Moving Out Notice provided by the Landlord on October 15, 2008 (Exhibit 5) giving an effective termination date of October 31, 2008. The Tenant testified that she actually left the unit on this date and moved in with her friend Witness #1. She further testified that she began looking for a new place to live on or about this date. The Tenant admitted that in response to Exhibit 5 she received a letter dated October 24, 2008 (Exhibit 8) advising her that she could not terminate her lease without sixty (60) days written notice before the end of the lease term.

The Tenant stated that she received a Notice that that Landlord would be spraying on October 18th and admitted that she received an accompanying 4 page information sheet regarding preparation of her unit. In cross-examination the Tenant admitted that she got this Notice on or about October 16th, only two (2) days after she complained to the Landlord about the bedbugs. The Tenant testified that she prepared the unit as instructed and showed photographs of her unit with her belongings in plastic garbage bags. The Tenant testified that when she returned to the unit after the spray there were still a substantial number of live bugs as well as many dead ones. She admitted that she did not write to the Landlord to advise of her dissatisfaction with the results of the spray.

The Tenant testified that she threw out all of her soft furnishings and kept only 2 chairs, a television, a table and a bedroom dresser. The Tenant supplied a list of discarded items as Exhibit 7.

The Tenant testified that after she left the unit the bites stopped. The Tenant testified that she could not prove that the bugs were in the unit when she moved in but was adamant in her assertion that she did not suffer from bedbug bites before she moved into the unit.

2008 CanLII 86704 (ON L.T.B.)

2008 CanLII 86704 (ON L.T.B.)

Witness #1 has known the Tenant for more than 5 years. She saw the Tenant on Tuesday October 14th and observed the skin irritation and bites on the skin of the Tenant and her daughter. The Tenant stayed with Witness #1 from October 14 to November 4, 2008. The Tenant showed Witness #1 bed bugs in a plastic bag. This witness stated that the rental unit was in a filthy condition when the Tenant moved in. Witness #1 stated that she saw dead and alive bugs when she assisted the Tenant in moving out of her unit on October 31, 2008.

Witness #2 testified that he assisted the Tenant in preparing for her apartment for fumigation in early October 2008. He testified he saw many live and dead bedbugs. He stated that he went to the laundromat to help the Tenant do her laundry. He also stated that he helped the Tenant move out of her former apartment and that there were no bedbugs.

Witness #2 stated that he spoke to the tenant in the unit across the hall from the Tenant's and that this individual told him that he had a severe infestation of bedbugs in his unit.

Witness #3 has been employed by Magical Pest Control for three (3) years. He has been in the pest control industry for thirty (30) years. He admitted in cross-examination that he did not personally attend the unit but advised that he holds three (3) different government licenses in relation to pest control. He testified that Magical Pest Control has a monthly maintenance contract for common areas of the residential complex in which the rental unit is located. In addition, they are contracted by the Landlord to perform special calls. In establishing his credentials, the witness stated that he is a member of a federal government task force addressing the issue of bedbugs. He testified that in the 1930's and 1940's bed bugs had all but disappeared; however, he stated that about ten (10) years ago they made a resurgence and that they are now a very serious problem in Canada, much more so than cockroaches.

The witness stated that this Landlord has never refused to authorize any pest control treatment plan recommended by the company.

The witness identified an invoice for spraying the rental unit dated October 17, 2008. He testified that this unit had never previously been treated and that it has not been treated since. He did admit, in cross-examination, that he is not aware of whether there have been other complaints about bed bugs on the floor or in the building.

During cross-examination, Witness #3 gave a detailed explanation of the manner in which bedbug infestations occur. He advised that bedbugs do not exhibit the same travel patterns as cockroaches. Cockroaches will travel extensively throughout a building using electrical and plumbing conduits; however, bedbugs travel exclusively with a host and as a result are spread by people moving either themselves or their belongings from one lodging to another.

Witness #3 advised that bedbugs can live one full year without a blood meal. They are attracted to heat and carbon dioxide so they can live in floors, floor boards or furniture for up to a year and then appear at the feel and smell of humans. They mature in 21 days, lay 1 to 5 eggs per day and can produce up to 500 offspring in a lifetime.

Witness #3 was emphatic and unshakeable in his evidence that bedbugs do not generally move from one unit to another without a human carrier. He was also adamant that people do not need

to throw out their soft furnishings upon the happening of an infestation if the unit is treated properly which he feels his company is very capable of doing.

Finally, the Landlord led evidence through Witness #4 who is the Property Manager of this 750 unit building. She has held this position for 8 years and has herself been a tenant in the building for eleven (11) years. She testified that no previous tenant of this unit had reported a bedbug problem, and that the subsequent tenant, who moved in on December 1, 2008 and remains a tenant to this day, has also not had any problems. She candidly admitted that there have been units in the building with pest issues of this nature. She testified that her practise is to have every vacant unit in the building fogged for bugs whether or not a problem has been reported and that she followed this practise with this unit prior to the Tenant's occupation. She stated that in the past she has even called Public Health in an instance where a tenant has refused treatment of their unit when a problem became evident. That unit was not on this floor.

2008 CanLII 86704 (ON L.T.B.)

In regard to the Tenant's infestation, Witness #4 testified that the Tenant took possession on October 1, 2008, that a complaint was received on October 14 or 15 and that Magical Pest Control was called immediately. She testified that the Tenant never contacted the office again regarding dissatisfaction regarding the spray and she identified the course of correspondence between the parties in which the Tenant was advised that early termination of her tenancy was not acceptable.

The Law:

Counsel for the Tenant asked me to find that this unit was uninhabitable and asked me to follow the reasoning of courts in Manitoba and Nova Scotia in order to find for the Tenant and give her the relief sought. The cases provided by counsel for the Tenant are neither helpful nor binding.

Section 20 of the *Residential Tenancies Act, 2006* governs the circumstances of the parties before me. Under the provisions of Section 20 a landlord has the responsibility to both provide and maintain the residential complex in a good state of repair; and to provide and maintain the residential complex fit for habitation. The "fit for habitation" standard does not mean that the premises is literally impossible to live in; rather, the test will be whether the premises meets current standards of decent accommodation.

Section 20 establishes a rigorous standard, and imposes on a landlord a contractual obligation which is not dependent on negligence; however, once a violation of Section 20 has been determined the quantum of damages, if any are awarded, is to be determined in regard to the Landlord's conduct.

Determinations:

In my view, the Tenant's rental unit was not unfit for habitation. The photographic evidence led by the Tenant shows a clean, bright space with appropriate flooring and wall covering. There was no evidence led by the Tenant regarding the attendance of the Health Department of the City of Toronto. The Tenant's claim of unfitness for habitation stems solely from the bedbug infestation and I do not find it to be of such severity that, on the facts, it made the unit uninhabitable and warranted termination of the tenancy or the other relief sought.

2008 CanLII 86704 (ON L.T.B.)

Clearly, all of the evidence was consistent in establishing that there was a bedbug infestation in the unit in question. For the purposes of these reasons, I specifically decline to find that the Tenant brought the bedbugs into the unit with her. I find it just as likely that the previous tenant had bedbugs and did not disclose this to the building management. Based on the evidence of the pest control expert, I also find that even with one general fogging of the unit before the Tenant assumed occupancy it is possible that bedbugs already in the unit continued to reside there waiting for the next occupant.

As a result of these findings of fact, I conclude that the Landlord was in breach of its maintenance obligations to the Tenant. However, the un-contradicted evidence before me was that the Landlord took immediate steps to remedy the breach upon being informed of the bedbug problem. In my view, the steps taken by the Landlord were timely and appropriate.

Further, in my view, the steps taken by the Tenant were an over-reaction to a common and entirely resolvable problem. It was unnecessary for the Tenant to vacate the unit. It was also unnecessary for the Tenant to dispose of the vast quantity of her belongings.

It is for these reasons that I find the Tenant is not entitled to any of the compensation she has claimed for the Landlord’s breach of maintenance.

Regarding the claim for substantial interference, this claim is based on the identical set of facts as the maintenance claim and is properly a maintenance claim not an interference claim. As a result, the T2 is also dismissed.

It is ordered that:

1. The Tenant's applications are dismissed.

October 30, 2009
Date Issued

Christina Budweth Mingay
Member, Landlord and Tenant Board

Toronto South Region
2nd Floor, 79 St. Clair Ave. E
Toronto ON M4T 1M6

If you have any questions about this order, call 416-645-8080 or toll free at 1-888-332-3234.
