# Supplementary material for: Analysis of 44 Cases before the Landlord and Tenant Board Involving Bed Bug Infestations in Ontario, Canada: Focus on Adjudicator Decisions Based on Entomological/Pest Management Evidence and Accountability under the Residential Tenancy Act and Other Applicable Legislation
Source: Insects. 2011 Jul 19;2(3):343–53. doi: 10.3390/insects2030343 (PMC4553548; doi:10.3390/insects2030343)
Supplement: Supplementary File 1 [file insects-02-00343-s001.zip › supplementary-2/2009canlii01497.pdf]

Landlord and Tenant Board
Ontario

Order under Section 30
Residential Tenancies Act, 2006

File Number: TST-01497

C. H. and T. V.(the 'Tenants') applied for an order determining that D. A. (the 'Landlord') failed to meet the Landlord's maintenance obligations under the *Residential Tenancies Act, 2006* (the 'Act') or failed to comply with health, safety, housing or maintenance standards.

This application was heard in Toronto on June 23, 2008, August 1, 2008, September 22, 2008, and November 28, 2008.

The Tenants and the Landlord’s representative, D. C., attended the hearing.

Determinations:

1. The Tenant brought bedbugs into her apartment by taking a bedbug-infested headboard from the garbage disposal area and bringing it into her unit. While the Landlord is obligated to treat the unit to eradicate the bedbugs, the Landlord is not liable for any loss of property or suffering on the part of the Tenants.

2. In mid-February 2008, the Tenant requested repairs with respect to holes in the drywall and rotting wood under the sinks in the bathroom and kitchen, as well as a crack in a window in the unit. These repairs were not done before the tenancy was terminated on July 31, 2005.

3. Therefore, I find that the Landlord failed to meet the Landlord's obligations under subsection 20(1) of the Act to repair or maintain the rental unit and failed to comply with maintenance standards.

It is ordered that:

1. The Landlord shall pay to the Tenants a rent abatement of $250.00.

2. The Landlord shall also pay the Tenants $45.00 for the cost of filing the application.

3. The total amount the Landlord owes the Tenants is $295.00.

4. The Landlord shall pay the Tenants the full amount owing by February 15, 2009.

5. If the Landlord does not pay the Tenants the full amount owing by February 15, 2009 the Landlord will owe interest. This will be simple interest calculated from February 16, 2009 at 4.00% annually on the outstanding balance.

6. The Tenants have the right, at any time, to collect the full amount owing or any balance outstanding under this order.

February 4, 2009
Date Issued

____
Egya Sangmuah
Member, Landlord and Tenant Board

Toronto South Region
2nd Floor, 79 St. Clair Ave. E
Toronto ON M4T 1M6

If you have any questions about this order, call 416-645-8080 or toll free at 1-888-332-3234.

File Number: TST-01497

REASONS

Reasons to Order TST-01497 issued on February 4, 2009 by Egya Sangmuah.

The Tenants applied for an order determining that the Landlord failed to meet the Tenants' maintenance obligations under the Act or failed to comply with health, safety, housing or maintenance standards.

This application was heard in Toronto on June 23, 2008, August 1, 2008, September 22, 2008, and November 28, 2008.

The Tenants and the Landlord’s representative, D.C., attended the hearing.

Bedbugs

The Tenants alleged that the rental unit was infested with bedbugs as a result of lack of maintenance by the Landlord. They further alleged that the Landlord did not adequately address the problem when the Landlord was notified that there were bedbugs in the unit. The Tenants sought an abatement of rent, compensation for property that was disposed off, lost wages, and general damages in the amount of $5,000 for pain and suffering. The Landlord claimed the Tenant was responsible for bringing the bedbugs into the unit and that the Landlord’s response to the problem was adequate. The Landlord argued that it was not liable to the Tenant for any losses or pain and suffering on the part of the Tenant’s because the Tenants were the authors of their own misfortune.

The Tenants claimed that the bedbugs came into the unit from another part of the complex, along the pipes and through the hole under the kitchen sink in the unit and space between the walls and the floors of the unit. I find on a balance of probabilities that the female Tenant brought the bedbugs into the unit by bringing a bedbug infested headboard into the unit. This headboard was discarded by the resident of unit 807, which was treated for bedbugs on January 18, 2008. The Tenant salvaged the headboard from the garbage collection area. The Tenant did not dispute that she brought the headboard into the unit, but contended that it was not the source of the bedbugs because she cleaned the headboard and used it on the balcony of the unit. I find the Tenants’ theory of causation wanting. In the first place, no bedbugs were found in the envelope of apartments surrounding the rental unit. Secondly, it is a lot easier for bedbugs to enter the unit if they are brought in via a headboard. The Tenants began experiencing bite and rashes shortly after the headboard was brought in. It is pertinent to note that unit 807 was treated for bedbugs on January 18, 2008 and by the end of the first week of February 2008 the male Tenant saw his doctor for rash over his body that was later attributed to bedbugs. Furthermore, Ms. H. first testified that the bedbugs were first discovered on the headboard and bed frame in the bedroom as well as the baseboards in the bedroom. When she was further questioned about the headboard she changed her testimony to say that they did not use a headboard in the bedroom. It makes more sense that the Tenants did not have a headboard and therefore salvaged a

headboard from the garbage to use as a headboard. In light of the fact that the Tenants were the source of the bedbug problem, no compensation is warranted for their property loss or pain and suffering. They should not benefit from their mistake or wrong.

Although the Tenants brought the bedbugs into the unit, the Landlord is still responsible for treating the unit as part of its maintenance obligations and to prevent other units from infestation. In this regard, the Tenants alleged that Ms. H informed the Landlord's agents, the superintendent and assistant superintendent, of the bedbug problem on March 18, 2008, and that they told her to deal with the problem herself. Ms. H testified that she cleaned the unit with bleach to no avail, so she requested treatment by the Landlord's agents and the unit was treated on March 31, 2008. The Landlord's agents testified that Ms. H told them about the problem, but also stated that she said she was going to deal with it with bleach. They were not sure whether that would work, so the assistant superintendent did some research on the matter. They Landlord's agent also testified that Ms. H later requested treatment and they responded by requesting treatment by their pest control company on March 25, 2008, a week after they were first notified. I find the Landlord's agent to be credible on this issue. Ms H came across though her testimony and her conduct during the hearing as a person who likes to do things her own way, and who is hesitant to allow others entry into her unit. She wanted the pest control person to treat only one bedroom and was hesitant to leave the unit for the spraying to be done. Thus, it is credible that her initial response to the problem was to try to deal with it herself through cleaning.

The Tenants also sought compensation for bringing in their own contractor, Clean Air King Inc., to treat to unit on April 1, 2008 and May 24, 2008 with Surekill bug killer. The scope of the contract was to inspect for bedbugs, yeast, mould and fungal spores. The Tenant alleged that the Landlord's pest control contractor did not do a good job because he allegedly sprayed the unit in five minutes. It was not necessary to have the unit treated again a day after the Landlord treated the unit, as it takes some time to ascertain whether bed bug treatment has been effective. The Tenant did not wait to see if the treatment on March 31, 2008 was effective, and indeed broadened the scope to include investigating for yeast and mould. Furthermore, the Landlord's pest control contractor testified that Surekill bug killer is not licensed for treating bedbugs. With respect to the second treatment on May 24, 2008, the Tenants did not inform the Landlord that a second treatment was required and indeed when Clean Air King Inc. inspected the unit on May 24, 2008, they determined that the problem was contained and yet proceeded to spray the unit again. Ms. H also testified that Clean Air King Inc. does a follow up as matter of course, and thus the costs flow from the first treatment requested. Under the circumstances, it is not appropriate to reimburse the Tenants for expenses incurred in retaining Clean Air King Inc.

<u>Repairs</u>

Ms. H testified that in mid-February 2008, the Tenants requested repairs with respect to holes in the drywall and rotting wood under the sinks in the bathroom and kitchen, as well as a crack in a window in the unit. This is supported by a letter from the Landlord requesting keys to the unit in order to make repairs. These repairs were not done before the tenancy was terminated on July 31, 2005. The Landlord contended that the repairs were not done because the Tenants failed to provide the Landlord with keys. It was open to the Landlord to give notice to the Tenants, as the Landlord did with the bedbugs, so that the Tenants would either provide keys to the unit or be available to grant access to the unit. Therefore, I find that the Landlord failed to meet the

Landlord's obligations under subsection 20(1) of the Act to repair or maintain the rental unit and failed to comply with maintenance standards.

Ms. H testified that they were not really concerned about the lack of repairs until they came to believe that the bedbugs originated from the holes in the wall. I have provided reasons for rejecting this theory. The repairs required were not major and given the length of time the repairs went unaddressed, a lump sum abatement of $250.00 is appropriate.

February 4, 2009
Date Issued

____
Egya Sangmuah
Member, Landlord and Tenant Board

Toronto South Region
2nd Floor, 79 St. Clair Ave. E
Toronto ON M4T 1M6
