# Supplementary material for: Analysis of 44 Cases before the Landlord and Tenant Board Involving Bed Bug Infestations in Ontario, Canada: Focus on Adjudicator Decisions Based on Entomological/Pest Management Evidence and Accountability under the Residential Tenancy Act and Other Applicable Legislation
Source: Insects. 2011 Jul 19;2(3):343–53. doi: 10.3390/insects2030343 (PMC4553548; doi:10.3390/insects2030343)
Supplement: Supplementary File 1 [file insects-02-00343-s001.zip › supplementary-2/2009canlii067622.pdf]

Landlord and Tenant Board
Ontario

Amended Order
Order under Section 30
Residential Tenancies Act, 2006
And section 21.1 of the Statutory Powers Procedure Act

File Number: TST-02331
TST-01231-09

In the matter of: [*Address removed*]

Between: [*Tenant's name removed*] Tenant

and

[*Landlord's name removed*] Landlord

[*Tenant's name removed*] (the 'Tenant') applied for an order determining that [*Landlord's name removed*] (the 'Landlord') failed to meet the Landlord's maintenance obligations under the *Residential Tenancies Act, 2006* (the 'Act') or failed to comply with health, safety, housing or maintenance standards.

The Tenant also applied for an order determining that the Landlord' substantially interfered with the reasonable enjoyment of the rental unit or residential complex by the Tenant or by a member of her household.

The combined application was heard in Toronto on July 15, 2009 and December 10, 2009.

The Tenant and the Landlord's representative, [*Landlord Representative's name removed*], attended the hearings.

This amended order is issued to correct a clerical error in the original order issued on January 20, 2010.

Determinations:

1. On February 3, 2009, the Tenant confirmed that her rental unit was infested with bedbugs after inspecting the unit with the help of a friend, [*Tenant Friend's name removed*]. She immediately informed the Landlord's management office. The Tenant was provided with a preparation sheet for bedbug treatment and the Landlord arranged for the unit to be treated by its pest control contractor, [*Pest Control name removed*] Pest Control.

2. [*Pest Control name removed*] treated the rental unit for bedbugs on February 6, 2009, February 23, 2009, March 2, 2009, without success. On March 17, 2009, a Public Health Inspector inspected the unit and found that it was still infested with bedbugs. [*Pest Control name removed*] treated the unit again on March 24, 2009.

3. The Tenant was advised by a doctor to reside elsewhere until the bedbug infestation was resolved. The Tenant stayed with family and friends for most of the time until she moved into a new rental unit on April 1, 2009. She also spent 16 nights in hotels. On February 24, 2009 the Tenant gave notice to terminate her tenancy on April 30, 2009.

4. The Tenant left her belongings in the rental unit and on April 30, 2009, she authorized the Landlord to dispose of her belongings.

5. In light of the above, I find that the Landlord failed to meet the Landlord's obligations under subsection 20(1) of the Act to maintain the rental unit.

6. I also find that the Landlord substantially interfered with the reasonable enjoyment of the rental unit or residential complex by the Tenant or by a member of her household.

7. The Tenant is entitled to an abatement of rent in the amount of $1,215.28.

8. The Tenant is also entitled to compensation in the amount of $1,125.03, which is the reasonable out-of-pocket expense the Tenant has incurred for alternative accommodation, purchasing caulking materials, and the cost of a doctor’s note.

It is ordered that:

1. The Landlord shall pay to the Tenant a rent abatement of $1,215.28.

2. The Landlord shall pay to the Tenant $1,125.03 which is the reasonable out of pocket expenses the Tenant has incurred for alternative accommodation, purchasing caulking materials, and the cost of a doctor’s note.

3. The Landlord shall also pay the Tenant $45.00 for the cost of filing the application.

4. The total amount the Landlord owes is $2,385.31.

5. The Landlord shall pay the Tenant the full amount owing by January 31, 2010.

6. If the Landlord does not pay the Tenant the full amount owing by January 31, 2010 the Landlord will owe interest. This will be simple interest calculated from February 1, 2010 at 2.00% annually on the outstanding balance.

7. The Tenant has the right, at any time, to collect the full amount owing or any balance outstanding under this order.

January 20, 2010
Date Issued

March 8, 2010
Date Amended

____
Egya Sangmuah
Member, Landlord and Tenant Board

Toronto South Region
2nd Floor, 79 St. Clair Ave. E
Toronto ON M4T 1M6

If you have any questions about this order, call 416-645-8080 or toll free at 1-888-332-3234.

File Number: TST-02331
TST-01231-09

REASONS

In the matter of: [*Address removed*]

Between: [*Tenant's name removed*] Tenant

and

[*Landlord's name removed*] Landlord

Reasons to Order TST-02331 issued on January 20, 2010 by Egya Sangmuah.

[*Tenant's name removed*] (the 'Tenant') applied for an order determining that [*Landlord's name removed*] (the 'Landlord') failed to meet the Landlord's maintenance obligations under the *Residential Tenancies Act, 2006* (the 'Act') or failed to comply with health, safety, housing or maintenance standards.

The Tenant also applied for an order determining that the Landlord' substantially interfered with the reasonable enjoyment of the rental unit or residential complex by the Tenant or by a member of her household.

The combined application was heard in Toronto on July 15, 2009, and December 10, 2009.

The Tenant and the Landlord's representative, [*Landlord Representative's name removed*], attended the hearings.

Most of the facts in this case are not in dispute. The areas of dispute concern whether the Landlord should be held responsible for the Tenant's experience, whether an abatement of rent or other compensation is merited, and the extent to which the Tenant should have mitigated her losses.

At the end of January 2009, the Tenant's ankles were itchy and she discovered rashes on her forearms, lower legs, and ankles. On February 3, 2009, the Tenant confirmed that her rental unit was infested with bedbugs after inspecting the unit with the help of a friend, [*Tenant Friend's name removed*]. She immediately informed the Landlord's management office. The Tenant was provided with a preparation sheet for bedbug treatment and the Landlord arranged for the unit to be treated by its pest control contractor, [*Pest Control name removed*] Pest Control.

[*Pest Control name removed*] treated the rental unit for bedbugs on February 6, 2009, February 23, 2009, March 2, 2009, without success. On March 17, 2009, a Public Health Inspector

inspected the unit and found it still infested with bedbugs. [*Pest Control name removed*] treated the unit again on March 24, 2009.

The bites from the bedbugs continued after the first treatment of the unit, so the Tenant saw a doctor, who diagnosed her as suffering from bedbug bites. The doctor advised the Tenant to reside elsewhere until the bedbug infestation was resolved. The Tenant stayed with family and friends for most of the time until she moved into a new rental unit on April 1, 2009. She also spent 16 nights in hotels. On February 24, 2009 the Tenant gave notice to terminate her tenancy on April 30, 2009. The Tenant left her belongings in the rental unit and on April 30, 2009, she authorized the Landlord to dispose of her belongings.

Relying on *McQuestion v. Schneider* (1975) 8 O.R. (2d) 249 (C.A.), the Landlord contended that the Landlord should not be held liable because the bedbug infestation in the unit was a latent defect in that the bedbugs were not visible and the Landlord was unaware of the infestation. The decision in *McQuestion v. Schneider* does not assist the Landlord. The bedbug infestation was not a latent defect. The bedbugs were visible on careful inspection and in any event the Landlord was required to the address the infestation once the Tenant informed the Landlord about the problem.

In my view, while the Landlord responded promptly, the treatments of the unit were ineffective. The treatments were doomed to fail because [*Pest Control name removed*] did not identify the source of the bedbugs. The [*Pest Control name removed*] technician reported bedbugs in a stereo box and no where else The Tenant and [*Tenant Friend's name removed*] testified that apart from the Tenant's box spring, bed bugs were concentrated behind a radiator, clustered around a hole in the ceiling of the bedroom near the window and on the walls of the bedroom closet. The location of the bedbugs indicated that they were coming from another part of the complex through holes in the walls and openings for mechanical systems. Nonetheless, the [*Pest Control name removed*] technician did not detect bed bugs in the block units. His failure to detection the locations of the bedbugs in the Tenant's unit does not inspire confidence in his ability to detect bedbugs in the block units. The Landlord did not take the precaution of having the block units treated for bedbugs. Indeed, the pest control reports introduced at the hearing show that the Tenant's unit was not the only unit in the complex infested with bedbugs and, as in the case of the Tenant's unit, the Landlord chose the treat the other infested units in isolation. Thus, there was an ongoing infestation in units of the complex that the Landlord's response was inadequate. It is not unusual to have to do a second treatment to eradicate bedbugs. In this case, the unit was determined to be bedbug free in June 2009, months after the Landlord was notified of the problem.

It goes without saying that the tenant is entitled to reasonable enjoyment of her unit and that the bedbug infestation that the Landlord failed to prevent or respond effectively to substantially interfered with the Tenant's reasonable enjoyment of her unit. She was subjected to bedbug bites and had to reside elsewhere because a doctor advised her to avoid the rental unit until the infestation was cleared.

In light of the finding above, the Tenant is entitled to an abatement of rent from February 3, 2009 to March 31, 2009 in the amount of $1,215.28. The unit was essentially not fit for habitation and on the advice of a doctor the Tenant did not reside in the unit until she found new accommodation on April 1, 2009. The abatement is therefore 100% of the rent minus per diem compensation for

the days the Tenant stayed in a hotel (to avoid double recovery), as the Landlord is required to reimburse the Tenant for the cost of staying in hotels. The Tenant mitigated her hotel costs by staying with family and friends for most of the period in question. However, the Tenant left her belongings in the unit and did not give vacant possession to the Landlord until April 30, 2009, when she authorized the Landlord to dispose of her possessions. By March 17, 2009, the day the Health Inspector confirmed the continuing bedbug infestation it was reasonable for the Tenant to terminate her tenancy forthwith and on March 20, 2009 she signed a tenancy agreement with a new Landlord with the tenancy commencing on April 1, 2009. To mitigate her losses and to allow the Landlord to mitigate its losses the Tenant should have given vacant possession of the unit to the Landlord on March 31, 2009. Consequently, the abatement of rent awarded does not apply to the rent for April 2009.

The Tenant is also entitled to **compensation in the amount** of $1,125.03 for expenses incurred as a result of the infestation, namely hotel costs ($1085.03), caulking materials to seal baseboards ($20.00), and the cost of a doctor's note ($20.00). The Tenant did not claim compensation for the items left in the rental unit, but she claimed compensation for a bed, clothing and linens allegedly damaged by the bedbug infestation, totalling $2,500. I am not persuaded that those items could not have been salvaged by bedbug treatment and laundering, therefore, the Tenant is not entitled to compensation for those items. I have also denied the Tenant's claim for lost wages, as I am not satisfied that she had to absent from work as a result of the bedbug infestation. The doctor's note does not mention the need for time off work.

January 20, 2010
Date Issued

March 8, 2010
Date Amended

____
Egya Sangmuah
Member, Landlord and Tenant Board

Toronto South Region
2nd Floor, 79 St. Clair Ave. E
Toronto ON M4T 1M6
