# Supplementary material for: Analysis of 44 Cases before the Landlord and Tenant Board Involving Bed Bug Infestations in Ontario, Canada: Focus on Adjudicator Decisions Based on Entomological/Pest Management Evidence and Accountability under the Residential Tenancy Act and Other Applicable Legislation
Source: Insects. 2011 Jul 19;2(3):343–53. doi: 10.3390/insects2030343 (PMC4553548; doi:10.3390/insects2030343)
Supplement: Supplementary File 1 [file insects-02-00343-s001.zip › supplementary-2/2009canlii19858.pdf]

Landlord and Tenant Board
Ontario

Order under Sections 30, 31 and 69
Residential Tenancies Act, 2006

File Number: TET-01678
and **File Number:** TET-01760
and **File Number:** TEL-19858

E.S. (the 'Tenant') applied for an order determining that M.G. (the 'Landlord') or the Landlord's superintendent or the Landlord's agent harassed, obstructed, coerced, threatened or interfered with him and substantially interfered with the reasonable enjoyment of the rental unit or residential complex by the Tenant or by a member of his household (T2).

The Tenant also applied for an order determining that the Landlord failed to meet the Landlord's obligations under the Residential Tenancies Act, 2006 (the 'Act') or failed to comply with health, safety, housing or maintenance standards (T6).

The Landlord applied for an order to terminate the tenancy and evict the Tenant because the Tenant did not pay the rent that he owes (L1).

These three applications were heard in Toronto on January 28, 2009.

The Tenant and the Landlord attended the hearing. The Landlord was represented by P.M..

Relevant evidence and facts:

a. This tenancy began in November 2003.

b. The lawful rent is $385.00 per month.

c. The Tenant produced a receipt for November 2008 rent which the Landlord claimed was an altered receipt. No compelling evidence was entered to prove this allegation and I accept the receipt as 'best evidence'. The Landlord brought a witness who signed an affidavit stating the Tenant bragged about altering a 2007 receipt to look like 2008, however when asked directly by the Member while under oath, "did E.S. tell you he changed this receipt?" He responded, "No".

d. December rent was paid by bank draft cashed December 9, 2008. The N4 notice gave the Tenant until December 13, 2008 to pay the arrears and void the notice.

e. The date of payment is earlier than the date on the N4 notice.

f. The Landlord filed the L1 application on December 22, 2008.

g. The Tenant's T2 and T6 applications are based on the same issue of bed bugs and the substantial interference they caused with the Tenant's enjoyment of his rental unit.

h. The Tenant raised some maintenance issues that the Landlord had addressed immediately. The Tenant mentioned cockroaches and rodents, but had no compelling evidence of their presence.

i. The Tenant adduced a number of letters he asserted were sent to the Landlord advising him of the presence of bed bugs beginning April 3, 08 and continuing through November 12, 2008.

j. The Tenant adduced a number of receipts to show that he spent $220.00 of his own money on purchasing sprays for repeated attempts at debugging his unit.

k. The Landlord argued that he has a monthly service from PCO to visit and spray the entire building. However, one of the Landlord’s witnesses testified that he had to spray repeatedly in his own unit because the bed bugs just kept showing up in his unit.

l. The Landlord adduced a record of a PCO visit November 28, 2008 when the Tenant refused entry. No was other evidence or record of any other attempts to address this Tenant’s problem.

Determinations:

1. I find the Tenant’s evidence of a receipt for November 2008 rent is acceptable.

2. Based on the evidence heard, I find the Tenant had voided the N4 notice when he made the payment on December 9, 2008 therefore the L1 application brought December 22, 2008 is without cause and must be dismissed.

3. The Landlord made repeated attempts to collect money from the Tenant that was not owed. Therefore, I find that the Landlord or the Landlord's superintendent or the Landlord's agent harassed, obstructed, coerced, threatened and interfered with the Tenant and substantially interfered with the reasonable enjoyment of the rental unit or residential complex by the Tenant or by a member of his household. In this regard a rent rebate of 10% for the 9 months since the Landlord was notified totalling $346.50 is awarded.

4. I find that the Landlord failed to maintain the rental unit as required by the Residential Tenancies Act, 2006 and as such allowed the spread of bed bugs in the complex. I find the Tenant’s request for reimbursement of money spent on spray is warranted and reasonable and so award in the amount of $220.00.

It is ordered that:

1. The Landlord’s application TEL-19858 is dismissed.

2. The Landlord shall pay to the Tenant $220.00 which is the reasonable out of pocket expenses the Tenant has incurred for bed bug sprays.

3. The Landlord shall also pay the Tenant $346.50 as a rent abatement for interference with his reasonable enjoyment of the rental unit.

4. The Landlord shall also pay the Tenant $45.00 for the application filing fee.

5. The total amount the Landlord owes is $611.50.

6. The Landlord shall pay the Tenant the full amount owing by February 10, 2009.

7. If the Landlord does not pay the Tenant the full amount owing by February 10, 2009, he will owe interest. This will be simple interest calculated from February 11, 2009 at 4.00% annually on the balance outstanding.

8. If the Landlord does not pay the Tenant the full amount owing by February 10, 2009, the Tenant may recover this amount by deducting $205.75 from the rent in March and April and $200.00 from the rent for the month of May 2009.

9. The Tenant has the right, at any time, to collect the full amount owing or any balance outstanding under this order.

January 30, 2009
Date Issued

____
Gerald Taylor
Member, Landlord and Tenant Board

Toronto East/Durham Region
Unit # 2, 2275 Midland Avenue
Toronto ON M1P 3E7

If you have any questions about this order, call 416-645-8080 or toll free at 1-888-332-3234.
