# Supplementary material for: Analysis of 44 Cases before the Landlord and Tenant Board Involving Bed Bug Infestations in Ontario, Canada: Focus on Adjudicator Decisions Based on Entomological/Pest Management Evidence and Accountability under the Residential Tenancy Act and Other Applicable Legislation
Source: Insects. 2011 Jul 19;2(3):343–53. doi: 10.3390/insects2030343 (PMC4553548; doi:10.3390/insects2030343)
Supplement: Supplementary File 1 [file insects-02-00343-s001.zip › supplementary-2/2009canlii78053.pdf]

Landlord and Tenant Board

Ontario

Order under Section 30 & 69
Residential Tenancies Act, 2006

2009 CanLII 78053 (ON L.T.B.)

File Number: SWT-02146 & SWL-27484

DT (the 'Tenant') applied for an order determining that LCSC (the 'Landlord') failed to meet the Landlord's maintenance obligations under the *Residential Tenancies Act, 2006* (the 'Act') or failed to comply with health, safety, housing or maintenance standards.

LCSC (the 'Landlord') applied for an order requiring DT (the 'Tenant') to pay compensation for the damages caused by her or a person she permitted in the residential complex

This application was heard in Windsor on August 19, 2009 in the presence of the Tenant and JW, the Landlord Representative.

BB, Legal Assistance Windsor, represented the Tenant.
JB, Solicitor, represented the Landlord.

PC, friend, attended as a witness for the Tenant.
PB, Complex Coordinator, attended as a witness for the Landlord.

For the reasons attached, it is determined that:

1. The Tenant has not wilfully or negligently caused undue damage to the rental unit.

2. The Landlord has not failed to meet the Landlord's obligations under subsection 20(1) of the Act to repair and maintain the rental unit.

It is ordered that:

1. The Tenant’s application is dismissed.

2. The Landlord's application is dismissed.

September 18, 2009
Date Issued

Kim Bugby
Vice Chair, Landlord and Tenant Board

SouthWest Region
4th floor, 150 Dufferin Avenue
London ON N6A 5N6

If you have any questions about this order, call 416-645-8080 or toll free at 1-888-332-3234.

Landlord and Tenant Board
Ontario

File Number: SWT-02146 & SWL-27484

2009 CanLII 78053 (ON L.T.B.)

REASONS

Reasons to Order SWL-27484 issued on September 18, 2009 by Kim Bugby.

Undisputed Facts:

1. The facts of the case were largely undisputed.

2. The tenancy commenced in February 2007 and is ongoing.

3. The Tenant noted the presence of bedbugs in the unit in October 2008, 21 months after commencement of the tenancy.

4. The Landlord was informed of the issue on October 14, 2008 and the unit was treated on October 23, 2008.

5. On October 22, 2008, the Tenant signed an agreement stating that she would repay the Landlord for the full cost of treating the rental unit.

6. The issue recurred in January 2009 and the Landlord arranged for treatment of the unit which was completed on January 30, 2009.

SWT-02146:

7. In her application, the Tenant seeks an abatement of rent in consideration of the impact of the bedbugs and compensation for possessions disposed of due to the issue.

8. Section 20.(1) of the Act provides:

   "A landlord is responsible for providing and maintaining a residential complex, including the rental units in it, in a good state of repair and fit for habitation and for complying with health, safety, housing and maintenance standards."

9. Section 30.(1), which speaks to the remedies requested by the Tenant, provides:

   *"**If** the Board determines in an application under paragraph 1 of subsection 29 (1) that a landlord has breached an obligation under subsection 20 (1) or section 161, the Board may do one or more of the following:"…*(emphasis added)

10. Accordingly, while the Tenant was adversely affected by the presence of bedbugs and may have disposed of possessions as a result of the issue, the first question before the Board is whether the Landlord breached its obligation to repair or maintain the rental unit.

Pursuant to s. 30 of the Act, remedy is available to the Tenant only **if** the Board determines that the Landlord is in breach of its obligations under s. 20.

11. In determining whether the Landlord has breached its obligation to repair and maintain, the Board must first consider whether a maintenance or repair issue existed. In this case there was no dispute that the unit was subject to a bed bug infestation which required professional treatment.

12. Next the Board must consider when the Landlord was informed of the issue or should reasonably have been aware of the issue. In this case, there is no reasonable basis to conclude that the Landlord should have been aware that an issue existed prior to notice from the Tenant. The tenancy was 21 months old and there were no issues with bedbugs in the unit prior to October 2009. While there was one unit in the complex which required treatment for bedbugs, that unit was not adjacent to the Tenant's unit and in fact, was in a separate row of town homes located in the same complex.

13. Accordingly, the Board must consider the Landlord's response to the issue once they were informed by the Tenant. In this case, the Landlord was informed of the issue on October 14, 2009 and treatment of the unit occurred on October 23, 2009, 9 days after the Landlord was informed of the issue. Given the nature of the issue, the fact that the Landlord was dependant on the availability of a third party to affect treatment and the need to allow the Tenant time to prepare the unit for treatment; I find the Landlord's response to issue to be reasonable and timely.

14. The Tenant argued that the Landlord breached its obligation to repair by requiring the Tenant to agree, in writing, that she would cover the costs of the treatment. The Tenant gave evidence that, although not implicitly stated, she felt that treatment of the unit was contingent upon her agreement to repay the costs of the treatment. The Landlord denied that such was the case.

15. Treatment of the unit occurred one day after the Tenant signed the agreement to repay the costs incurred by the Landlord. The Tenant's evidence, that she was given and required three days to prepare the unit prior to treatment, is therefore more consistent with the Landlord's claim that they immediately arranged for treatment prior to and without promise of payment by the Tenant.

16. I find on a balance of probabilities, that while the Landlord did request that the Tenant repay the amount, that treatment of the unit was not conditional upon the Tenant's agreement. As such, the Landlords' request for repayment does not constitute a breach of its obligations.

17. Having responded to the issue in a reasonable period of time to appropriately address the issue, the Landlord cannot be deemed to have breached its obligations.

18. Even were I to accept that the Landlords' responsibilities were so absolute that any maintenance or repair issue which arose would constitute a breach of the Landlords' maintenance obligations, the outcome with respect to remedy would be no different.

2009 CanLII 78053 (ON L.T.B.)

2009 CanLII 78053 (ON L.T.B.)

19. In determining whether a requested remedy is warranted, the Board must take into consideration a number of factors, not least of which is the Landlord's response to the situation. In this case the Landlord acted in a reasonable and timely manner and could have taken no other action which would have mitigated the Tenant's losses. As such, the remedies requested by the Tenant would not, in any event, be appropriately considered.

20. Based on the foregoing, I find that the Landlord has not breached its obligations to repair and maintain the rental unit and the Tenant's application is dismissed. Accordingly, the remedies requested by the Tenant are not appropriately considered.

SWL-27487:

21. In SWL-24287, the Landlord requested compensation for the costs of twice treating the rental unit for a bedbug infestation.

22. Section 34 of the Act provides:

> *"The tenant is responsible for the repair of undue damage to the rental unit or residential complex caused by the wilful or negligent conduct of the tenant, another occupant of the rental unit or a person permitted in the residential complex by the tenant."*

23. Section 89.(1), which speaks to the remedy requested by the Landlord, provides:

> *"A landlord may apply to the Board for an order requiring a tenant to pay reasonable costs that the landlord has incurred or will incur for the repair of or, where repairing is not reasonable, the replacement of damaged property, if the tenant, another occupant of the rental unit or a person whom the tenant permits in the residential complex wilfully or negligently causes undue damage to the rental unit or the residential complex and the tenant is in possession of the rental unit."*

24. As with the Tenant application, while there is no dispute that the Landlord incurred costs to treat the rental unit, pursuant to s. 89, remedy is available to the Landlord only **if** the Board finds that the Tenant wilfully or negligently caused undue damage to the rental unit.

25. Pursuant to s. 89 the Landlord may apply for compensation for damages. However, the costs incurred to remedy an issue, while potentially damages in other jurisdictions, are not damages for the purpose of this Act. Under the Act, damage relates to physical damage to the rental unit rather than financial losses.

26. Therefore, the first question before the Board is whether the presence of bedbugs in the unit constitutes "*undue damage to the rental unit*".

27. The presence of bedbugs is not actual physical damage to the unit. However, the Landlord is charged with providing a rental unit "*in a good state of repair and fit for habitation and for complying with health, safety, housing and maintenance standards*." A bedbug infestation, if untreated, would potentially render the unit unfit for habitation and

not in compliance with health, safety, housing and maintenance standards. Further, the presence of bedbugs falls beyond the scope of normal wear and tear or expected deterioration from normal use of a unit. As such, I find that the presence of bedbugs does constitute undue damage to the rental unit.

2009 CanLII 78053 (ON L.T.B.)

28. Given that finding, the Board must consider if the undue damage is a result of wilful or negligent conduct on the part of the Tenant.

29. There was no allegation that the Tenant wilfully caused damage to the rental unit.

30. Therefore, the Board must consider whether the Tenant negligently caused undue damage to the rental unit.

31. There were several theories presented by both parties regarding how the bedbug infestation arose in the Tenant's unit. However, those theories were based in speculation and there was no evidence to lend greater weight to one over another.

32. What the evidence does support is a finding, a balance of probabilities, that the bedbug infestation originated with the Tenant, her guests or possessions which she placed in the rental unit. The Tenant had resided in the unit for a period of approximately 20 months prior to the issue arising and no adjacent units were subject to an infestation. Accordingly, it is less likely that the bedbug infestation predated the tenancy or was transferred from another unit in the complex.

33. However, that finding does not, in itself, lead to a finding that the bedbug infestation or the need for subsequent treatment arose from negligence on the part of the Tenant.

34. To find that the Tenant negligently caused undue damage to the rental unit, the Board must consider whether the issue resulted from some action taken by the Tenant which she should reasonably have known would result in the infestation, or some lack of action which the Tenant should reasonably have known would result in damage.

35. In this case, there is no basis to conclude that the Tenant took any action which she should reasonably have foreseen would result in a bedbug infestation. Whether the infestation was transferred to the unit by guests, family members, friends of her children or some other means, on balance, it occurred through normal activity in which a reasonable person would not anticipate the issue arising.

36. Likewise, the Tenant cannot be found to have exacerbated the issue through any action or inaction. The Tenant immediately reported the issue to the Landlord, diligently completed three days worth of onerous preparation of the unit for treatment and fully cooperated with respect to access to the unit for the purpose of treatment.

37. Based on the foregoing, the Tenant has not wilfully of negligently caused undue damage to the rental unit and the Landlord's application is dismissed.

<u>September 18, 2009</u>
Date Issued

Kim Bugby
Vice Chair, Landlord and Tenant Board

SouthWest Region
4th floor, 150 Dufferin Avenue
London ON N6A 5N6

2009 CanLII 78053 (ON L.T.B.)
