# Supplementary material for: Analysis of 44 Cases before the Landlord and Tenant Board Involving Bed Bug Infestations in Ontario, Canada: Focus on Adjudicator Decisions Based on Entomological/Pest Management Evidence and Accountability under the Residential Tenancy Act and Other Applicable Legislation
Source: Insects. 2011 Jul 19;2(3):343–53. doi: 10.3390/insects2030343 (PMC4553548; doi:10.3390/insects2030343)
Supplement: Supplementary File 1 [file insects-02-00343-s001.zip › supplementary-2/2009canlii78485.pdf]

Landlord and Tenant Board

Order under Section 30
Residential Tenancies Act, 2006

File Number: SOT-02439

2009 CanLII 78485 (ON L.T.B.)

G.R. (the 'Tenant') applied for an order determining that C.H.H.C. (the 'Landlord') failed to meet the Landlord's maintenance obligations under the *Residential Tenancies Act, 2006* (the 'Act') or failed to comply with health, safety, housing or maintenance standards.

This application was heard in Hamilton on September 17, 2009, where it was adjourned on consent. The merits of this application were heard in Hamilton on October 8, 2009.

The Tenant and the Landlord's representative, S.C., attended the hearing. H.B. also attended the hearing as a witness for the Landlord.

Reasons are attached.

Determinations:

1. The Landlord did not fail to meet the Landlord's maintenance obligations under the *Residential Tenancies Act, 2006* (the 'Act'), nor failed to comply with health, safety, housing or maintenance standards, with regard to the bed bug infestation in the rental unit.

It is ordered that:

1. The Tenant's application is dismissed.

<u>October 28 2009</u>
Date Issued

Bittu George
Member, Landlord and Tenant Board

Southern Region
6th floor, 119 King Street West
Hamilton ON L8P 4Y7

If you have any questions about this order, call 416-645-8080 or toll free at 1-888-332-3234.

REASONS

2009 CanLII 78485 (ON L.T.B.)

Reasons to Order SOT-02439 issued on October 28 2009 by Bittu George.

Testimony and evidence:

1. The Tenant requested that the consent of the Board be granted to amend his application to provide the correct name of the Landlord, state that he first moved into the unit on April 1, 2008, and amended the quantum and type of remedies requested in his application. The Landlord's representative did not object to these amendments. The Board granted the Tenant's request to amend his application.

2. The Tenant testified that he was aware that there was a bed bug problem in the residential complex prior to moving in to the rental unit. The Tenant testified that he first became aware of the bed bug infestation in the unit in June 2008. The Tenant also testified that he first informed the Landlord about the bed bug infestation on June 15 or 16, 2008. The Tenant testified that the Landlord's representative has seen the bed bugs.

3. The Landlord's representative informed the Board that she has never entered the Tenant's unit, and that the first time she saw the bed bugs was in a jar that the Tenant brought with him to the first hearing on September 17, 2009. The Landlord's representative acknowledged that there was a bed bug infestation in the unit, but that it has been addressed. The Landlord's representative informed the Board that the Landlord was first notified by the Tenant regarding the bed bug infestation in early Sept 2008. The Landlord's representative informed the Board that the Landlord applied the first treatment to the unit on September 11, the second treatment on October 20, and the third treatment on November 27, 2008. The Landlord's representative stated that the Landlord provided the Tenant with a letter confirming the bed bug treatment to allow the Tenant to obtain community start up funds from Ontario Disability Support Program (ODSP).

4. The Landlord's representative stated that the Tenant did not notify the Landlord of any further bed bug infestation from November 27, 2008 until May 2009. The Landlord then began a second round of treatments, the first on May 25, the second on June 24 and the third on September 14, 2009. The Landlord attempted to inspect the unit on September 30, 2009, but the Tenant refused entry. The Tenant did allow entry on October 5, 2009, and no bed bugs were present. The Landlord's representative also stated that G.S.W. carried out the bed bug treatments. The Landlord's representative informed the Board that it would cost the Landlord $580.00 to treat each unit, and $6,300.00 to treat each hallway. The Landlord's representative stated that the residential complex has 350 unit and 23 floors. The Landlord's representative also stated that the tenants are informed about how to prepare the unit for treatment, not to bring items into the residential complex in bulk garbage bins, and are provided a room to stay in if their unit is treated for free. The Landlord's representative stated that the Tenant did not make a written request to transfer to another unit due to the bed bug

infestation. The Landlord’s representative also stated that no government authority has confirmed the Tenant’s allegation that the unit is still infected with bed bugs.

5. H.B. provided sworn testimony at the hearing. H.B. stated that she is the Director of G.S.W. and has knowledge of the bed bug treatment applied to the Tenant’s unit. She confirmed the dates of treatment applied to the unit as stated in paragraph 3 above. The witness provided an overview of G.S.W.’s procedures on treating a unit for bed bug infestation. The Landlord’s representative submitted a copy of documentation regarding the bed bug treatment process for the Tenant’s unit, which was confirmed by H.B. H.B. stated that G.S.W. had been in the Tenant’s unit 5 times for bed bug treatment. H.B. testified that G.S.W. attempted to prepare the unit for a second treatment on September 14, 2009. The Tenant refused G.S.W. to remove items or steam clean the wall unit in the living room and the china cabinet in another room. The Tenant also refused G.S.W. to enter his closet. The Tenant did permit G.S.W. to remove the couch to be disposed. H.B. also testified that the Tenant refused G.S.W. on September 30, 2009 to prepare the unit for 2nd treatment to the rental unit. No visible bed bug activity was seen on September 30, 2009. G.S.W. returned to the unit on October 5, 2009 to prepare the unit for 2nd treatment, but the Tenant refused entry to the unit. The Tenant told G.S.W. that day that he still had bed bugs in the unit. H.B. testified that G.S.W. is treating 107 of the 350 units in the residential complex for bed bug infestation. H.B. testified that the Tenant’s request for the entire residential complex to be treated is not necessary because the entire residential complex had been inspected, and there was only evidence of a bed bug infestation in 107 units.

6. Under cross examination by the Tenant, H.B. testified that there is no treatment to prevent bed bugs, only treatment after the bed bugs has been detected. As well, H.B. stated that frequent treatment is not advisable, since this would allow bed bugs to develop immunity to the treatment. Regarding the removal of carpets, H.B. stated that it is not done since bed bugs require blood meal to survive, and there is no source of blood meals in carpeting.

7. The Tenant testified that he made a request to be transferred to another unit that was free of bed bugs, but that he was denied. The Landlord’s representative stated that if the Tenant had made such a request, the Tenant would have received a formal denial letter, which outlines the appeal process that a Tenant may use to overturn a refusal to transfer the Tenant to another unit. The Landlord’s representative stated that she has no knowledge of the Tenant making such a request to the Landlord.

8. In summation, the Tenant requested an order from the Board directing the Landlord to reimburse the Tenant for his personal items that were disposed of due to bed bugs. The Landlord’s representative stated that the Tenant signed a waiver authorizing any disposal of his belongings, and acknowledging that the Landlord is not liable for reimbursing the Tenant. Otherwise, the Tenant would have signed a waiver refusing the disposal of his personal items and confirming that the Landlord is not liable for any bed bug infestation of those items. The Landlord’s representative stated that there is no evidence that the Landlord caused the bed bug infestation; otherwise the Landlord would be liable for the Tenant’s claim for compensation. The Landlord’s representative stated that the Tenant was aware of a bed bug problem in the residential complex prior to moving in, and still chose to do so. The Landlord is undertaking all reasonable efforts to address this problem, but has not violated subsection

2009 CanLII 78485 (ON L.T.B.)

20(1) of the Act. The Landlord's representative also referred to Orders SOT-01370 and SOT-01460 as precedents on this issue.

Determinations:

9. The Tenant's application alleged that the Landlord failed to comply with subsection 20(1) of the *Residential Tenancies Act, 2006*, which states:

Landlord's responsibility to repair

20. (1) A landlord is responsible for providing and maintaining a residential complex, including the rental units in it, in a good state of repair and fit for habitation and for complying with health, safety, housing and maintenance standards.

I find that, based on the evidence presented, that the Landlord has not failed their obligations under subsection 20(1) of the Act. The Tenant did not present evidence that, on a balance of probabilities, the Landlord was at fault for the bed bug infestation. The Tenant believes that the Landlord should be held to strict liability standard, but that is not appropriate in this circumstance. The Landlord is undertaking all reasonable efforts to remedy a situation that it did not create.

10. Also, Section 8 of *Ontario Regulation 516/06* outlines the steps that a Landlord must comply with when undertaking repairs that would interfere with the reasonable enjoyment of a tenant. The *Regulation* states that if the landlord acts reasonably and professionally in carrying out repairs and gives tenants reasonable notice of the work then the landlord will not be liable for the disruption caused by the repairs. The Tenant did not provide evidence that the Landlord was in contravention of this *Regulation*.

11. While I find that the Landlord is not liable for the lost belongings of the Tenant due to the bed bug infestation, I will note that the Tenant has obligations too in regard to this situation. Section 16 of the Act states:

Minimize losses

16. When a landlord or a tenant becomes liable to pay any amount as a result of a breach of a tenancy agreement, the person entitled to claim the amount has a duty to take reasonable steps to minimize the person's losses.

The Tenant has an obligation to undertake all reasonable steps to minimize any loss regarding a bed bug infestation. The Landlord's evidence indicates that on a few occasions, the Tenant either did not permit entry to the unit, permit the disposal or treatment of some personal belongings, or did not permit the treatment of the unit. The Landlord tendered evidence showing that the cooperation of tenants is vital for the Landlord to be able to treat a bed bug infestation effectively. If a tenant fails to do this, they are contributing to the problem, rather than assisting the Landlord in remedying it.

2009 CanLII 78485 (ON L.T.B.)

October 28 2009
Date Issued

Bittu George
Member, Landlord and Tenant Board

Southern Region
6th floor, 119 King Street West
Hamilton ON L8P 4Y7

2009 CanLII 78485 (ON L.T.B.)
