# Supplementary material for: Analysis of 44 Cases before the Landlord and Tenant Board Involving Bed Bug Infestations in Ontario, Canada: Focus on Adjudicator Decisions Based on Entomological/Pest Management Evidence and Accountability under the Residential Tenancy Act and Other Applicable Legislation
Source: Insects. 2011 Jul 19;2(3):343–53. doi: 10.3390/insects2030343 (PMC4553548; doi:10.3390/insects2030343)
Supplement: Supplementary File 1 [file insects-02-00343-s001.zip › supplementary-2/2009canlii79045.pdf]

Landlord and Tenant Board

Ontario

Order under Section 31
Residential Tenancies Act, 2006

File Number: TET-00735-09

2009 CanLII 79045 (ON L.T.B.)

D. P. and J.P. (the 'Tenants') applied for an order determining that P.M. (the 'Landlord') the Landlord's agent substantially interfered with the reasonable enjoyment of the rental unit or residential complex by the Tenants or by a member of their household.

This application was heard in Toronto on October 26, 2009. The Tenants and the Landlord's Representatives, Property Managers, A.A. and R.L. attended the hearing.

Service:

At the hearing, it was determined that the Tenants personally delivered the hearing notice and application to the Landlord; and the service was deemed to be proper.

For the reasons attached to this order,

It is determined that:

1. The Landlord **or** the Landlord's agent substantially interfered with the reasonable enjoyment of the rental unit or residential complex by the Tenants or by a member of their household, by failing to effectively address the Tenants' complaints about bed bug infestation.

2. The problem was severe enough to warrant termination of the tenancy, and rent abatement, compensation as set out in this order.

It is ordered that:

1. The tenancy between the Landlord and the Tenants is terminated effective, October 31, 2009. The Landlord is entitled to a daily rate of $32.88, for the use of the unit for each day that the Tenants remain in the unit after the termination date.

2. The Landlord shall pay to the Tenants a rent abatement of $600.00.

3. The Landlord shall pay to the Tenants $300.00 which is the estimated reasonable moving expenses; and $50.00 for out of pocket expenses the Tenants have incurred bed bug sprays/treatment.

4. The total amount the Landlord owes is $950.00

5. The Landlord shall pay the Tenants the full amount owing by November 27, 2009.

6. If the Landlord does not pay the Tenants the full amount owing by November 27, 2009, the Landlord will owe interest. This will be simple interest calculated from November 28, 2009 at 2.00% annually on the balance outstanding.

2009 CanLII 79045 (ON L.T.B.)

November 16, 2009
Date Issued

Claudette Leslie
Member, Landlord and Tenant Board

Toronto East-RO
2275 Midland Avenue, Unit 2
Toronto ON M1P3E7

If you have any questions about this order, call 416-645-8080 or toll free at 1-888-332-3234.

File Number: TET-00735-09

REASONS

2009 CanLII 79045 (ON L.T.B.)

D. P. and J.P. (the 'Tenants') applied for an order determining that P.M. (the 'Landlord') the Landlord's agent substantially interfered with the reasonable enjoyment of the rental unit or residential complex by the Tenants or by a member of their household.

This application was heard in Toronto on October 26, 2009. The Tenants and the Landlord’s Representatives, Property Managers, A.A. and R.L.) attended the hearing.

Service:

At the hearing, it was determined that the Tenants personally delivered the hearing notice and application to the Landlord; and the service was deemed to be proper.

Reasons to Order TET-00735-09 issued on November 16, 2009 by Claudette Leslie.

1. The unit is located in a multi-unit complex. The Tenants moved into the unit in December of 2008. The Tenants gave evidence that in May of 2009, they noticed bugs crawling up the walls of the unit including the bedroom, bathroom and living room. They were bed bugs and the Tenants on their own initiative tried to control the situation by purchasing and repeatedly spraying the bugs. The Tenants evidence indicates that the situation did not subside and the infestation worsened and in addition to the bedding, the bugs had spread to their furniture including sofas. The infestation was such that the Tenants threw out the affected furniture and purchased new ones. Sometime during June or July, the Tenants complained to the Landlord about the situation, and claimed that the Landlord requested a $70.00 deposit from the Tenants to address the situation. The Tenants did not pay the deposit.

2. Subsequently in July or August the Landlord’s contractors sprayed the unit; but shortly thereafter, it was determined that the treatment had not addressed the problem. In September the Tenants told the Landlord that the treatment had not worked. Nothing further has been done according to the Tenants and they have continued to use spray to quell the increasingly worse situation. The Tenants claimed that since May of 2009 they have spent approximately $100.00 for the purchase of spray.

3. The Tenants also testified that to avoid the situation they have spent much time in motels. They found the situation intolerable and sought to move out of the unit immediately. The Tenants’ evidence included photographs depicting small dark areas which the Tenants claimed were bugs in a mattress and on walls of the unit.

4. The Landlord did not deny the Tenants’ testimony about the infestation, and stated that the unit was sprayed on August 26. They admitted that approximately 10 to 20 days later, the Tenants informed them that the bugs had not been eradicated. The Landlords claimed at that point, the Tenant was told to inform them when the unit would be prepared for them to again send in their contractors to treat the unit. The female Tenant stormed out of their

office, according to the Landlord, and they felt it was not necessary to conduct any further follow up. Instead, according to the Landlord, they sprayed other units in the building where the occupants with bed bugs had placed their names on a list, but made no attempt to address the infestation in the Tenants' unit. According to the Landlord's representatives, who claimed to have been Landlords for approximately 35 years, they had met their responsibilities by having the unit treated in August and their further request that the Tenants inform them when they would be prepared to allow another spraying.

2009 CanLII 79045 (ON L.T.B.)

5. In addition to termination of the tenancy on October 31, 2009, the Tenants seek rent abatement in the amount of $600.00 for having to live with bedbugs for 3 months; $5,000.00 for the purchase of new furniture due to the infestation by the bed bugs; moving expenses in the amount of approximately $300.00; and compensation in the amount of $400.00 for having to live with the bugs during the months of October and November 2008.

Analysis/Conclusion:

6. The issue to be determined is whether the Landlord conducted themselves in a manner that seriously interfered with the Tenants enjoyment of the unit, and whether this conduct forced the Tenants to consider moving out of the unit. I have considered the following in making a decision.

7. There was no dispute that the Tenants made the Landlord aware of the bed bug infestation. The evidence also indicates that although the Tenants tried to manage the problem themselves, it was severe enough that they had to dispose of items, including a sofa. According to the evidence presented, the Tenants disposed of the items before they formally brought the problem to the Landlords attention, sometime between July and August.

8. There was also no dispute that the Landlord had the unit sprayed for bed bugs in August; and that after the spraying the Landlord was advised that the problem still existed.

9. I find it was up to the Landlord, despite the Tenants' attitude, to ensure that the unit was treated, after they were advised that the problem persisted. The Landlord did not issue any notice requesting access to the unit for the purpose of dealing with the infestation, but instead the Landlord deliberately, avoided the Tenants' unit; while treating other units located around the Tenants' unit. I find having been a Landlord for 35 years, the Landlord ought to have known that treating selected units instead of all of the units in the building/on the floor, was not an effective means of preventing the spread of the infestation. In fact, in my view, the problem was compounded as the bugs would likely have moved from the other units to the Tenants' unit.

10. The fact that the Tenants might have demonstrated frustration in their communication with the Landlord, does not in my mind, warrant the Landlord's behaviour of ignoring the fact that there was a thriving bed bug infestation in the Tenants' unit; nor did it prevent the Landlord from taking steps to deal with the problem, in order to meet their maintenance responsibilities, and essentially protect their interest.

2009 CanLII 79045 (ON L.T.B.)

11. By ignoring the continuing problem, the Landlord essentially left it up to the Tenants to deal with the problem; which according to the evidence, they had been trying to contain for some time, independent of the Landlord. I find living under such conditions, undoubtedly substantially prevented the Tenants from being able to enjoy their apartment/complex.

12. By failing to effectively address the bed bug infestation, the Landlord substantially interfered with the Tenants' reasonable enjoyment of the unit; and further the evidence indicates that the Landlord was aware of this problem since at lease August of 2009. I find termination of the tenancy to be a reasonable remedy in this case, given that due to the lack of action on the Landlord's part, the Tenants furniture will likely continue to become infested if they remain in the unit; and their quality of life will continue to be negatively affected.

13. I also find the Tenants' claim for a rent rebate of $600. 00 for three months (from August to October), as well as approximately $300.00 moving expenses, to be reasonable under the circumstances; especially since there was no indication that the Tenants had any plans to move out of the unit, had there not been a bed bug infestation. I find it is also reasonable, notwithstanding the fact that the Tenants' provided no receipts, that the Landlord compensate them for 50% of the realistic amount ($100.00) they believe was spent on the purchase of products to control the infestation. This amounts to $50.00.

14. According to the Tenants testimony, they discarded beds, dressers and a chesterfield. According to the evidence, this was done before they formally complained of the problem to the Landlord in August; and furthermore, they provided no receipt for new/replacement purchases. I therefore find no other monetary awards are necessary in this case.

November 16, 2009
Date Issued

Claudette Leslie
Member, Landlord and Tenant Board

Toronto East-RO
2275 Midland Avenue, Unit 2
Toronto ON M1P3E7
