# Supplementary material for: Analysis of 44 Cases before the Landlord and Tenant Board Involving Bed Bug Infestations in Ontario, Canada: Focus on Adjudicator Decisions Based on Entomological/Pest Management Evidence and Accountability under the Residential Tenancy Act and Other Applicable Legislation
Source: Insects. 2011 Jul 19;2(3):343–53. doi: 10.3390/insects2030343 (PMC4553548; doi:10.3390/insects2030343)
Supplement: Supplementary File 1 [file insects-02-00343-s001.zip › supplementary-2/2009canlii79941.pdf]

Order under Sections 30, 31 & 87(1)
Residential Tenancies Act, 2006

2009 CanLII 79941 (ON L.T.B.)

File Numbers: TST-01845
TSL-16937

The Tenant's Applications:

RP (the 'Tenant') applied for an order determining that EM (the 'Landlord') failed to meet the Landlord's maintenance obligations under the *Residential Tenancies Act, 2006* (the 'Act') or failed to comply with health, safety, housing or maintenance standards.

In addition, the Tenant applied for an order determining that the Landlord harassed, obstructed, coerced, threatened or interfered with her, substantially interfered with the reasonable enjoyment of the rental unit or residential complex by the Tenant or by a member of her household and withheld or deliberately interfered with the reasonable supply of a vital service.

The Landlord's Application:

In addition, the Landlord (N Number PM Inc) applied for an order requiring the Tenant to pay the rent that she owes.

This application was heard in Toronto on October 15, 2008, December 10, 2008, March 10, 2009, July 28, 2009 and November 18, 2009. The Tenant, the Landlord's representative LM, the Landlord's paralegal SS attended all hearings.

The Tenant was represented by Counsel GW at the December 10, 2008 and March 10, 2009 hearings. Mr. GW ceased to represent the Tenant after the March 10, 2009 hearing.

On October 15, 2008 and December 10, 2008, Tenant's witness DD from Toronto Public Health gave evidence. He is referred to in this order as the "Public Health Inspector".

On December 10, 2008, Landlord witness SB, the Service Technician for [*Company name removed*] Pest Control gave evidence and Tenant witness Dr. SH research scientist from [*Hospital name removed*] Hospital gave evidence. SB is referred to in this order as the "Landlord's Pest Control Service Provider". Dr. H is referred to in this order as "Dr. H.".

Facts and Determinations:

The Tenant's Applications:

2009 CanLII 79941 (ON L.T.B.)

1. The Tenant moved into the rental unit on April 1, 2008 and vacated the rental unit on August 31, 2008.

2. In her application, the Tenant alleged that the Landlord breached its maintenance obligations, that the rental unit was infested with bed bugs and mice, and that the maintenance issues and the Landlord's lack of timely response substantially interfered with the Tenant's reasonable enjoyment of the rental unit. In addition, the Tenant alleged that the Landlord inactions and responses about the Tenant's complaints constituted harassment.

3. The Tenant the Landlord knew her rental unit was infested with bed bugs and mice when she moved into the rental unit in April 2008. The Tenant said that "…it only took a few weeks to get bug bites" after she moved in. The Tenant stated that she didn't discover they were bed bugs bites until she attended a walk-in medical clinic on August 2, 2008. The Tenant stated that the Landlord's female superintendent and male superintendent removed several dead mice from her rental unit.

4. The Tenant's allegations were not consistent with the independent 3rd party expert evidence that was introduced.

5. The Toronto Public Health Inspector gave evidence that he had been a public health inspector since 2000. He stated that "you can never know who brought bed bugs into the residential complex". On August 29, 2008, he inspected the rental unit after the unit had been treated 2 times. The only evidence of bed bugs that he found was 1 dead bed bug in a kitchen light fixture. In his evidence, he stated that "…based on his observations the bed bug population was low". The only evidence of mouse droppings that he found was 1 feces inside the stove which he stated described as old which had turned blue. The Toronto Public Health Inspector stated that the colour of the feces indicated that the mouse had consumed "bait". He stated that there was no evidence of a rodent problem in the rental unit.

6. The Landlord's Pest Control Service Provider gave evidence that his company has an ongoing service contract with the Landlord. This service contract was in place throughout the time material to the Tenant's application. He stated that on March 25, 2008, he saw no evidence of any infestation and that the Landlord is "pro-active" in its treatment of bed bugs and mice. In response to the Tenant's complaints the rental unit was treated 3 times (once on August 8, 2008, once on August 22, 2008 and once on Friday September 5, 2008).

7. The Landlord gave evidence that there is a posting in the common area near the elevator which states that pest control services company attends the complex each month. No evidence was introduced which contradicted this evidence.

8. **I believe that most reasonable people would find any problems with bed bugs or mice distressing.** I accept Dr. H.'s evidence that he has observed that exposure to bed bugs can result in emotional and mental distress in some people. However, the presence of bed bugs and mice in the rental unit, does not automatically make the Landlord in breach of its maintenance obligations and liable to the Tenant.

2009 CanLII 79941 (ON L.T.B.)

9. The Tenant's allegation that the Landlord knew that the rental unit was infested with bed bugs and mice prior to renting it to the Tenant is not supported by the evidence. If the rental unit was infested prior to the Tenant assuming occupancy, it would not have taken a few weeks for the Tenant to be bitten, and it is more likely than not that the Public Health Inspector would have found more evidence to support the Tenant's claim regarding bed bugs and mice. The Tenant's evidence does not support a finding that the Landlord is in breach of its maintenance obligations regarding the Tenant's claim about bed bugs and mice.

10. While there was a couple of small gaps between the walls and the floor which should have been finished with quarter round, however, I do not find that constitutes a breach of the Landlord's maintenance obligations.

11. I find that the Tenant failed to introduce sufficient evidence to establish that it is more likely than not that the Landlord breached its maintenance obligations regarding the common areas of the residential complex.

12. There was a city order regarding the garbage containers at the back of the residential complex and I find that the Landlord breached its maintenance obligations regarding the garbage containers. After considering the Tenant's evidence regarding how this impacted upon her, and the duration of the issue, I find that it is appropriate to grant the Tenant a lump sum abatement of rent in the amount of $150.00.

13. The grounds for the Tenant's rights application are based in the Tenant's maintenance application. I reviewed the correspondence and interaction between the Landlord and the Tenant in response to the Tenant's complaints about bed bugs and mice. I do not find that the Landlord's response to the Tenant's complaints constitute harassment or substantial interference with the Tenant's reasonable enjoyment of the rental unit.

The Landlord's Application:

14. The Tenant was in possession of the rental unit on the date the application was filed.

15. The Tenant was on a fixed term lease and did not provide the Landlord with a notice to terminate the tenancy in accordance with the requirements set out in the *Residential Tenancies Act, 2006 (*the "Act").

16. The Tenant did not pay the total rent she was required to pay for the period from August 1, 2008 to September 30, 2008.

17. The Landlord incurred bank charges of $5.00 as the Tenant stopped payment on her rent cheque for August 2008. The Landlord also incurred $20.00 administrative costs regarding the Tenant's stop payment cheque.

18. The Landlord gave notice that the rental unit was re-rented for December 1, 2008. The Landlord requested that the Tenant be required to pay the rent for August 2008 and September 2008. Under section 88 of the Act, I find that it is appropriate to grant this request.

It is ordered that:

Tenant's Applications:

1. The Landlord shall pay the Tenant a rent abatement in the amount of $150.00.

Landlord's Application:

2. The Tenant shall pay N Number PM Inc.'s $150.00 cost of filing this application with the Board. This cost is offset against the $150.00 rent abatement the Landlord was ordered to pay the Tenant in paragraph 1 above.

3. The Tenant shall pay to N Number PM Inc $1,900.00, which represents the amount of rent owing up to September 30, 2008.

4. The Tenant shall also pay to N Number PM Inc $25.00 in respect of the "stop payment" cheque.

5. The total amount of money the Tenant owes N Number PM Inc is $1,925.00. This amount represents rent arrears of $1,900.00, plus $25.00 in respect of the "stop payment" cheque, plus the Landlord's $150.00 cost of filing the application, less the $150.00 rent abatement awarded to the Tenant.

6. If the Tenant does not pay the N Number PM Inc. the full amount owing on or before January 4, 2010, the Tenant will start to owe interest. This will be simple interest calculated from January 5, 2010 at 2.00% annually on the balance outstanding.

December 21, 2009
Date Issued

Caroline King
Member, Landlord and Tenant Board

Toronto South Region
2nd Floor, 79 St. Clair Ave. E
Toronto ON M4T 1M6

If you have any questions about this order, call 416-645-8080 or toll free at 1-888-332-3234.

2009 CanLII 79941 (ON L.T.B.)
