# Supplementary material for: Analysis of 44 Cases before the Landlord and Tenant Board Involving Bed Bug Infestations in Ontario, Canada: Focus on Adjudicator Decisions Based on Entomological/Pest Management Evidence and Accountability under the Residential Tenancy Act and Other Applicable Legislation
Source: Insects. 2011 Jul 19;2(3):343–53. doi: 10.3390/insects2030343 (PMC4553548; doi:10.3390/insects2030343)
Supplement: Supplementary File 1 [file insects-02-00343-s001.zip › supplementary-2/2009canlii80648.pdf]

Landlord and Tenant Board

Order under Section 30
Residential Tenancies Act, 2006

File Number: SOT-01716-09

2009 CanLII 80648 (ON L.T.B.)

K. S and K.W (the 'Tenants') applied for an order determining that R.T (the 'Landlord') failed to meet the Landlord's maintenance obligations under the *Residential Tenancies Act, 2006* (the 'Act') or failed to comply with health, safety, housing or maintenance standards.

The Tenants also applied for an order determining that R.T (the 'Landlord') or the Landlord's superintendent or the Landlord's agent harassed, obstructed, coerced, threatened or interfered with them, entered the rental unit illegally and substantially interfered with the reasonable enjoyment of the rental unit or residential complex by the Tenants or by a member of their household.

This application was heard in Hamilton on December 15, 2009.

The Tenants and the Landlord’s representatives, R.T and J.A., attended the hearing.

Preliminary Matter:

The application is amended to change the Landlord’s name from R.T. to APM P.M. R.T is a mere employee of the Landlord.

Determinations:

1. I find that the Landlord failed to meet the Landlord's obligations under subsection 20(1) of the Act to maintain the rental unit and failed to comply with housing maintenance standards.

2. The Tenants bed and mattress and ancillary parts that come with the bed were damaged and disposed of as a result of the Landlord's failure to maintain the rental unit.

3. I am not persuaded that the Landlord harassed the Tenants. However, I find that the Landlord’s agent RT did react strongly to emails and accusations that the Tenants levelled against her or against the Landlord. I find that this is a normal response when one is under attack or accosted by the other side.

4. I find that the agents of the Landlord illegally entered the unit but that the entry can be used as they were done in an effort to attend to the Tenants’ complaints.

5. I find that the Tenants enjoyment of the unit was substantially interfered with when they learnt of the presence of bed bugs in the unit and the Landlord appeared to be putting the responsibility of presenting evidence of the presence of the bugs on the Tenants instead of investigating the infestation themselves. I also find that the Landlord’s means of response to the bed bug infestation unacceptable.

6. I find that the Landlord withheld a reasonable supply of a vital service that the Landlord was obligated to supply under the tenancy agreement for at least one month.

It is ordered that:

1. The Landlord shall pay to the Tenants a rent abatement of $225.00 for lack of heat and low hot water provision in the unit.

2. The tenancy between the Landlord and the Tenants was terminated as of December 1, 2009.

3. The Landlord shall also pay to the Tenants $1,346.94. This represents part of the costs that the Tenants will incur in replacing bed and mattress that was damaged and disposed of as a result of the Landlord's failure to repair and or maintain the rental unit.

4. The Landlord shall also pay to the Tenants $698.35 which is the reasonable out-of-pocket expenses the Tenants incurred with respect to dealing with the bed bug problems. The amount is broken down as follows:

   - $472.50 for extermination of the rental unit;
   - $43.67 costs for food;
   - $150.00 laundry expenses;
   - $32.18 for the allergy medication;

5. The Landlord shall also pay the Tenants $45.00 for the cost of filing the application.

6. The total amount the Landlord owes the Tenants is $2,315.29.

7. The Landlord shall pay the Tenants the full amount owing by January 2, 2010.

8. If the Landlord does not pay the Tenants the full amount owing by January 2, 2010 the Landlord will owe interest. This will be simple interest calculated from January 3, 2010 at 2.00% annually on the outstanding balance.

Reasons for the Order are attached.

2009 CanLII 80648 (ON L.T.B.)

December 22, 2009
Date Issued

Freda Shamatutu
Member, Landlord and Tenant Board

Southern-RO
119 King Street West, 6th Floor
Hamilton ON L8P4Y7

If you have any questions about this order, call 416-645-8080 or toll free at 1-888-332-3234.

File Number: SOT-01716-09

REASONS

2009 CanLII 80648 (ON L.T.B.)

Reasons to Order SOT-01716-09 issued on December 22, 2009 by Freda Shamatutu.

Electric wiring in the unit:

1. The Tenants moved into the rental unit on or around August 1, 2009. Soon after moving into the unit they noticed that a fuse in the basement would blow leading to the Tenants not having hydro in their living room, den and kitchen.

2. The Landlord investigated the problem and found that the blowing fuse was due to the many electric appliances that the Tenants used concurrently in the rental unit. The Landlord also found that the Tenants were using two air-conditioners in the unit which affected the electric voltage in the unit and caused the hydro to trip.

3. After considering the evidence of both parties, I find that the Landlord attended to the problem as soon as they were informed.

4. Section 16 of the Act requires a party to mitigate their losses. The Tenants should have known that the use of two air-conditioners would affect the electric wiring of the unit.

Leaky pipe/ tap in the bathroom:

5. In September 2009, the Tenants noticed that soapy water leaked from the bathroom pipe or faucet when they were washing their hands. The Landlord attempted to fix the problem soon after being informed, but the problem persisted. The problem was finally resolved after 4 attempts.

6. I am satisfied that the Landlord did not ignore the problem and that attempts were made to fix whatever was wrong. It has to be noted that although the expectation by the Tenants was that the problem would be resolved immediately, sometimes delays will happen in resolving problems. I find that any such delays are normal and should be expected.

7. The Tenants claim that personal possessions stored under the sink were damaged. The Tenants did not provide evidence of the damaged belongings.

Squirrels in the ceiling and walls:

8. The Tenants had a problem with squirrels which were living in the ceiling and the walls of the rental unit. The Landlord sent a handyman to open a hole in the ceiling or wall to let the squirrels out. The Tenants issue is that the Landlord did not close the hole after

that and that this caused cold drafts into the unit. The Tenants also complained that the Landlord did not give them 24 hours notice to enter the unit to do the repairs as required under the Act.

9. While I find that the Landlord has to give the Tenants notice to enter their unit, I also find that the Tenants had a number of maintenance complaints at the time which required the Landlord to enter the unit on a regular basis. Be that as it may, the Tenants are expected to cooperate with the Landlord in resolving the issues that they were having in the unit. A review of the various emails exchanged between the parties reveal that occasionally the Tenants did not provide the Landlord with the necessary cooperation to enable the Landlord do their work.

10. The hole that was made in the ceiling or wall was never closed after wards. The Tenants presented pictures of the open hole. The Landlord's evidence is that the hole appeared to have been tampered with possibly by the Tenants in order to make the problem appear larger than it really was.

11. The Tenants evidence is that the squirrels continued to be present in the house.

2009 CanLII 80648 (ON L.T.B.)

Lack of Heat and hot water:

12. The Tenants had no heat in the rental unit from September 1, 2009 per the requirement of the City of Hamilton By-Law No. 04-091. When told, the Landlord initially said that heat did not have to be on until October 15, 2009. The Tenants supplied the Landlord with the By-Law requiring heat to be on from September 1, to May 31st of the following year.

13. The Landlord then decided to turn the heat on. However they found that the boiler in the building had broken down. The Landlord supplied the Tenants with space heaters and also compensated the Tenants $25.00 per week (total $50.00 for two weeks). The Tenants claim that they had the space heater for two and half weeks but were only compensated for 2 weeks.

14. The Tenants subsequently reported the problem with the heat to the City of Hamilton. Upon the City's directive, the Landlord went into the unit and found that the valves to the radiators where shut. The valves were opened and heat came on after a service call on November 20, 2009 by a heating company employed by the Landlord.

15. At the hearing, the Landlord's argued that the Tenants must have shut the valves.

16. I find that it was the responsibility of the Landlord to go into the unit and investigate the problem immediately after the Tenants informed them that they had no heat in the unit. The Landlord cannot put the onus on the Tenants to check and ensure that the valves are open.

17. During the same period, the hot water supply was limited to 10 to 15 minutes of use per time. The Tenants claim that as at the time they filed the application with the Board on

November 24, 2009, they had intermittent heat and hot water and that the problem had not been resolved.

18. I am satisfied that the Tenants had heat as of November 20, 2009 after the valves were opened by a company contracted by the Landlord.

19. I find that some of the problems the parties had with each other were compounded by the fact that the female Tenant claimed that she was a plumber and could resolve the issues in the rental unit herself. The Landlord then entrusted her to do some of the work and when the problems were not resolved it lead to frustration on both parties. In addition it appeared that the Landlord had on a temporary basis engaged the services of the female Tenant as an assistant superintendent. This arrangement did not work out and lead to some of the acrimony that the parties had against each other.

2009 CanLII 80648 (ON L.T.B.)

Bed bugs:

20. On October 22, 2009, the Tenants discovered that their rental unit was infested with bedbugs. The female Tenant had noticed that she was getting bitten by something soon after moving into the unit. However she did not know what was biting her until she went to a walk in clinic and saw a doctor. The doctor told her that the bite marks appeared consistent to being bitten by bedbugs.

21. The Tenants informed the Landlord about the infestation immediately who assured the Tenants that the problem would be attended to immediately.

22. The Tenants investigated the bedbugs on the internet and found that the only way to get rid of the pests was by extermination. The Landlord was informed about the extermination.

23. Meanwhile instead of contracting a reputable pest control company to carry out the extermination, the Landlord gave the Tenants pesticides that they thought would rid the unit of the pests. The Tenants insisted that the only way to get rid of the pests was through extermination however the Landlord became rude and yelled at the Tenants blaming them of having brought the pests into the complex.

24. The Tenants reported the problem of the bedbugs to the Health and Safety Department of the City of Hamilton. Arrangements were made by the Health Department to come and inspect the unit. However the Landlord’s superintendent, R, who had agreed to be present did not show up thereby frustrating the efforts of the City who could not enter the unit as there was no key to the unit.

25. The Landlord subsequently consulted their lawyer and afterwards agreed to exterminate the unit. The Tenants were asked to contact a pest control company engaged by the Landlord who instead of attending the unit to inspect the unit themselves insisted that the Tenants should first drop of a sample of the bedbugs at their offices. The Tenants felt that the pest control company were not professional in their approach and asked the Landlord to get another company. The Landlord refused.

2009 CanLII 80648 (ON L.T.B.)

26. The pest control company engaged by the Landlord eventually went to the unit to fumigate the unit on October 26, 2009 after the Tenants had taken a bedbug sample to them. The Tenants claim the person sent to carry out the extermination was in the unit for about 15 minutes, refused to answer their questions and was rude. The person also left their doors and windows open after fumigating the unit.

27. Soon after the fumigation, the Landlord's superintendent, R, with who the Tenants had been dealing came to the unit and informed the Tenants that they (Landlord) had acquired the residential complex a few months earlier and that although the complex was renovated no fumigation for vermin was done to the unit. The complex had been empty for some time before the Landlord acquired it and had also been used by drug addicts and prostitutes. R apparently told the Tenants that she did not want to enter the Tenants' unit "because it was disgusting".

28. After the fumigation of the unit, the Tenants went to stay with relatives for a while. On return they found live bedbugs on their box spring and mattress. The Landlord informed them that it was normal to continue to have live bugs even after the fumigation. The Landlord said she could only arrange a second fumigation after about 10 days or so. No material, documents or counselling was provided to the Tenants regarding the bedbugs, how to get rid of them, preparation for fumigation and how to protect their belongings. No inspection of the unit was also done to check places that the bedbugs could have been hiding in. No after inspections were also done after the fumigation by the pest control company.

29. While preparing the unit for fumigation, the Tenants found mice droppings in the unit. The Landlord was informed but no action has been taken to inspect the unit and eradicate the mice (if still present in the unit).

30. When the problems with the mice persisted, the Tenants contracted a pest control company (O) who fumigated the unit and inspected their bed and mattress. The pest control company advised the Tenants to get rid of their bed and mattress. The Tenants presented pictures of damage done to their bed and mattress. The pictures show that the bed and mattress had the pests' fecal matter and blood tracks. The Tenants also presented pictures of live bedbugs on their belongings, and pictures of the bites the female Tenant had suffered.

31. The Tenant suffered allergic reactions to the bedbugs for which she had to be prescribed topical medication by a doctor.

Findings:

32. Over the last couple of years, there has been an increased concern about bedbug infestations in various communities. It has been found that infestation can cause anxiety secondary infections, allergic reactions and financial hardship. Public health departments in various cities have developed guidelines on how landlords and tenants can deal with the problem of bedbug infestation.

2009 CanLII 80648 (ON L.T.B.)

33. It has been generally found by various municipalities that multi-unit dwellings, including apartments are high-risk locations for bed bug infestations. It has also been found that the best method to deal with bed bugs is Integrated Pest Management (IPM), which combines a variety of techniques and products that pose the least risk to human health and the environment. It has also been found that collaboration between tenants and landlords is necessary to eliminate bed bug infestations. The normal recommended steps for landlords and property managers dealing with bed bug infestations include:

- An early response to tenant's complaint about bed bugs, and conducting proper inspections.
- Consulting the Public Health department or a professional pest control services about how to confirm bed bugs infestations;
- Recommendations for the control of bed bugs include the use of a professional pest control service experienced in bed bug control and Integrated Pest Management (IPM);
- Preparations for treatment of an infected unit include consulting with tenants in bed bug-affected units to ensure adequate preparation steps have been taken to prepare the units prior to treatment.
- After the treatment, an inspection of the unit should be carried out either by landlord or pest control professional is carried out following treatment to assess the treatment's effectiveness and determine if more spray is needed. Often more than one treatment is required;
- To prevent re-infestation the Landlord should seal cracks and crevices between baseboards, on wood bed frames, floors and walls with caulking. Repair or remove peeling wallpaper, tighten loose light switch covers, and seal any openings where pipes, wires or other utilities come into the home;
- The health department usually recommend the disposing of infected furniture.

34. At the hearing the Landlord did not provide evidence that they had engaged in any of the above recommendations. The Landlord did not inspect the unit to confirm the presence of the bugs instead they wanted to rely upon the Tenants providing them with evidence. The Landlord did not work with the Tenants in dealing with the problem and although they engaged a pest control company to exterminate the bugs, no evidence was given to the Board at the hearing of any assurances that the Landlord had made (if at all) to the Tenants that the engaged company had experience in bedbugs eradication or efforts made to work with the Tenants to prepare the unit for the eradication; after eradication education; and or how to protect the Tenants’ belongings from being re-infected.

35. Therefore, I find that the Landlord did not act prudently in attending to the problem of the bed bug infestation. This is further compounded by the Landlord’s evidence at the hearing that another unit in the complex was also infected with bedbugs.

Remedies:

36. I find that the Landlord became aware of the bedbug infestation in October 2009 and soon thereafter made attempts (though not effective) to get rid of the bugs. I am not convinced that the Landlord should be made responsible for abatement of rent for that month as

extermination and ridding the unit of the bedbugs started almost immediately the parties became aware of the bugs.

37. I find however that the Tenants had to dispose of their bed and mattress. I find that the Landlord should be responsible for at least half of the cost of replacement of the bed and mattress. Chances are that the bed bugs were in the unit when the Tenants took possession of the unit. Upon the Landlord’s admission no effort was made to fumigate the unit when the Landlord purchased the complex. According to the Tenants’ evidence which was not refuted by the Landlord at the hearing, the Landlord knew when they purchased the complex that the complex had been used by drug addicts and prostitutes.

2009 CanLII 80648 (ON L.T.B.)

38. I find that the Tenants are entitled to compensation for the professional pest control extermination of the unit; the refund for laundry expenses and refund of expenses for topical medication they had purchased.

39. The Tenants did not provide receipts for the extra food costs so I have only allowed a half of these costs.

40. I have also not allowed the claim for the day that the male Tenant took time off to speak to the Landlord about maintenance issues as there was no need for the Tenant to do that or in the alternative he could have done so without taking time off work.

41. I find that the Tenants are entitled to compensation for the lack of heat in the unit from September 1, 2009. I have allowed 10% rent abatement per month. The Tenants only had effective heat in the unit from November 20, 2009. Therefore, I have allowed 3 month’s rent abatement under this head.

42. I find that after the Landlord became aware of the need for them to provide heat in the unit, the Tenants were compensated for the extra cost of heating and were provided with space heaters during the period that repairs were being carried out in the unit. However, space heaters cannot be used as the main means of heating a rental unit ((O. Reg. 517/06, s.15 (4)).

43. I am satisfied that with all the other maintenance issues the Landlord did respond to the Tenants requests in a fairly reasonable manner. However the fact that some repairs took a little longer is understandable as the Landlord had to find the source of the problems before they could attend to the problem.

December 22, 2009
Date Issued

Freda Shamatutu
Member, Landlord and Tenant Board

Southern-RO
119 King Street West, 6th Floor
Hamilton ON L8P4Y7
