# Supplementary material for: Analysis of 44 Cases before the Landlord and Tenant Board Involving Bed Bug Infestations in Ontario, Canada: Focus on Adjudicator Decisions Based on Entomological/Pest Management Evidence and Accountability under the Residential Tenancy Act and Other Applicable Legislation
Source: Insects. 2011 Jul 19;2(3):343–53. doi: 10.3390/insects2030343 (PMC4553548; doi:10.3390/insects2030343)
Supplement: Supplementary File 1 [file insects-02-00343-s001.zip › supplementary-2/2010canlii13731.pdf]

Order under Section 31
Residential Tenancies Act, 2006

File Number: SOT-02512-10

2010 CanLII 13731 (ON L.T.B.)

N. K. (the 'Tenant') applied for an order determining that T. C. (the 'Landlord') harassed, obstructed, coerced, threatened or interfered with her. This application was heard in St. Catharines on February 18, 2010. The Tenant and the Landlord attended the hearing. The Landlord was represented by J. C..

At the hearing, the Landlord stated on the record that he suffers from post traumatic stress disorder. He advised that when under stress his answer to questions may be vague or confused. He asked the Board to accommodate him. I accommodated the Landlord by giving him additional time to answer questions. I allowed his Representative to lead him when he appeared to become agitated. He was advised that he could take a break at any time during the proceedings if he needed to.

The Tenant amended her application to include a request for abatement in her rent in the amount of $1750.00, $500.00 for moving expenses and an order for $200.00 a month for twelve months towards her rent.

On consent of the parties, the Tenant agreed to terminate her tenancy at the end of March 2010.

Determinations:

1. Following an inspection at the rental unit by the fire department, the Landlord was advised by the fire department chief that he faced several charges and substantial fines for failing to ensure that the smoke detectors were in good working order at the residential complex.

2. The Landlord attended at the rental unit after receiving this information. I am satisfied that the Landlord demanded the Tenant sign a note which he had prepared which implicated the Tenant or her former roommate in removing the smoke detector in the unit. I am satisfied the Landlord‘s behaviour during this visit was intimidating and threatening.

3. Despite the Tenant’s willingness to sign the Landlord’s note and accept culpability, the Landlord immediately served the Tenant with a notice of termination for safety impairment. I am not satisfied the fire department recommended the Landlord proceed to evict the Tenant. On the contrary, I am satisfied the Landlord substantially interfered with the Tenant’s reasonable enjoyment by demanding she sign the note and serving her with a notice of termination because of his apprehension to the charges and fines he faces with the fire department.

4. The Tenant waived the portion of her application related to bed bugs.

5. The Tenant’s request for an abatement of rent is reduced to $500.00.

6. The Tenant's request for moving costs and ongoing abatement towards her new rent are denied.

7. The Tenant's request for an administrative fine is denied.

2010 CanLII 13731 (ON L.T.B.)

It is ordered that:

1. The Landlord shall pay to the Tenant $500.00. This represents an abatement in rent for the month of January 2010.

2. The total amount the Landlord owes is $500.00.

3. The Landlord shall pay the Tenant the full amount owing by March 7, 2010.

4. If the Landlord does not pay the Tenant the full amount owing by March 7, 2010, he will owe interest. This will be simple interest calculated from March 8, 2010 at 2.00% annually on the balance outstanding.

5. The Tenant has the right, at any time, to collect the full amount owing or any balance outstanding under this order.

February 24, 2010
Date Issued

____
Jonelle Van Delft
Member, Landlord and Tenant Board

Southern-RO
119 King Street West, 6th Floor
Hamilton ON L8P4Y7

If you have any questions about this order, call 416-645-8080 or toll free at 1-888-332-3234.
