# Supplementary material for: Analysis of 44 Cases before the Landlord and Tenant Board Involving Bed Bug Infestations in Ontario, Canada: Focus on Adjudicator Decisions Based on Entomological/Pest Management Evidence and Accountability under the Residential Tenancy Act and Other Applicable Legislation
Source: Insects. 2011 Jul 19;2(3):343–53. doi: 10.3390/insects2030343 (PMC4553548; doi:10.3390/insects2030343)
Supplement: Supplementary File 1 [file insects-02-00343-s001.zip › supplementary-2/2010canlii13735.pdf]

Landlord and Tenant Board

Order under Sections 30 & 31
Residential Tenancies Act, 2006

File Number: SOT-02844-10

2010 CanLII 13735 (ON L.T.B.)

(S.P) (the 'Tenant') applied for an order determining that (W.P.) (the 'Landlord') substantially interfered with the reasonable enjoyment of the rental unit or residential complex by the Tenants or by a member of their household.

The Tenant also applied for an order determining that the Landlord failed to meet the Landlord's maintenance obligations under the *Residential Tenancies Act, 2006* (the 'Act') or failed to comply with health, safety, housing or maintenance standards.

These applications were heard in Hamilton on February 18, 2010.

The Tenant's representative, (L.B.) and the Landlord's representative, (E.S.), attended the hearing. (C.K.) and (B.H.) attended the hearing as witnesses for the Landlord.

Reasons are attached.

At the hearing, the Tenant's representative requested the consent of the Board to withdraw her T6 application.

Determinations:

1. L.B. is not a Tenant for the rental unit.

2. In accordance with subsection 200(4) of the Act, I consent to the withdrawal of the T6 application.

3. The notices of entry served on the Tenant by the Landlord did not specify times of entry, contrary to subsection 27(3) of the Act.

4. The Tenant did not object to the entry of the Landlord's agent into the unit, despite the deficiency in the notices of entry.

5. BH is an expert witness for the Landlord in this proceeding.

6. The Landlord treated the rental unit for the alleged bed bug infestation on October 7 and November 13, 2009, and January 8, 22 and 29, 2010.

7. The Landlord did not treat the rental unit for the alleged bed bugs on October 30 and December 18, 2009 due to a lack of unit preparation.

8. There is no evidence of a beg bug infestation in the unit.

9. The Tenant's claim for a rent abatement of $2,610.00 is denied.

10. The Tenant's claim for $4,500.00 of compensation for damaged furniture is denied.

11. The Tenant's claim for $300.00 of laundry expenses is denied.

It is ordered that:

1. The Tenant's application is dismissed.

2010 CanLII 13735 (ON L.T.B.)

February 24, 2010
Date Issued

Bittu George
Member, Landlord and Tenant Board

Southern-RO
119 King Street West, 6th Floor
Hamilton ON L8P4Y7

If you have any questions about this order, call 416-645-8080 or toll free at 1-888-332-3234.

File Number: SOT-02844-10

REASONS

2010 CanLII 13735 (ON L.T.B.)

Reasons to Order SOT-02844-10 issued on February 23, 2010 by Bittu George.

1. L.B. was listed as a Tenant on the T2 and T6 applications. E.S. objected to L.B. as a Tenant party to the proceeding. E.S. submitted a copy of Order SOL-21276 issued on July 29, 2009 listing S.P. as the only Tenant for the unit. L.B. resides in the unit. L.B. did not present any documentary evidence to support his claim that he is a Tenant in the rental unit. I find on a balance of probabilities that L.B. is not a Tenant of the rental unit, but an occupant of the unit.

2. L.B. requested that the Tenant's T6 application be withdrawn, as the grounds and remedies in the T6 application are the same as the T2 application. L.B. stated that the Tenant is not concerned with the $45.00 application fee for the T6 application. I consented to the Tenant's withdrawal of her T6 application.

3. C.K. testified that 24 hours written notice was served on the Tenant prior to treatment of the Tenant's unit for bed bugs. E.S. submitted to the Board copies of the notices of entry served on the Tenant. L.B. stated that no specific time was listed on the notices of entry, which prevented the Tenant from preparing the unit for bed bug treatment on 2 occasions. B.H. testified that the Tenant did not prevent him from entering the unit to treat the alleged bed bug infestation. I find on a balance of probabilities that the notices of entry served on the Tenant do not comply with subsection 27(3) of the Act requiring a specified time on a notice of entry. I also find that despite the lack of time specified on the notices of entry, the Tenant did consent to the entry of the Landlord's agent on all occasions, whether the rental unit was treated or not.

4. B.H. testified that he has been a licensed exterminator since 1983. B.H. testified to the education and qualifications required to be a government-licensed exterminator in Ontario. B.H. testified that he is licensed as a "structural exterminator" in Ontario. E.S. requested that B.H. be certified as an expert witness in this proceeding. I granted the request.

5. C.K. testified that there were complaints of a bed bug infestation in the rental unit prior to October 2009. B.H. testified that he treated the rental unit for the alleged bed bug infestation of on 4 occasions (October 7 and November 13, 2009, January 8 and 22, 2010) and attempted to do so on 2 occasions (October 30 and December 18, 2009). B.H. testified that he saw no evidence of a bed bug infestation in the rental unit on the occasions that he entered the unit. C.K. testified that M. P.C (another company) treated the rental unit for the alleged bed bug infestation on January 29, 2010. C.K. testified that the technician from M.P.C saw no evidence of a bed bug infestation. E.S. submitted a copy of the invoice from M.P.C indicating that treatment was only 70% completed due to the Tenant not removing a box spring and mattress, and not completely emptying the

dresser or chest drawer. C.K. testified that tenants are provided with documentation on how to prepare their unit for treatment. E.S. submitted a copy of the treatment preparation document to the Board. The invoice from M.P.C states that there was no evidence of a bed bug infestation in the unit. I find on a balance of probabilities that there was no bed bug infestation in the rental unit.

6. Since I have found in the paragraph above that there was no bed bug infestation in the rental unit, the Tenant's claim for rent abatement of $2,610.00, compensation of $4,500.00 for disposed furniture, and $300.00 for laundry expenses is denied.

7. Therefore, the Tenant's application is dismissed.

2010 CanLII 13735 (ON L.T.B.)

February 24, 2010
Date Issued

____
Bittu George
Member, Landlord and Tenant Board

Southern-RO
119 King Street West, 6th Floor
Hamilton ON L8P4Y7
