# Supplementary material for: Analysis of 44 Cases before the Landlord and Tenant Board Involving Bed Bug Infestations in Ontario, Canada: Focus on Adjudicator Decisions Based on Entomological/Pest Management Evidence and Accountability under the Residential Tenancy Act and Other Applicable Legislation
Source: Insects. 2011 Jul 19;2(3):343–53. doi: 10.3390/insects2030343 (PMC4553548; doi:10.3390/insects2030343)
Supplement: Supplementary File 1 [file insects-02-00343-s001.zip › supplementary-2/2010canlii13782.pdf]

Landlord and Tenant Board

Ontario

Order under Section 30
Residential Tenancies Act, 2006

File Number: TET-02403

2010 CanLII 13782 (ON L.T.B.)

(S.D.) (the 'Tenant') applied for an order determining that (C.P.A.) (the 'Landlord') failed to meet the Landlord's maintenance obligations under the *Residential Tenancies Act, 2006* (the 'Act') or failed to comply with health, safety, housing or maintenance standards.

This application was heard in Toronto on February 23, 2010.

The Tenant and her representative (S.E.) and the Landlord's representative (D.C.) attended the hearing.

Relevant facts:

1. The Tenant moved into the unit on or about May 1, 2009. This is a one bedroom apartment on the 20th floor of a high rise building.

2. Shortly after moving into the apartment, the Tenant noted some red marks on her arms. She had no idea what was causing the problem and dismissed it.

3. When the problem continued, the Tenant showed some of her co-workers at a medical clinic. Someone suggested that it may be bed bugs. Being unaware of what these were, the Tenant did some research and determined that this was a possibility.

4. At no time did the Tenant ever seek proper medical advice and with the exception of two out of focus pictures of someone's arm, had no evidence of any bites of any description.

5. When the Tenant had determined what the possible problem was, she discussed the problem with the live in building Manager who took action immediately by having the room treated for any bed bugs. The Tenant agreed with this statement.

6. This first occurred in mid August of 2009. For the next four to five months, the Tenant would come back to the office every two weeks and complain again and again the exterminators were sent as a precautionary measure.

7. The building Manager stated under oath if that any one in her building ever complained about any bug problem she automatically reported it to the extermination company for spraying. As she stated, she also lives in the building with her family and did not want these bugs in the building either.

8. In mid October while the problem was continuing, the building Manager conducted an inspection of the unit with no bugs being found. The company that was doing the spraying at that time also provided a letter stating that there was no bugs present even though the spraying continued at the request of the Tenant. The building manager also inspected the

units on both sides of the Tenant and again found no bugs. The people on both sides of the Tenant have not complained of having a bug problem.

9. In November, the parties agreed that the Tenant could leave the building as soon as she found other suitable accommodation. The Tenant vacated the unit on January 9, 2010.

10. On January 5, 2010, a thorough inspection of the unit was conducted personally by (D.M.). This gentleman is a recognized expert in the field of bedbugs. He has been in the business of extermination for over 30 years, has taken multiple chemical courses in the use of chemicals and extermination and sits on a Canada-USA committee looking into bed bugs and their extermination.

11. He stated that on his inspection, not only did he not find any live bugs, he could find no evidence of them ever having been there. He went on to say that the bugs leave a fecal print which is basically expelled blood. This stains any material that it touches and is very difficult to remove. He stated that he could find no fecal prints at all.

12. The Tenant's representative did not dispute that D.M. was an expert in his field.

2010 CanLII 13782 (ON L.T.B.)

Discussion/Reasons:

1. The Tenant was unable to provide any evidence that she had in fact ever been bitten by a bed bug. The pictures that she gave as evidence were out of focus and were merely pictures of someone's arm that had some type of bite. Despite working in a medical situation, the Tenant chose never to obtain a proper medical opinion and nothing was provided as evidence.

2. The building Manager had stated that she had attended the unit on at least three or four times with the exterminators and had never seen any evidence of bed bugs.

3. There is no doubt that the Landlord took every action possible in this situation despite not having any evidence that there was or was not a problem.

4. Both times in the written letters (October, 2009 and January, 2010) the inspectors state that there is no evidence of bed bugs in the rental unit.

5. In a case such as this, the burden of proof falls upon the party that has brought the accusations. In this case I do not believe that the Tenant has proved her case.

6. Based on the evidence that was presented I have no proof that the bed bugs existed therefore I have no choice other than to dismiss the case.

It is ordered that:

1. The Tenant's application is dismissed.

<u>February 24, 2010</u>
Date Issued

Jim McMaster
Member, Landlord and Tenant Board

Toronto East/Durham Region
Unit # 2, 2275 Midland Avenue
Toronto ON M1P 3E7

If you have any questions about this order, call 416-645-8080 or toll free at 1-888-332-3234.

2010 CanLII 13782 (ON L.T.B.)
