# Supplementary material for: Analysis of 44 Cases before the Landlord and Tenant Board Involving Bed Bug Infestations in Ontario, Canada: Focus on Adjudicator Decisions Based on Entomological/Pest Management Evidence and Accountability under the Residential Tenancy Act and Other Applicable Legislation
Source: Insects. 2011 Jul 19;2(3):343–53. doi: 10.3390/insects2030343 (PMC4553548; doi:10.3390/insects2030343)
Supplement: Supplementary File 1 [file insects-02-00343-s001.zip › supplementary-2/2010canlii13809.pdf]

Landlord and Tenant Board

Ontario

Order under Section 30
Residential Tenancies Act, 2006

File Number: TNT-02142

2010 CanLII 13809 (ON L.T.B.)

NG (the 'Tenant') applied for an order determining that FMGWL (the 'Landlord') failed to meet the Landlord's maintenance obligations under the *Residential Tenancies Act, 2006* (the 'Act') or failed to comply with health, safety, housing or maintenance standards.

This application was heard in Toronto on February 24, 2010.

The Tenant was represented by his son, VG.

The Landlord was represented by SK. The Landlord's witnesses include DM (pest control expert) and SP (property manager).

Determinations:

1. The Tenant began tenancy of the rental unit on May 1, 2009. A couple of weeks after he moved in, the Tenant complained to the Landlord about having bed bugs in the unit. The pest control technician hired by the Landlord treated the apartment for bed bugs on May 15, 2009. Responding to further complaints from the Tenant, the Landlord hired the pest control company to treat the apartment again on June 15, 2009 and August 21, 2009.

2. The Landlord's evidence indicates that the Tenant's rental unit is the *only* apartment unit in the whole building that was infested with bed bugs. The pest control service report also indicates that the Tenant did not follow the instruction given to him to "prepare" the unit for bed bug treatment. The Tenant did not refute the Landlord's claim.

3. There is no clear evidence to indicate the cause of the bed bug problem in the Tenant's unit. There is also no evidence to indicate that the bed bug infestation in the Tenant's rental unit was due to the Landlord's failure to meet maintenance obligations under the *Residential Tenancies Act, 2006* (the 'Act') or due to the Landlord's failure to comply with health, safety, housing or maintenance standards.

4. Before the Tenant brought on the complaint to the Landlord, the Landlord had no knowledge of the bed bug problem. The Landlord responded to the Tenant's complaint promptly by sending a pest control technician to "treat" the rental unit. The Tenant did not follow the instructions given by the Landlord to "prepare" his unit before the treatment for bed bugs. Responding to the Tenant's continued complaint, and the Landlord ordered bed bug treatment for the rental unit two more times. I find that the Landlord has acted responsibly and has exercised due diligence in addressing the maintenance complaint made by the Tenant.

5. In the application, the Tenant requested the Board to order the Landlord to pay him $15,000.00 to compensate for the furniture and clothing that they had thrown out as a

result of the bed bug infestation. The Tenant also requested a reimbursement of $3,500.00 of rent that he paid to a friend for the period May to November 2009 because he was not able to reside in the rental unit due to bed bugs.

6. The Tenant provided no evidence to support his claim that he had paid $3,500.00 to a friend because he could not continue to reside in the rental unit due to bed bug infestation.

7. The Tenant provided inconsistent testimonies concerning “what” he had disposed and therefore required compensation by the Landlord. His photographic evidence showing the furniture that he had in his unit are different from the photographs showing the furniture that was put in the apartment dumping area by the tenants in the building.

8. The Tenant did not provide adequate evidence to support his claim that he had disposed all of his furniture and clothing. He provided one sales receipt indicating that he had purchased 3 mattress sets and one bedroom set to the cost of $3,400.00. However, this receipt does not indicate the vendor’s name and address and there is no breakdown in the price of each of the items purchased. Overall, I did not find that the Tenant has provided adequate or credible evidence to support his claim for compensation.

2010 CanLII 13809 (ON L.T.B.)

It is ordered that:

The Tenant's application is dismissed.

February 26, 2010
Date Issued

____
Vincent Ching
Member, Landlord and Tenant Board

Toronto North Region
7th Floor, 47 Sheppard Ave E
Toronto ON M2N 5X5

If you have any questions about this order, call 416-645-8080 or toll free at 1-888-332-3234.
