# Supplementary material for: Analysis of 44 Cases before the Landlord and Tenant Board Involving Bed Bug Infestations in Ontario, Canada: Focus on Adjudicator Decisions Based on Entomological/Pest Management Evidence and Accountability under the Residential Tenancy Act and Other Applicable Legislation
Source: Insects. 2011 Jul 19;2(3):343–53. doi: 10.3390/insects2030343 (PMC4553548; doi:10.3390/insects2030343)
Supplement: Supplementary File 1 [file insects-02-00343-s001.zip › supplementary-2/2010canlii18631.pdf]

Landlord and Tenant Board

Order under Section 69
Residential Tenancies Act, 2006

File Number: SOL-03132-10

2010 CanLII 18631 (ON L.T.B.)

E. T. (the 'Landlord') applied for an order to terminate the tenancy and evict J. T. (the 'Tenant') because the Tenant did not pay the rent that the Tenant owes.

This application was heard in Hamilton on March 25, 2010.

The Landlord representative, T. F., and the Tenant attended the hearing.

At the hearing the Tenant raised the following issues pursuant to section 82 of the *Residential Tenancies Act, 2006* (the 'Act'): Bed bugs and maintenance

Determinations:

1. The Tenant has not paid the total rent he was required to pay for the period from January 1, 2010 to March 31, 2010. Because of the arrears, the Landlord served a Notice of Termination effective January 31, 2010.

2. The Tenant is still in possession of the rental unit.

3. The monthly rent is $796.97.

4. The Tenant has made not payments since the application was filed.

5. The Landlord collected a rent deposit of $796.97 from the Tenant and this deposit is still being held by the Landlord.

6. Interest on the rent deposit is owing to the Tenant for the period from June 1, 2008 to January 31, 2010.

7. The Tenant requested an abatement of rent and compensation, under s. 82, for losses relating to bed bugs, and other maintenance issues. For the reasons attached to this order, I found that the Tenant did not meet his evidentiary burden with respect to such issues.

8. I have considered all of the disclosed circumstances in accordance with subsection 83(2) of the *Residential Tenancies Act, 2006* (the 'Act'), and find that it would be unfair to grant relief from eviction pursuant to subsection 83(1) of the Act.

It is ordered that:

1. The tenancy between the Landlord and the Tenant is terminated. The Tenant must move out of the rental unit on or before April 12, 2010.

2010 CanLII 18631 (ON L.T.B.)

2. The Tenant shall pay to the Landlord $1,727.04*, which represents the amount of rent owing and compensation up to April 1, 2010, less the rent deposit and interest the Landlord owes on the rent.

3. The Tenant shall also pay to the Landlord $26.96 per day for compensation for the use of the unit starting April 2, 2010 to the date he moves out of the unit.

4. The Tenant shall also pay to the Landlord $170.00 for the cost of filing the application.

5. If the Tenant does not pay the Landlord the full amount owing* on or before April 12, 2010, the Tenant will start to owe interest. This will be simple interest calculated from April 13, 2010 at .00% annually on the balance outstanding.

6. If the unit is not vacated on or before April 12, 2010, then starting April 13, 2010, the Landlord may file this order with the Court Enforcement Office (Sheriff) so that the eviction may be enforced.

7. Upon receipt of this order, the Court Enforcement Office (Sheriff) is directed to give vacant possession of the unit to the Landlord, on or after April 13, 2010.

8. If, on or before April 12, 2010, the Tenant pays the amount of $2,742.00** to the Landlord or to the Board in trust, this order for eviction will be void. This means that the tenancy would not be terminated and the Tenant could remain in the unit. If this payment is not made in full and on time, the Landlord may file this order with the Court Enforcement Office (Sheriff) so that the eviction may be enforced.

9. The Tenant may make a motion to the Board under subsection 74(11) of the Act to set aside this order if they pay the amount required under that subsection on or after April 13, 2010 but before the Sheriff gives vacant possession to the Landlord. The Tenant is only entitled to make this motion once during the period of the tenancy agreement with the Landlord.

April 1, 2010
Date Issued

Michael Soo
Member, Landlord and Tenant Board

Southern-RO
119 King Street West
Hamilton ON L8P4Y7

If you have any questions about this order, call 416-645-8080 or toll free at 1-888-332-3234.

In accordance with section 81 of the Act, the part of this order relating to the eviction expires on October 13, 2010 if the order has not been filed on or before this date with the Court Enforcement Office (Sheriff) that has territorial jurisdiction where the rental unit is located.

* Refer to section A on the attached Summary of Calculations.
** Refer to section B on the attached Summary of Calculations.

Schedule 1
SUMMARY OF CALCULATIONS

File Number: SOL-03132-10

2010 CanLII 18631 (ON L.T.B.)

A. Amount the Tenant must pay if the tenancy is terminated:

Reasons for amount owing	Period	Amount
Arrears: (up to the termination date in the Notice of Termination)	January 1, 2010 to January 31, 2010	$932.00
Plus compensation: (from the day after the termination date in the Notice to the date of the order)	February 1, 2010 to April 1, 2010	$1,617.60
Less the rent deposit:		-$796.97
Less the interest owing on the rent deposit:	June 1, 2008 to January 31, 2010	-$25.59
Less amount owing to the Tenant for abatement/rebate:		$0.00
Amount owing to the Landlord on the order date:(total of previous boxes)		**$1,727.04**
Additional costs the Tenant must pay to the Landlord:		$170.00
Plus daily compensation owing for each day of occupation starting April 2, 2010:		$26.96 (per day)
**Total the Tenant must pay the Landlord if the tenancy is terminated:**		**$1,897.04, + $26.96 per day starting April 2, 2010**

B. Amount the Tenant must pay to void the eviction order and continue the tenancy:

Reasons for amount owing	Period	Amount
Arrears:	January 1, 2010 to April 30, 2010	$2,572.00
Less amount owing to the Tenant for abatement/rebate:		$0.00
Additional costs the Tenant must pay to the Landlord:		$170.00
**Total the Tenant must pay to continue the tenancy:**	On or before April 12, 2010	$2,742.00

Landlord and Tenant Board
Ontario

File Number: SOL-03132-10

REASONS

Reasons to Order SOL-03132-10 issued on April 1, 2010 by Michael Soo.

2010 CanLII 18631 (ON L.T.B.)

Section 82 Issues

1. In response to the Landlord's application for arrears, and for termination and eviction, the Tenant raised several issues, pursuant to s. 82 of the Act. The main issue was an alleged bed bug infestation.

Bed Bugs

2. The Tenant has lived in the rental unit for approximately five years. He testified that he has had a bed bug issue for approximately two years. When asked to give a more specific timeline, he suggested that the issue might have started in summer of 2007. He testified that he told the Landlord after approximately two months, and that an exterminator attended to address the issue.

3. The bed bugs persisted, causing the Tenant to throw out a futon, a mattress and an entire bedroom set approximately one year ago. He could not remember the exact date on which these items were disposed of.

4. The Tenant further testified that the exterminator returned on several occasions, to other rental units in the building. He took this to mean that other tenants were also experiencing the same issue.

5. The Tenant called a witness, Mr. E, to testify that he personally saw bed bugs in the rental unit.

6. The Landlord's evidence was that no maintenance requests were ever sent about this issue, and that the exterminator had been sent on the Landlord's own initiative. It was also submitted that exterminators were periodically called to address regular pest control issues, not for specific bed bug problems.

Other Issues

7. The Tenant's next issue was that the rental unit has old windows that have not been replaced. He testified that the Landlord has promised to do so for the last two years, but has not. The Tenant acknowledged that he has submitted no maintenance requests with respect to this issue.

8. The Tenant also complained that the building elevators are unreliable. On a recent occasion, two out of the three elevators were out of service.

9. The Landlord's response with respect to the elevator issue was that the elevators are currently undergoing regular maintenance.

2010 CanLII 18631 (ON L.T.B.)

10. The Tenant complained of the building's water supply being cut off twelve times in the last year, though advance notice was given.

11. The Landlord responded that the water supply issue was also simply a matter of routine maintenance.

Analysis

12. I must find that the Tenant has fallen far short of his burden of proof with respect to the maintenance issues raised, particularly as the Landlord was never provided with maintenance requests.

13. The bed bug issue was especially undermined by vague evidence on the Tenant's part. I was provided with very unspecific dates about the infestation and the disposal of furniture. There were no maintenance requests submitted. The Tenant provided no evidence about the replacement cost for the furniture. The evidence was manifestly scant and unreliable. I could also place no weight on the Tenant's assumption that bed bugs were a pervasive problem in the building, based solely on having seen exterminators attend on other occasions.

14. The evidence with respect to the old windows was also lacking. No maintenance requests were submitted. No evidence was provided with respect to any loss or inconvenience. I was unable to draw any conclusions on this issue.

15. I further found no significant issue with respect to the elevator and water issues. The Landlord cannot be faulted for performing regular maintenance work, which might cause mild inconvenience to tenants for short periods of time.

16. There is no merit to any of the issues raised by the Tenant under s. 82.

April 1, 2010
Date Issued

____
Michael Soo
Member, Landlord and Tenant Board

Southern-RO
119 King Street West
Hamilton ON L8P4Y7

In accordance with section 81 of the Act, the part of this order relating to the eviction expires on October 13, 2010 if the order has not been filed on or before this date with the Court Enforcement Office (Sheriff) that has territorial jurisdiction where the rental unit is located.
