# Supplementary material for: Analysis of 44 Cases before the Landlord and Tenant Board Involving Bed Bug Infestations in Ontario, Canada: Focus on Adjudicator Decisions Based on Entomological/Pest Management Evidence and Accountability under the Residential Tenancy Act and Other Applicable Legislation
Source: Insects. 2011 Jul 19;2(3):343–53. doi: 10.3390/insects2030343 (PMC4553548; doi:10.3390/insects2030343)
Supplement: Supplementary File 1 [file insects-02-00343-s001.zip › supplementary-2/2010canlii37684.pdf]

Landlord and Tenant Board

Order under Section 30 and 31
Residential Tenancies Act, 2006

File Number: SOT-04359-10

2010 CanLII 37684 (ON L.T.B.)

A. D. (the 'Tenant') applied for an order determining that:

G. P. M. C. (the 'Landlord') or the Landlord's superintendent or the Landlord's agent substantially interfered with the reasonable enjoyment of the rental unit or residential complex by the Tenant, and

The Landlord failed to meet their maintenance obligations under the *Residential Tenancies Act, 2006* (the 'Act') or failed to comply with health, safety, housing or maintenance standards.

These applications were heard in Hamilton on May 4, 2010.

The Tenant and the Landlord’s representative attended the hearing. Three witnesses testified at the hearing.

The maintenance issue raised in the applications is a bedbug infestation in the rental unit.

Preliminary Matter:

1. The applications were amended, upon the request of the Tenant, to replace the claim for expenses for the treatment of bedbugs in the amount of $1500.00 and for the loss of clothing in the amount of $500.00 with the amount of $700.00 for the replacement of a bed.

Determinations:

1. The Landlord breached his obligation under s.20 of the Act because there are bedbugs in the unit. However, I find that the Landlord reacted expeditiously and reasonably when the Tenant brought the bedbug infestation to the Landlord’s attention.

2. The Landlord’s bedbug treatment efforts were reasonable and sufficient. Accordingly the Tenants’ request for remedy is denied.

3. I find there is not sufficient evidence to establish it was necessary to replace the bed due to the bedbug infestation.

It is ordered that:

1. The Tenant filed a T2 application on the same grounds as the T6 application for maintenance therefore the T2 application is dismissed.

2. The T6 application is dismissed for the reasons attached.

May 7, 2010
Date Issued

Shirley Collins
Member, Landlord and Tenant Board

Southern-RO
119 King Street West, 6th Floor
Hamilton ON L8P4Y7

If you have any questions about this order, call 416-645-8080 or toll free at 1-888-332-3234.

2010 CanLII 37684 (ON L.T.B.)

File Number: SOT-04359-10

REASONS

2010 CanLII 37684 (ON L.T.B.)

Reasons to Order SOT-04359-10 issued on May 7, 2010 by Shirley Collins.

The Tenant has resided in the rental unit for 40 years.

At the hearing the Landlord's representative said the Landlord is considering charging the Tenant for the cost of the treating the bedbug infestation in the unit. The Landlord has paid $1500.00, to date.

The Tenant said the only amount he is requesting is $700.00 for the new bed he purchased to replace the bedbug infested bed.

The Tenant testified he told the superintendent of the rental complex sometime in March 2010 that he had bugs in his unit. The superintendent acted immediately and treatment followed.

The superintendent and property manager testified that the pest control company which is on contract with the Landlord was contacted the same day the bugs were reported. The bugs were identified as bedbugs.

The representative of the pest control company testified that on March 22, 2010 he met with the Tenant and discussed the action required to prepare the unit for treatment. The first treatment of the unit took place on the following day. There have been three treatments to date.

The Tenant testified he threw out his bed because it was infested with bedbugs. The representative of the pest control company testified that he did not tell the Tenant to throw it out and that it could have been treated. I find there is not sufficient evidence that it was necessary to replace the bed due to the bedbug infestation.

The representative of the pest control company testified that there has been no other complaint of bedbug infestations in this residential complex. The property manager testified that there has been no complaint of a bedbug infestation in the 3½ years she has been in that position.

The Landlord's representative submitted the Tenant is responsible for the bedbug infestation. She provided two previous decisions of the Board, EAT-00878-09 and SWT-01422 for consideration.

In EAT-00878-09 the Member found the Tenant responsible for bringing the bedbugs into the rental unit. He found the Landlord not responsible for the effects of the bedbug problem. There is no clear finding of the Landlord's responsibility under s. 20 of the Act and no reasons attached to the order.

In SWT-01422 the Member made a finding that the Tenant did not establish that the bedbug infestation was caused by an act or omission of the Landlord. Another finding was that the

Tenant did not establish the Landlord failed to meet the Landlord's responsibilities under subsection 20(1) of the Act.

I have considered the previous decisions submitted. I am not bound by the decision of other Members.

Though the Landlord did not bring the bedbugs into the unit, the Landlord has a responsibility under s. 20 (1) of the Act which states, in part:

A landlord is responsible for providing and maintaining a residential complex, including the rental unit in it, in a good state of repair and fit for habitation and for complying with health, safety, housing and maintenance standards.

I find that the Landlord breached their obligation under s.20 of the Act because there are bedbugs in the unit. However, I find that the Landlord reacted expeditiously and reasonably when the Tenant brought the bedbug infestation to the Landlord's attention.

2010 CanLII 37684 (ON L.T.B.)

May 7, 2010
Date Issued

Shirley Collins
Member, Landlord and Tenant Board

Southern-RO
119 King Street West, 6th Floor
Hamilton ON L8P4Y7
