# Supplementary material for: Analysis of 44 Cases before the Landlord and Tenant Board Involving Bed Bug Infestations in Ontario, Canada: Focus on Adjudicator Decisions Based on Entomological/Pest Management Evidence and Accountability under the Residential Tenancy Act and Other Applicable Legislation
Source: Insects. 2011 Jul 19;2(3):343–53. doi: 10.3390/insects2030343 (PMC4553548; doi:10.3390/insects2030343)
Supplement: Supplementary File 1 [file insects-02-00343-s001.zip › supplementary-2/2010canlii37735.pdf]

Landlord and Tenant Board
Ontario

Order under Section 30
Residential Tenancies Act, 2006

File Number: TET-02033-09

2010 CanLII 37735 (ON L.T.B.)

In the matter of: [*Address removed*]

Between: [*Tenant's name removed*] Tenant

and

[*Landlord's name removed*] Landlord

[*Tenant's name removed*] (the 'Tenant') applied for an order determining that [*Landlord's name removed*] (the 'Landlord') failed to meet the Landlord's maintenance obligations under the *Residential Tenancies Act, 2006* (the 'Act') or failed to comply with health, safety, housing or maintenance standards.

This application was heard in Toronto on January 27, 2010.

The Tenant and the Landlord's [*Housing Supervisor's name removed*] attended the hearing. The Landlord was represented by [*Landlord Representative's name removed*]. [*Projector Supervisor's name removed*], a Project Supervisor with [*Landlord's name removed*], testified on behalf of the Landlord.

Determinations:

1. The Tenant alleges that the Landlord has failed to comply with health, safety, housing or maintenance standards because his unit is infested with bed bugs.

2. The Tenant testified that he moved into the unit in February 2008 and first saw the bed bugs around August 2008. The Tenant cannot recall the date he first complained to the Landlord about the bedbugs, but the parties agree that the unit was treated by a professional exterminator on February 12, 2009. The Tenant alleges that the exterminator soaked his futon with chemicals, forcing him to spend $200.00 for a new mattress. The Tenant states that treatment worked at first, but that the bed bugs reappeared several months later. Citing an August 2006 report issued by the United States Armed Forces Pest Management Board, the Tenant believes that the treatment was unsuccessful because the entire (multi-unit) building is infested, and the Landlord does not have a comprehensive plan to address the problem.

3. The Tenant requested a second treatment by an exterminator in November 2009. However, citing a Wikipedia entry, the Tenant now believes that the use of chemicals to treat the bed bugs is dangerous to his health. Therefore, he cancelled the second scheduled treatment. The Tennant paid a contractor $200.00 to seal up his unit in March 2009. The Tenant testified that this has helped reduce the bedbug problem (along with

steam cleaning and keeping many items covered by plastic), but that bed bugs are still present.

4. The Landlord questioned the validity of the materials submitted by the Tenant in support of his argument that the use of chemicals to treat bed bugs is harmful, and submitted that the [*Landlord's name removed*] bedbug treatment program is consistent with the City of Toronto guidelines for the treatment of bed bugs in multi-unit dwellings for Pest Management Professionals.

5. [*Housing Supervisor's name removed*] testified that [*Lead Specialist name removed*], the City of Toronto Public Health Lead Specialist, Toronto Bed Bug Project, inspected the rental unit on January 26, 2009 with the Tenant present and found no signs of live bed bugs or bed bug fecal matter. As per my direction, the Landlord also submitted to the Board an email from [*Lead Specialist name removed*] dated January 26, 2009 confirming her findings.

6. [*Project Supervisor's name removed*] testified that it is the Landlord's practice to take measures such as sealing up all gaps and holes in a rental unit if several rounds of treatment by an exterminator do not adequately address a bed bug problem.

7. I find that the Tenant has failed to establish that the Landlord has breached its obligations to comply with health, safety, housing or maintenance standards. The best evidence with respect to the current condition of the unit, the inspection by [*Lead Specialist name removed*] from the Toronto Public Health, suggests that the unit is not currently infested with bed bugs. Further, in the event there are some bed bugs remaining in the rental unit, the Tenant is likely at least partially responsible because he has refused the Landlord's offer to have his unit treated a second time by a professional exterminator. While I accept that the Tenant has a genuine concern about the possible harmful health effects of the chemicals used by the exterminator, I am satisfied that the Landlord's proposed means of addressing bed bugs falls within the generally accepted practice in the City of Toronto for dealing with this type of problem. The Tenant cannot claim that the Landlord has failed to meet its maintenance obligations if he rejects the Landlord's attempts to address the problem in a manner that is generally accepted.

2010 CanLII 37735 (ON L.T.B.)

It is ordered that:

1. The Tenant's application is dismissed.

February 2, 2010
Date Issued

____
Eli Fellman
Vice Chair, Landlord and Tenant Board

Toronto East-RO
2275 Midland Avenue, Unit 2
Toronto ON M1P3E7

If you have any questions about this order, call 416-645-8080 or toll free at 1-888-332-3234.
