# Supplementary material for: Analysis of 44 Cases before the Landlord and Tenant Board Involving Bed Bug Infestations in Ontario, Canada: Focus on Adjudicator Decisions Based on Entomological/Pest Management Evidence and Accountability under the Residential Tenancy Act and Other Applicable Legislation
Source: Insects. 2011 Jul 19;2(3):343–53. doi: 10.3390/insects2030343 (PMC4553548; doi:10.3390/insects2030343)
Supplement: Supplementary File 1 [file insects-02-00343-s001.zip › supplementary-2/2010canlii37783.pdf]

Landlord and Tenant Board

Ontario

Order under Sections 30 and 31
Residential Tenancies Act, 2006

File Number: TST-03117

2010 CanLII 37783 (ON L.T.B.)

In the matter of: [*Address removed*]

Between: [*Tenant's name removed*] Tenant

and

[*Landlord(1)'s name removed*] Landlords
[*Landlord(2)'s name removed*]

[*Tenant's name removed*] (the 'Tenant') applied for an order determining that [*Landlord(1)'s name removed*] and [*Landlord(2)'s name removed*] (the 'Landlords') failed to meet the Landlords' maintenance obligations under the *Residential Tenancies Act, 2006* (the 'Act') or failed to comply with health, safety, housing or maintenance standards.

The Tenant also applied for an order determining that the Landlords substantially interfered with the reasonable enjoyment of the rental unit or residential complex by the Tenant or by a member of her household.

This application was heard in Toronto on February 10, 2010. The Tenant and the Landlords attended the hearing.

Determinations:

1. The Tenant's maintenance application (T6) alleges that the Landlords failed to comply with subsection 20(1) of the *Residential Tenancies Act, 2006* (the 'Act'), because they failed to adequately address an infestation of bedbugs in the rental unit.

2. The tenant rights application (T2) alleges that the Landlords' failure to resolve the bedbug issue substantially interfered with the Tenant's reasonable enjoyment of the rental unit contrary to section 22 of the Act.

3. Pursuant to 20(1) of the Residential Tenancies Act, 2006 (the 'Act'):

   > *A landlord is responsible for providing and maintaining a residential complex, including the rental units in it,* ***in a good state of repair*** *and fit for habitation and for complying with health, safety, housing and maintenance standards****.*** [Emphasis added]

4. Subsection 20(1) of the Act requires a landlord to maintain a rental unit in a good state of repair. Clearly, between December 7, 2009 and January 12, 2010 (the date that the inspection confirmed the unit was bedbug free) the rental unit could not be said to have

been in a good state of repair due to the presence of bedbugs and as a result, I am satisfied that the Landlord failed to meet the Landlord's obligations under subsection 20(1) of the Act.

2010 CanLII 37783 (ON L.T.B.)

5. Based on the evidence before me, I am satisfied that the Tenant has established that the presence of bedbugs in her rental unit had substantially interfered with her reasonable enjoyment of the rental unit.

6. I am of the view that a reasonable abatement of the rent would be $704.22 which represents a 70% rent abatement for the period commencing December 7th (the date the Tenant notified the Landlords) and January 12, 2010 (the date that the inspection confirmed the unit was bedbug free).

7. I am satisfied that the reasonable costs that the Tenant will incur to replace the mattress as a result of the Landlords' breach to be $639.99.

8. I am satisfied that the reasonable costs that the Tenant will incur to replace the sofa as a result of the Landlords' breach to be $500.00.

9. The Tenant's request for compensation for out of pocket expenses for laundering her clothing and bedding in the amount of $135.00, plastic bags for laundered items $26.37 and 2 boxes in the amount of $19.98 is granted pursuant to subparagraph 30(1)5,ii of the Act. These items are directly related to the Landlords' contractual breach and are reasonable.

10. The Tenant's request for compensation for out of pocket expenses incurred for medical expenses in the amount of $90.00 is granted pursuant to subparagraph 30(1)5,ii of the Act. These items are directly related to the Landlords' contractual breach and are reasonable.

11. The Tenant's request for compensation for moving expenses, public storage and plastic bins in the amount of $651.12 is denied.

12. The Tenant's request that the tenancy be terminated on January 18, 2010 is denied

It is ordered that:

1. The Landlords shall pay to the Tenant a rent abatement of $704.22.

2. The Landlords shall also pay to the Tenant $1,139.99. This represents the costs that the Tenant will incur to replace property that was disposed of as a result of the Landlords' breach.

3. The Landlords shall also pay to the Tenant $271.35 which is the reasonable out-of-pocket expenses the Tenant has incurred for medial expenses, laundering and protecting clothing and bedding due to the presence of bedbugs in the rental unit. These expenses were incurred because of the Landlords' breach.

4. The Landlords shall also pay the Tenant $45.00 for the cost of filing the application.

5. The total amount the Landlords owe the Tenant is $2,160.56.

6. The Landlords shall pay the Tenant the full amount owing by March 31, 2010.

7. If the Landlords do not pay the Tenant the full amount owing by March 31, 2010 the Landlords will owe interest. This will be simple interest calculated from April 1, 2010 at 2.00% annually on the outstanding balance.

2010 CanLII 37783 (ON L.T.B.)

March 3, 2010
Date Issued

Guy Savoie
Vice Chair, Landlord and Tenant Board

Toronto South Region
2nd Floor, 79 St. Clair Ave. E
Toronto ON M4T 1M6

If you have any questions about this order, call 416-645-8080 or toll free at 1-888-332-3234.

Landlord and Tenant Board

Ontario

File Number: TST-03117

REASONS

2010 CanLII 37783 (ON L.T.B.)

In the matter of: [*Address removed*]

Between: [*Tenant's name removed*] Tenant

and

[*Landlord(1)'s name removed*] Landlords
[*Landlord(2)'s name removed*]

Reasons to Order TST-03117 issued on February 16, 2010 by Guy Savoie.

1. The Tenant's maintenance application (T6) alleges that the Landlords failed to comply with subsection 20(1) of the *Residential Tenancies Act, 2006* (the 'Act'), because they failed to adequately address an infestation of bedbugs in the rental unit.

2. The tenant rights application (T2) alleges that the Landlords' failure to resolve the bedbug issue substantially interfered with the Tenant's reasonable enjoyment of the rental unit contrary to section 22 of the Act.

3. The complex consists of a commercial unit on the first floor and two self contained one bedroom residential units on the second floor. The parties entered into a one year tenancy agreement starting on December 1, 2009. The monthly rent for the unit was $850.00.

4. It was the evidence before me that the Tenant moved into the rental unit on December 5, 2009. The following morning she awoke to discover that she had sustained multiple bug bites on her arms. The Tenant put the Landlords on notice of the problem on December 7th. The Landlords immediately contacted a pest control company that attended the unit that same day. It was determined that both bedbugs and fleas were present in the rental unit and the unit was treated. The Tenant followed the instructions provided by the exterminator to address the bedbug problem by laundering her bedding and clothing, bagging items, vacuuming, et cetera. The Tenant experienced an allergic reaction to the bedbug bites and was under a doctor's care.

5. Based on the evidence, I am satisfied that the Landlords were not aware of any bedbug or flea infestation in the rental unit until the Tenant notified them of the problem on December 7, 2009.

6. The Landlords authorized the Tenant to contact the pest control company directly to have the rental unit treated as many times as required to rid the unit of bedbugs and fleas.

2010 CanLII 37783 (ON L.T.B.)

7. On the evening of December 7th the Tenant decided to sleep on the sofa in the living room instead of her bedroom. She awoke the next morning to discover that she had received more bedbug bites. The Tenant placed a call to the exterminator the following day and the unit was treated again on December 10th.

8. It was the evidence before me that when the exterminator attended on December 10th he instructed the Tenant to discard the mattress. The Tenant removed the mattress from the unit and it was taken to the dump approximately a week later. The Tenant's evidence with respect to exterminator's recommendation to dispose the mattress was not shaken under cross-examination. In addition, the Landlords had no personal knowledge of the conversation between the exterminator and the Tenant. In the absence of any rebuttal witness called by the Landlords, I find that the Tenant discarded the mattress on the recommendation of the exterminator.

9. The rental unit was treated once again on December 11th at the Tenant's request.

10. On December 12, 2009, due to the ongoing problem with bedbugs in the unit the Tenant went to stay at her sister's home until December 26th. However, the Tenant did return to the unit periodically to pick up bagged clothing and to provide access to the exterminator to treat the unit. On December 14th, when the exterminator and the Tenant pulled back the carpet they discovered an infestation of bedbugs and fleas. The exterminator recommended that the Tenant throw out her sofa because it was "not salvageable". The Tenant's evidence in this regard was not shaken under cross-examination. The Landlords again had no personal knowledge of the conversation between the exterminator and the Tenant. In the absence of any rebuttal witness called by the Landlords, I find that the Tenant discarded the sofa on the recommendation of the exterminator.

11. On December 16, 2009, the Landlords replaced all the carpets and also sealed all cracks between the walls and baseboards within the rental unit. The unit was treated once again on December 21, 2009.

12. On December 26th the Tenant slept in the rental unit for the first time since December 12th. When she awoke the next morning she was unable to open her left eye due to swelling caused by an allergic reaction to bedbug bites on her face. The Tenant submitted photographs of her arms and face into evidence chronicling the impact of the bedbug bites on her body during the time she resided in the rental unit. The Tenant moved back to her sister's home. On December 31, 2009, the Tenant e-mailed the Landlords to inform them that she was unable to reside in the unit because of the on-going problem of bedbugs and expressed a desire to terminate her tenancy. The Landlords did not consent to the Tenant's request to terminate the tenancy.

13. The Tenant's unit was treated a total of seven times from December 7th to December 31, 2009.

14. On January 7, 2010, the Tenant provided the Landlords vacant possession of the rental unit. The Tenant, after calling other pest control companies and fearing that she would bring the bedbugs to her new unit, took it upon herself to discard numerous items

including bookshelves, bedding, a dining room set, nightstands, coffee table, curtains, et cetera.

15. On January 12, 2010, the Landlords hired another pest control company to inspect the rental unit to ensure that it was completely free of any bedbugs or fleas. The inspection report submitted into evidence confirmed the unit was pest free.

2010 CanLII 37783 (ON L.T.B.)

16. Pursuant to 20(1) of the Act:

> *A landlord is responsible for providing and maintaining a residential complex, including the rental units in it,* ***in a good state of repair*** *and fit for habitation and for complying with health, safety, housing and maintenance standards.* [emphasis added]

17. Subsection 20(1) of the Act requires a landlord to maintain a rental unit in a good state of repair. Clearly, between December 7, 2009 and January 12, 2010 (the date that the inspection confirmed the unit was bedbug free) the rental unit could not be said to have been in a good state of repair due to the presence of bedbugs and as a result, I am satisfied that the Landlord failed to meet the Landlord's obligations under subsection 20(1) of the Act.

18. Furthermore, based on the evidence before me, I am satisfied that the Tenant has established that the presence of bedbugs in her rental unit substantially interfered with her reasonable enjoyment of the rental unit.

Remedies:

19. Abatement of rent is a contractual remedy based on the principle that if you are paying 100% of the rent then you should be getting 100% of what you are paying for and if you are not getting that, then a tenant should be entitled to abatement equal to the difference in value. The monthly rent for the unit was $850.00. Although I am not bound by it, I find the Board's Guideline #5 entitled Breach of Maintenance Obligations helpful. It says in part "This remedy should not be seen as punishment for landlord conduct or inaction. It is compensation to the tenant for the inadequate state of repair and any inconvenience or actual loss of use of the rental unit or common facilities". In determining the quantum of the rent abatement I have considered the serious impact the problem had on the Tenant and the steps taken by the Landlords to address the issue when they were made aware of the problem. Given all of the evidence before me I am of the view that a reasonable abatement of the rent would be $704.22 which represents a 70% rent abatement for the period commencing December 7th (the date the Tenant notified the Landlords) and January 12, 2010 (the date that the inspection confirmed the unit was bedbug free).

20. The Tenant requested compensation for the mattress and sofa that she disposed of on the advice of the exterminator. The situation surrounding this is rather unique. In most cases a tenant would notify a landlord of a maintenance issue and that landlord would arrange to correct the problem. If the problem was not corrected to the tenant's satisfaction, the tenant would normally notify the landlord again and the landlord would decide on the next course of action. In the case before the Board, the Landlords removed

themselves from the equation and permitted the Tenant to contact a third party contractor (the pest control company) directly to correct the problem. In doing so the pest control company or in this case, the exterminator, effectively became an agent of the Landlords. It was in this capacity that the exterminator instructed the Tenant to dispose of her mattress and sofa and therefore the Tenant is entitled to be compensated for those two items.

2010 CanLII 37783 (ON L.T.B.)

21. The Tenant submitted into evidence an on-line price for a mattress in the amount of $639.99 which the Tenant asserts is of similar quality to the one she disposed of. The Landlords did not provide any submissions as to what they believed the value of the mattress to be. Based on the evidence, I am satisfied that the reasonable costs that the Tenant will incur to replace the mattress as a result of the Landlords' breach to be $639.99.

22. The Tenant submitted that the sofa disposed of was a mid-century Danish teak three seat sofa. The Tenant puts the value at $2,000.00. The Tenant submitted into evidence an EBay screen that reflects a "Vintage Volther Poul Danish Teak 3-Seat Sofa Couch" offered for sale at $3,000.00 USD. I am not satisfied based on the evidence before me that this sofa is an appropriate comparable. There was no evidence before me that the sofa was a "Volther Poul" model. The onus is on the party requesting damages to provide sufficient evidence to support a finding of the value of that item. In the absence of that evidence, and based on common sense and my own experience, it seems to me a reasonable replacement for the Tenant's sofa would be $500.00.

23. The Tenant's request for compensation for other furnishings and bedding in the rental unit that she discarded or left behind for the Landlord to discard in the amount of $3,689.99 is denied. Unlike the circumstances surrounding the mattress and sofa that were disposed of on the recommendation of the exterminator, the Tenant took it upon herself to discard these items. More importantly section 16 of the Act says:

> *When a landlord or a tenant becomes liable to pay any amount as a result of a breach of a tenancy agreement,* ***the person entitled to claim the amount has a duty to take reasonable steps to minimize the person's losses****.* [Emphasis added]

In this instance, the Tenant took it upon herself to discard or abandon many of her furnishings and bedding for fear of bringing the bedbugs to her new rental unit. The onus is on the Tenant to demonstrate that she took reasonable steps to mitigate the Landlords' damages by laundering or treating the items. Based on the evidence before me, I do not find that to be the case. It is understandable the Tenant wanted to put this unfortunate incident behind her however it does not relieve her of her obligation to minimize the Landlords' losses.

24. The Tenant's request for compensation for out of pocket expenses for laundering her clothing and bedding in the amount of $135.00, plastic bags for laundered items for $26.37 and two boxes in the amount of $19.98 is granted pursuant to subparagraph 30(1)5,ii of the Act. These items are directly related to the Landlords' contractual breach and are reasonable.

25. The Tenant’s request for compensation for out of pocket expenses for medical expenses in the amount of $90.00 is granted pursuant to subparagraph 30(1)5,ii of the Act. These items are directly related to the Landlords’ contractual breach and are reasonable.

26. The Tenant’s request for compensation for moving expenses, public storage and plastic bins in the amount of $651.12 is denied. Subsection 31(2) says that the Board may award moving costs where the actions of the Landlord induced the Tenant to vacate the rental unit. Based on the evidence before me, I do not find that to be the case. It was not the Landlords’ actions that induced the Tenant to move out; rather it was the presence of pests in the unit that caused her to move. The Landlords took immediate action when notified of the presence of bedbugs in the rental unit. The rental unit was treated seven times in the period between December 7th and December 31st and in addition the Landlords replaced the carpeting in the unit. Although the initial extermination efforts were unsuccessful, I am of the view that the Landlords’ actions cannot be said to have caused the Tenant to move out.

27. The Tenant request that the tenancy be terminated on January 18, 2010 is also denied. Guideline # 5 states termination of the tenancy should only be granted in serious cases; for example, where the rental unit is unfit for habitation. It was the evidence before me that the rental unit was free of pests as of January 12, 2010 so it cannot be said the situation was so serious that it warrants terminating the tenancy.

2010 CanLII 37783 (ON L.T.B.)

<u>March 3, 2010</u>
Date Issued

Guy Savoie
Vice Chair, Landlord and Tenant Board

Toronto South Region
2nd Floor, 79 St. Clair Ave. E
Toronto ON M4T 1M6
