# Supplementary material for: Analysis of 44 Cases before the Landlord and Tenant Board Involving Bed Bug Infestations in Ontario, Canada: Focus on Adjudicator Decisions Based on Entomological/Pest Management Evidence and Accountability under the Residential Tenancy Act and Other Applicable Legislation
Source: Insects. 2011 Jul 19;2(3):343–53. doi: 10.3390/insects2030343 (PMC4553548; doi:10.3390/insects2030343)
Supplement: Supplementary File 1 [file insects-02-00343-s001.zip › supplementary-2/2010canlii44231.pdf]

Landlord and Tenant Board

Order under Sections 30 and 31
Residential Tenancies Act, 2006

File Number: TET-04982-10

2010 CanLII 44231 (ON L.T.B.)

AM and SM (the 'Tenants') applied for an order determining that SHL (the 'Landlord') or the Landlord's superintendent or the Landlord's agent substantially interfered with the reasonable enjoyment of the rental unit or residential complex by the Tenants or by a member of their household (T2).

The Tenants also applied for an order determining that the Landlord failed to maintain the rental unit as stipulated by the Residential Tenancies Act, 2006 (T6).

This combined application was heard in Whitby on June 22, 2010.

The Tenants and the Landlord's representative, CJ, attended the hearing.

Relevant facts heard:

a. The Tenants moved into this rental unit in September 2009 and the rent was set at $675.00 per month.
b. In December the Tenants asserted that an upstairs unit (201) had bedbugs. Management sprayed immediately (three times) and the bugs were gone.
c. In January 2010 the Tenants discovered bedbugs in their rental unit.
d. A new Property Manager took over February 10, 2010, was notified of the bedbug problem in March.
e. In mid-March one half of the unit was sprayed. The Tenants later gave a mason jar containing live bedbugs to the Landlord and he ordered another spray but there was no third spray. The Landlord argued that the Tenants left before the third spray took place (June 1, 2010).
f. The Tenants moved out of the rental unit on May 31, 2010.
g. The Tenants threw the following items in the garbage, not wanting to take infested possessions with them to a new residence: Couch worth $150.00; Curtains worth $100.00; Bedding worth $100.00; Mattress and box spring worth $300.00 and a computer chair worth $30.00.
h. The Tenants requested a rent abatement of $3,473.00.
i. The Tenants stated that the Landlord seriously interfered with their enjoyment of the rental unit by letting go the former Superintendent and removing video cameras. The landlord argued that the Superintendent was an elderly lady who still resides in the building. The cameras were hers and the Landlord no longer wanted them in place for the privacy of the Tenants.

Determinations:

1. I find on facts heard that the Landlord or the Landlord's superintendent or the Landlord's agent substantially interfered with the reasonable enjoyment of the rental unit or residential complex by the Tenants or by a member of their household.

2. I find, the Landlord failed to maintain the rental unit as required by the RTA and did not control the spread of bedbugs.

It is ordered that:

1. The Landlord shall pay to the Tenants $680.00 for replacing belongings that were infested by bedbugs.

2. The Landlord shall also pay to the Tenants $135.00 as a rent abatement for the 2 months since reporting the bedbugs.

3. The Landlord shall pay to the Tenants $45.00 for the cost of filing the application.

4. The total amount the Landlord owes is $860.00.

5. The Landlord shall pay the Tenants the full amount owing by July 4, 2010.

6. If the Landlord does not pay the Tenants the full amount owing by July 4, 2010, the Tenant will owe interest. This will be simple interest calculated from July 5, 2010 at 2.00% annually on the balance outstanding.

7. The Tenants have the right, at any time, to collect the full amount owing or any balance outstanding under this order.

2010 CanLII 44231 (ON L.T.B.)

June 23, 2010
Date Issued

Gerald Taylor
Member, Landlord and Tenant Board

Toronto East-RO
2275 Midland Avenue, Unit 2
Toronto ON M1P3E7

If you have any questions about this order, call 416-645-8080 or toll free at 1-888-332-3234.
