# Supplementary material for: Analysis of 44 Cases before the Landlord and Tenant Board Involving Bed Bug Infestations in Ontario, Canada: Focus on Adjudicator Decisions Based on Entomological/Pest Management Evidence and Accountability under the Residential Tenancy Act and Other Applicable Legislation
Source: Insects. 2011 Jul 19;2(3):343–53. doi: 10.3390/insects2030343 (PMC4553548; doi:10.3390/insects2030343)
Supplement: Supplementary File 1 [file insects-02-00343-s001.zip › supplementary-2/2010canlii44277.pdf]

Landlord and Tenant Board

Ontario

Order under Subsection 87(1)
Residential Tenancies Act, 2006

File Number: TEL-04584-10

2010 CanLII 44277 (ON L.T.B.)

(A.A.) (the 'Landlord') applied for an order requiring (D.M.) (the 'Tenant') to pay the rent that he owes.

This application was heard in Toronto on June 8, 2010.

The Landlord’s representative (E.P.) and the Tenant attended the hearing. The Tenant was assisted by (A.M.) duty counsel.

Determinations:

1. The Tenant was in possession of the rental unit on the date the application was filed.

2. The Tenant did not pay the total rent he was required to pay for the period from May 1, 2010 to May 31, 2010.

3. The Tenant stated at the hearing that he had been the subject of a bed bug invasion in April of 2010. This information was agreed to by the Landlord and it appears to me that the Landlord took care of the problem in an admirable manner. Further it was agreed by both parties that the Tenant never complained after the treatment of any continuation of the bug problem.

4. The only matter of contention was the amount of time that passed between the Tenant informing the Landlord through the Superintendent of the problem and the start of the treatment which was May 7, 2010.

5. It was the Tenants recollection that it was the middle of April when he first told the Superintendent of the problem. While being cross examined, the superintendent was unsure as to what the date actually was but agreed that it was in April and may have been mid month.

6. Based on the two having somewhat similar recollections it appears that the action took some two and a half to three weeks to occur which is far too long for a serious problem such as bed bugs.

7. Since the Landlord did a very good job in cleaning up the problem once started, I have limited the award of rental abatement to 20% of one months rent or $244.32.

It is ordered that:

1. The Tenant shall pay to the Landlord $973.69, which represents the amount of rent owing up to May 31, 2010 and less the amount of the abatement the Landlord owes the Tenant.

2. The Tenant shall also pay to the Landlord $170.00 for the cost of filing the application.

3. If the Tenant does not pay the Landlord the full amount owing on or before July 31, 2010, the Tenant will start to owe interest. This will be simple interest calculated from August 1, 2010 at 2.00% annually on the balance outstanding.

June 10, 2010
Date Issued

Jim McMaster
Member, Landlord and Tenant Board

Toronto East-RO
2275 Midland Avenue, Unit 2
Toronto ON M1P3E7

If you have any questions about this order, call 416-645-8080 or toll free at 1-888-332-3234.

2010 CanLII 44277 (ON L.T.B.)
