# Supplementary material for: Analysis of 44 Cases before the Landlord and Tenant Board Involving Bed Bug Infestations in Ontario, Canada: Focus on Adjudicator Decisions Based on Entomological/Pest Management Evidence and Accountability under the Residential Tenancy Act and Other Applicable Legislation
Source: Insects. 2011 Jul 19;2(3):343–53. doi: 10.3390/insects2030343 (PMC4553548; doi:10.3390/insects2030343)
Supplement: Supplementary File 1 [file insects-02-00343-s001.zip › supplementary-2/2010canlii48718.pdf]

Landlord and Tenant Board

Ontario

Order under Section 30
Residential Tenancies Act, 2006

File Number: SOT-05596-10

2010 CanLII 48718 (ON L.T.B.)

L. W. and L. W. (the 'Tenants') applied for an order determining that A. T. (the 'Landlord') failed to meet the Landlord's maintenance obligations under the *Residential Tenancies Act, 2006* (the 'Act') or failed to comply with health, safety, housing or maintenance standards. Specifically, the Tenants allege there is a bedbug infestation; a mice infestation; a cockroach infestation; a broken mailbox; an inadequate heating system; a crack in the bathroom ceiling; broken kitchen appliances; and broken laundry facilities. The Tenants are also requesting reimbursement for paint and changing the locks.

This application was heard in Hamilton on June 17, 2010.

The Tenants and the Landlord and the Landlord’s representative attended the hearing. The Landlord’s superintendent testified at the hearing.

Determinations:

1. The Landlord breached his obligation under S. 20 of the Act. There are bedbugs, mice and cockroaches in the unit, a crack in the bathroom ceiling; broken laundry facilities and kitchen appliances; and a broken mailbox.

2. I find the Landlord’s actions in response to the Tenants’ complaints about the heat, mailbox and appliances have been reasonable and expeditious.

3. I find the Tenants are not entitled to compensation for changing the locks.

4. The Landlord became aware of a possible leak in the bathroom ceiling at the hearing. He has undertaken to address the issue.

5. The Tenants signed a rental agreement that included a clause requiring the Landlord’s consent to paint the rental unit. I am satisfied that the Tenants did not do so. Nor did they prove on a balance of probabilities that it was necessary to paint the unit.

6. The Tenants are entitled to a rebate of $1300.00 for the maintenance issues for the reasons attached to this order which represents 25% abatement of rent for the months December 2009 to July 2010 inclusive.

7. The Tenants are entitled to compensation for the reasons attached to this order in the amount of $300.00 for the couch; $340.00 for two mattress covers; $150.00 for the box spring and $80.00 for laundry expenses.

8. It is the intention of the Tenants to move out of the rental unit. Considering the multiple infestations the Tenants have had to live with in this unit, the Tenants are given the right

to terminate their tenancy on 10 days written notice if this notice is given on or before September 1, 2010.

It is ordered that:

1. The Landlord shall pay the Tenants a rebate of $1300.00 for the maintenance issues which represents 25% abatement of rent for the months December 2009 to July 2010 inclusive.

2. The Landlord shall pay the Tenants compensation in the amount of $300.00 for the couch; $340.00 for two mattress covers; $150.00 for the box spring and $80.00 for laundry expenses. The total amount of compensation is $870.00.

3. The Tenants have the right to terminate their tenancy on 10 days written notice if this notice is given on or before September 1, 2010.

4. The Landlord shall pay the sum of money that is owed as a result of this order. If the Landlord does not pay the Tenants the full amount owing by August 1, 2010 the Landlord will owe interest. This will be simple interest calculated from August 2, 2010 at 2%.

5. The Tenants have the right at any time to collect the full amount owing or any balance outstanding under this order.

2010 CanLII 48718 (ON L.T.B.)

<u>July 7, 2010</u>
Date Issued

Shirley Collins
Member, Landlord and Tenant Board

Southern-RO
119 King Street West, 6th Floor
Hamilton ON L8P4Y7

If you have any questions about this order, call 416-645-8080 or toll free at 1-888-332-3234.

File Number: SOT-05596-10

REASONS

2010 CanLII 48718 (ON L.T.B.)

Reasons to Order SOT-05596-10 issued on July 7, 2010 by Shirley Collins.

Infestations

The Landlord testified he purchased the rental complex in March 2009. At that time he knew of the cockroach and mice infestations but not the bedbug infestation. He contracted a pest control company in May 2009 to address all infestations. The contractor treats the common areas and if there is a complaint about a specific unit he treats that unit. As tenants moved out of the complex the units were cleaned, painted, and the carpets were replaced with hardwood or laminate floors. The Tenants' unit was painted and hardwood floors were installed before they moved in.

The tenants testified they are brother and sister and moved into the rental unit on October 31, 2009. They started painting the unit on October 27, 2009 at which time they first noticed cockroaches. They told the superintendent about the roaches but he didn't follow through on his commitment to spray at that time.

On November 14, 2009, they first noticed bedbugs. They became aware that there had been an ongoing problem of bedbugs in the building for six months. The Tenants informed the Landlord of the problem but it took until January 27, 2010 for the Landlord to arrange to have the unit treated. A month later the bed bugs returned.

The Tenants made the Landlord aware of the bedbug infestation in November 2009. He testified he gathered information about the best approach to rid the complex of bedbugs by contacting the municipality and two pest control companies. He sent a letter to all of the tenants on December 31, 2009 about the problem and suggested a course of action which included all tenants cleaning their units and the Landlord replacing carpeting in common areas with new flooring before beginning chemical treatments. On January 9, 2010 he followed up with another letter informing all tenants to prepare their units for the treatment which was starting on the top floor that week. He then sent a letter dated January 20, 2010 stating the treatment would start January 27, 2010.

The Tenants testified they first noticed mice in their kitchen on November 18, 2009 and informed the Landlord and superintendent. The superintendent and a pest control company employee attended at the unit and placed bait traps. The Tenants claim this attracted more mice to the apartment and therefore made the problem worse throughout their unit. The mice damaged their mattress and couch. It was necessary to dispose of the couch. The Landlord testified that when the Tenants complained about the mice, the pest control contractor suggested using a particular type of bait because the Tenants own a dog.

On December 31, 2009 the Landlord provided a letter to all of the tenants advising them of a bedbug infestation in the building and proposing a course of action which included cleaning all common areas and units, replacing carpeting with vinyl flooring in common areas and treating all units with chemicals. Tenants were provided with bedbug treatment preparation information.

The Tenants would like to move but feel morally obligated to disclose the bedbug infestation to any prospective landlord. Therefore they do not want to move their furniture and the bedbug problem with them.

2010 CanLII 48718 (ON L.T.B.)

Heat

The rental unit is heated by radiators. The Tenants testified that the knob on the radiator is broken and therefore they cannot control the heat. The problem was reported to the Landlord and he arranged for a service call by a heating and refrigeration company. As the radiator would have to be removed and part of the floor needed to be cut out exposing the lower unit it was recommended that the work be done in warmer weather.

Mailbox

The Tenants testified that the mailboxes have been regularly broken into causing damage to the boxes. Their mail has been stolen and Canada Post has stopped delivery for a few days on occasion. The superintendent testified that whenever it comes to his attention the mailbox is broken the repairs are made.

Painting

The Landlord and the superintendent testified the rental unit had been freshly painted but the Tenants didn’t like the colour and painted without his permission. The rental agreement signed by the Parties has a clause which states a tenant must obtain the Landlord’s prior written consent to paint or wallpaper the premises or install any panelling. The Tenants’ testified they had permission from the superintendent to paint. The superintendent testified the female Tenant talked to him about painting and he told her to put her request in writing.

Locks

The Tenants testified that they had to change the locks on the door and give the superintendent a key because the lock wasn’t changed by the Landlord when they moved in. The superintendent testified that he changed the locks prior to the Tenants moving into the unit. He received a key from the Tenants after they changed the locks. He didn’t know the locks had been changed by the Tenants until afterwards.

Bathroom Ceiling

The Tenants testified there is a crack in the bathroom ceiling. The ceiling is stained and the drywall is crumbling. The Landlord testified that there is an advertising billboard on the top of the building and the advertising company is responsible for any damage caused by the sign. When there was a leak in the bathroom ceiling of the unit last year the company responded, patched it

and suggested waiting to see if there were any further problems. The Landlord said he wasn't aware of any leak but he would contact the advertising company to make the repair.

Kitchen Appliances

The Tenants testified that the refrigerator and stove were broken when they moved into the unit and they asked that both be replaced. The refrigerator freezes all of their food. The stove was replaced with another broken stove. The week of the hearing that stove was replaced again.

The Landlord testified he checked the appliances prior to the Tenants moving in and they were in working order. The Tenants wanted new ones and he told them he couldn't afford to replace all of the appliances in all of the units. When the Tenants complained the stove wasn't working he checked it and found that someone had cut a wire to the element. He arranged for the stove to be replaced with another used stove that has a one year warranty.

Laundry Room

The Tenants testified that they are unable to use the facilities in the laundry room at times because of people breaking in to steal money out of the machines. The Landlord testified that he installed a camera in the laundry room in September 2009 and has provided the local polices services with the evidence. The machines are fixed as break-ins come to his attention.

Analysis and Findings

The Landlord breached his obligation under S. 20 of the Act. There are mice, cockroach and bedbug infestations; a crack in the bathroom ceiling; broken laundry facilities and kitchen appliances; and a broken mailbox. There was uncontested testimony that the Tenants reported the cockroach infestation on October 27, 2009, just a few days prior to moving in.

The Landlord knew about the pest infestations before the Tenants moved into the unit and he did not disclose this to them. The bedbugs were first noticed in the Tenants' unit on November 14, 2009. The Tenants did not testify as to the date they first informed the Landlord of the bedbugs but they submitted emails including one which is dated December 10, 2009 wherein the female Tenant appears to bring the problem to his attention for the first time. The first treatment of the Tenants' unit took place January 27, 2010. Though the Landlord has a pest control company on contract and testified the company acts on complaint, the Landlord decided to take time to put together a plan to eliminate the problem. The Landlord has a responsibility to take action within a reasonable period of time. He did not do so. He is making serious efforts to contain and eliminate the infestation but more than six weeks is an unreasonable amount of time for Tenants to be expected to wait for a first treatment.

I find the Landlord's actions in response to the Tenants' complaints about the heat, mailbox and appliances have been reasonable and expeditious. I am satisfied that the Landlord and superintendent have taken appropriate action to respond to the mailbox and laundry room break-ins. The Landlord has installed video cameras and has provided the evidence to police services.

I find the superintendent credible as he gave his testimony in an honest and forthright manner. He changed the locks before the Tenants moved into the unit. The Tenants may have acted on an

2010 CanLII 48718 (ON L.T.B.)

assumption that the locks had not been changed. The Tenants are not entitled to compensation for changing the locks.

The Landlord became aware of a possible leak in the bathroom ceiling at the hearing. He has undertaken to address the issue.

The Tenants signed a rental agreement that included a clause requiring the Landlord’s consent to paint the rental unit. I am satisfied that the Tenants did not do so. Nor did they prove on a balance of probabilities that it was necessary to paint the unit.

Compensation and Rent Abatement

The Tenants are claiming reimbursement for replacement furniture and an abatement of rent. I am satisfied it is necessary for the Tenants to replace their bed and couch due to the damage from the mice. They were required to purchase two mattress covers due to the infestations at a cost $170.00 each. The Tenants laundered all of their clothes and linens at a cost of $80.00 due to the bedbug infestation.

At the hearing the Tenants said the cost of replacing the couch is $500.00 and they have purchased a replacement bed for $788.96. In their application the Tenants request $300.00 for the couch and $150.00 for a box spring. The Tenants did not justify the higher amounts put forward at the hearing. The Tenants are entitled to $300.00 for the couch; $340.00 for the mattress covers; $150.00 for the box spring and $80.00 for laundry expenses.

An abatement of rent is justified in this case. In determining the abatement to be ordered factors for consideration include the period of time that the problem existed and the severity of the problem and the impact on a tenant. In addition, the rebate should not be used as or seen as punishment for the landlord’s conduct or inaction. Rather it is compensation to a tenant for the inadequate state of repair and any inconvenience or actual loss of use of the rental unit or any portion thereof. Taking into consideration the nature, severity, and duration of the problems and the impact on the Tenants, I find the Tenants are entitled to a rebate of rent in the amount of $1300.00 for the maintenance issues which represents 25% monthly rent rebate for the months of December 2009 to July 2010 inclusive. It is the intention of the Tenants to move out of the rental unit. Considering the multiple infestations the Tenants have had to live with in this unit, the Tenants are given the right to terminate their tenancy on 10 days written notice if this notice is given on or before September 1, 2010.

July 7, 2010
Date Issued

Shirley Collins
Member, Landlord and Tenant Board

Southern-RO
119 King Street West, 6th Floor
Hamilton ON L8P4Y7

2010 CanLII 48718 (ON L.T.B.)
