# Supplementary material for: Analysis of 44 Cases before the Landlord and Tenant Board Involving Bed Bug Infestations in Ontario, Canada: Focus on Adjudicator Decisions Based on Entomological/Pest Management Evidence and Accountability under the Residential Tenancy Act and Other Applicable Legislation
Source: Insects. 2011 Jul 19;2(3):343–53. doi: 10.3390/insects2030343 (PMC4553548; doi:10.3390/insects2030343)
Supplement: Supplementary File 1 [file insects-02-00343-s001.zip › supplementary-2/2010canlii48769.pdf]

Landlord and Tenant Board

Ontario

Order under Section 30
Residential Tenancies Act, 2006

File Number: NOT-02075-10

2010 CanLII 48769 (ON L.T.B.)

DM (the 'Tenant') applied for an order determining that BN (the 'Landlord') failed to meet the Landlord's maintenance obligations under the *Residential Tenancies Act, 2006* (the 'Act') or failed to comply with health, safety, housing or maintenance standards.

This application was heard in Sudbury on July 7, 2010.

The Tenant and the Landlord attended the hearing. Also appearing were PS as witness for the Tenant and CM, as witness for the Landlord.

Determinations:

1. The Tenant had bug bites on his body in the third week of April, 2010. He was told, by his family doctor, to wash all his clothes in hot water and bag them. He purchased two cans of bug spray and sprayed areas of his rental unit.

2. The Tenant reported the problem to the district health unit, who inspected the rental unit. The inspector informed the Tenant that the bugs were not “bed bugs” and could be “plant bugs”. The inspector told the Tenant to wash his clothes in hot water.

3. The Landlord was told of the “bug problem” on May 26, 2010, when the tenant reported it to CK, the building superintendent. CK immediately inspected the rental unit and although she saw bugs she could not determine their origin or type.

4. The Landlord immediately had the rental unit inspected by Martin Pest Control on May 29, 2010. The inspection did not find bed bugs and the exterminators did not recommend any spraying or other remedial action. They recommended the Tenant wash his clothing in hot water and drying with high heat, in the event the problem was body lice.

5. In all applications before the Board the onus rests with the applicant to prove his case on the balance of probabilities.

6. The Tenant has proven that there is some type of bug present in his rental unit but has not proven that the Landlord is in any way responsible for their presence, either directly or through a lack of maintenance.

7. I cannot find that the Landlord has failed to meet his maintenance obligations under the *Residential Tenancies Act, 2006*. Upon being advised of the “bug problem” the Landlord took all reasonable steps to investigate the problem by requesting an investigation by the appropriate professional exterminators. The Landlord has not failed to take any steps recommended by the exterminators or the district health unit.

It is ordered that:

1. The Tenant's application is dismissed.

2010 CanLII 48769 (ON L.T.B.)

July 12, 2010
Date Issued

Lisa Stevens
Member, Landlord and Tenant Board

Northern-RO
199 Larch Street, Suite 301
Sudbury ON P3E5P9

If you have any questions about this order, call 416-645-8080 or toll free at 1-888-332-3234.
