# Supplementary material for: Analysis of 44 Cases before the Landlord and Tenant Board Involving Bed Bug Infestations in Ontario, Canada: Focus on Adjudicator Decisions Based on Entomological/Pest Management Evidence and Accountability under the Residential Tenancy Act and Other Applicable Legislation
Source: Insects. 2011 Jul 19;2(3):343–53. doi: 10.3390/insects2030343 (PMC4553548; doi:10.3390/insects2030343)
Supplement: Supplementary File 1 [file insects-02-00343-s001.zip › supplementary-2/2010canlii48882.pdf]

Landlord and Tenant Board

Order under Section 87(1)
**Residential Tenancies Act, 2006**

**File Number:** TSL-03422-10

2010 CanLII 48882 (ON L.T.B.)

CIiGNOI (the 'Landlord') applied for an order to terminate the tenancy and evict D B, L B, J (J) B (the 'Tenants') because the Tenants did not pay the rent that the Tenants owe.

This application was heard in Toronto on May 26, 2010. The Landlord's representative, MPZ, and J (J) B (JB) attended the hearing.

At the hearing the Tenants raised the following issues pursuant to section 82 of the *Residential Tenancies Act, 2006* (the 'Act'): the Tenants alleged that the Landlord failed to meet its maintenance obligations with respect to a bed bug infestation in the unit.

**Determinations:**

1. The Tenants were in possession of the rental unit on the date the application was filed. The unit was primarily occupied by JB and his roommate.

2. The tenancy was terminated by mutual consent on May 31, 2010.

3. The Tenants have not paid the total rent they were required to pay for the period from January 1, 2010 to May 31, 2010.

4. The Tenants paid $566.00 after the application was filed.

5. The Tenants alleged that the Landlord failed to meet its maintenance obligations with respect to a bed bug infestation in the unit. JB testified that on January 4, 2010 he informed the Landlord's superintendent that there were bed bugs in his unit and that the superintendent promised to take some action. According to JB, nothing had been done by the end of January 2010, so he reported the problem to the management office. JB's evidence with respect to when and how he informed the Landlord of the problem was not contradicted. It is not contested that the unit was first treated for bed bugs on February 23, 2010 and that at that time the unit was messy, and cluttered, with clothes everywhere. The Landlord's superintendent asked the pest control technician to do his best under the circumstances. While JB claimed he did not receive detailed instructions with respect to preparing his unit for treatment, suffice it to say that the pest control technician would have done a more effective job had the unit been tidy. The Landlord refused to treat the unit again, but later relented. In March 2010, JB refused the pest control technician entry to treat the unit for bedbugs on the ground that he did not receive 24 hours' written notice and that, because of his asthma, he would have to out of the unit for 24 hours. The unit was treated in early April 2010, but the Tenant rejected a follow-up treatment in May 2010.

2010 CanLII 48882 (ON L.T.B.)

6. By not responding to the bed bug infestation between January 4, 2010 and February 22, 2010, I find that the Landlord failed to fulfill its maintenance obligations under the Act. The Tenants are entitled to an abatement of rent of $380.00, which is approximately 20 percent of the rent for that period. I decline to award an abatement of rent after February 23, 2010, because JB hindered the Landlord's efforts to treat the unit. The abatement ordered reflects the fact that the Tenants were able to use the unit for most purposes. The main problem was dealing with bed bug bites at night. JB testified that, notwithstanding the infestation, he held a meeting in the unit and entertained friends on occasion.

7. The Tenants' claim for compensation for discarding bedding and a mattress is denied. JB did not provide proof of that he owned any of the items claimed. He did not provide reliable evidence as to the value of those items. Finally, he did not establish the items could not be laundered or cleaned.

**It is ordered that:**

1. The Tenants shall pay to the Landlord $3,026.00, which represents the amount of rent owing up to May 31, 2010 and less the amount of the abatement the Landlord owes the Tenants.

2. The Tenants shall also pay to the Landlord $170.00 for the cost of filing the application.

3. If the Tenants do not pay the Landlord the full amount owing on or before July 26, 2010, the Tenants will start to owe interest. This will be simple interest calculated from July 27, 2010 at 2.00% annually on the balance outstanding.

**July 15, 2010**
**Date Issued**

_________________________
Egya Sangmuah
Member, Landlord and Tenant Board

Toronto South-RO
79 St. Clair Avenue East, Suite 212, 2nd Floor
Toronto ON M4T1M6

If you have any questions about this order, call 416-645-8080 or toll free at 1-888-332-3234.
