# Supplementary material for: Analysis of 44 Cases before the Landlord and Tenant Board Involving Bed Bug Infestations in Ontario, Canada: Focus on Adjudicator Decisions Based on Entomological/Pest Management Evidence and Accountability under the Residential Tenancy Act and Other Applicable Legislation
Source: Insects. 2011 Jul 19;2(3):343–53. doi: 10.3390/insects2030343 (PMC4553548; doi:10.3390/insects2030343)
Supplement: Supplementary File 1 [file insects-02-00343-s001.zip › supplementary-2/2010canlii58936.pdf]

Landlord and Tenant Board

Ontario

Order under Section 30
Residential Tenancies Act, 2006

File Number: SWT-11556-10

2010 CanLII 58936 (ON L.T.B.)

SS (the 'Tenant') applied for an order determining that RTA (the 'Landlord') failed to meet the Landlord's maintenance obligations under the *Residential Tenancies Act, 2006* (the 'Act') or failed to comply with health, safety, housing or maintenance standards.

This application was heard in Windsor on August 19, 2010. The Tenant attended the hearing. BP attended the hearing as the Landlord’s legal representative. DL attended the hearing as agent for the Landlord.

Preliminary Matters:

1. There was a preliminary issue about whether the proper parties were named in the application. The Tenant applied against BD. The Landlord’s legal representative submitted that the proper name of the Landlord is RTA, which is the name of the company that operates the residential complex. The Tenant submitted that BD is the owner of the residential complex because “everybody knows he is” but she made her rent cheques out to RT. The Landlord was permitted to file a copy of written tenancy agreement as a post-hearing submission. On August 21, 2010, the Board received a copy of the tenancy agreement, which names RTA as the Landlord. The application is amended to accord with the tenancy agreement.

2. There was no issue about service of the application or Notice of Hearing. The parties were prepared to proceed with the hearing.

Determinations and Reasons:

1. The rental unit is an apartment located on the third floor of a residential complex.

2. The Tenant moved into the rental unit in 1998 and vacated the rental unit on January 18, 2010.

3. The monthly rent was $810.00.

4. Subsection 20(1) of the Act provides that a landlord is responsible for providing and maintaining a residential complex, and the rental units in it, in a good state of repair and fit for habitation, and for complying with health, safety, housing and maintenance standards.

5. In August 2009, the Tenant began noticing bites on her body. Although her doctor suggested they were bed bug bites, the Tenant did not believe it.

6. In November 2009, the Tenant was still finding bites on her face and body.

7. From speaking to other tenants, the Tenant became aware of a bed bug problem in unit 202, and was concerned because the tenants of unit 202 moved their bedroom furniture down the hallway, into the elevator and into the garbage room of the complex where it sat for days, and the furniture was not covered in plastic before it was moved.

8. In November 2009, there was a bed bug problem in at least one other rental unit in the residential complex, as unit 404 was treated for bed bugs on November 4, 2009. There was no evidence that the Tenant was aware of this treatment.

9. On November 4, 2009, the Tenant found a bed bug on the shower curtain in the rental unit and showed the insect to the Landlord's building manager, D.L. The Tenant also reported that she was being bitten. Since a pest control technician was already in the complex to treat unit 404, D.L. had the technician inspect the Tenant's rental unit that same day. According to D.L., the technician found no bed bugs in the Tenant's rental unit. The Landlord paid the $40.00 inspection fee for the Tenant's rental unit but did not have the unit treated.

10. The Tenant continued to receive bites and on November 10, 2009, she found another bed bug in the rental unit. The Tenant spoke to the building manager, D.L., but he said he could not authorize spending another inspection fee so soon after the first inspection, and he suggested the Tenant should let him know if she finds another bed bug. The Landlord did not have the rental unit treated.

11. On November 12, 2010, the Tenant showed her bites to D.L. and to one of the Landlord's employees, J.D. D.L.. asked the Tenant who she was letting into her bedroom and J.D. commented "if you don't lie down with dogs, you won't get fleas". The Tenant told them it was the Landlord's legal responsibility to deal with the problem and the Landlord's employees told her "good luck" and "get in line". These inappropriate responses to a legitimate problem caused the Tenant's reasonable belief that the Landlord would not take the problem seriously.

12. On November 13, 2010, the Tenant found a third bed bug in the rental unit and posted a notice in the hallway informing other tenants that there was a bed bug problem in the building, that bed bugs were spotted on the third floor and that tenants had to be watchful and work together to prevent an outbreak. This annoyed the building manager, D.L., who approached the Tenant and asked when she was moving out. The Tenant gave evidence that D.L. asked her to move out more than once after she posted the notice on November 13, 2010, so she began making arrangements to move. D.L., in his evidence, did not deny asking the Tenant to move out, and I find that he did.

13. On November 16, 2010, the Tenant wrote a detailed complaint letter addressed to another of the Landlord's employees, M. In the letter, the Tenant described the bed bug problem and wrote that she was making provisions to move her belongings out, but she did not specify a termination date. Even after receiving the complaint letter, the Landlord took no action to treat the rental unit for bed bugs.

14. Having received no real assistance from the Landlord, the Tenant did some research and determined that she had to treat the rental unit to ensure she did not transfer bed bugs to

2010 CanLII 58936 (ON L.T.B.)

a new residence. The Tenant purchased a steamer unit and diatomaceous earth. The Tenant moved her furniture away from the walls, sprinkled the diatomaceous earth around the baseboards and furniture, steamed her mattress and other items, and washed her clothing and kept it in bags. The Tenant also purchased cream and herbal supplements, and used a sauna, to treat her bites. The Tenant repeated the treatments according to her understanding of the bed bug life cycle, and moved her treated belongings into storage. Slowly it appeared that the problem was brought under control. However, arranging her belongings to facilitate the treatments kept the Tenant's living space in disarray until she moved out. From December 2009 onwards, the Tenant slept in the living room with a turtleneck sweater over her face. The Tenant had to delay employment projects, specifically video shoots, until the bites on her face healed. In early January 2010, the Tenant became ill, which delayed her move until January 18, 2010.

2010 CanLII 58936 (ON L.T.B.)

15. The Landlord was already aware of a bed bug problem in the residential complex on November 4, 2009, when the Tenant made her first report. The Tenant found bed bugs in the rental unit and received multiple bites on her face and body. The rental unit was not reasonably free from pests and therefore was not in a good state of repair as required by subsection 20(1) of the Act. The Landlord had the unit inspected once, which did not resolve the problem. Despite being aware of the problem, and despite the Tenant's multiple complaints, the Landlord did not have the rental unit inspected again or treated for bed bugs by January 18, 2010, the date the Tenant moved out. Instead, the Landlord's employees made inappropriate comments and asked the Tenant when she was moving out, which was not a proper response to the problem. For these reasons, I find that the Landlord failed to meet the Landlord's obligations under subsection 20(1) of the Act.

16. The Tenant sought an abatement of rent. Considering the impact of the Landlord's breach on the Tenant and on her enjoyment of the rental unit, I find that the Tenant is entitled to an abatement of 50% of the rent for the period from November 4, 2010, the date of first complaint, to January 18, 2010, the date she moved out. The abatement is calculated as follows. For November 2009: 27 days X $26.63 per day X 0.50, which totals $359.51. For December 2009: $810.00 X 0.50, which totals $405.00. For January 2010: 18 days X $26.63 per day X 0.50, which totals $239.67. The total rent abatement owed to the Tenant is $1,004.18.

17. The Tenant sought compensation of $285.00 for expenses she incurred because of the Landlord's failure to maintain the rental unit. The Tenant submitted that her storage expenses were not part of her claim. The Tenant's claim for the steamer unit, mattress covers and turtleneck sweater are denied because the Tenant retains those items for her continuing benefit. The Tenant's claim for herbal products (black seed oil, florasone, eucalyptus essential oil and red clover) is also denied because the Tenant provided insufficient evidence to establish a connection between those products and their use as a bed bug or bite treatment. The Tenant is entitled to compensation for the following reasonable expenses: sauna treatment ($31.50 including tax), diatomaceous earth ($8.91 including tax) and bandages ($6.20 including tax), which totals $46.61.

18. The Tenant did not seek an order terminating the tenancy. In her view, the tenancy terminated by agreement because the building manager, D.L., told her to move out and she complied.

19. Since the Tenant obtained a substantial portion of the relief she requested, it is appropriate for the Landlord to pay the application filing fee as costs.

2010 CanLII 58936 (ON L.T.B.)

It is ordered that:

1. The Landlord shall pay to the Tenant a rent abatement of $1,004.18.

2. The Landlord shall also pay to the Tenant $46.61, which represents the reasonable out-of-pocket expenses the Tenant has incurred because of the Landlord's failure to maintain the rental unit.

3. The Landlord shall also pay the Tenant $45.00 for the cost of filing the application.

4. The full amount the Landlord owes the Tenant is $1,095.79, and the Landlord shall pay the Tenant the full amount owing on or before September 28, 2010.

5. If the Landlord does not pay the Tenant the full amount owing on or before September 28, 2010 the Landlord will owe interest. This will be simple interest calculated from September 29, 2010 at 2.00% annually on the outstanding balance.

6. The Tenant has the right, at any time, to collect the full amount owing or any balance outstanding under this order.

September 17, 2010
Date Issued

Brenna Homeniuk
Member, Landlord and Tenant Board

South West-RO
150 Dufferin Avenue, Suite 400, 4th Floor
London ON N6A5N6

If you have any questions about this order, call 416-645-8080 or toll free at 1-888-332-3234.
