# Supplementary material for: Analysis of 44 Cases before the Landlord and Tenant Board Involving Bed Bug Infestations in Ontario, Canada: Focus on Adjudicator Decisions Based on Entomological/Pest Management Evidence and Accountability under the Residential Tenancy Act and Other Applicable Legislation
Source: Insects. 2011 Jul 19;2(3):343–53. doi: 10.3390/insects2030343 (PMC4553548; doi:10.3390/insects2030343)
Supplement: Supplementary File 1 [file insects-02-00343-s001.zip › supplementary-2/2010canlii58958.pdf]

Landlord and Tenant Board

Order under Section 30
Residential Tenancies Act, 2006

File Number: TNT-08445-10

2010 CanLII 58958 (ON L.T.B.)

ST (the 'Tenant') applied for an order determining that MLI (the 'Landlord') failed to meet the Landlord's maintenance obligations under the *Residential Tenancies Act, 2006* (the 'Act') or failed to comply with health, safety, housing or maintenance standards.

The hearing was held in Toronto on September 2, 2010.

The Tenant attended the hearing and gave testimony.

The Landlord was represented by MF. JG (property manager), AB (resident manager) and FJ (superintendent) gave evidence.

Determinations:

1. The Tenant raised a number of maintenance problems that she experienced in her unit and in the building, the most significant of which is the bedbug infestation in her unit. The Tenant raised other maintenance issues such as power surges, electric shocks on the stove and refrigerator, over-heating of electrical appliances, missing tile in the kitchen and bathroom, filthy carpet (outside the unit) and stench from the garbage bin located near her balcony.

2. The Tenant's unit is without hydro supply because of non-payment of hydro bills. Besides her direct testimony, the Tenant did not provide any objective evidence to support her claim about "power surges, electric shocks on the stove and refrigerator, over-heating of electrical appliances, missing tile in the kitchen and bathroom, filthy carpet (outside the unit) and stench from garbage bin." Hence, I decided to adjudicate only on the issue of bedbugs in this application.

3. The Tenant wrote to the Landlord on July 14, 2010, stating that as a result of the bedbug infestation in her unit, "she can no longer sleep in her bed, and the bed bugs are in her very expensive heirloom sofa, she cannot sleep at night, and her health is being endangered." The Landlord responded to the Tenant's desperate plea for help by giving her a standard form called "preparation instructions and permission to enter for bedbug control" on August 5, 2010. As a result of the delay in returning the form to the Landlord, the rental unit was not treated for bedbugs until August 30, 2010. The Tenant stated at the hearing that she continued to suffer from bedbug problem after the first spray.

4. The Landlord did not respond promptly to the Tenant's complaint about the bedbug infestation in her unit. The Tenant first complained in writing to the Landlord about bedbugs on July 14, 2010. Three weeks later, the Landlord left in the Tenant's unit a form giving her instruction on how to "prepare" her unit for pest control treatment.

Since the Landlord took no action to respond to the Tenant's complaint, the Tenant complained (in writing) to the Landlord again on August 17, 2010. She also filed a "maintenance request" with the Landlord on August 21, 2010. The Landlord then sent a pest control technician to "spray" the unit on August 30, 2010. The Landlord did not show any sense of urgency or concern in dealing with this serious problem that has been bothering the Tenant for nearly two months. The Tenant could not sit on the sofa or sleep on the mattresses because they were infested with bedbugs. She had to seek medical treatment for the allergy and infection caused by the bedbug bites. Apart from ordering a spray of the unit which took place six weeks after the Tenant first complained about the bedbug infestation, the Landlord did not send any staff to attend the rental unit to investigate and assess the problem before and after the first and only spray.

2010 CanLII 58958 (ON L.T.B.)

5. In the view, a landlord's responsibility in dealing with bedbug infestation goes beyond simply ordering a pest control treatment. The Landlord has a duty to work with the Tenant to ensure that the rental unit is totally free of bedbugs. The Landlord should also provide assistance to the tenants to "restore" the "infested properties" (i.e., mattresses, sofa, clothing, etc.) to the condition that they were in before the bedbug infestation.

6. I find that in this case the Landlord has failed to meet the Landlord's maintenance obligations under the *Residential Tenancies Act, 2006* (the 'Act') and has failed to comply with health, safety, housing or maintenance standards.

7. The Tenant claimed $300.00 for "cleaning and laundry bills, upholstery cleaning and pest control." Although the Tenant did not provide any receipts to support her claim, I find the amount of claim to be reasonable.

8. The Landlord shall pay to the Tenant a rent abatement of $517.50 to compensate for the loss of enjoyment of the rental unit by the Tenant as a result of the bedbug infestation. The evidence indicates that the Landlord did not act on the Tenant's complaint until August 30, 2010. As a result of the bedbug infestation, the Tenant has to sleep on the floor and she was bitten by the bedbugs. This abatement is based on 50% of the monthly rent for a period of 1.5 months (from July 14 to August 30, 2010.) I set the compensation rate to be higher in this case because I find that there is gross negligence and delay by the Landlord in addressing the Tenant's complaint.

It is ordered that:

1. The Landlord shall pay to the Tenant compensation in the amount of $817.50

2. The Landlord shall pay to the Tenant the application fee of $45.00.

3. The Landlord shall pay the total amount owing of $862.50 to the Tenant on or before September 20, 2010. If the Landlord does not pay this amount to the Tenant by the due date, the Tenant may deduct this amount from her future rent.

4. The hearing was held four days after the Landlord did the first treatment of the bedbugs. The Tenant complained that the bedbugs were still present in her unit after the first and only treatment. Hence, I ordered the Landlord shall take immediate action to rid the unit of any bedbugs. After this is done, the Landlord shall clean and restore the Tenant's furnishings (i.e., mattresses, sofa, clothing, etc.) to the same condition before the bedbug infestation. The Landlord shall complete all of the above work within one month after this order is issued.

5. Should the Landlord fail to comply with this order, the Tenant may advise the Landlord and the Board in writing of the Landlord's failure to comply and request that the hearing of this application be reconvened.

2010 CanLII 58958 (ON L.T.B.)

<u>September 8, 2010</u>
Date Issued

Vincent Ching
Member, Landlord and Tenant Board

Toronto North-RO
47 Sheppard Avenue East, Suite 700, 7th Floor
Toronto ON M2N5X5

If you have any questions about this order, call 416-645-8080 or toll free at 1-888-332-3234.
