# Supplementary material for: Analysis of 44 Cases before the Landlord and Tenant Board Involving Bed Bug Infestations in Ontario, Canada: Focus on Adjudicator Decisions Based on Entomological/Pest Management Evidence and Accountability under the Residential Tenancy Act and Other Applicable Legislation
Source: Insects. 2011 Jul 19;2(3):343–53. doi: 10.3390/insects2030343 (PMC4553548; doi:10.3390/insects2030343)
Supplement: Supplementary File 1 [file insects-02-00343-s001.zip › supplementary-2/2010canlii65536.pdf]

Order under Sections 30 & 31
Residential Tenancies Act, 2006

File Number: TET-07116-10

2010 CanLII 65536 (ON L.T.B.)

Tenant's T2 application.
J.P., J.P.2. and R.P. (the 'Tenants') applied for an order determining that A.W. (the 'Landlord') substantially interfered with the reasonable enjoyment of the rental unit or residential complex by the Tenants or by a member of their household.

Tenant's T6 application.
J.P.,J.P.2. and R.P. (the 'Tenants') also applied for an order determining that A.W. (the 'Landlord') failed to meet the Landlord's maintenance obligations under the *Residential Tenancies Act, 2006* (the 'Act') or failed to comply with health, safety, housing or maintenance standards.

These applications were heard in Whitby on September 28, 2010.

The Tenants and the Landlord attended the hearing.

Relevant facts:

1. The three Tenants moved into the unit on May 29, 2010 while attending school the following September. The unit is a front section of a duplex in Oshawa.

2. As a first issue, the Landlord agrees that he did not give the Tenants an address that he could be reached at. The Landlord now recognizes that this was a mistake and has corrected the problem but not before the Tenants had to "hunt" him down.

3. At the start of June 2010, Tenant J.P. started to notice bites or marks on her skin. It appears that at first she may have thought this to be hives.

4. As the month progressed it became more apparent that these were not hives and one of the Tenants determined that it was bed bugs on or about July 9, 2010. At no time in the hearing was medical evidence ever presented.

5. The Tenants informed the Landlord of the bed bug problem around the middle of July. The Landlord insists that it was the end of July however on the balance of probabilities and the existence of the Tenants rather intensive timeline of the whole incident, I find that the time of mid July is more believable.

6. The unit was first sprayed on August 16, 2010. Two of the Tenants had vacated the unit before this time and the third did so just after this time. School of course did not start until September.

7. There seems to have been poor communications between the parties. Since the Tenants had not heard of the unit being sprayed for a second time, they arranged for their own inspector to look at the unit on August 30, 2010. The Inspector found evidence of dead

bugs by the sofa but no evidence of any live bugs and no evidence of bugs in any of the bedrooms.

8. The Landlord did arrange for a second spraying of the unit but due to a mistake, never told the Tenants of the second spraying. This spraying was completed on September 9, 2010. Again it was noted one dead bug was found by the sofa but no evidence of any presence in the bedrooms at all.

9. The Tenants remain out of the unit despite the all clear from the inspector that they hired themselves.

10. At the hearing, the parties agreed that it was best to terminate the tenancy and agreed to a termination clause to be included in this order. This was done.

11. Rent is paid up to the end of August and there is a last month's rental deposit.

2010 CanLII 65536 (ON L.T.B.)

Discussion:

1. Firstly, I have awarded the Tenants the sum of $100.00 for the inconvenience that they were subject to because the Landlord failed to give them his address that he was reachable and servable at.

2. There is no doubt that there was a bed bug problem in this unit. While the Tenants may have over reacted to the situation, I can understand their fear of the bugs. However this is not the Landlord's problem either.

3. I have awarded the Tenants a 50% rental rebate for the 1 and ½ month time frame from mid July till the end of August. While the Landlord did respond, I feel he could have done so much quicker and with better communication with his Tenants. He may have even caused the program to slow down a bit at one time due to him requesting that the Tenants pay half the costs. The Landlord now knows that this is not the way to do business. The award therefore is for 1.5 x $1150 x 50% = $862.50.

4. The Landlord can not be held liable for the time leading up to mid July as he was not aware of any problems at the rental unit.

5. As the Landlord did not communicate better with the Tenants, I understand the Tenants need to have someone inspect the unit and have also awarded the $118.65 that the Tenants paid to have the unit inspected.

6. However, the Tenants inspection gave the all clear in the apartment and the Tenants could have moved back safely at that time yet they choose not to do so. Again this is a decision that they have made but should not be reflected on the Landlord.

7. As in any bed bug problem, all clothing and sheets must be thoroughly laundered so I have also awarded a sum of $300.00 to do this.

8. The Tenants had requested a large sum of money to allow them to dump any furniture in their apartment. I have disallowed this request as there is no evidence presented that would show to me that the furniture is not usable.

2010 CanLII 65536 (ON L.T.B.)

It is ordered that:

1. The tenancy between the Landlord and the Tenants is terminated as of October 9, 2010.

2. If the unit is not vacated on or before October 9, 2010, then starting October 10, 2010, the Landlord may file this order with the Court Enforcement Office (Sheriff) so that the eviction may be enforced.

3. Upon receipt of this order, the Court Enforcement Office (Sheriff) is directed to give vacant possession of the unit to the Landlord on or after October 10, 2010.

4. The Landlord shall pay to the Tenants a rent abatement of $862.50.

5. The Landlord shall also pay to the Tenants a rent abatement of $100.00 for the deliberate withholding of the Landlord's address.

6. The Landlord shall also pay to the Tenants $418.65. This represents the costs that the Tenants will incur or have already incurred in cleaning of all household items ($300.00) and also the cost of the independent ($118.65) bug inspection performed on August 30, 2010

7. The Landlord shall also pay the Tenants $45.00 for the cost of filing the application.

8. The total amount the Landlord owes the Tenants is $1,426.15.

9. The Tenants last month's rental deposit will be used for the month of September 2010 rent. (As noted at the hearing all rent was paid up to end of August, 2010.)

10. The Tenants will also pay to the Landlord 9 days of occupation in October at a cost of $37.80 per day for a sum of $340.20.

11. The total amount that the Tenants owe to the Landlord is $340.20.

12. **By taking into account lines 8 and 11, the Landlord owes the Tenants a grand total of $1,085.95**

13. The Landlord shall pay the Tenants the full amount owing by October 15, 2010.

14. If the Landlord does not pay the Tenants the full amount owing by October 15, 2010 the Landlord will owe interest. This will be simple interest calculated from October 16, 2010 at 2.00% annually on the outstanding balance.

<u>October 4, 2010</u>
Date Issued

Jim McMaster
Member, Landlord and Tenant Board

Toronto East-RO
2275 Midland Avenue, Unit 2
Toronto ON M1P3E7

If you have any questions about this order, call 416-645-8080 or toll free at 1-888-332-3234.

In accordance with section 81 of the Act, the part of this order relating to the eviction expires on April 10, 2011 if the order has not been filed on or before this date with the Court Enforcement Office (Sheriff) that has territorial jurisdiction where the rental unit is located.

2010 CanLII 65536 (ON L.T.B.)
