# Supplementary material for: Analysis of 44 Cases before the Landlord and Tenant Board Involving Bed Bug Infestations in Ontario, Canada: Focus on Adjudicator Decisions Based on Entomological/Pest Management Evidence and Accountability under the Residential Tenancy Act and Other Applicable Legislation
Source: Insects. 2011 Jul 19;2(3):343–53. doi: 10.3390/insects2030343 (PMC4553548; doi:10.3390/insects2030343)
Supplement: Supplementary File 1 [file insects-02-00343-s001.zip › supplementary-2/2010canlii65642.pdf]

Landlord and Tenant Board
Ontario

Order under Section 31
Residential Tenancies Act, 2006

File Number: TET-07190-10

2010 CanLII 65642 (ON L.T.B.)

CW and SW (the 'Tenants') applied for an order determining that ML (the 'Landlord') **or** the Landlord's agent harassed, obstructed, coerced, threatened or interfered with them and withheld or deliberately interfered with the reasonable supply of a vital service, care service, or food that the Landlord is obligated to supply under the tenancy agreement.

This application was heard in Toronto on September 28, 2010. The Tenant, SW and the Landlord's legal representative, MF and property manager, EH, attended the hearing.

<u>Appearing as witnesses for the Landlord were</u>: D.Mc., Vice President of MPC; C.H., Resident Building Manager; and S. B., Senior Resident Manager.

Determinations:

1. The attending Tenant claimed that the Landlord interfered with or withheld a vital service, because she noticed that their hot and cold water service operated adversely.

2. The Tenant's claim was based solely on assumptions on their part, and she provided no evidence that would indicate to me that any deliberate action on the Landlord's/or the Landlord's agents' part was responsible for the alleged dysfunctioning of the water service.

3. This issue is dismissed.

4. The Tenant also claimed that the Landlord/Landlord's agent harassed them in the following ways. They exterminated the neighbour's unit 10 times, while her unit was only treated 3 times; the resident manager, CH, was observed by another tenant, who the Tenant claimed did not want to attend the hearing, placing a note on the door of the Tenants' unit. The note, presented at the hearing essentially discourages the Tenants from speaking out against the Landlord, and suggest that by doing so they ran the risk of being evicted. As well, the Tenant claimed they were wrongfully accused of having a washing machine in the unit, causing malfunctioning of the drain system; and of having a satellite dish. According to the Landlord, all tenants in the building were asked to remove satellite dishes. As well, the Tenant stated that the Landlord constantly issued N4 Notices of Termination for non-payment of rent, after they had cashed the rent cheque; and issued excessive notices of entry, specifically 3 during the month of September. The Tenant provided no evidence as to the specifics regarding her claim of inappropriately served N4 Notices. The Tenant also went on to state that CH referred to her spouse using a derogatory racial slur on August 12 when her spouse called to address concerns with the resident manager.

5. The Landlord disputed the Tenant's claims and gave evidence by way of its witnesses as follows. The Landlord has a contract for regular, monthly pest control treatment, including bed bugs, of the complex, or additional treatments as required; the Tenants' unit was treated for bedbugs on August 3 and reportedly no bed bugs were found at that time; a scheduled treatment of August 26 was aborted because the Tenants' unit was not properly prepared; a September 22 treatment report revealed no bed bugs were found as well.

2010 CanLII 65642 (ON L.T.B.)

6. Based on the evidence provided, I find that the Landlord was diligent in maintaining the complex against infestations. There is nothing in the Landlord's actions in this case that would suggest that the Landlord did anything other than meet its maintenance obligations regarding this issue. Nothing in the evidence presented would lead me to believe that the Landlord was overlooking the Tenants' unit with regards to pest control treatment.

7. CH testified that the Tenants received proper 24-hour notices of entry. In September, for example, 1 notice was served for general inspection, a second one for extermination purposes which they were unable to execute as they did not get access to the unit, and a third notice was issued in place of the second one. When shown the unsigned, hand-written note the Tenant alleged he posted on the door of their unit, CH stated he had seen the note on the door of his office sometime ago, which he had removed and discarded. Compared to his writing on a folder he presented at the hearing, the handwriting in the note was evidently not that of the manager.

8. I am not persuaded by the Tenant's testimony that there was any wrong-doing on the Landlord's part in serving 24-hour notices of entry; nor am I convinced that the Landlord's agent wrote or posted the warning note as the Tenant claimed. In fact I find the circumstances surrounding the unsigned note to be doubtful, especially since the Tenant claimed it was found on the door of the rental unit, while CH recalled removing it from the door of his office. Furthermore, the Tenant was unable to produce the eyewitness who she claimed had observed the note being placed on the door of the rental unit. I find the Tenant's evidence lacks credibility.

9. The Landlord's agent also denied the Tenant's allegations that he was insulting to her husband. The agent went on to state that, contrary to the Tenant's claim that the co-Tenant came to his office on August 12 where he was allegedly insulted by him, he spoke to the co-Tenant by phone. Furthermore, CH stated that the Tenant was habitually hostile towards him.

10. With regards to the issue of the backed up drain, the Landlord's testimony leads me to believe that this was nothing more that an investigation on their part to determine the source of a backed up drainage system, and the potential cause.

11. SB further testified that the Tenant was usually combative in demeanour whenever she called, and often used derogatory remarks, including the term "jackass" in reference to CH. In fact, the Landlord presented a noted from the Tenant in response to their letter requesting a spare key, where she writes," This key is for 910. I want it back before I move from this bed bug infested dump that you pick and choose who you will spray from who you wont. (sic)"

12. I find the Tenants' claim of harassment to be unfounded. Nothing in the Tenant's evidence, on who the burden of proof rests, would lead me to make a finding that the Landlord or the Landlord's agents' actions constitute harassment.

13. I find no merit in the Tenants' claims, and I am of the view that they are frivolous and vexatious in nature.

14. The Tenants' application will be dismissed.

2010 CanLII 65642 (ON L.T.B.)

It is ordered that:

1. The Tenants' application is dismissed.

October 1, 2010
Date Issued

Claudette Leslie
Member, Landlord and Tenant Board

Toronto East-RO
2275 Midland Avenue, Unit 2
Toronto ON M1P3E7

If you have any questions about this order, call 416-645-8080 or toll free at 1-888-332-3234.
