# Supplementary material for: Analysis of 44 Cases before the Landlord and Tenant Board Involving Bed Bug Infestations in Ontario, Canada: Focus on Adjudicator Decisions Based on Entomological/Pest Management Evidence and Accountability under the Residential Tenancy Act and Other Applicable Legislation
Source: Insects. 2011 Jul 19;2(3):343–53. doi: 10.3390/insects2030343 (PMC4553548; doi:10.3390/insects2030343)
Supplement: Supplementary File 1 [file insects-02-00343-s001.zip › supplementary-2/2010canlii76098.pdf]

Landlord and Tenant Board
Ontario

Order under Section 30
Residential Tenancies Act, 2006

File Number: CET-07423-10

2010 CanLII 76098 (ON L.T.B.)

RH (the 'Tenant') applied for an order determining that NUMBERED COMPANY, (the 'Landlord') failed to meet the Landlord's maintenance obligations under the *Residential Tenancies Act, 2006* (the 'Act') or failed to comply with health, safety, housing or maintenance standards.

This application was heard in Barrie on November 10, 2010.

The Tenant and the Landlord’s representative, HL attended the hearing. The Tenant was represented by Duty Counsel, GB.

Determinations:

1. The Landlord failed to meet the Landlord's obligations under section 20 of the Act. In particular, the Landlord failed to keep the unit in a good state of repair and fit for habitation due to the bed bugs in the rental unit.

2. There was already an issue with bed bugs in the rental unit and the surrounding units before this tenancy commenced.

3. The Landlord did respond when they were made aware of the problem and hired [*Company name removed*] pest control to treat the units for bed bugs. However, despite the treatment, the bed bugs continued to be a problem.

4. The Tenant is entitled to a rent abatement of $750.00 since the rental unit was not in a good state of repair and fit for habitation as a result of the bed bugs.

5. The Tenant’s request to terminate the tenancy as of October 1, 2010 is granted. The bed bug problem began in 2009 before this tenancy commenced and was still a problem in this unit or neighbouring units at the time the Tenant vacated on October 1, 2010.

6. The Tenant’s bed (mattress and box spring) were damaged and disposed of as a result of the Landlord’s failure to keep the unit in a good state of repair and fit for habitation. The Tenant incurred expenses of $777.44 to replace the bed. His request for this amount is granted.

It is ordered that:

1. The tenancy between the Landlord and the Tenant is terminated effective October 1, 2010.

2. The Landlord shall pay the Tenant a rent abatement of $750.00.

3. The Landlord shall also pay the Tenant $777.44 for the cost the Tenant incurred to replace his bed.

4. The Landlord shall also pay the Tenant $45.00 for the cost of filing the application.

5. The total amount the Landlord owes the Tenant is $1,572.44. The Landlord shall pay the Tenant the full amount owing by November 27, 2010.

6. If the Landlord does not pay the Tenant the full amount owing by November 27, 2010 the Landlord will owe interest. This will be simple interest calculated from November 28, 2010 at 2.00% annually on the outstanding balance.

2010 CanLII 76098 (ON L.T.B.)

November 16, 2010
Date Issued

____
Karen Wallace
Member, Landlord and Tenant Board

Central-RO
3 Robert Speck Pkwy, Suite 520, 5th Floor
Mississauga Ontario L4Z2G5

Reasons for this order are attached.

If you have any questions about this order, call 416-645-8080 or toll free at 1-888-332-3234.

REASONS

Reasons to Order CET-07423-10 issued on November 16, 2010 by Karen Wallace.

2010 CanLII 76098 (ON L.T.B.)

1. The Landlord agreed that there were bed bugs in the rental unit before this tenancy commenced. An invoice from [*Company name removed*] pest control confirmed that the unit was treated on December 7, 2009. This was just three weeks before this tenancy commenced on January 1, 2010.

2. There was no dispute that there were also beg bugs in other units surrounding this rental unit.

3. The Landlord argued that they were not in breach of their maintenance obligations since they responded promptly when they became aware of the problem with bed bugs.

4. Subsection 20(1) of the *Residential Tenancies Act*, 2006 (the 'Act') states: "A landlord is responsible for providing and maintaining a residential complex, including the rental units in it, in a good state of repair and fit for habitation and for complying with health, safety, housing and maintenance standards".

5. Subsection 20(1) of the Act does not contain an element of fault. In other words, the reasons for a landlord's breach of subsection 20(1) are not relevant. Regardless of the reasons why a landlord is unable to provide a rental unit in a good state of repair and fit for habitation, if they are unable to do so, they are in breach of their obligation under the Act.

6. The Landlord's maintenance obligations and the irrelevance of fault has been referred to in court cases and was upheld by the Divisional Court in *Offredi v. 751768 Ontario Ltd* [1994] O.J. No. 1204. In that case the Divisional Court stated: "The question of fault on the landlord's part is not the issue... What the tenants claim is a breach of contract. The tenants were paying full rent for premises which the landlord was under an obligation… to keep in a good state of repair and fit for habitation. The landlord failed to do that. That is the basis for the claim for an abatement …"

7. The irrelevance of fault was discussed by the Ontario Court of Justice (General Division) in the case of *35 Charles Street West v. Metropolitan Toronto Housing Authority* [1996] O.J. No. 5469. The Court stated that the no fault approach to a landlord's obligations is consistent with the approach taken to a tenant's obligation to pay rent. A tenant is required to pay rent regardless of whether or not they have the money to pay. It is no excuse for a tenant in arrears of rent to explain they have no money to pay rent due to circumstances beyond their control.

8. The Landlord did respond when they became aware of the bed bug problem. They relied on the treatment plan by [*Company name removed*] pest control. Yet, it is clear that the Tenant did not get what he paid for. He paid the full rent for the period of time that the unit had bed bugs. The Landlord was simply unable to provide a rental unit that was in a good

state of repair and fit for habitation. Since the Landlord was in breach of subsection 20(1) of the Act, the Tenant is entitled to a rent abatement.

Amount of Rent Abatement

2010 CanLII 76098 (ON L.T.B.)

9. The Tenant requested a 100% rent abatement for August and September when the bed bugs were an active issue in his unit.

10. The amount requested is not reasonable. The Tenant continued to reside in the rental unit during this period and continued to receive many services and facilities. It is appropriate that some amount of rent be paid for the services and facilities he continued to receive.

11. Furthermore, on one of the occasions that the pest control company attended to treat the unit, the Tenant failed to fully prepare his unit for treatment. This was confirmed by the [*Company name removed*] letter dated September 27, 2010.

12. Therefore, the amount requested is reduced to a 50% rent abatement ($750.00 x 50% = $375.00 x 2 months = $750.00). The amount ordered recognizes the considerable inconvenience and discomfort caused by the bed bugs in the unit.

Damage to the Mattress

13. The Landlord argued they were under no legal obligation to inform prospective tenants of past issues with bed bugs, especially if treatment was done.

14. Yet, it was reasonably foreseeable that a new tenant moving into a unit that had problems with bed bugs may also encounter problems themselves with bed bugs.

15. As the Landlord’s representative (HL) herself stated in the letter to the Tenant dated September 1, 2010, it can take several treatments to eliminate bed bugs.

16. There was no dispute that the Tenant left his bed behind when he vacated the rental unit on October 1, 2010. The Landlord confirmed that it was left at the property.

17. The damage to the Tenant’s mattress was reasonably foreseeable given the prior outbreak of bed bugs in this unit and in the surrounding units. If the Tenant had been warned of the bed bug problem the damage could possibly have been prevented. Therefore, I find the Landlord responsible for the cost of replacing the mattress.

18. The Tenant presented an invoice from a furniture company to support the amount claimed for the bed.

Termination of the tenancy

19. There is a fixed term lease to December 31, 2010. The Tenant vacated the rental unit on October 1, 2010. The Landlord stated that the unit has been re-rented as of December 1, 2010.

20. A letter from the Landlord to tenants in the building dated September 22, 2009 confirmed that, as of September 2009, there was a minor infestation of bed bugs in a portion of the building.

21. The [*Company name removed*] invoice confirmed a live bed bug was found in the rental unit during the December 2009 treatment.

22. Letters from the resident in an adjacent unit established that bed bugs were an ongoing problem in March and April 2010.

23. Letters and invoices from [*Company name removed*] confirm that further treatment of the rental unit occurred in July, August and September 2010. The bed bugs in the unit and/or in the adjacent units had not been eliminated by the time the Tenant vacated the rental unit. The September 27, 2010 letter from [*Company name removed*] confirmed that live bedbugs were seen in the unit next door (unit 202) at the time of treatment.

24. Due to the ongoing bed bug problem it is reasonable to terminate the tenancy. The ongoing pest control in the building has not completely eliminated the bed bug infestation.

Prior Board Orders

25. The Landlord presented three prior Board decisions regarding bed bugs for consideration. Prior Board decisions are not binding on me. Furthermore, the decisions can be distinguished since they found that the landlord was not in breach of their maintenance obligations and/or that the tenants were the probable cause of the bed bug infestation. That is not the case here.

November 16, 2010
Date Issued

____
Karen Wallace
Member, Landlord and Tenant Board

Central-RO
3 Robert Speck Pkwy, Suite 520, 5th Floor
Mississauga Ontario L4Z2G5

2010 CanLII 76098 (ON L.T.B.)
