# Supplementary material for: Analysis of 44 Cases before the Landlord and Tenant Board Involving Bed Bug Infestations in Ontario, Canada: Focus on Adjudicator Decisions Based on Entomological/Pest Management Evidence and Accountability under the Residential Tenancy Act and Other Applicable Legislation
Source: Insects. 2011 Jul 19;2(3):343–53. doi: 10.3390/insects2030343 (PMC4553548; doi:10.3390/insects2030343)
Supplement: Supplementary File 1 [file insects-02-00343-s001.zip › supplementary-2/2010canlii76210.pdf]

Landlord and Tenant Board

Ontario

Order under Section 30
Residential Tenancies Act, 2006

File Number: SWT-14278-10

2010 CanLII 76210 (ON L.T.B.)

MCH (the 'Tenant') applied for an order determining that NE (the 'Landlord') failed to meet the Landlord's maintenance obligations under the *Residential Tenancies Act, 2006* (the 'Act') or failed to comply with health, safety, housing or maintenance standards. The Tenant also applied for an order determining that the Landlord, the Landlord's agent or superintendent had substantially interfered with the reasonable use and enjoyment of the premises for all usual purposes under the *Residential Tenancies Act, 2006* (the 'Act').

This application was heard in St. Thomas on October 29, 2010. The Tenant attended the hearing and was self-represented. The Landlord also attended the hearing, represented by paralegal, WH DG of EPC appeared as witness for the Landlord.

Procedural Matter:

I did not consider it necessary to hear from the Tenant's witness as, based on what the Tenant anticipated she would say, she would not have anything to say which the Tenant had not already told me. The only fact to which that person would have testified which the Tenant had not already spoken to was a fact not in dispute by the Landlord.

Determinations:

1. Based on the evidence, I find on a balance of probabilities that the Landlord failed to maintain the rental unit or residential complex in accordance with his obligations under section 20 of the Act.

2. However, a breach of maintenance obligation will not necessarily always result in an abatement of rent or other compensation being awarded to the Tenant. Where the Board finds a breach a breach of maintenance obligation, it remains for the Board to determine what remedies, if any, are reasonable and warranted in all the disclosed circumstances. In making that determination, the Board must consider whether the tenant contributed to the problem, making it worse, by failing to mitigate their losses, or by interfering with the landlord's remediation efforts. Consideration of those factors may result in the tenant not being entitled to an abatement of rent or other compensation within the Board's authority to order under the Act.

3. In this case, despite the breach arising from the presence of bed bugs in the rental unit and residential complex, I do not find the Landlord liable to the Tenant for an abatement of rent or other compensation within the Board's power to order under the Act.

4. In April of 2009, the complex became infested with bed bugs. The Landlord promptly retained a pest control contractor to inspect and treat the complex. No further bed bug activity was reported at the complex for sixteen months thereafter.

5. On August 15, 2010, the Tenant reported being bitten by something in the rental unit. After having the bites examined at the local hospital, the Tenant believed them to be spider bites. The building superintendent immediately provided the Tenant with a treatment spray for spiders. When the spray proved ineffective and the Tenant continued to be bitten in the unit, the Landlord investigated further. On August 16, 2010, the Landlord determined that the Tenant's rental unit and seven others were, in fact, infested with bed bugs.

6. EPC, the same contractor the Landlord had retained for the 2009 infestation, started treating the affected units on August 18, 2010. Treatment of the entire complex was completed on August 19, 2010. All tenants of the complex were then informed that, starting August 23, 2010, EPC would be conducting weekly inspections of all rental units to monitor its bed bug remediation efforts at the complex. This was done in accordance with EPC's remediation protocols, which had not substantially changed since the first infestation in 2009.

7. Despite reports of bed bug activity in the Tenant's rental unit after August 23, 2010, no bed bugs were found at the complex upon eight subsequent inspections by EPC. There was no evidence that any of the other tenants of the complex had complained to the Landlord of further bed bug bites after treatment was completed.

8. There was no independent medical evidence to confirm that the bites the Tenant reported after August 19, 2010 were, in fact, bed bug bites.

9. The Tenant's position was never that the Landlord had failed to take appropriate remedial steps when the infestation was reported in 2010, but that it had failed to do so in April of 2009.

10. The evidence did not support such a finding.

11. The bed bug remediation protocols followed by the Landlord and its contractor had not substantially changed between the two infestations.

12. In both cases, the Landlord acted swiftly and decisively with due regard for the seriousness of the problem and its impact upon the tenants of the complex.

13. The pest control contractor used one measure in 2010 not used in 2009. In 2010, holes were drilled into the walls of some rental units and substances were injected into the wall cavities there. However, the uncontested evidence of the Landlord's contractor was that such an approach is only followed where bed bugs are found in more than one rental unit. In 2009, specimens were found in only one rental unit, meaning that neighbouring units would need to be treated, but not calling for the spraying of wall cavities.

2010 CanLII 76210 (ON L.T.B.)

14. It was for the Tenant to prove on a balance of probabilities (*i.e.* “more likely than not”) that the contractor’s failure to treat wall cavities in 2009 resulted directly in the infestation of August 2010. There was no direct evidence to support such a finding. In the absence of direct evidence on that point, the Board was not free to engage in conjecture. It was not plain and obvious on these facts that, except for the decision not to treat wall cavities in 2009, the infestation sixteen months later would not have occurred. Too much time had past between the first event and the second to infer any causal connection on a balance of probabilities.

15. I do not find that the Tenant was being in any way untruthful in giving her evidence in this matter. I believe she was reporting to the best of her ability to the facts as she understood them. Nevertheless, a case stands to be proved (or not) based upon the evidence presented at the hearing in accordance with the Act. There was insufficient evidence to establish the Tenant’s entitlement to an order for compensation from the Landlord due to any failure to act reasonably once these infestations were discovered.

16. In light of the timely and sufficient efforts at remediation made at the time, I do not find that the Landlord, the Landlord’s agent or superintendent has substantially interfered with the Tenant’s reasonable use or enjoyment of the premises for all usual purposes under the Act.

2010 CanLII 76210 (ON L.T.B.)

All of the reasons for this order are set out above.

It is ordered that:

1. The Tenant's application is dismissed.

November 2, 2010
Date Issued

____
B. Wallace
Member, Landlord and Tenant Board

South West-RO
150 Dufferin Avenue, Suite 400
London ON N6A5N6

If you have any questions about this order, call 416-645-8080 or toll free at 1-888-332-3234.
