# Supplementary material for: Analysis of 44 Cases before the Landlord and Tenant Board Involving Bed Bug Infestations in Ontario, Canada: Focus on Adjudicator Decisions Based on Entomological/Pest Management Evidence and Accountability under the Residential Tenancy Act and Other Applicable Legislation
Source: Insects. 2011 Jul 19;2(3):343–53. doi: 10.3390/insects2030343 (PMC4553548; doi:10.3390/insects2030343)
Supplement: Supplementary File 1 [file insects-02-00343-s001.zip › supplementary-2/2010canlii76235.pdf]

Landlord and Tenant Board
Ontario

Order under Section 69
Residential Tenancies Act, 2006

File Number: TEL-08019-10

2010 CanLII 76235 (ON L.T.B.)

The Landlord applied for an order to terminate the tenancy and evict L.C. (the 'Tenant') because the Tenant did not pay the rent that the Tenant owes.

This application was heard in Toronto on November 19, 2010.

The Landlord's Agent B.D and the Tenant attended the hearing. G.C. testified on behalf of the Landlord.

Determinations:

Rent Arrears

1. The Tenant has not paid the total rent she was required to pay for the period from November 1, 2009 to November 30, 2010. Because of the arrears, the Landlord served a Notice of Termination effective September 20, 2010.

2. The monthly rent increased to $831.09 effective September 1, 2010.

3. The Landlord collected a rent deposit of $814.00 from the Tenant and this deposit is still being held by the Landlord.

4. Interest on the rent deposit is owing to the Tenant for the period from July 15, 2009 to September 20, 2010.

5. The Tenant has paid $2,442.00 to the Board in trust since the application was filed as per the terms of an interim order issued on October 21, 2010 **.

Section 82

6. At the hearing the Tenant raised a number of maintenance issues pursuant to section 82 of the *Residential Tenancies Act, 2006* (the 'Act'). Most of these issues were first brought to the current Landlord's attention in a work order submitted by the Tenant on December 7, 2009, and in a letter that was provided to the Landlord on December 5, 2009 and resubmitted on September 14, 2009.

7. Based upon the Tenant's pictures, submitted work orders and oral testimony, I find that the following items currently require repair/replacement:

   i. back door to residential complex has lacked a working locking device for a number of months compromising the Tenant's security;

ii. the kitchen tap has been leaking since the summer of 2010;

iii. Window sills in master bedroom and living room have mould growth and are in poor shape;

iv. windows lack locking devices (except for kitchen and bathroom windows);

v. no window screen in living room, the screens in kitchen and second bedroom are broken;

vi. electrical outlet for the stove is located on floor – this placement may be a fire hazard;

vii. frame around bathroom door is cracked – cannot completely close bathroom door as a result;

viii. bathroom sink not drain properly – backs-up and smells;

ix. entire rental unit should be sealed and caulked as per oral advise from Toronto Public Health to help prevent reoccurrence of bed bugs and cockroaches. (Tenant's son allergic to bed bugs as per letter from doctor).

8. At the hearing the Landlord stated that all the necessary repairs could be completed by November 22, 2010.

9. The Tenant provided insufficient evidence to establish that the kitchen countertop must be replaced. With respect to dust on the radiators, it is the Tenant who is responsible for the ordinary cleanliness of the rental unit pursuant to section 33 of the Act.

10. While the Landlord began addressing some of the needed repairs several days before the hearing, no reasonable explanation was provided as to why the Landlord waited so long to start the repairs. While the Tenant made it more difficult for the Landlord to do the repairs in November 2010 by insisting that the Landlord could only enter when she was home, this does change the fact that the Landlord did not attempt to meet its maintenance obligations for approximately 11 months.

11. The following items were addressed by the Landlord several days before the hearing:

i. bathroom ceiling patched and painted, and bathtub regrouted (earlier repairs were performed by the Landlord but there was additional leaking in April 2010 and Landlord was advised);

ii. a new lock for the front door to the residential complex was installed approximately one week before the hearing (there had been no lock since the Tenant moved-in in August 2009);

iii. bedroom door was fixed, but not painted;

2010 CanLII 76235 (ON L.T.B.)

iv. the Tenant's mailbox was recently fixed and can now be locked. (Tenant had no mail delivery for five months due to lack of lock).

12. Having considered the significant number of maintenance deficiencies, their impact upon the Tenant's ability to safely enjoy the rental unit and the Landlord's failure to start addressing many of these deficiencies for over 11 months, I find that the Tenant is entitled to a rent abatement equal to approximately 15% of the rent payable since the first work order was submitted to the Landlord in December 2009. This rent abatement, $1,371.00, shall be applied to rent arrears owing by the Tenant. The Landlord shall also be ordered to perform the need repairs.

13. I have considered all of the disclosed circumstances in accordance with subsection 83(2) of the *Residential Tenancies Act, 2006* (the 'Act'), and find that it would be unfair to grant relief from eviction pursuant to subsection 83(1) of the Act.

2010 CanLII 76235 (ON L.T.B.)

It is ordered that:

1. The tenancy between the Landlord and the Tenant is terminated. The Tenant must move out of the rental unit on or before December 4, 2010.

2. As of the date of this order, the Tenant owes no money to the Landlord because the amount of the rent deposit and interest the Landlord owes on the rent deposit plus the abatement the Landlord owes the Tenant exceeds the arrears of rent and compensation the Landlord is entitled to by $1,270.45*.

3. The Landlord is authorized to offset the following amounts from the amount the Landlord owes the Tenant:
   $26.76 per day for compensation for the use of the unit starting November 24, 2010 to the date the Tenant moves out of the unit, and
   $170.00 for the cost of filing the application.

4. The Landlord or the Tenant shall pay to the other any sum of money that is owed as a result of this order.

5. If the unit is not vacated on or before December 4, 2010, then starting December 5, 2010, the Landlord may file this order with the Court Enforcement Office (Sheriff) so that the eviction may be enforced.

6. Upon receipt of this order, the Court Enforcement Office (Sheriff) is directed to give vacant possession of the unit to the Landlord, on or after December 5, 2010.

7. If the Tenant wishes to void this order and continue the tenancy, she must pay to the Landlord or to the Board in trust $810.30 on or before December 4, 2010**. If the Tenant does not make full payment in accordance with this paragraph and by the appropriate deadline, then the Landlord may file this order with the Court Enforcement Office (Sheriff) so that the eviction may be enforced.

2010 CanLII 76235 (ON L.T.B.)

8. The Tenant may make a motion to the Board under subsection 74(11) of the Act to set aside this order if they pay the amount required under that subsection on or after December 5, 2010 but before the Sheriff gives vacant possession to the Landlord. The Tenant is only entitled to make this motion once during the period of the tenancy agreement with the Landlord.

9. The Board shall pay to the Landlord the amount of $2,442.00 together with any accrued interest. **

10. On or before December 10, 2010, the Landlord shall perform the following repairs:

    i. ensure that the back door to residential complex has a working locking device;

    ii. repair or replace leaking kitchen tap;

    iii. clean/remove mold from window sills in master bedroom and living room and ensure that window sills are in a good state of repair without excessive cracks, gouges, etc;

    iv. ensure that all windows have locking devices;

    v. ensure that all windows have a functioning window screen;

    vi. have a licensed electrician examine the electrical outlet for the stove and provide a written report with respect to whether it should be relocated for safety reasons. Relocate plug to wall if so advised by electrician;

    vii. repair/replace frame around bathroom door and ensure that bathroom door can completely close;

    viii. ensure that bathroom sink drains properly;

    ix. seal and/or caulk the entire rental unit to help prevent reoccurrence of bed bugs and cockroaches; and

    x. paint bedroom door.

11. If the above-identified repairs have not been completed by the specified deadline, the Tenant is authorized to have the outstanding repairs performed herself, and deduct all costs associated with having the repairs done from the rent owing for the months of January or February 2011 upon providing the Landlord with bills/invoices for the materials and labour.

November 23, 2010
Date Issued

Eli Fellman
Vice Chair, Landlord and Tenant Board

Toronto East-RO
2275 Midland Avenue, Unit 2
Toronto ON M1P3E7

If you have any questions about this order, call 416-645-8080 or toll free at 1-888-332-3234.

In accordance with section 81 of the Act, the part of this order relating to the eviction expires on June 5, 2011 if the order has not been filed on or before this date with the Court Enforcement Office (Sheriff) that has territorial jurisdiction where the rental unit is located.

* Refer to section A on the attached Summary of Calculations.
** Refer to section B on the attached Summary of Calculations.

2010 CanLII 76235 (ON L.T.B.)

Schedule 1
SUMMARY OF CALCULATIONS

File Number: TEL-08019-10

2010 CanLII 76235 (ON L.T.B.)

A. Amount the Tenant must pay if the tenancy is terminated:

Reasons for amount owing	Period	Amount
Arrears: (up to the termination date in the Notice of Termination)	November 1, 2009 to September 20, 2010	$1,664.17
Less the amount the Tenant paid to the Board in trust		-$2,442.00
Plus compensation: (from the day after the termination date in the Notice to the date of the order)	September 21, 2010 to November 23, 2010	$1,712.64
Less the rent deposit:		-$814.00
Less the interest owing on the rent deposit:	July 15, 2009 to September 20, 2010	-$20.26
Less amount owing to the Tenant for abatement/rebate:		-$1,371.00
Amount owing to the Landlord on the order date: (total of previous boxes)		**-$1,270.45**
Additional costs the Tenant must pay to the Landlord:		$170.00
Plus daily compensation owing for each day of occupation starting November 24, 2010:		$26.76 (per day)
**Total the Tenant must pay the Landlord if the tenancy is terminated:**		**-$1,100.45, + $26.76 per day starting November 24, 2010**

B. Amount the Tenant must pay to void the eviction order and continue the tenancy:

1. If the payment is made after November 30, 2010 but on or before December 4, 2010:

Reasons for amount owing	Period	Amount
Arrears:	November 1, 2009 to December 31, 2010	$4,453.30
Less the amount the Tenant paid to the Board:		-$2,442.00
Less amount owing to the Tenant for abatement/rebate:		-$1,371.00
Additional costs the Tenant must pay to the Landlord:		$170.00
**Total the Tenant must pay to continue the tenancy:**	On or before December 4, 2010	$810.30

2010 CanLII 76235 (ON L.T.B.)
