# Supplementary material for: Analysis of 44 Cases before the Landlord and Tenant Board Involving Bed Bug Infestations in Ontario, Canada: Focus on Adjudicator Decisions Based on Entomological/Pest Management Evidence and Accountability under the Residential Tenancy Act and Other Applicable Legislation
Source: Insects. 2011 Jul 19;2(3):343–53. doi: 10.3390/insects2030343 (PMC4553548; doi:10.3390/insects2030343)
Supplement: Supplementary File 1 [file insects-02-00343-s001.zip › supplementary-2/2010canlii79677.pdf]

Landlord and Tenant Board
Ontario

Order under Sections 30, 31
Residential Tenancies Act, 2006

File Number: TST-00961-09
TST-00745-09

2010 CanLII 79677 (ON L.T.B.)

BB (the 'Tenant') applied for an order determining that TCHC (the 'Landlord') substantially interfered with the reasonable enjoyment of the rental unit or residential complex by the Tenant and failed to meet the Landlord's maintenance obligations under the *Residential Tenancies Act, 2006* (the 'Act') or failed to comply with health, safety, housing or maintenance standards. These applications were resolved by a mediated agreement on April 23, 2010.

On July 19, 2010, the Tenant filed a request to re-open the Tenant's applications because the Landlord did not comply with the terms of their mediated settlement.

This request was heard and granted in Toronto on September 8, 2010 as set out in an interim order issued September 13, 2010.
The re-opened applications were heard in Toronto on December 1, 2010.

The Tenant and the Landlord attended the hearings.
The Landlord was represented by GS.
CF appeared as a witness for the Landlord.

All the reasons for this order appear below and no further reasons shall issue.

Facts and Determinations:

1. This matter concerns two applications. TSL-00961-09 is a T2 regarding a card tenants use to operate the laundry facilities. TSL-00745-09 is a T2 regarding visitor parking and the ‘deterioration of services’ the Tenant attributes to the Landlord taking over the building in January 2007, which, by the time of the hearing, included only an issue with the outdoor garbage area; and, a T6 regarding recurring flooding in the Tenant’s unit.

Serious interference with reasonable enjoyment

Laundry Card

2. Since July 2007, the laundry facilities in the residential complex are operated with a card that the Tenant ‘loads’ with money and then uses to operate the machines. The Tenant’s issue with the cards is that if a card is lost, the tenant loses any balance on the card, and, except for the first time, must pay a $10.00 replacement fee. The Tenant does not think this is fair in a subsidized rent environment, where $10.00 may be a substantial percentage of the monthly rent. The Tenant submits the policy seriously interferes with his reasonable enjoyment because he has no choice but to comply with the new rule. The Tenant became aware of the policy when he lost his card in or around January or February 2009 and had to replace it. The Tenant asked that the Landlord be required to

change the policy to charge only the actual cost of replacing the card, rather than charging $10.00.

3. The evidence before me is not sufficient to find that the Landlord seriously interfered with the Tenant's reasonable enjoyment of the residential complex. Even if it could be said that the card replacement policy of the third party vendor is governed by the *Residential Tenancies Act, 2006* (the 'Act'), the evidence does not support that the policy 'seriously' interfered with the Tenant's reasonable enjoyment.

2010 CanLII 79677 (ON L.T.B.)

Visitor Parking

4. Until July 2008, the tenants had access to visitor parking spots which could be used upon request. No fee was charged. Since July 2008, five spots previously used for visitor parking were reallocated. 'Non-tenant' parking is still available; however, more documentation is required to reserve a spot and spots are available at a rate of $195 per month. The Tenant submits that the change in visitor parking seriously interferes with his reasonable enjoyment because it may affect the decisions of friends or caregivers to attend at his apartment. The residential complex is downtown. The Tenant did not dispute that there is paid parking available at certain times on the street and in lots nearby and that parking spots are available to tenants who require them. The Tenant asked that free visitor parking be reinstated and for a rent abatement for the time it has not been available.

5. The evidence before me is not sufficient to find that the Landlord seriously interfered with the Tenant's reasonable enjoyment of the residential complex. Whether visitor parking is a service the removal of which should result in a reduction of rent is not the subject of this application. In the context of this application, the Tenant has not demonstrated that the change in parking 'seriously' interfered with his reasonable enjoyment.

Garbage

6. The residential complex is a large multi-storey building located in the downtown core. Items are often placed in the garbage area outside the proper bins by other tenants and non-tenants. From Monday to Friday the garbage is cleaned up first thing in the morning. Complaints about garbage are responded to quickly during the week but on the weekend, such complaints are not considered urgent and the problem is not addressed until the next business day. This seriously interferes with the Tenant's reasonable enjoyment because it creates an 'eyesore' and, in his view, creates a deterioration of living conditions. The Tenant also submitted that the garbage, which often contains pieces of furniture, may result in a bed bug infestation or could cause disease but provided no evidence in support of this. The Tenant asks that the Landlord be required to do more, for example, install cameras at the garbage site, and asked for a rent abatement.

7. In all the circumstances, I find that the evidence before is not sufficient to find that the Landlord's approach to the garbage seriously interferes with the Tenant's reasonable enjoyment of the residential complex.

Maintenance

8. The Tenant experienced multiple floods in his unit: February and July 2005; January 2006; and, April and May 2008. These incidents were all resolved in a mediated agreement of a different application and are not part of this application, except as background. There were two more floods that are the subject of this application (which was filed September 3, 2009): November 18, 2009 and June 20, 2009. The flood occurred always in the same place, in the bedroom from the ceiling in the wardrobe. The water entered in a steady drip. By the time the Tenant would notice the drip, the water would have flowed under his clothes. The Tenant would place a pail under the leak and contact the Landlord. The leak would be clamped and once it was determined that the issues had been addressed along the line, the repair would be completed and the wall closed and repaired.

9. The Tenant indicated that he was no longer pursuing an issue regarding mould in this application.

10. Pursuant to subsection 20(1) of the *Residential Tenancies Act, 2006* (the 'Act'):

> A landlord is responsible for providing and maintaining a residential complex, including the rental units in it, in a good state of repair and fit for habitation and for complying with the health, safety, housing and maintenance standards.

11. Subsection 20(1) of the Act requires a landlord to maintain a rental unit in a good state of repair. Clearly, while the pipes were leaking the unit cannot be said to have been in a good state of repair. In the result, I am satisfied that the Landlord failed to meet the Landlord's obligations under subsection 20(1) of the Act regarding the flooding.

Remedy

12. The Tenant asked for a 50% rent abatement from one year before the application was filed to June 2010 when the problem was fixed.

13. In accordance with the Act and Interpretation Guidelines, if the Landlord responded within a reasonable time and the response was appropriate to affect the repair, no abatement or other remedy should be ordered.

14. The Tenant agreed that the Landlord always responded and stopped the leak within two days. The Landlord acknowledged there were problems with the lines, not just with the Tenant's unit and it would be several days before the repair could be completed and the wall repaired. The pipes in the entire building were replaced in June 2010. Tenant agrees that as of the hearing the problem appears to have been remedied.

15. In all these circumstances, I find that while the Landlord's response was timely, the pattern of leaks is indicative that the repairs were not effective. The Landlord indicated that it took time to identify the problem and acquire the funding to effect the required repairs. In the meantime however, the Tenant experienced several more floods. Having regard to the nature of the problems and the impact statement of the Tenant, I find the

2010 CanLII 79677 (ON L.T.B.)

Tenant is entitled to a lump sum rent abatement for the days the flood recurred. The evidence before me is that each incident was remedied within two days. The Tenant's monthly rent is $85.00. In all the circumstances, I find the Tenant is entitled to a lump sum rent abatement of $100.00. The Tenant is also entitled to his application fee.

2010 CanLII 79677 (ON L.T.B.)

16. The Tenant also asked for $2,000.00 for mental distress due to living in what he described as a 'substandard' apartment. While the Tenant clearly found it stressful living in the unit no knowing when the flood may recur, I find that the remedy requested is too remote considering the circumstances described.

17. The Tenant also asked for $170.00 for costs he incurred having the cable outlet replaced. The evidence before me is that the Tenant did not notify the Landlord until after the expense was incurred, by not filling out a request form. The Landlord was therefore limited in its ability to mitigate the expense. In these circumstances, I find the Tenant is entitled to compensation of $85.00, representing one half of the cost incurred.

18. The total amount the Landlord owes the Tenant is $230.00. The Tenant received $1,500.00 from the Landlord in the mediated agreement that was declared of no force or effect when these matters were reopened. I confirm that the Tenant must return this amount to the Landlord, minus the amount the Landlord owes the Tenant as a result of this order.

19. The Tenant asked and the Landlord agreed that, if funds were owed to the Landlord, that he be given sufficient time to pay it back. I agree.

It is ordered that:

1. The Landlord shall pay to the Tenants a lump sum rent abatement of $100.00 and out of pocket expenses of $85.00.

2. The Landlord shall also pay the Tenant $45.00 for the cost of filing the application.

3. The total amount the Landlord owes the Tenants is $230.00.

4. This amount shall be subtracted from the $1,500.00 the Tenant must return to the Landlord as a result of reopening this matter.

5. In the result, the Tenant must pay to the Landlord $1,270.00 ($1,500.00 - $230.00).

6. The Tenant shall pay the amount set out in paragraph at the rate of $10.00 per month on or before the 15th of each month until the amount is paid in full. If the Tenant fails to make any of the payments in full or on time, the entire balance remaining will become immediately due and owing.

December 31, 2010
Date Issued

Louise Horton
Member, Landlord and Tenant Board

Toronto South-RO
79 St. Clair Avenue East, Suite 212, 2nd Floor
Toronto ON M4T1M6

If you have any questions about this order, call 416-645-8080 or toll free at 1-888-332-3234.

2010 CanLII 79677 (ON L.T.B.)
