# Supplementary material for: Analysis of 44 Cases before the Landlord and Tenant Board Involving Bed Bug Infestations in Ontario, Canada: Focus on Adjudicator Decisions Based on Entomological/Pest Management Evidence and Accountability under the Residential Tenancy Act and Other Applicable Legislation
Source: Insects. 2011 Jul 19;2(3):343–53. doi: 10.3390/insects2030343 (PMC4553548; doi:10.3390/insects2030343)
Supplement: Supplementary File 1 [file insects-02-00343-s001.zip › supplementary-2/2010canlii93933.pdf]

Landlord and Tenant Board

Order under Section 69
Residential Tenancies Act, 2006

File Number: TNL-09093-10

2010 CanLII 93933 (ON LTB)

(OL) (the 'Landlord') applied for an order to terminate the tenancy and evict (SM) and (MS) (the 'Tenants') because the Tenants did not pay the rent that the Tenants owe.

This application was heard in Toronto on March 21, 2010.

The Landlord’s representative, (RH), and one of the Tenants, MS, attended the hearing. Also in attendance as a witness for the Landlord was (NG) an employee of the Landlord.

At the hearing the Tenants raised the following issues pursuant to section 82 of the *Residential Tenancies Act, 2006* (the 'Act'): breach of the Landlord’s maintenance obligations.

Determinations:

1. The Tenants have paid $855.00 to the Board in trust since the application was filed.

2. The Tenants vacated the rental unit on October 31, 2010.

3. Taking this payment in trust into account, the Tenants have not paid $2,137.50 of the total rent they were required to pay for the period from July 1, 2010 to October 31, 2010. Because of the arrears, the Landlord served a Notice of Termination. The Tenants have also not paid the $170.00 cost of filing the Landlord’s application.

4. The Landlord collected a rent deposit of $855.00 from the Tenants and this deposit is still being held by the Landlord.

5. Interest on the rent deposit is owing to the Tenants from April 1, 2010.

6. There was a bedbug infestation in the rental unit. The Tenants moved into the unit on April 1, 2010, and within two weeks discovered the presence of bedbugs. The Landlord sprayed the unit by April 27, 2010, fairly immediately after the Landlord was informed of the problem. The problem persisted, and the Landlord returned three weeks later to spray again. The problem still persisted, and the Tenants’ furniture became infested as well.

7. In the meantime, the Tenant purchased cans of spray himself and attempted to spray the unit himself, without success.

8. The Tenant returned to the office in May and then in July of 2010 to speak to the manager about the bedbug problem, but there was no follow up until July 13, 2010. This spraying was also not successful. The Landlord returned in late August, around August 20, 2010 to spray again, again without eradicating the bedbugs.

2010 CanLII 93933 (ON LTB)

9. Ultimately, SM, who was recovering from surgery and was vulnerable to infection from bites, moved out in August 1, 2010 (but did not vacate the rental unit yet) and rented a room from a friend for about two months, paying $470.00 a month. The Tenant brought in a Medical Doctor's note regarding his treatment for a bug bite. Because the Doctor did not attend the hearing to testify, I can only give this note limited weight, but I will not discount it either. Also, it should be acknowledged that the Tenant SM brought in photos of bug bites on his body.

10. The Tenants gave back vacant possession to the Landlord on October 31, 2010. Almost all of their furniture was left behind with the exception of a glass table, a disassembled desk and some other minor items.

11. SM submitted that he left behind some $5,885.00 in new furniture and clothing and bedding. He brought photos of much of these items, but was not able to bring receipts. I recognize that it would be difficult in most circumstances for anyone to be able to produce furniture receipts after this length of time. In the photos, the furniture does look new and of good quality.

12. SM also submitted that the Landlord knowingly rented him the unit when it was already infested with bedbugs. NF, an employee of the Landlord, attended the hearing and testified that the Landlord always sprays units before new Tenants move in. I am not persuaded that there was any intent on the part of the Landlord to rent the Tenants an infested unit.

13. However, the problem remains that the Landlord did not spray the unit between May of 2010 and July 13, of 2010, although I am persuaded that it was reasonably informed of this ongoing issue.

14. I find that, overall, the Tenant is entitled to an abatement of roughly 25% of his total claim of $5,885.00. This abatement works out to $1,442.00, and recognizes the fact that the Tenant did suffer significant loss of property, and also recognizes the fact that there was a significant gap in time when the Landlord should have at least continued to try to rid the unit of the bedbugs.

15. I cannot award the entire amount requested by the Tenant because it is too difficult to substantiate his entire claim for the lost items, and it is also too difficult to substantiate that all of the items were entirely unsalvageable and therefore could not be taken away by the Tenant.

16. Also, the Landlord cannot in all fairness be held entirely liable for the bedbug problem as it did make some effort to address this very difficult issue.

17. However, I believe the Tenants inasmuch as the unit became essentially uninhabitable as of August 1, 2010, which is when SM moved in with a friend. (The other Tenant had vacated the unit earlier for other reasons unrelated to this claim).

18. Had the Tenant actually vacated the rental unit as of the date that he believed it was no longer inhabitable, both parties would have been able to mitigate their losses from an

earlier date. However, there is no *obligation* on the part of a Tenant to vacate a unit due to maintenance obligations, and therefore the concept of mitigation cannot be applied to the Tenants in this situation.

19. Ultimately, this abatement has the effect of, after the amount in the trust account is paid out to the Landlord and the last month's rent deposit and interest are applied, leaving the parties with no further liability to each other. The table of calculations appended to this order will show that there is essentially nothing owed by the Tenant to the Landlord at this point and vice versa.

2010 CanLII 93933 (ON LTB)

It is ordered that:

1. The abatement of rent offsets any arrears of rent and application fee that were owed to the date the rental unit was vacated, which was October 31, 2010.

2. The Board shall pay to the Landlord the amount of $855.00 together with any accrued interest.

May 4, 2010
Date Issued

Joseph Berkovits
Member, Landlord and Tenant Board

Toronto North-RO
47 Sheppard Avenue East, Suite 700, 7th Floor
Toronto ON M2N5X5

If you have any questions about this order, call 416-645-8080 or toll free at 1-888-332-3234.

* Refer to section A on the attached Summary of Calculations.

Schedule 1
SUMMARY OF CALCULATIONS

File Number: TNL-09093-10

2010 CanLII 93933 (ON LTB)

A. Amount the Tenants must pay:

**Reasons for amount owing**	**Period**	**Amount**
Arrears:	July 1, 2010 to October 31, 2010	$2,992.50
Less the amount the Tenants paid to the Board in trust		-$855.00
Less the rent deposit:		-$855.00
Less the interest owing on the rent deposit:	From April 1, 2010	-$10.53
Less amount owing to the Tenant for abatement/rebate:		-$1,442.00
Amount owing to the Landlord: (total of previous boxes)		**-$170.03**
Additional costs the Tenants must pay to the Landlord:		$170.00
**Total the Tenants must pay the Landlord:**		**-$0.03**
