# Supplementary material for: Analysis of 44 Cases before the Landlord and Tenant Board Involving Bed Bug Infestations in Ontario, Canada: Focus on Adjudicator Decisions Based on Entomological/Pest Management Evidence and Accountability under the Residential Tenancy Act and Other Applicable Legislation
Source: Insects. 2011 Jul 19;2(3):343–53. doi: 10.3390/insects2030343 (PMC4553548; doi:10.3390/insects2030343)
Supplement: Supplementary File 1 [file insects-02-00343-s001.zip › supplementary-2/2011canlii13390.pdf]

Landlord and Tenant Board

Order under Section 30
Residential Tenancies Act, 2006

File Number: SOT-11191-10

2011 CanLII 13390 (ON LTB)

N.A. and L.S. (the 'Tenants') applied for an order determining that the Landlord failed to meet the Landlord's maintenance obligations under the *Residential Tenancies Act, 2006* (the 'Act') or failed to comply with health, safety, housing or maintenance standards.

This application was heard in Hamilton on January 25, 2011.

The Tenant N.A. and M.A., the Landlord's representative, attended the hearing. A.A.B. attended the hearing to assist and interpret for the Tenant.

Determinations:

1. The unit was infested with bedbugs. Therefore, I find that the Landlord failed to meet the Landlord's obligations under subsection 20(1) of the Act to maintain the rental unit.

2. I am not satisfied that the Tenant's furniture was damaged or needed to be disposed of as a result of the Landlord's failure to maintain the rental unit.

3. I am satisfied that the Tenants are entitled to costs of $380.00 for a hotel room on October 12, 2010.

It is ordered that:

1. The Landlord shall pay to the Tenants $380.00 which is the reasonable out-of-pocket expenses the Tenants have incurred for a hotel room on October 12, 2010. These expenses were incurred because of the Landlord's failure to maintain the rental unit.

2. The Landlord shall pay to the Tenants $45.00 which is the cost of filing this application.

3. The total amount the Landlord owes the Tenants is $425.00.

4. The Landlord shall pay the Tenants the full amount owing by February 28, 2011.

5. If the Landlord does not pay the Tenants the full amount owing by February 28, 2011 the Landlord will owe interest. This will be simple interest calculated from March 1, 2011 at 3.00% annually on the outstanding balance.

6. If the Landlord does not pay the Tenants the full amount owing by February 28, 2011, the Tenants may recover this amount by deducting $425.00 from the rent for March 2011.

7. The Tenants have the right, at any time, to collect the full amount owing or any balance outstanding under this order.

February 4, 2011
Date Issued

Petar Guzina
Member, Landlord and Tenant Board

Southern-RO
119 King Street West, 6th Floor
Hamilton ON L8P4Y7

If you have any questions about this order, call 416-645-8080 or toll free at 1-888-332-3234.

2011 CanLII 13390 (ON LTB)

File Number: SOT-11191-10

REASONS

2011 CanLII 13390 (ON LTB)

Reasons to Order SOT-11191-10 issued on February 4, 2011 by Petar Guzina.

1. The Tenants T6 application alleges that the Landlord has failed to maintain the rental unit because it is infested with cockroaches and bedbugs. The Tenants claim $7000.00 for the replacement of all furniture, mattresses, sheets, pillows, clothes, carpets etc. The Tenants also claim $200.00 for special cleaners used to try and get rid of the insects.

2. The Tenants stated that their children were itchy for 2 to 3 months before they took them to a doctor. The Tenants say that they went to the doctor about 5 or 6 months ago. The Tenants submitted two notes from a doctor dated December 31. 2010. Both notes confirm that the family is being treated for bed bug bites. Accordingly, I am satisfied that the unit was infested with bedbugs. Therefore, I find that the Landlord failed to meet the Landlord's obligations under subsection 20(1) of the Act to maintain the rental unit.

3. The Tenants claim that they have been complaining to the Landlord for nearly a year. They submitted no letters of complaint to the Landlord. The Tenants want to be compensated for all of their furniture, bedding, clothing and carpeting because they want to move and do not want to take these items with them. They are not certain, but believe that there may be bedbug or cockroach eggs in the furniture and carpet. They submitted no independent evidence of the furniture being untreatable and in need of disposal. The Tenants are making an assumption, which is not enough to prove their case on a balance of probabilities. Accordingly, I am not satisfied that the Tenant's furniture was damaged or needed to be disposed of as a result of the Landlord's failure to maintain the rental unit.

4. The Tenants submitted no evidence with respect to any specialty cleaners used by them.

5. The Landlord's representative, who is also the building property manager, stated that the Tenants did not complain to him until August 2010. He inspected the unit but has never seen any evidence of cockroaches or bedbugs. At the Tenants' request he arranged for treatment on September 28, 2010. The treatment did not take place because the Tenants did not prepare the apartment as required by the pest control company. The treatment was rescheduled and conducted on October 12, 2010. A second round of treatment was to take place on November 12, 2010. On that day the Tenants refused treatment because the children were ill. The treatment was rescheduled and conducted on December 3, 2010. The Landlord also had the unit below the Tenants treated.

6. At the hearing the Tenants requested compensation for a hotel room on October 12, 2010. This cost claim is added as it is directly related to the Landlord's failure to maintain the unit free of pests. The female Tenant is pregnant and the pest control company recommended pregnant women stay out of treated units for a period of 24 hours. The Landlord did not dispute the reasonableness of the Tenants out of pocket expense. Accordingly, I am satisfied that the Tenants are entitled to costs of $380.00 for a hotel room on October 12, 2010.

February 4, 2011
Date Issued

Petar Guzina
Member, Landlord and Tenant Board

Southern-RO
119 King Street West, 6th Floor
Hamilton ON L8P4Y7

2011 CanLII 13390 (ON LTB)
