# Supplementary material for: Analysis of 44 Cases before the Landlord and Tenant Board Involving Bed Bug Infestations in Ontario, Canada: Focus on Adjudicator Decisions Based on Entomological/Pest Management Evidence and Accountability under the Residential Tenancy Act and Other Applicable Legislation
Source: Insects. 2011 Jul 19;2(3):343–53. doi: 10.3390/insects2030343 (PMC4553548; doi:10.3390/insects2030343)
Supplement: Supplementary File 1 [file insects-02-00343-s001.zip › supplementary-2/2011canlii13393.pdf]

Order under Section 69
**Residential Tenancies Act, 2006**

**File Number:** SOL-11453-10

2011 CanLII 13393 (ON LTB)

The Landlord applied for an order to terminate the tenancy and evict A.D. and B.H. (the 'Tenants') because the Tenants did not pay the rent that the Tenants owe.

This application was heard in Hamilton on January 26, 2011.

The Landlord’s representative and the Tenants attended the hearing.

At the hearing the Tenants raised the following issues pursuant to section 82 of the *Residential Tenancies Act, 2006* (the 'Act'):

Illegal entry by the Landlord,
Mice, bedbug and cockroach infestation,
Refrigerator requires replacement or repair.

**Determinations:**

1. I find that the agent of the Landlord or the Landlord’s agent illegally entered the rental unit.

2. I find the Tenants are entitled to $100.00 for the illegal entry.

3. I find the Tenants did not prove on a balance of probabilities that the Landlord harassed them.

4. The Tenants have not paid the total rent they were required to pay for the period from November 1, 2010 to January 31, 2011. Because of the arrears, the Landlord served a Notice of Termination effective December 20, 2010.

5. I find the Tenants have not proven on a balance of probabilities that the Landlord is responsible for the loss of the couch.

6. The Landlord collected a rent deposit of $250.00 from the Tenants and this deposit is still being held by the Landlord.

7. Interest on the rent deposit is owing to the Tenants for the period from October 10, 2010 to December 20, 2010.

8. The Tenants lost food valued at $60.00 due to a refrigerator that did not work properly. I find the Landlord is responsible for this loss.

9. The Landlord did not meet their obligation under Section 20 of the Act to provide and maintain a residential complex, including the unit in it, in a good state of repair and fit for habitation and for complying with health, safety, housing, and maintenance standards. There were mice and bug infestations in the building.

10. I am satisfied that the Landlord reacted expeditiously when the Tenants brought the issue to the Landlord's attention. I am required to follow what is set out in section 8 of Ontario Regulation 516/06. Having determined that the Landlord's actions were prompt, reasonable and professional, I do not find that the Landlord was in contravention of the Regulation.

11. I have considered all of the disclosed circumstances in accordance with subsection 83(2) of the *Residential Tenancies Act, 2006* (the 'Act'), and find that it would be unfair to grant relief from eviction pursuant to subsection 83(1) of the Act.

2011 CanLII 13393 (ON LTB)

**It is ordered that:**

1. The Landlord shall pay the Tenants $100.00 for the illegal entry.

2. The Landlord shall pay the Tenants $60.00 for the loss of food.

3. The tenancy between the Landlord and the Tenants is terminated. The Tenants must move out of the rental unit on or before February 20, 2011.

4. The Tenants shall pay to the Landlord $3,076.42*, which represents the amount of rent owing and compensation up to February 9, 2011, less the rent deposit and interest the Landlord owes on the rent deposit and less the amount of the compensation and abatement the Landlord owes the Tenants.

5. The Tenants shall also pay to the Landlord $32.71 per day for compensation for the use of the unit starting February 10, 2011 to the date they move out of the unit.

6. The Tenants shall also pay to the Landlord $170.00 for the cost of filing the application.

7. If the Tenants do not pay the Landlord the full amount owing* on or before February 20, 2011, the Tenants will start to owe interest. This will be simple interest calculated from February 21, 2011 at 3.00% annually on the balance outstanding.

8. If the unit is not vacated on or before February 20, 2011, then starting February 21, 2011, the Landlord may file this order with the Court Enforcement Office (Sheriff) so that the eviction may be enforced.

9. Upon receipt of this order, the Court Enforcement Office (Sheriff) is directed to give vacant possession of the unit to the Landlord, on or after February 21, 2011.

10. If, on or before February 20, 2011, the Tenants pay the amount of $3,990.00** to the Landlord or to the Board in trust, this order for eviction will be void. This means that

the tenancy would not be terminated and the Tenants could remain in the unit. If this payment is not made in full and on time, the Landlord may file this order with the Court Enforcement Office (Sheriff) so that the eviction may be enforced.

11. The Tenants may make a motion to the Board under subsection 74(11) of the Act to set aside this order if they pay the amount required under that subsection on or after February 21, 2011 but before the Sheriff gives vacant possession to the Landlord. The Tenants are only entitled to make this motion once during the period of the tenancy agreement with the Landlord.

2011 CanLII 13393 (ON LTB)

**February 9, 2011**
**Date Issued**

_______________________
Shirley Collins
Member, Landlord and Tenant Board

Southern-RO
119 King Street West, 6th Floor
Hamilton ON L8P4Y7

If you have any questions about this order, call 416-645-8080 or toll free at 1-888-332-3234.

In accordance with section 81 of the Act, the part of this order relating to the eviction expires on August 21, 2011 if the order has not been filed on or before this date with the Court Enforcement Office (Sheriff) that has territorial jurisdiction where the rental unit is located.

* Refer to section A on the attached Summary of Calculations.
** Refer to section B on the attached Summary of Calculations.

**Schedule 1**
**SUMMARY OF CALCULATIONS**

**File Number: SOL-11453-10**

2011 CanLII 13393 (ON LTB)

## A. Amount the Tenants must pay if the tenancy is terminated:

| Reasons for amount owing | Period | Amount |
|---|---|---|
| Arrears: (up to the termination date in the Notice of Termination) | November 1, 2010 to December 20, 2010 | $1,649.25 |
| Plus compensation: (from the day after the termination date in the Notice to the date of the order) | December 21, 2010 to February 9, 2011 | $1,668.21 |
| Less the rent deposit: | | -$250.00 |
| Less the interest owing on the rent deposit: | October 10, 2010 to December 20, 2010 | -$1.04 |
| Less amount owing to the Tenant for abatement/rebate: | | $160.00 |
| Amount owing to the Landlord on the order date: (total of previous boxes) | | **$3,066.42** |
| Additional costs the Tenants must pay to the Landlord: | | $170.00 |
| Plus daily compensation owing for each day of occupation starting February 10, 2011: | | $32.71 (per day) |
| **Total the Tenants must pay the Landlord if the tenancy is terminated:** | | **$3,076.42, + $32.71 per day starting February 10, 2011** |

## B. Amount the Tenants must pay to void the eviction order and continue the tenancy:

| Reasons for amount owing | Period | Amount |
|---|---|---|
| Arrears: | November 1, 2010 to February 28, 2011 | $3,980.00 |
| Less amount owing to the Tenant for abatement/rebate: | | $160.00 |
| Additional costs the Tenants must pay to the Landlord: | | $170.00 |
| **Total the Tenants must pay to continue the tenancy:** | On or before February 20, 2011 | $3,990.00 |

**File Number:** SOL-11453-10

REASONS

2011 CanLII 13393 (ON LTB)

Reasons to Order SOL-11453-10 issued on February 9, 2011 by Shirley Collins.

**Arrears of Rent**

The Landlord's representative testified that the Tenants moved into the rental unit in October 2010. They paid one half of the rent for that month and the last month's rent deposit. They have not paid rent since. The Tenants did not dispute the testimony once they perused the written tenancy agreement however they claim there are numerous issues for which they are claiming an abatement of rent.

There is nothing in the Residential Tenancies Act, 2006 (the 'Act") which states a tenant can withhold rent for tenant issues. I find the Tenants are in arrears of rent and are responsible for the $170.00 application filing fee paid by the Landlord.

**Section 82 issues**

*Preliminary Issue*

The Landlord's representative was not aware of the issues being raised by the Tenant pursuant to section 82 but chose to proceed with the hearing.

*Illegal Entry*

During the tenancy, the Landlord sent maintenance personnel to fix the cupboard doors in the kitchen of the rental unit. The persons said they would need to return with a part to fix it but didn't return that day. The next morning they knocked on the door. The Tenants were in bed and didn't want to answer their door. The workers entered the rental unit and fixed the cupboard.

The Landlord's representative said the repairs were needed in the unit and the workers needed to return to finish the work. The worker knocked and entered.

Section 27 of the Act states, in part:

> (1) A landlord may enter a rental unit in accordance with written notice given to the tenant at least 24 hours before the time of entry….

Written notice of entry is required. This was not an emergency situation. No written notice was given to enter on the day that the workers returned with the part for the cupboard. I find that the agent of the Landlord illegally entered the rental unit.

*Harassment by Landlord's Representative*

The female Tenant requested an apology from the Landlord because the Landlord's representative told a neighbour the Tenants' relationship had broken up. The Landlord's representative testified she didn't make the comments and submitted it was only hearsay evidence.

I prefer the direct evidence of the Landlord's representative over that of the hearsay evidence of the female Tenant. I find the Tenants did not prove on a balance of probabilities that the Landlord harassed them.

*Loss of the Tenants' Couch*

The female Tenant had two weeks left in her pregnancy at the time the Tenants moved into the rental unit. Their love seat was moved into their unit but their sofa bed couch was moved into a storage room because the service elevator was not available. No one in the building was available to assist them. The female Tenant was in the hospital off and on for a week until she had her baby. The Tenant returned from the hospital. The couch was gone. The Tenants were told by the Landlord that it was their responsibility. The female Tenant said that she did not know the value of the couch as it was a gift from her mother but claims a minimum of $400.00 as it was brand new.

The Landlord's representative testified that the loading dock is on first floor. All tenants moving in and out use that loading dock. She said she was told the Tenants' couch wouldn't fit in the rental unit. The doors of the loading dock are wide open during the day and closed and locked at night. She said the Tenants were told that the Landlord could not be responsible for their couch.

I find the testimony of the Landlord's representative that the loading dock area where the Tenants left their couch is not a secure area credible. The Tenants did not provide details of the efforts they made to move their couch. They seemed unaware of the length of time it was sitting in the area. The female Tenant was in and out of the hospital during the days following their move into the building. Likely, the Tenants had other more pressing problems at that time with the pregnancy and did not follow up on moving the couch from the loading dock area. I find that the Tenants have not proven on a balance of probabilities that the Landlord is responsible for the loss of the couch.

*Infestations*

The first day the Tenants moved in they heard that there were pest problems in the rental complex. The Tenants saw three dead cockroaches. The Landlord told them there were no problems and the Tenants must have brought the cockroaches into the building.

In November 2010 the male Tenant and the two week old baby were bitten by bedbugs. The Tenants testified that they took the plastic off the bed when they moved into the unit so they know they didn't bring the bedbugs into the rental unit. The Landlord told them to vacuum their bed.

The Tenants saw cockroaches in the hall of the rental complex. The Landlord's representative told them that she would put gel in the cupboards and spray. The Tenants received notice that there would be pest control treatment on December 19, 2010. The Tenants left the rental unit with

2011 CanLII 13393 (ON LTB)

their children. When they returned, their unit had not been sprayed; only gel was put throughout the cupboards and rental unit.

The Tenants found two dead mice under their love seat couch in the living room. The Tenants were told that there was poison in the ceilings of the hallways and that the mice eat it and then die. However they think they hear baby mice in their couch. They Landlord told them to vacuum the couch.

The Landlord’s representative testified the female Tenant didn’t complain about mice, only that she found a couple of dead mice. The Landlord puts poison in all common areas and the garbage room to control mice in the building. They also supply traps to tenants who ask.

The Landlord’s representative testified there was no cockroach infestation in the rental unit. The former tenants of the unit still live in the building and they have never had an infestation.

She said the Tenants were told how to prepare the unit for pest control spraying. The Tenants did not follow the instructions to prepare the rental unit and therefore gel was used instead which is safer to use under those circumstances.

*Refrigerator*

The Tenants testified their fridge turned into a freezer a couple of weeks prior to the date of the hearing. The Tenants were at the hospital and when they returned home the $60.00 meat order for two adults and one child they had bought and frozen was partly thawed. They had to throw out the meat. They said they told the Landlord. The Landlord’s representative testified she had no knowledge of the problem.

I find the Tenants testimony about the problem with their refrigerator to be credible in terms of losing $60.00 worth of meat. Their testimony had the ring of truth about it and they claimed an amount of money that does not seem excessive. The evidence was that the loss of the meat was within a couple of weeks of the hearing. I find the Landlord is responsible for the loss of the meat.

Section 20 of the Act states:

(1) A landlord is responsible for providing and maintaining a residential complex, including the rental units in it, in a good state of repair and fit for habitation and for complying with health, safety, housing and maintenance standards.

(2) Subsection (1) applies even if the tenant was aware of a state of non-repair or a contravention of a standard before entering into the tenancy agreement.

There were mice and bug infestations in the building hence the treatment arranged by the Landlord. However, the evidence is that the Landlord responded by arranging for treatment.

2011 CanLII 13393 (ON LTB)

Having made these determinations, the Landlord did not meet their obligation under Section 20 of the Act to provide and maintain a residential complex, including the unit in it, in a good state of repair and fit for habitation and for complying with health, safety, housing, and maintenance standards.

However, I have considered whether there should be an abatement of rent.

The issue is whether or not the Landlord acted in a reasonable manner to eradicate the infestations. I find the Tenants did not properly prepare their unit for the treatment application. The Landlord can only respond to the infestations if the Tenants co-operate and follow the instructions of the pest control professionals.

I am satisfied the Landlord’s treatment efforts were reasonable. I am also satisfied that the Landlord reacted expeditiously when the Tenants brought the issue to the Landlord’s attention. I am required to follow what is set out in section 8 of Ontario Regulation 516/06. Having determined that the Landlord’s actions were prompt, reasonable and professional, I do not find that the Landlord was in contravention of the Regulation.

Taking into consideration the illegal entry and the impact on the Tenant, I find the Tenant is entitled to a rebate of rent in the amount $100.00 plus $60.00 for the loss of food from the broken refrigerator.

2011 CanLII 13393 (ON LTB)

**February 9, 2011**
**Date Issued**

Shirley Collins
Member, Landlord and Tenant Board

Southern-RO
119 King Street West, 6th Floor
Hamilton ON L8P4Y7
