# Supplementary material for: Analysis of 44 Cases before the Landlord and Tenant Board Involving Bed Bug Infestations in Ontario, Canada: Focus on Adjudicator Decisions Based on Entomological/Pest Management Evidence and Accountability under the Residential Tenancy Act and Other Applicable Legislation
Source: Insects. 2011 Jul 19;2(3):343–53. doi: 10.3390/insects2030343 (PMC4553548; doi:10.3390/insects2030343)
Supplement: Supplementary File 1 [file insects-02-00343-s001.zip › supplementary-2/2011canlii13413.pdf]

Landlord and Tenant Board

Ontario

Order under Section 30
Residential Tenancies Act, 2006

File Number: TST-11344-10

2011 CanLII 13413 (ON LTB)

MB and MDS (the 'Tenants') applied for an order determining that EH (the 'Landlord') failed to meet the Landlord's maintenance obligations under the *Residential Tenancies Act, 2006* (the 'Act') or failed to comply with health, safety, housing or maintenance standards (T6 Application).

The Tenants also applied for an order determining that the Landlord has collected or retained money illegally (T1 Application).

These applications were heard in Toronto on January 26, 2011 together with the Landlord's application TSL-11345-10. An order with respect to TSL-11345-10 is issued simultaneously with this order.

The Tenants and the Landlord attended the hearing. The Landlord was represented by MZ.

The Tenants testified under oath.

Witnesses for the Tenants:
LOS, a friend of the Tenants
LS, mother of the Tenant, MDS

Witnesses for the Landlord:
RK, property manager of the residential complex
AC, superintendent of the residential complex

The Tenant MDS is referred to in this order as MS

This order contains my reasons, no further reasons will issue.

Determinations:

T1 Application:

1. The Tenants' T1 application must be dismissed, as there was no evidence presented to establish that the Landlord charged illegal rent.

T6 Application:

2. The Tenant's maintenance application alleges that the Landlords failed to comply with subsection 20(1) of the *Residential Tenancies Act, 2006* (the 'Act'), because they failed to adequately address an infestation of bedbugs in the rental unit.

3. It is not disputed that the Tenants had bed bugs in their rental unit.

4. Pursuant to 20(1) of the Residential Tenancies Act, 2006 (the ‘Act’):

   > *A landlord is responsible for providing and maintaining a residential complex, including the rental units in it,* ***in a good state of repair*** *and fit for habitation and for complying with* ***health****, safety, housing and maintenance standards.* [Emphasis added]

5. Subsection 20(1) of the Act requires a landlord to maintain a rental unit in a good state of repair. Clearly, while there were bed bugs in the rental unit, the rental unit could not have been in a good state of repair, and could not have complied with health standards. As a result, I am satisfied that the Landlord failed to meet the Landlord's obligations under subsection 20(1) of the Act.

6. The question is whether the Landlord took reasonable and timely steps to exterminate the bed bugs after the Landlord was informed of the presence of the bedbugs in the Tenants’ rental unit. The issue to be determined is, when did the Landlord find out about the presence of the bed bugs in the rental unit.

7. The Tenants submitted that the Landlord ought to have known that there were bed bugs in the rental unit when they moved into the rental unit in September 2009, because according to an online bed bug registry the residential complex was identified as a building with bed bug problems.

8. The Landlord acknowledged that there have been isolated bed bug issues in the residential complex previously, but not in the Tenants’ rental unit. The Landlord had no knowledge of any such problems in the Tenants rental unit at the time the Tenants moved in.

9. Furthermore, the property manager testified that the rental unit had been cleaned and prepared before the Tenants moved into the rental unit and the previous tenant did not complain of bed bugs.

10. The Tenants testified that they have been bitten from the day they moved into the rental unit in September 2009. The Tenants did not know that they had a bed bug infestation until they sought medical attention in January 2010.

11. The Tenants testified that on February 10, 2010 they informed the superintendent that there were bed bugs in their rental unit. The Tenants called a witness who was present in the rental unit on February 10, 2010, when the superintendent attended at the door of the rental unit. The witness stated that he heard bits and pieces of the conversation between the Tenant MS and the superintendent. The witness was engaged in a conversation with the other Tenant and did not hear exactly what had been said between the superintendent and the other Tenant, MS.

12. MS testified that he had also told the superintendent about the bedbugs in the summer of 2010.

2011 CanLII 13413 (ON LTB)

2011 CanLII 13413 (ON LTB)

13. The superintendent denied having been told of the existence of the bed bugs in February 2010 or in the summer of 2010.

14. The Tenants took no further action to inform the Landlord. They have not complained about the bed bugs in writing, nor did they contact the property manager.

15. It was undisputed evidence that the Tenant MS happened to meet the property manager in front of the residential complex sometime in September 2010 and informed him of the bed bugs in the unit. The property manager attended at the rental unit at once and a pest control company subsequently attended at the rental unit on September 29, 2010. The unit was treated two more times on or about October 20, 2010 and October 29, 2010.

16. It is the Tenant's burden of proof to establish that they informed the Landlord of the existence of the bed bugs. I am not satisfied based on the evidence presented, that the Tenants established that they informed the Landlord of the bed bugs on February 10, 2010. The testimony of the witnesses and the Tenants' pattern of conduct do not support the Tenants' position that they informed the Landlord on February 10, 2010 or that the Landlord ought to have known that there were bed bugs in the rental unit.

17. After the pest control company treated the rental unit three times, the Tenants, who had an infant child and were concerned about the possibly harmful effects of the treatment, refused further treatment. The Tenants also refused to have their unit inspected by a trained bed bug detection dog on November 30, 2010.

18. The Tenants submitted that there were still bed bugs present in their rental unit at the time of the hearing.

19. In conclusion, based on the evidence presented at the hearing I find that after the Landlord was informed about the presence of the bed bugs in the Tenants' rental unit, the Landlord took reasonable and timely steps to eliminate the problem. Any further treatment was thwarted by the Tenants' actions. As such, it would not be appropriate to hold the Landlord financially liable in the circumstances.

20. The only remedy the Tenants requested was a monetary award, including compensation for property that may or may not have been infested by the bed bugs. The Tenants did not request that the Landlord eradicate the bed bugs. As a monetary award was found inappropriate in the circumstances, no remedy to the Tenants will issue.

It is ordered that:

1. The Tenants' application is dismissed.

February 14, 2011
Date Issued

____
Jana Rozehnal
Member, Landlord and Tenant Board

Toronto South-RO
79 St. Clair Avenue East, Suite 212, 2nd Floor
Toronto ON M4T1M6

If you have any questions about this order, call 416-645-8080 or toll free at 1-888-332-3234.

2011 CanLII 13413 (ON LTB)
