# Supplementary material for: Analysis of 44 Cases before the Landlord and Tenant Board Involving Bed Bug Infestations in Ontario, Canada: Focus on Adjudicator Decisions Based on Entomological/Pest Management Evidence and Accountability under the Residential Tenancy Act and Other Applicable Legislation
Source: Insects. 2011 Jul 19;2(3):343–53. doi: 10.3390/insects2030343 (PMC4553548; doi:10.3390/insects2030343)
Supplement: Supplementary File 1 [file insects-02-00343-s001.zip › supplementary-2/2011canlii13438.pdf]

Landlord and Tenant Board

Ontario

Order under Section 69
Residential Tenancies Act, 2006

File Number: SOL-10799-10

2011 CanLII 13438 (ON LTB)

The Landlord applied for an order to terminate the tenancy and evict D.C. and S.R. (the 'Tenants') because the Tenants did not pay the rent that the Tenants owe.

This application was heard in Hamilton on February 3, 2011. The Landlord representative, K.H., and the Tenants attended the hearing.

At the hearing the Tenants raised the following issues pursuant to section 82 of the *Residential Tenancies Act, 2006* (the 'Act'): Bed bugs

Determinations and Reasons:

1. The Tenants have not paid the total rent they were required to pay for the period from May 1, 2010 to February 28, 2011. Because of the arrears, the Landlord served a Notice of Termination effective October 27, 2010.

2. The Tenants remain in possession of the rental unit.

3. The monthly rent is $799.00.

4. The Landlord collected a rent deposit of $759.00 from the Tenants and this deposit is still being held by the Landlord.

5. Interest on the rent deposit is owing to the Tenants for the period from March 29, 2010 to October 27, 2010.

6. The Tenants did not dispute the quantum of arrears. They believe that they should not be required to pay rent for the months in which their rental unit was infested with bed bugs.

7. The Tenants became aware of a bed bug infestation shortly after taking possession of the rental unit in April of 2010. The Landlord was informed and promptly arranged for spraying by Magical Pest Control.

8. Magical Pest Control inspected the rental unit on April 14, 2010. They sprayed the rental unit on April 19. After the Tenants reported that the bedbugs persisted, re-treatment occurred on May 17, June 17 and July 13. Finally, heat treatment was performed on September 20, 2010. The heat treatment has resolved the infestation.

9. In addition to requesting re-treatment each time the Tenants reported bed bugs, the Landlord voluntarily provided 100% rent abatements for the months of May, June and July.

2011 CanLII 13438 (ON LTB)

10. The Tenants sought a further rent abatement. They also sought compensation for heat damage to two televisions caused by the heat treatment.

11. Pictures and documentation submitted by the Tenants confirm that they suffered considerably from the bed bugs. However, the Landlord has responded promptly and responsibly to each complaint. I also find that the Landlord was more than reasonable in offering a 100% abatement of rent for the months of May, June and July. In light of these factors, I decline to offer a further abatement of rent.

12. I further decline to order compensation for the heat damage to the televisions. The Tenants acknowledged that the televisions were older and that they continue to operate properly.

13. I have considered all of the disclosed circumstances in accordance with subsection 83(2) of the *Residential Tenancies Act, 2006* (the 'Act'), and find that it would not be unfair to postpone the eviction until February 28, 2011, pursuant to subsection 83(1)(b) of the Act.

It is ordered that:

1. The tenancy between the Landlord and the Tenants is terminated. The Tenants must move out of the rental unit on or before February 28, 2011.

2. The Tenants shall pay to the Landlord $3,165.75*, which represents the amount of rent owing and compensation up to February 7, 2011, less the rent deposit and interest the Landlord owes on the rent deposit.

3. The Tenants shall also pay to the Landlord $26.27 per day for compensation for the use of the unit starting February 8, 2011 to the date they move out of the unit.

4. The Tenants shall also pay to the Landlord $170.00 for the cost of filing the application.

5. If the Tenants do not pay the Landlord the full amount owing* on or before February 28, 2011, the Tenants will start to owe interest. This will be simple interest calculated from March 1, 2011 at 3.00% annually on the balance outstanding.

6. If the unit is not vacated on or before February 28, 2011, then starting March 1, 2011, the Landlord may file this order with the Court Enforcement Office (Sheriff) so that the eviction may be enforced.

7. Upon receipt of this order, the Court Enforcement Office (Sheriff) is directed to give vacant possession of the unit to the Landlord, on or after March 1, 2011.

8. If, on or before February 28, 2011, the Tenants pay the amount of $4,684.00** to the Landlord or to the Board in trust, this order for eviction will be void. This means that the tenancy would not be terminated and the Tenants could remain in the unit. If this payment is not made in full and on time, the Landlord may file this order with the Court Enforcement Office (Sheriff) so that the eviction may be enforced.

9. The Tenants may make a motion to the Board under subsection 74(11) of the Act to set aside this order if they pay the amount required under that subsection on or after March 1, 2011 but before the Sheriff gives vacant possession to the Landlord. The Tenants are only entitled to make this motion once during the period of the tenancy agreement with the Landlord.

February 7, 2011
Date Issued

Michael Soo
Member, Landlord and Tenant Board

Southern-RO
119 King Street West, 6th Floor
Hamilton ON L8P4Y7

If you have any questions about this order, call 416-645-8080 or toll free at 1-888-332-3234.

In accordance with section 81 of the Act, the part of this order relating to the eviction expires on September 1, 2011 if the order has not been filed on or before this date with the Court Enforcement Office (Sheriff) that has territorial jurisdiction where the rental unit is located.

* Refer to section A on the attached Summary of Calculations.
** Refer to section B on the attached Summary of Calculations.

2011 CanLII 13438 (ON LTB)

Schedule 1
SUMMARY OF CALCULATIONS

File Number: SOL-10799-10

2011 CanLII 13438 (ON LTB)

A. Amount the Tenants must pay if the tenancy is terminated:

Reasons for amount owing	Period	Amount
Arrears: (up to the termination date in the Notice of Termination)	May 1, 2010 to October 27, 2010	$2,427.25
Less the amount the Tenants paid to the Landlord		-$1,199.00
Plus compensation: (from the day after the termination date in the Notice to the date of the order)	October 28, 2010 to February 7, 2011	$2,705.81
Less the rent deposit:		-$759.00
Less the interest owing on the rent deposit:	March 29, 2010 to October 27, 2010	-$9.31
Amount owing to the Landlord on the order date:(total of previous boxes)		**$3,165.75**
Additional costs the Tenants must pay to the Landlord:		$170.00
Plus daily compensation owing for each day of occupation starting February 8, 2011:		$26.27 (per day)
**Total the Tenants must pay the Landlord if the tenancy is terminated:**		**$3,335.75, + $26.27 per day starting February 8, 2011**

B. Amount the Tenants must pay to void the eviction order and continue the tenancy:

Reasons for amount owing	Period	Amount
Arrears:	May 1, 2010 to February 28, 2011	$5,713.00
Less the amount the Tenants paid to the Landlord		-$1,199.00
Less amount owing to the Tenant for abatement/rebate:		$0.00
Additional costs the Tenants must pay to the Landlord:		$170.00
**Total the Tenants must pay to continue the tenancy:**	On or before February 28, 2011	$4,684.00
