# Supplementary material for: Analysis of 44 Cases before the Landlord and Tenant Board Involving Bed Bug Infestations in Ontario, Canada: Focus on Adjudicator Decisions Based on Entomological/Pest Management Evidence and Accountability under the Residential Tenancy Act and Other Applicable Legislation
Source: Insects. 2011 Jul 19;2(3):343–53. doi: 10.3390/insects2030343 (PMC4553548; doi:10.3390/insects2030343)
Supplement: Supplementary File 1 [file insects-02-00343-s001.zip › supplementary-2/2011canlii13481.pdf]

Landlord and Tenant Board

Ontario

Order under Section 30
Residential Tenancies Act, 2006

File Number: CET-08623-10

2011 CanLII 13481 (ON LTB)

AC (the 'Tenant') applied for an order determining that PK (the 'Landlord') failed to meet the Landlord's maintenance obligations under the *Residential Tenancies Act, 2006* (the 'Act') or failed to comply with health, safety, housing or maintenance standards.

This application was heard in Bracebridge on December 16, 2010. The Tenant and the Landlord attended the hearing.

Determinations:

1. Based on the Tenant's evidence, the Landlord has failed to rectify an issue with bed bugs in the rental unit since July 2010. Therefore, I find that the Landlord failed to meet the Landlord's obligations under subsection 20(1) of the Act to maintain the rental unit.

2. The Landlord is not responsible for the losses claimed for by the Tenant in her application because she has either failed to demonstrate the loss or has failed to demonstrate that she attempted to mitigate her loss.

3. The allegation by the Tenant that the Landlord has failed to respond to a concern with bed bugs since 2008 is falsely exaggerated.

4. The Tenant has paid $900.00 to the Board in trust since the application was filed. *

It is ordered that:

1. The Landlord shall correct the problem with bed bugs in the rental unit immediately.

2. The Landlord shall pay the Tenant $45.00 for the cost of filing the application by February 28, 2011.

3. If the Landlord does not pay the Tenant the full amount owing by February 28, 2011, the Tenant may recover this amount by deducting $45.00 from the rent for March 2011.

4. The Board shall pay to the Landlord the amount of $900.00 together with any accrued interest. *

February 22, 2011
Date Issued

Enza Buffa
Member, Landlord and Tenant Board

Central-RO
3 Robert Speck Pkwy, Suite 520, 5th Floor
Mississauga Ontario L4Z2G5

If you have any questions about this order, call 416-645-8080 or toll free at 1-888-332-3234.

2011 CanLII 13481 (ON LTB)
