# Supplementary material for: Analysis of 44 Cases before the Landlord and Tenant Board Involving Bed Bug Infestations in Ontario, Canada: Focus on Adjudicator Decisions Based on Entomological/Pest Management Evidence and Accountability under the Residential Tenancy Act and Other Applicable Legislation
Source: Insects. 2011 Jul 19;2(3):343–53. doi: 10.3390/insects2030343 (PMC4553548; doi:10.3390/insects2030343)
Supplement: Supplementary File 1 [file insects-02-00343-s001.zip › supplementary-2/2011canlii26964.pdf]

Landlord and Tenant Board

Ontario

Order under Section 69
Residential Tenancies Act, 2006

File Number: TEL-12483-11

2011 CanLII 26964 (ON LTB)

('RM') (the 'Landlord') applied for an order to terminate the tenancy and evict ('BH') (the 'Tenant') because the Tenant did not pay the rent that the Tenant owes.

This application was heard in Toronto on March 31, 2011.

The Landlord's Agent, ('DF') and the Tenant attended the hearing.

At the hearing the Tenant raised the following issue pursuant to section 82 of the *Residential Tenancies Act, 2006* (the 'Act'): The Tenant claimed that the Landlord has not been responsive to her requests to address a problem with a bed bug infestation in her rental unit.

Determinations:

1. The Tenant has not paid the total rent she was required to pay for the period from February 1, 2011 to March 31, 2011. Because of the arrears, the Landlord served a Notice of Termination effective February 22, 2011.

2. The current lawful rent is $868.74 per month.

3. After the application was filed on February 23, 2011, the Tenant made no further payment to the Landlord leaving a rent arrears balance of $1,737.48 owing to March 31, 2011.

4. The Landlord collected a rent deposit of $869.35 from the Tenant and this deposit is still being held by the Landlord.

5. Interest on the rent deposit is owing to the Tenant for the period from February 1, 2011 to February 22, 2011.

6. The Tenant testified that she verbally advised the Landlord's superintendent about the presence of bed bugs in her unit towards the end of January 2011. The Tenant then put her concerns in writing on or about February 15, 2011. The Landlord promptly provided the Tenant with a 24 hour written notice to enter the unit on February 17, 2011 to respond to the bed bug complaint. The Tenant was also provided the Tenant with instructions on how to prepare the rental unit prior to receiving the bed bug treatment. The Tenant objected to her unit being treated with pesticides citing health concerns including the fact that she has a young child. The Tenant then requested that the unit be treated for bed bugs by chemical free means and in particular a heat treatment. According to the Tenant, the Landlord refused this approach citing cost considerations.

7. The Landlord maintains that city of Toronto public health recommends spraying with insecticides as the standard treatment for bed bugs. When the Tenant refused to have

the unit sprayed, the Landlord agreed to pay for the cost of a steam cleaner that the Tenant requested as a way to address the bed bug issued.

8. The Landlord served the Tenant with a subsequent notice of entry for March 3, 2011 for the purpose of inspecting the rental unit for the presence of bed bugs. The Tenant denied entry to the pest control workers retained by the Landlord to carry out the inspection. The Tenant cited the apparent presence of pesticides on the uniforms worn by the workers.

9. I am not satisfied that the Landlord has failed to meet its maintenance obligations with respect to responding to the complaint regarding bed bugs. In arriving at this determination, I take into consideration the Landlord's efforts to respond to the complaint within a reasonable period of time and the Tenant's conduct which interfered with the Landlord's efforts to treat the rental unit with an approved treatment method using a professional pest control company.

10. However, the Tenant is entitled to a rent rebate of $93.82 which the Landlord's Agent conceded he had offered to reimburse the Tenant for the cost of renting a steamer.

11. I have considered all of the disclosed circumstances in accordance with subsection 83(2) of the *Residential Tenancies Act, 2006* (the 'Act'), and find that it would be unfair to grant relief from eviction pursuant to subsection 83(1) of the Act. The Tenant advised that she has the financial resources to pay all of the arrears owing to the Landlord by April 15, 2011.

2011 CanLII 26964 (ON LTB)

It is ordered that:

1. The tenancy between the Landlord and the Tenant is terminated. The Tenant must move out of the rental unit on or before April 15, 2011.

2. The Tenant shall pay to the Landlord $835.77*, which represents the amount of rent owing and compensation up to April 4, 2011, less the rent deposit and interest the Landlord owes on the rent deposit and less the amount of the rebate the Landlord owes the Tenant.

3. The Tenant shall also pay to the Landlord $28.56 per day for compensation for the use of the unit starting April 5, 2011 to the date she moves out of the unit.

4. The Tenant shall also pay to the Landlord $170.00 for the cost of filing the application.

5. If the Tenant does not pay the Landlord the full amount owing* on or before April 15, 2011, the Tenant will start to owe interest. This will be simple interest calculated from April 16, 2011 at 3.00% annually on the balance outstanding.

6. If the unit is not vacated on or before April 15, 2011, then starting April 16, 2011, the Landlord may file this order with the Court Enforcement Office (Sheriff) so that the eviction may be enforced.

7. Upon receipt of this order, the Court Enforcement Office (Sheriff) is directed to give vacant possession of the unit to the Landlord, on or after April 16, 2011.

8. **If, on or before April 15, 2011, the Tenant pays the amount of $2,682.40** to the Landlord or to the Board in trust, this order for eviction will be void. This means that the tenancy would not be terminated and the Tenant could remain in the unit. If this payment is not made in full and on time, the Landlord may file this order with the Court Enforcement Office (Sheriff) so that the eviction may be enforced.**

9. The Tenant may make a motion to the Board under subsection 74(11) of the Act to set aside this order if they pay the amount required under that subsection on or after April 16, 2011 but before the Sheriff gives vacant possession to the Landlord. The Tenant is only entitled to make this motion once during the period of the tenancy agreement with the Landlord.

2011 CanLII 26964 (ON LTB)

April 4, 2011
Date Issued

Louis Bourgon
Member, Landlord and Tenant Board

Toronto East-RO
2275 Midland Avenue, Unit 2
Toronto ON M1P3E7

If you have any questions about this order, call 416-645-8080 or toll free at 1-888-332-3234.

In accordance with section 81 of the Act, the part of this order relating to the eviction expires on October 16, 2011 if the order has not been filed on or before this date with the Court Enforcement Office (Sheriff) that has territorial jurisdiction where the rental unit is located.

* Refer to section A on the attached Summary of Calculations.
** Refer to section B on the attached Summary of Calculations.

Schedule 1
SUMMARY OF CALCULATIONS

File Number: TEL-12483-11

2011 CanLII 26964 (ON LTB)

A. Amount the Tenant must pay if the tenancy is terminated:

**Reasons for amount owing**	**Period**	**Amount**
Arrears: (up to the termination date in the Notice of Termination)	February 1, 2011 to February 22, 2011	$628.35
Plus compensation: (from the day after the termination date in the Notice to the date of the order)	February 23, 2011 to April 4, 2011	$1,170.96
Less the rent deposit:		-$869.35
Less the interest owing on the rent deposit:	February 1, 2011 to February 22, 2011	-$0.37
Less amount owing to the Tenant for abatement/rebate:		-$93.82

Amount owing to the Landlord on the order date:(total of previous boxes)	**$835.77**
Additional costs the Tenant must pay to the Landlord:	$170.00
Plus daily compensation owing for each day of occupation starting April 5, 2011:	$28.56 (per day)
**Total the Tenant must pay the Landlord if the tenancy is terminated:**	**$1,005.77, + $28.56 per day starting April 5, 2011**

B. Amount the Tenant must pay to void the eviction order and continue the tenancy:

Reasons for amount owing	Period	Amount
Arrears:	February 1, 2011 to April 30, 2011	$2,606.22
Less amount owing to the Tenant for abatement/rebate:		-$93.82
Additional costs the Tenant must pay to the Landlord:		$170.00
**Total the Tenant must pay to continue the tenancy:**	On or before April 15, 2011	$2,682.40

2011 CanLII 26964 (ON LTB)
