# Supplementary material for: Analysis of 44 Cases before the Landlord and Tenant Board Involving Bed Bug Infestations in Ontario, Canada: Focus on Adjudicator Decisions Based on Entomological/Pest Management Evidence and Accountability under the Residential Tenancy Act and Other Applicable Legislation
Source: Insects. 2011 Jul 19;2(3):343–53. doi: 10.3390/insects2030343 (PMC4553548; doi:10.3390/insects2030343)
Supplement: Supplementary File 1 [file insects-02-00343-s001.zip › supplementary-2/2011canlii27139.pdf]

Landlord and Tenant Board

Ontario

Order under Sections 69 and 89
Residential Tenancies Act, 2006 and
Order under Section 21.2 of the
Statutory Powers Procedure Act

File Numbers: TSL-02945-10-RV
TSL-05538-10
TSL-11420-10

2011 CanLII 27139 (ON LTB)

RMMSA (the 'Landlord') applied for an order to terminate the tenancy and evict RWT (the 'Tenant') because the Tenant did not pay the rent that the Tenant owes. That application is contained in Board file TSL-02945-10 and was resolved by order TSL-02945-10 issued on March 26, 2010. On May 25, 2010, the Tenant requested a review of the order.

Then on May 28, 2010 the Landlord filed the application contained in Board file TSL-05538-10 for an order to terminate the tenancy and evict the Tenant because he or another occupant of the rental unit has committed an illegal act or has carried out, or permitted someone to carry out an illegal trade, business or occupation in the rental unit or the residential complex involving the production of an illegal drug, the trafficking in an illegal drug or the possession of an illegal drug for the purposes of trafficking.

Finally, on December 10, 2010 the Landlord filed the application contained in Board file TSL-11420-10 for an order to terminate the tenancy and evict the Tenant because he, another occupant of the rental unit or someone he permitted in the residential complex has substantially interfered with the reasonable enjoyment or lawful right, privilege or interest of the Landlord or another tenant; and because he, another occupant of the rental unit or someone he permitted in the residential complex has wilfully or negligently caused undue damage to the premises. The Landlord has also applied for an order requiring the Tenant to compensate the Landlord for the damage.

The request for review and the two subsequent applications were heard together in Toronto on January 28 and April 7, 2011.

The Landlord and the Tenant attended the hearing. The Landlord was represented by DC. The Tenant was represented by LM. The Landlord called as witnesses: AT (the ‘Security Supervisor’); LB (the ‘Resident Manager’); and MP (the ‘Police Constable’). The Tenant testified on his own behalf and called no further witnesses.

Determinations:

Preliminary Issue

1. The applications before the Board actually name two different Landlords. After hearing the submissions of the parties, I amended the applications to more accurately reflect the name of the corporate Landlord.

The Request for Review TSL-02945-10

2. As I stated at the hearing the request for review with respect to Board file TSL-02945-10 appears to have become moot with the passage of time. In that case the parties appeared before the Board on March 25, 2010 and consented to an order that gave the Tenant until April 30, 2010 to pay all of the arrears of rent and costs owing. On May 25, 2010 the Tenant filed a request to review. In response to my questions the Landlord's representative stated that in the meantime the Tenant has paid everything that was owed under the order and there are no longer any outstanding arrears. Under the circumstances it seems to me the logical thing to do is grant the review and dismiss the Landlord's application. As the Landlord's representative did not object to this solution, an order will issue accordingly.

2011 CanLII 27139 (ON LTB)

The Illegal Act Application TSL-05538-10

3. On April 22, 2010 the Landlord served a notice of termination on the Tenant that alleged the Tenant had committed an illegal act involving drug trafficking or illegal drug production. The particulars on the notice of termination refer to an incident that occurred on April 1, 2010 when the police attended the unit "and found crack pipes, baggies, and pieces of crack inside…". The Landlord's Security Supervisor and the Tenant both testified about an incident involving the police which occurred the night of April 1, 2010. However, the police records that were summonsed for the hearing by the Landlord do not record any such incident.

4. According to the Landlord's Security Supervisor he entered the rental unit the night of April 1, 2010 in company with the police who did not have a warrant but entered without one because they had received a report of gunshots being heard inside the unit. The Security Supervisor testified he smelled something which made him feel dizzy, saw capsules of some sort spill onto the floor and incense bottles had fallen over. He created a report that day which said the police found crack pipes, baggies and pieces of crack. According to him no charges were laid because the police entered without a warrant. According to the Tenant no shots were fired, no drugs were found on the premises and the only reason crack pipes were there was because he used to collect them for a harm reduction organisation. The Tenant further testified that he does not let people use his unit to take drugs although he does use marijuana himself. The police records indicate the unit is a "known crack house" but no charges have ever been laid with respect to drugs in the unit. The records also indicate the Tenant told a police officer he smoked crack in the unit with a friend on another occasion when a friend died there but the Tenant denied that he had actually said that.

5. The primary difficulty with the Landlord's application here is that the notice of termination was served on the Tenant pursuant to subsection 61(2) of the *Residential Tenancies Act, 2006* (the 'Act'). Essentially section 61 creates two different notices of termination for illegal acts: those illegal acts where twenty days' notice is required; and illegal acts involving drug trafficking; drug production, or possession for the purposes of trafficking where only ten days' notice is required. Here the Landlord's notice indicated it was for drug trafficking, production or possession for the purposes of trafficking. The best that can be said for the Landlord's evidence is that it might support a finding that the Tenant

was consuming drugs for his own personal use on the night in question. Even if the evidence was sufficient to make such a finding given the burden of proof that rests on the Landlord, possession for the purposes of personal use does not constitute possession for the purposes of trafficking. Trafficking involves the possession of significant quantities of drugs beyond that which would normally be personally consumed. The heart of the offence is that the possessor has drugs to sell not to use. Possession for personal use is an illegal act which requires a twenty day notice of termination. That being said, based on the evidence before me the only thing I am satisfied the Tenant was in possession of that evening was drug paraphernalia. As a result, I find that the Landlord led insufficient evidence to establish the Tenant committed the offence of drug trafficking, production of an illegal substance, or possession for the purposes of trafficking.

6. As a result of all of the above, the application contained in Board file TSL-05538-10 shall be dismissed.

The Substantial Interference and Damage Application TSL-11420-10

7. On November 30, 2010 the Landlord service a notice of termination on the Tenant for substantial interference pursuant to section 64 of the Act and for negligent or wilful damage under section 62 of the Act. The Landlord’s application requests termination of the tenancy and includes a claim for damages in the amount of $970.34 pursuant to section 89 of the Act.

The Substantial Interference Claims

8. For my purposes here I would group and categorise the claims in the notice of termination with respect to substantial interference as follows: the bed bug and cockroach issue; the changing of the locks and doorknob; and the behaviour of the Tenant’s visitors.

THE BED BUG AND COCKROACH ISSUE

9. It was the evidence before me that on October 7, 2010 the Tenant requested treatment of his unit for pests by signing a work order. The Landlord happened to have pest control already scheduled for that day so they attended the unit but it was not ready for treatment so the landlord arranged for them to treat the unit on October 21, 2010. The records received from the pest control company indicate that when they went that day to spray the Tenant had just been released from the hospital and was not able to properly prepare the unit for treatment. On November 11, 2010 they attended at the unit again and were unable to treat the unit because the Tenant’s possessions had not been properly organised.

10. According to the Resident Manager she contacted Public Health because she knew that they had people who would come and help prepare tenants’ units for treatment if difficulties arose. A person from Public Health attended with her on November 22, 2010 at the Tenant’s unit and offered assistance in preparing for treatment. According to her the Tenant refused the assistance of Public Health and told them that he had friends who would help him prepare the unit for treatment.

2011 CanLII 27139 (ON LTB)

2011 CanLII 27139 (ON LTB)

11. On December 2, 2010, which was during the seven day voiding period following service of the notice of termination, the Landlord arranged for the pest control company to again go to the unit to treat it but the Tenant refused them entry. As a result of all of the above, the Resident Manager testified the Landlord has been physically unable to treat the Tenant's unit for pests even though (as the Security Supervisor testified) bugs have been seen crawling out of the Tenant's unit.

12. The Tenant did not contradict any of the Landlord's evidence concerning the Landlord's attempts to have the unit treated up to December 2, 2010 nor did he deny refusing access on December 2, 2010 or refusing the help offered by Public Health in November of 2010. Rather his evidence was all about what happened after December of 2010. As a result, I am satisfied that the Tenant substantially interfered with a lawful right, privilege or interest of the Landlord. I say this because the Landlord has a legal obligation to ensure the residential complex is pest free. By failing to co-operate with the Landlord's efforts to treat his unit and refusing access to pest control the Tenant effectively prevented the Landlord from fulfilling its legal obligation.

13. That being said it was the Tenant's evidence that after the hearing before me on January 28, 2011 he told the Resident Manager to contact Public Health again. Public Health re-attended his unit and told him some things would have to be thrown away, like his couch, but he could contact his worker at the Ontario Disability Support Programme to apply for Community Start Up and Maintenance Benefit to replace whatever furniture needed to be disposed of. According to the Tenant he arranged his unit ready for treatment and they were supposed to return to spray and treat the unit in mid-March but they never did and he heard nothing further from them. The Landlord led no reply evidence to contradict the Tenant's evidence concerning what happened with respect to pest control after January 28, 2011. As a result, I accept the Tenant's evidence that after January 28, 2011 he was prepared to co-operate with the Landlord and Public Health with regards to treating his rental unit. This evidence is relevant to the issue of relief from eviction which is discussed below.

THE CHANGING OF THE LOCKS AND DOORKNOB

14. It was the evidence before me that the Tenant's lock has actually been destroyed twice. The Tenant testified the first time it happened was because someone tried to break down his door. The second time it happened was because the police drilled the lock looking for him as he was scheduled to testify at a hearing involving criminal proceedings and he was not home so did not answer their knock when they came to transport him for the hearing. Only the first of these two occasions is mentioned in the notice of termination.

15. According to the Landlord's witnesses the Tenant did not complain about the broken lock or ask that it be repaired. The Security Superintendent noticed that the door and the lock deteriorated over time until eventually the door could be seen hanging from its hinges. Apparently the Tenant then installed a new door knob and lock of his own. Although the evidence was not clear on this point, I believe that it was not until after the first hearing before me on January 28, 2011 that the Landlord took steps to repair the Tenant's lock and door. No evidence was lead that the Landlord brought an application pursuant to

section 35 of the Act to get a key or to require the Tenant to pay the costs of changing the lock back to one the Landlord's key could open.

16. Clearly if a tenant changes the locks on his or her rental unit that can substantially interfere with a lawful interest of a landlord as a landlord needs to be able to get into a rental unit in case of emergencies. The notice of termination served on the Tenant specifically stated that to void the notice the Tenant had to restore the original doorknob and provide the Landlord with a key to the new lock but no evidence was led the Tenant did so. The Tenant offered no explanation as to why he did not request repairs from the Landlord or why he changed the locks and doorknob himself or why he failed to provide the Landlord with a replacement key. As a result, I am satisfied that the Tenant substantially interfered with a lawful interest of the Landlord's by changing the locks and failing to provide a replacement key.

2011 CanLII 27139 (ON LTB)

THE BEHAVIOUR OF THE TENANT'S VISITORS

17. The notice of termination served on the Tenant alleges that in the small hours of the morning the Tenant regularly has a large number of visitors constantly coming and going. It also details incidents that occurred overnight on November 18 to 19, 2010.

18. The Tenant did not deny that he has a significant number of visitors in the wee hours of the morning. According to him he suffers from insomnia and his friends will visit late because that is when he is up and wanting company. He estimated that five or six people will visit him every day.

19. The Security Supervisor testified that he has seen people exit the Tenant's unit smoking drugs and he has seen people who have been issued trespass notices for the building going into the Tenant's unit but neither of those allegations is contained in the notice of termination. The Security Supervisor also testified about a videotape of activity outside the Tenant's unit one night which was shown to me. It shows the Tenant receiving a number of visitors going in and out. However, it also shows dozens of people entering the back door and walking past the Tenant's unit into the rest of the residential complex during the same period of time. In answer to my question as to who all these other people were he stated they were mostly other tenants of the building returning home late. The number of people visiting the Tenant seen on the tape was small in comparison to the number of people entering and exiting the building past the Tenant's unit. As a result, I do not believe the shear number of visitors to the Tenant's unit is particularly problematic as the unit is adjacent to the rear door and dozens of other people use that door regularly late at night.

20. The Landlord led considerable evidence about the kinds of people who visit the Tenant. According to both of the Landlord's witnesses the Tenant's guests are street involved people whose behaviour intimidates the Landlord's staff and other tenants. It was the submission of the Landlord that the Tenant is a "magnet" for undesirables. It was the submission of the Tenant's representative that because the Tenant lived on the street and in the City's shelters for many years it is not surprising that his guests are often street involved people. I agree with the submission of the Tenant's representative that a tenant cannot be evicted because of the way his guests look; rather the issue is what behaviour

the guests engage in that disturb others. If the behaviour of the Tenant's guests disturbs the Landlord or other tenants in the residential complex then the notice of termination must set out some details with respect to that behaviour. Here the only details in the notice with respect to guest behaviour involve the events of the night of November 18 to 19, 2010 and the guests' involvement in the damage to the unit. (The issue of damage to the unit is discussed separately and in more detail below.)

2011 CanLII 27139 (ON LTB)

21. With respect to the events of November 18 to 19, 2010 the only evidence led by the Landlord was hearsay. The Landlord entered into evidence a report of the security guard who was on duty that night. The security report essentially says that a visitor asked to be accompanied to the Tenant's unit and was asked to leave. When the visitor refused the security guard said he would call the police and then the Tenant could be heard saying "no cops" inside the unit. The Tenant then let the visitor in. When patrolling the exterior of the unit the security guard noticed the Tenant's window had been broken and he then called police "regarding problems in 107" which are not specified. According to the Resident Manager she was told the problems involved noise and a suspicion of physical violence. When the police arrived and knocked on the Tenant's door there was no answer and the lights were out so the police left. The police records filed into evidence before me do not mention anything concerning this event.

22. The difficulty with the Landlord's application concerning the behaviour of the Tenant's guests is that very little of the behaviour complained of is actually referred to in the notice of termination. If it is not in the notice of termination then it cannot be grounds for terminating the tenancy. With regards to the particular incident that is referred to in the notice, the evidence led merely establishes that the Tenant had a guest who refused to leave, someone broke the Tenant's window and the police were called as a result of some sort of disturbance. Although I have no reason to doubt the veracity of this evidence it simply is no sufficient to make a finding that the Tenant or his guests substantially interfered with the Landlord or another tenant. For example, no evidence was led as to why the Tenant's guest was asked to leave. The security report merely refers to the guest's appearance. No evidence was led as to who broke the Tenant's window, what noise was heard or how it disturbed anyone, or what violence if any occurred. As a result of all of the above, I am of the view that the Landlord led insufficient evidence to establish that the behaviour of the Tenant's guests set out in the notice of termination substantially interfered with the Landlord or another tenant's reasonable enjoyment or other lawful interest, privilege or right.

<u>The Damage Claims</u>

23. The notice of termination served on the Tenant on November 30, 2010 alleged that the Tenant wilfully or negligently damaged the rental unit or residential complex by breaking the window and destroying closet doors.

24. With respect to the broken window the Landlord's witnesses could not say who broke the Tenant's window. They believe it was broken when one of his frequent guests threw a brick or other object at the window. On cross-examination the Security Supervisor conceded that it was certainly possible that the individual in question did this because the Tenant refused to let him in. The Tenant testified that was exactly what happened.

Someone he did not want as a guest attempted to gain entry and when the Tenant refused the person broke the window. As the individual in question was not permitted into the residential complex by the tenant then it cannot be said he was a guest of the Tenant's. As a result, I am not prepared to find that the Tenant or a guest of the Tenant's wilfully or negligently broke the Tenant's window.

2011 CanLII 27139 (ON LTB)

25. There was no real dispute between the parties that the Tenant did break the closet doors. The Tenant uses a wheelchair but has some mobility in his legs. According to him the closet doors were damaged because he would fall into them and they would come off their runners and be damaged. He had friends remove them from the rental unit. There was also no dispute that the Tenant has not paid the Landlord the cost of replacing the closet doors since the notice was served. According to the Tenant he has no objection to paying for the doors but does not want them replaced until after he vacates the rental unit as he considers them a nuisance. The Tenant led no evidence at all concerning his medical condition so I have no idea if the Tenant's falling and subsequent destruction of the doors was related to a disability or otherwise unavoidable. As a result, I am satisfied that the Tenant negligently damaged the closet doors of the rental unit and failed to void the notice by paying to the Landlord the reasonable cost of repair within seven days of service.

26. It was the undisputed evidence of the Landlord that the cost of replacing the closet doors is $169.15 and the Landlord is entitled to recover that amount from the Tenant.

<u>Relief from Eviction</u>

27. Section 83 of the Act says that on an application for eviction the Board may refuse to grant the application "unless satisfied, having regard to all the circumstances, that it would be unfair to refuse". The Board also has the power to delay eviction or to make conditional orders. It has often been said that eviction is a remedy of last resort. In other words if the problems can be resolved so the tenancy can peacefully continue, eviction should not be ordered. With respect to the specific grounds and reasons for eviction raised by this application, those are amenable to conditional orders. For example, I could issue an order requiring the Tenant to pay the Landlord the cost of replacing the closet doors and refuse eviction if payment is made. I could also order the Tenant to co-operate with the Landlord's efforts to deal with pest control issues and to refrain from altering the locking system on his rental unit again with a section 78 clause permitting the Landlord to apply for eviction if the Tenant should fail to obey.

28. The problem with looking at relief in this way is that it ignores the requirement in the Act to look at "all the circumstances". I cannot ignore the evidence before me that the police were in the Tenant's unit at least ten times in 2010 due to a variety of complaints, or the evidence of the Security Superintendent that threats from the tenant's guests have caused one security guard to quit and another to transfer, or the fact that thirty-seven of the Tenant's neighbours signed a petition urging the Landlord to evict the Tenant "in order to prevent further health hazards and criminal activity". In response to a question from his representative about these kinds of concerns the Tenant belittled the people who signed the petition by saying he had seen many petitions in his day and the number of signatures was "nothing compared with the number of people who live in the building".

Similarly I cannot ignore the fact that the Tenant is frail, appears to be limited in his mobility and is low income. Nor can I ignore the fact that the Tenant was homeless for many years until Streets to Homes placed him in this unit in February of 2009. All of the Tenant's personal circumstances will make it difficult for him to find alternative housing.

29. It seems to me that the Tenant's attitude towards the neighbours who signed the petition speaks volumes about the unfairness that the Landlord will suffer should eviction be refused. Given that the Tenant cares very little about the concerns of thirty-seven of his neighbours I believe that he has very little insight into how his behaviour affects those around him. As a result it is likely that the Tenant will continue to be un-cooperative with the Landlord and its staff in their efforts to maintain the residential complex and keep it safe and secure for everyone in the building because he does not perceive his past behaviour as being particularly problematic. His attitude is such that it is inevitable that more conflict will occur and more incidents involving police calls late at night will arise. As a result, I am of the view that this is not an appropriate case for a conditional order. Rather, an eviction order will issue but the Tenant will be given extra time to find alternative housing given the challenges he will face in doing so.

2011 CanLII 27139 (ON LTB)

Costs and Compensation

30. The Landlord incurred costs of $170.00 for filing the application and is entitled to reimbursement for those costs.

31. Pursuant to subsection 89(2) of the Act the damage amount of $169.15 shall be deducted from the last month's rent deposit currently being held by the Landlord which reduces it to $543.65.

32. Interest on the rent deposit is owing to the Tenant for the period from February 1, 2010 to December 20, 2010. I calculate the interest owing is $13.24.

33. The Tenant has paid rent for the period ending April 30, 2011. Therefore, he shall he required to pay daily compensation for each day he remains in the rental unit after April 30, 2011. However, the Landlord shall credit the remainder of the deposit and the interest owed against the compensation due.

34. This order contains all of the reasons for my decision within it. No further reasons shall be issued.

It is ordered that:

1. The request to review Board order TSL-02945-10 issued on March 26, 2010 is granted. The order is cancelled and the Landlord's application is dismissed.

2. The Landlord's application contained in Board file TSL-05538-10 is also dismissed.

3. With respect to the application contained in Board file TSL-11420-10 the tenancy between the Landlord and the Tenant is terminated. The Tenant must move out of the rental unit on or before May 10, 2011.

4. The Landlord shall deduct the reasonable cost of replacing the closet doors of $169.15 from the last month’s rent deposit currently being held by the Landlord.

5. The Landlord shall refund to the Tenant the remainder of his deposit and the interest due on the deposit (which is $543.65 plus $13.24 or $556.89) less: $23.57 per day for compensation for the use of the unit from May 1, 2011 to the date the Tenant moves out of the unit; and $170.00 for the cost of filing the application.

6. If the unit is not vacated on or before May 10, 2011, then starting May 11, 2011, the Landlord may file this order with the Court Enforcement Office (Sheriff) so that the eviction may be enforced.

7. Upon receipt of this order, the Court Enforcement Office (Sheriff) is directed to give vacant possession of the unit to the Landlord on or after May 11, 2011.

2011 CanLII 27139 (ON LTB)

April 14, 2011
Date Issued

Ruth Carey
Member, Landlord and Tenant Board

Toronto South-RO
79 St. Clair Avenue East, Suite 212, 2nd Floor
Toronto ON M4T1M6

If you have any questions about this order, call 416-645-8080 or toll free at 1-888-332-3234.

In accordance with section 81 of the Act, the part of this order relating to the eviction expires on November 11, 2011 if the order has not been filed on or before this date with the Court Enforcement Office (Sheriff) that has territorial jurisdiction where the rental unit is located.
