# Supplementary material for: Analysis of 44 Cases before the Landlord and Tenant Board Involving Bed Bug Infestations in Ontario, Canada: Focus on Adjudicator Decisions Based on Entomological/Pest Management Evidence and Accountability under the Residential Tenancy Act and Other Applicable Legislation
Source: Insects. 2011 Jul 19;2(3):343–53. doi: 10.3390/insects2030343 (PMC4553548; doi:10.3390/insects2030343)
Supplement: Supplementary File 1 [file insects-02-00343-s001.zip › supplementary-2/2011canlii27370.pdf]

Landlord and Tenant Board

Ontario

Order under Section 135
Residential Tenancies Act, 2006

File Number: CET-11437-11

2011 CanLII 27370 (ON LTB)

EF, SD and JF (the 'Tenants') applied for an order determining that BCEI (the 'Landlord') has collected or retained money illegally.

This application was heard in Barrie on April 19, 2011.

The Tenants and the Landlord's representative, JG, attended the hearing.

Preliminary Matter:

The application was amended at the hearing to remove HD, as a Tenant. She is a two year old child and was an occupant of the rental unit.

Determinations:

1. The Landlord did not collect or retain money illegally.

2. The Tenants allege that the unit was not in a good state of repair or fit for habitation due to a beg bug infestation. They claim that the rent charged is illegal rent due to the bedbugs and they seek a return of the full rent paid to the Landlord.

3. The alleged bed bug infestation is a maintenance issue. The alleged infestation does not mean the Landlord charged illegal rent.

4. The application filed is not relevant to the issue and the remedy sought. There is a specific T6 maintenance application to address the issue the Tenants wish to raise and the remedy they are seeking.

It is ordered that:

The Tenants' application is dismissed.

April 20, 2011
Date Issued

Karen Wallace
Member, Landlord and Tenant Board

Central-RO
3 Robert Speck Pkwy, Suite 520, 5th Floor
Mississauga Ontario L4Z2G5

If you have any questions about this order, call 416-645-8080 or toll free at 1-888-332-3234.
