# Supplementary material for: Analysis of 44 Cases before the Landlord and Tenant Board Involving Bed Bug Infestations in Ontario, Canada: Focus on Adjudicator Decisions Based on Entomological/Pest Management Evidence and Accountability under the Residential Tenancy Act and Other Applicable Legislation
Source: Insects. 2011 Jul 19;2(3):343–53. doi: 10.3390/insects2030343 (PMC4553548; doi:10.3390/insects2030343)
Supplement: Supplementary File 1 [file insects-02-00343-s001.zip › supplementary-2/2011canlii34410.pdf]

Landlord
and
Tenant
Board
Ontario

Order under Sections 30, 69
Residential Tenancies Act, 2006

File Number: TEL-08981-10 /
TET-10469-10

2011 CanLII 34410 (ON LTB)

L1 application:

The 'Landlord' applied for an order to terminate the tenancy and evict K.R. (the 'Tenant') because the Tenant did not pay the rent that the Tenant owes.

T6 application:

The Tenant applied for an order determining that the Landlord failed to meet the Landlord's maintenance obligations under the *Residential Tenancies Act, 2006* (the 'Act') or failed to comply with health, safety, housing or maintenance standards.

This cross-application was heard in Toronto on March 15, 2011 and April 19, 2011.

Appearances:

D.R., the Landlord's Legal Representative (March 15, 2011 appearance)
F.A., the Landlord's Agent (superintendent) (March 15, 2011 appearance)
D.C., the Landlord's Legal Representative (April 19, 2011 appearance)
K.R., the Tenant

The Proceedings:

1. The Landlord filed its L1 application on October 22, 2010.

2. The parties first appeared before the Board on November 30, 2010. During this appearance the Tenant requested, and was granted, an adjournment to obtain legal representation and to raise his own claims pursuant to section 82 of the *Residential Tenancies Act, 2006* (the 'Act').

3. As a condition of the adjournment, Member Burke issued interim order TEL-08981-10-IN requiring the Tenant to disclose to the Landlord by December 20, 2010 a copy of all documents and evidence the Tenant intended to submit at the next hearing plus a list of all issues the Tenant intended to raise at the next hearing. The Tenant was further ordered to pay the Landlord $893.38 by January 1, 2011. It should be noted that the interim order was issued at the hearing using one of the Board's triplicate carbon forms. The Member inscribed an asterisk next to the cautionary sentences warning the parties of the possible consequences of non-compliance with the interim order; namely, that the hearing Member would have the discretion to refuse to accept or consider a party's evidence or submissions.

4. On December 16, 2010, the Tenant filed his T6 application with the Board.

2011 CanLII 34410 (ON LTB)

5. The parties made a second appearance before Vice-Chair Fellman on January 25, 2011. On consent of the parties, the hearing was adjourned so that the Landlord’s application could be heard along with the Tenant’s application.

6. The parties third appearance was held before me on the following day, January 26, 2011. Due to a scheduling overflow, I was unable to hear this cross-application and the matter was adjourned.

7. The fourth appearance was held before me on March 15, 2011. Considerable time was spent during the hearing receiving submissions from both parties concerning the Tenant’s non-compliance with Member’s Burke’s interim order for disclosure and payment of money to the Landlord. The Landlord’s Legal Representative, DR, advised the Board that apart from serving the Landlord with the T6 application form, the Tenant had never disclosed any documents or other evidence. The Tenant countered that he had previously attempted to serve the Landlord’s Legal Representative with documents while at the Board but that his efforts were met with rejection. I found the Tenant’s claim to be implausible and lacking in credibility. The Landlord’s Legal Representative would have no personal vested interest in refusing to accept documents from the Tenant which in turn would allow the Landlord to mount a defence to the Tenant’s claims. Furthermore, the Tenant went on to contradict himself by pointing to page 2 of his application where the Tenant writes: “There will be documents to show.” When I asked the Tenant when he had intended to “show” his documents to the Landlord, the Tenant responded by stating “at the hearing.”

8. Additionally, the Tenant advised that his rent is paid directly to the Landlord by Ontario Works and that his benefits were never discontinued. The Tenant denied the Landlord’s claim that the Tenant had failed to make any rent payments in 2011. When asked what evidence he had to corroborate his claim of recent rent payments, the Tenant became evasive and then re-directed the dialogue by stating that there were outstanding maintenance issues in his rental unit.

9. In the end, it became very clear that the Tenant had completely disregarded Member Burke’s interim order. Due to insufficient time, the March 15, 2011 hearing had to be adjourned. Notwithstanding the Tenant’s breach of Member Burke’s order, given that the parties would be returning before me to complete this matter I issued a new direction to the Tenant with a view to getting matters on the right track. Specifically, the Tenant was ordered to pay $1,767.28 into the Board by April 1, 2011. This amount represented the March and April 2011 rent payments ($883.64 x 2). The Tenant advised that he understood the process of paying funds into the Board’s trust account. Furthermore, the Tenant was ordered to disclose to the Landlord all documents that he intended to rely on at the next hearing including rent payment receipts by April 1, 2011. At the hearing, the Landlord’s Legal representative provided the Tenant with his business card to facilitate the task of disclosure. Before concluding the hearing, I cautioned the Tenant about the risks that he ran if he failed to comply with my order given his previous disregard of Member Burke’s interim order.

10. The fifth appearance was held before me on April 19, 2011. The Tenant's pattern of disobeying this Board continued. The Tenant had made no payment into the Board's trust account. Furthermore, the Landlord's Legal Representative, DC who is an associate of DR, advised the Board that his office had only received a package of documents from the Tenant on or about April 18, 2011. Given that DC had spent the day of April 18, 2011 representing clients before this Board, he had only been given the Tenant's package of documents on the morning of April 19, 2011. DC advised that he was not in a position to respond to the Tenant's claims given the late hour at which he was provided with disclosure. DC entered into evidence a Purolator shipping receipt dated April 15, 2011.

11. When asked why the Tenant had disobeyed my interim order, the Tenant became argumentative and belligerent. The Tenant appears to be of the view that an order of this Board is merely a request or a negotiable matter. The Tenant also had the temerity to insist that he had complied with my interim order when clearly this was not the case. The Tenant also maintained that during the March 15, 2011 hearing, the Landlord's Legal Representative had asked to withdraw the Landlord's application. No such request was ever made by the Landlord's Legal Representative.

2011 CanLII 34410 (ON LTB)

Costs Against the Tenant:

12. The Landlord's Legal Representative sought permission to make submissions with respect to costs. The request was granted and both parties were invited to make submissions on costs including Board costs.

13. The Board has made reasonable efforts to accommodate the self-represented Tenant's adjournment request. In one instance, the Board even overlooked the fact that the Tenant disobeyed an interim order of this Board. However, the Tenant's continued blatant disregard of a second order of this Board constitutes an abuse of process.

14. The Tenant offered no apology and showed no remorse for having disobeyed two substantially similar interim orders of this Board. On the contrary, when asked to make submissions regarding his failure to comply with orders of this Board, the Tenant was evasive, belligerent and disrespectful. Also disquieting is the fact that the Tenant's original adjournment request made on November 30, 2010 was granted so that he could obtain legal representation. The hearing attendance record for all four subsequent appearances reveals that the Tenant appeared without a legal representative.

15. At the time the Landlord filed its application on October 22, 2010, the Landlord claimed rent arrears totalling $1,786.76 up to October 31, 2010. By the time this hearing was concluded on April 19, 2011, the arrears had grown to $3,599.44 up to April 30, 2011. No further rent payment was made by the Tenant after December 22, 2010 despite the Tenant's repeated claim that his rent was paid by Ontario Works.

16. I find that the Tenant has engaged in unreasonable conduct by: delaying the hearing, failing to follow the directions of this Board, showing a lack of respect for the Board's process and by showing contempt towards a Member of the Board. Pursuant to sections 204(2) and 204(3) of the Act, rules 27.2 and 27.3 of the Board's *Rules of Practice* and guideline 3 of the Board's *Interpretation Guidelines* the Tenant shall be required to pay

costs to the Landlord totalling $300.00 and a further $300.00 to the Landlord and Tenant Board for its costs. This award takes into consideration the Tenant's conduct and delays caused by the Tenant with respect to the November 30, 2010, March 15, 2011 and April 19, 2011 appearances.

2011 CanLII 34410 (ON LTB)

Control of the Board's Process:

17. In view of the Tenant's blatant disregard of not one but two interim orders of this Board, the Tenant was denied permission to introduce any documents not previously disclosed.

18. The Tenant was however permitted to make submissions with respect to relief from eviction consideration. Furthermore the Tenant was permitted to introduce evidence that could support a finding that the Landlord is in serious breach of its obligations under the Act which would trigger a mandatory refusal of the Landlord's application pursuant to section 83(3)a) of the Act.

Determinations:

1. The Tenant has not paid the total rent he was required to pay for the period from September 1, 2010 to November 30, 2010. Because of the arrears, the Landlord served a Notice of Termination effective September 24, 2010.

2. The lawful rent decreased from $893.38 per month to $883.64 per month effective January 1, 2011 pursuant to a notice of rent reduction due to a property tax decrease which was issued by the City of Toronto on December 15, 2010.

3. The Landlord collected a rent deposit of $875.00 from the Tenant and this deposit is still being held by the Landlord.

4. Interest on the rent deposit is owing to the Tenant for the period from September 1, 2009 to September 24, 2010.

5. The Tenant paid $2,625.00 after the application was filed as corroborated by the Landlord's rent ledger.

6. I have considered all of the disclosed circumstances in accordance with subsection 83(2) of the *Residential Tenancies Act, 2006* (the 'Act'). The Tenant was also invited to provide evidence and submissions on whether mandatory refusal of the Landlord's application was required under section 83(3)(a) of the Act which provides as follows:

> **Circumstances where refusal required**
>
> 83 (3) Without restricting the generality of subsection (1), the Board shall refuse to grant the application where satisfied that,
>
> (a) the landlord is in serious breach of the landlord's responsibilities under this Act or of any material covenant in the tenancy agreement;

7. The Tenant claimed that the Landlord was in serious breach of its maintenance obligations. In this regard, the Tenant was invited to introduce evidence that could support such a finding. In particular, the Tenant produced a report from the City of Toronto's Public Health Department. The report indicates that the Tenant lodged a complaint on November 1, 2010 regarding the presence of bed bugs in his unit. The report contains the inspector's investigative notes made between November 2, 2010 and November 18, 2010. The inspection notes reveal that the inspector contacted the Landlord's property manager on November 3, 2010 regarding the bed bug complaint. The property manager informed the inspector that the matter would be addressed. The final entry in the inspector's log is dated November 18, 2010 and is a contact with the Tenant. The inspector writes: "Spoke with the complainant. He sates the apt. was treated for bed bugs November 13, 2010. Advised him to assure a follow up treatment is done. He seemed to indicate it will be. No further action necessary at this time." Based on the inspection report entered into evidence, I am not satisfied that the Landlord is in serious of its maintenance obligations.

2011 CanLII 34410 (ON LTB)

It is ordered that:

1. The tenancy between the Landlord and the Tenant is terminated. The Tenant must move out of the rental unit on or before May 9, 2011.

2. The Tenant shall pay to the Landlord $3,607.70*, which represents the amount of rent owing and compensation up to May 2, 2011, less the rent deposit and interest the Landlord owes on the rent deposit.

3. The Tenant shall also pay to the Landlord $29.05 per day for compensation for the use of the unit starting May 3, 2011 to the date he moves out of the unit.

4. The Tenant shall also pay to the Landlord $470.00 for the cost of filing the application and other costs.

5. If the Tenant does not pay the Landlord the full amount owing* on or before May 9, 2011, the Tenant will start to owe interest. This will be simple interest calculated from May 10, 2011 at 3.00% annually on the balance outstanding.

6. The Landlord may immediately file this order with the Court Enforcement Office (Sheriff) so that the eviction may be enforced on or after May 10, 2011.

7. Upon receipt of this order, the Court Enforcement Office (Sheriff) is directed to give vacant possession of the unit to the Landlord, on or after May 10, 2011.

8. **If, on or before May 9, 2011, the Tenant pays the amount of $5,836.72** to the Landlord or to the Board in trust, this order for eviction will be void. This means that the tenancy would not be terminated and the Tenant could remain in the unit. If this payment is not made in full and on time, the Landlord may file this order with the Court Enforcement Office (Sheriff) so that the eviction may be enforced.**

9. The Tenant may make a motion to the Board under subsection 74(11) of the Act to set aside this order if the Tenant pays the amount required under that subsection on or after May 10, 2011 but before the Sheriff gives vacant possession to the Landlord. The Tenant is only entitled to make this motion once during the period of the tenancy agreement with the Landlord.

10. The Tenant shall also pay to the Landlord and Tenant Board costs in the amount of $300.00 by May 6, 2011.

11. The Tenant's T6 application is dismissed without prejudice.

2011 CanLII 34410 (ON LTB)

May 2, 2011
Date Issued

____
Louis Bourgon
Member, Landlord and Tenant Board

Toronto East-RO
2275 Midland Avenue, Unit 2
Toronto ON M1P3E7

If you have any questions about this order, call 416-645-8080 or toll free at 1-888-332-3234.

In accordance with section 81 of the Act, the part of this order relating to the eviction expires on November 10, 2011 if the order has not been filed on or before this date with the Court Enforcement Office (Sheriff) that has territorial jurisdiction where the rental unit is located.

* Refer to section A on the attached Summary of Calculations.
** Refer to section B on the attached Summary of Calculations.

Schedule 1
SUMMARY OF CALCULATIONS

File Number: TEL-08981-10 / TET-10469-10

2011 CanLII 34410 (ON LTB)

A. Amount the Tenant must pay if the tenancy is terminated:

Reasons for amount owing	Period	Amount
Arrears: (up to the termination date in the Notice of Termination)	September 1, 2010 to September 24, 2010	$704.91

Less the amount the Tenant paid to the Landlord		-$2,625.00
Plus compensation: (from the day after the termination date in the Notice to the date of the order)	September 25, 2010 to May 2, 2011	$6,422.36
Less the rent deposit:		-$875.00
Less the interest owing on the rent deposit:	September 1, 2009 to September 24, 2010	-$19.57

Amount owing to the Landlord on the order date:(total of previous boxes)	**$3,607.70**

Additional costs the Tenant must pay to the Landlord:	$170.00
Additional costs the Tenant must pay to the Landlord (hearing costs):	$300.00

Plus daily compensation owing for each day of occupation starting May 3, 2011:	$29.05 (per day)

**Total the Tenant must pay the Landlord if the tenancy is terminated:**	**$4,077.70, + $29.05 per day starting May 3, 2011**

B. Amount the Tenant must pay to void the eviction order and continue the tenancy:

**Reasons for amount owing**	**Period**	**Amount**
Arrears:	September 1, 2010 to May 31, 2011	$7,991.72
Less the amount the Tenant paid to the Landlord		-$2,625.00
Additional costs the Tenant must pay to the Landlord:		$170.00
Additional costs the Tenant must pay to the Landlord (hearing costs):		$300.00
**Total the Tenant must pay to continue the tenancy:**	On or before May 9, 2011	$5,836.72

2011 CanLII 34410 (ON LTB)
