# Supplementary material for: Analysis of 44 Cases before the Landlord and Tenant Board Involving Bed Bug Infestations in Ontario, Canada: Focus on Adjudicator Decisions Based on Entomological/Pest Management Evidence and Accountability under the Residential Tenancy Act and Other Applicable Legislation
Source: Insects. 2011 Jul 19;2(3):343–53. doi: 10.3390/insects2030343 (PMC4553548; doi:10.3390/insects2030343)
Supplement: Supplementary File 1 [file insects-02-00343-s001.zip › supplementary-2/2011canlii5939.pdf]

Landlord and Tenant Board

Order under Section 30
Residential Tenancies Act, 2006

File Number: CET-09365-10

2011 CanLII 5939 (ON LTB)

NB and RG (the 'Tenants') applied for an order determining that BG (the 'Landlord') failed to meet the Landlord's maintenance obligations under the *Residential Tenancies Act, 2006* (the 'Act') or failed to comply with health, safety, housing or maintenance standards.

This application was heard in Barrie on January 19, 2011.

The Tenants and the Landlord attended the hearing.

Determinations:

1. The Landlord is obligated by section 20 of the Act to keep the rental unit in a good state of repair and fit for habitation. A rental unit that is infested with beg bugs is not in a good state of repair or fit for habitation.

2. Therefore, I find that the Landlord failed to meet the Landlord's obligations under section 20 of the Act.

3. The evidence presented did not establish that the Tenants' negligent or wilful conduct caused the bed bug infestation in the rental unit. Therefore, I find that the Landlord is responsible for the $819.25 exterminator cost.

4. Although the Tenants offered, by an email dated August 27, 2010, to split the cost of the exterminator bill, there was no dispute that the Landlord did not accept this offer. An agreement requires an offer and an acceptance. Since the offer was not accepted, there was no agreement between the parties which would prevent the Tenants from seeking the full amount of the exterminator bill.

5. The Tenants' request for $429.38 for the cost of a replacement mattress is refused. This cost was not claimed in the application. At the hearing, the Tenants agreed that the mattress was old and needed to be replaced regardless of the bed bugs.

It is ordered that:

1. The Landlord shall pay the Tenants $819.25, which is the reasonable out-of-pocket expenses the Tenants have incurred for an exterminator.

2. The Landlord shall also pay the Tenants $45.00 for the cost of filing the application.

3. The total amount the Landlord owes the Tenants is $864.25. The Landlord shall pay the Tenants the full amount owing by February 7, 2011.

4. If the Landlord does not pay the Tenants the full amount owing by February 7, 2011 the Landlord will owe interest. This will be simple interest calculated from February 8, 2011 at 3.00% annually on the outstanding balance.

5. If the Landlord does not pay the Tenants the full amount owing by February 7, 2011, the Tenants may recover this amount by deducting it from the rent commencing in March 2011 and continuing until the full amount has been recovered.

6. The Tenants have the right, at any time, to collect the full amount owing or any balance outstanding under this order.

2011 CanLII 5939 (ON LTB)

January 27, 2011
Date Issued

Karen Wallace
Member, Landlord and Tenant Board

Central-RO
3 Robert Speck Pkwy, Suite 520, 5th Floor
Mississauga Ontario L4Z2G5

Reasons for this order are attached.

If you have any questions about this order, call 416-645-8080

REASONS

Reasons to Order CET-09365-10 issued on January 27, 2011 by Karen Wallace.

2011 CanLII 5939 (ON LTB)

1. There was no dispute to the following: there was an infestation of bed bugs in the rental unit in August 2010; the parties agreed that a professional exterminator should be hired to eliminate the bugs; the exterminator remedied the problem by September 2010; the Tenants have paid the $819.25 exterminator bill.

2. The parties disagreed on who should be responsible for the exterminator bill. The Tenants argued it is the Landlord's responsibility.

3. The Landlord argued that the Tenants should be responsible for the bill.

4. The Landlord initially stated that this unit is shared student housing and the rental unit is not as clean as it should be. However, at the hearing, the Landlord conceded that a bed bug infestation is not caused by a lack of cleanliness.

5. At the hearing, the parties agreed that it is unclear where the bed bugs came from or how they infested the rental unit.

6. If the exterminator bill is the result of a maintenance issue, it is the Landlord's responsibility.

7. If the exterminator bill is the result of a damage issue, it is the Tenants' responsibility.

8. The Tenants' obligation, under section 34 of the *Residential Tenancies Act*, 2006 (the 'Act'), is to pay for undue damage to the rental unit that they wilfully or negligently caused.

9. The Landlord was not able to provide any evidence to suggest that the Tenants wilfully or negligently caused undue damage to the rental unit due to the presence of bed bugs.

10. Unless the Landlord can establish that the bed bugs resulted from the Tenants' wilful or negligent conduct, the extermination cost is the responsibility of the Landlord as a result of the obligations in section 20 of the Act.

11. Subsection 20(1) of the Act states: "A landlord is responsible for providing and maintaining a residential complex, including the rental units in it, in a good state of repair and fit for habitation and for complying with health, safety, housing and maintenance standards".

12. Subsection 20(1) of the Act does not contain an element of fault. In other words, the reasons for a landlord's breach of subsection 20(1) are not relevant. Regardless of the reasons why a landlord is unable to provide a rental unit in a good state of repair and fit

for habitation, if they are unable to do so, they are in breach of their obligation under the Act.

13. The Landlord’s maintenance obligations and the irrelevance of fault has been referred to in court cases and was upheld by the Divisional Court in *Offredi v. 751768 Ontario Ltd* [1994] O.J. No. 1204. In that case the Divisional Court stated: “The question of fault on the landlord’s part is not the issue... What the tenants claim is a breach of contract. The tenants were paying full rent for premises which the landlord was under an obligation… to keep in a good state of repair and fit for habitation. The landlord failed to do that. That is the basis for the claim…”

14. The irrelevance of fault was discussed by the Ontario Court of Justice (General Division) in the case of *35 Charles Street West v. Metropolitan Toronto Housing Authority* [1996] O.J. No. 5469. The Court stated that the no fault approach to a landlord’s obligations is consistent with the approach taken to a tenant’s obligation to pay rent. A tenant is required to pay rent regardless of whether or not they have the money to pay. It is no excuse for a tenant in arrears of rent to explain they have no money to pay rent due to circumstances beyond their control.

15. I agree that the Landlord did personally cause the bed bug infestation. There has been no wrong-doing by the Landlord. Bed bugs are simply becoming a more wide spread, common and frequent problem in our society. The Landlord, through no fault of his own, was unable to provide a rental unit that was in a good state of repair and fit for habitation. Since the Landlord was in breach of subsection 20(1) of the Act, the Tenants are entitled to the remedy requested, which reimbursement of the exterminator bill that they paid for.

2011 CanLII 5939 (ON LTB)

January 27, 2011
Date Issued

____
Karen Wallace
Member, Landlord and Tenant Board

Central-RO
3 Robert Speck Pkwy, Suite 520, 5th Floor
Mississauga Ontario L4Z2G5
