# Supplementary material for: Analysis of 44 Cases before the Landlord and Tenant Board Involving Bed Bug Infestations in Ontario, Canada: Focus on Adjudicator Decisions Based on Entomological/Pest Management Evidence and Accountability under the Residential Tenancy Act and Other Applicable Legislation
Source: Insects. 2011 Jul 19;2(3):343–53. doi: 10.3390/insects2030343 (PMC4553548; doi:10.3390/insects2030343)
Supplement: Supplementary File 1 [file insects-02-00343-s001.zip › supplementary-2/2011canlii5965.pdf]

Landlord and Tenant Board

Ontario

Order under Section 69
Residential Tenancies Act, 2006

File Number: TSL-04597-10

2011 CanLII 5965 (ON LTB)

RVE (the 'Landlord') applied for an order to terminate the tenancy and evict WT (the 'Tenant') because he, another occupant of the rental unit or someone he permitted in the residential complex has substantially interfered with the reasonable enjoyment or lawful right, privilege or interest of the Landlord or another tenant. The Landlord also claimed compensation for each day the Tenant remained in the unit after the termination date.

This application proceeded by way of a written hearing. The deadline for final submissions was January 5, 2011.

Determinations:

1. This application was filed with the Board on April 20, 2010. The Tenant had previously filed his own application with the Board on March 30, 2010. On September 1, 2010 I issued an interim order granting the Tenant’s request for a written hearing for both applications and setting out a process for the parties to follow for the filing of evidence and submissions.

2. The interim order required both parties to file their evidence in support of their own application on or before September 22, 2010. The Landlord met this deadline with respect to the Landlord’s application. The Tenant did not. As a result, I issued order TST-03827-10 on September 27, 2010 dismissing the Tenant’s application as abandoned.

3. After that order was issued, the Tenant started to file evidence and submissions with the Board with respect to his own claims. I directed those materials to be placed in this file for the Landlord’s application on the basis that they might be relevant to issues of relief from eviction under section 83 of the *Residential Tenancies Act, 2006* (the ‘Act’).

4. On March 22, 2010 the Landlord served on the Tenant a first notice of termination pursuant to subsection 64(1) of the Act. That section provides that a landlord may serve notice of termination on a tenant ”if the conduct of the tenant… is such that it substantially interferes with the reasonable enjoyment of the residential complex for all usual purposes by the landlord or another tenant or substantially interferes with another lawful right, privilege or interest of the landlord or another tenant”. The particulars on the notice of termination stated that on four occasions the Tenant had been notified that his unit was to be treated for bedbugs but the Tenant failed to properly prepare the unit for treatment. According to the notice, this resulted in bed bugs spreading from the Tenant’s unit to other areas of the residential complex.

5. Pursuant to subsection 64(3) of the Act the Tenant was given seven days to stop the behaviour complained of. Section 64(3) says: “The notice of termination under subsection (1) is void if the tenant, within seven days after receiving the notice, stops the conduct or activity or corrects the omission.” The conduct complained of in the notice of

termination was that the Tenant failed to prepare his unit adequately for scheduled pest control treatments.

6. On September 22, 2010 the Landlord filed an unsworn affidavit with the Board as its evidence in chief in support of its application. (A sworn copy was filed the following day.) It details how the Landlord attempted to treat the unit for bedbugs starting in August of 2009. It further states that treatment essentially failed repeatedly because the Tenant's unit was so cluttered and full that the pest control technician refused to treat the unit; but when prevailed upon by the Landlord did so, even though he was unable to fully access and treat the unit properly.

7. The affidavit contains no information or evidence whatsoever concerning what occurred during the seven day voiding period from March 22 to March 29, 2010. Rather the next incident referred to occurred on March 31, 2010. I have searched both parties' evidence as filed for any additional information concerning the voiding period and can find nothing further.

8. As a result, I can only conclude that no efforts were made to treat the Tenant's unit during the voiding period. Therefore it cannot be said that the Tenant failed to prepare his unit adequately for pest control treatment during the voiding period which is the behaviour complained of in the notice of treatment. As the only evidence before me would support the proposition that the Tenant did not repeat the behaviour complained of during the voiding period, I can only conclude the Tenant voided the notice. As the Board has no authority to evict a tenant based on a voided notice, the Landlord's application must be dismissed.

2011 CanLII 5965 (ON LTB)

It is ordered that:

1. The Landlord's application is dismissed.

January 6, 2011
Date Issued

____
Ruth Carey
Member, Landlord and Tenant Board

Toronto South-RO
79 St. Clair Avenue East, Suite 212, 2nd Floor
Toronto ON M4T1M6

If you have any questions about this order, call 416-645-8080 or toll free at 1-888-332-3234.
