# Supplementary material for: Analysis of 44 Cases before the Landlord and Tenant Board Involving Bed Bug Infestations in Ontario, Canada: Focus on Adjudicator Decisions Based on Entomological/Pest Management Evidence and Accountability under the Residential Tenancy Act and Other Applicable Legislation
Source: Insects. 2011 Jul 19;2(3):343–53. doi: 10.3390/insects2030343 (PMC4553548; doi:10.3390/insects2030343)
Supplement: Supplementary File 1 [file insects-02-00343-s001.zip › supplementary-2/APPENDIX.2 ANALYSIS.CASES.pdf]

NO	CASE	DATE	ADJUDICATOR	CLAIMANT	OUTCOME	LEGAL CLAIM	AWARD	CAUSE	DETAIL CLAIM	EVIDENCE	PREP. ISSUE	CONTROL ISSUE	RENT ARREARS	EXPERT	MISSING?	EVALUATION	APPRAISAL	SCORE	RATING (P/F)	EXPERT TYPE
1	37735	2008	E.FELLMAN	T	L	MAINTENANCE DEFICIENCY	0	ADJUDICATOR CONSIDERS LANDLORDS ACTIONS ADEQUATE, TENANT REFUSED SECOND TREATMENT CONSIDERED TO BE WITHIN STANDARDS	DAMAGES TO FUTON BY SPRAYING IT.	PUBLIC HEALTH LEAD SPECIALIST INSPECTED UNIT. DIDN'T FIND ANY EVIDENCE	0	1		LEAD PUBLIC HEALTH.. CONFLICT OF INTEREST.	NO DETAILS OF TIME SPENT TREATING UNIT.. NO MENTION OF ANY PREPARATION ISSUES..	THE FACT THAT THE LEAD SPECIALIST FROM TPH INSPECTED IS NOT PROOF THAT THERE IS NO INFESTATION. INAPPROPRIATE SPRAYING OF FUTON CREATED ANGST IN TENANT.. REFUSED SECOND TREATMENT. SHOULD HAVE BEEN REIMBURSED FOR THIS..	FLAWED DECISION			LPH
			**SCORE**					0	0	0				0	0	0		**0**	**F**	
2	79045	2009	C.LESLIE	T	T	INTERFERENCE WITH ENJOYMENT	RENT ABATEMENT, COST OF PESTICIDES, MOVING COSTS	LANDLORD IGNORED ISSUE WHEN TENANT DID NOT REQUEST SECOND TREATMENT.THEY HAD ADVISED TENANT TO "LET THEM KNOW WHEN READY", BUT NO OTHER FOLLOW-UP		TENANTS INITIALLY DID NOT ADVISE THE LANDLORD BUT TRIED TO HANDLE ALONE..	0	1		NO	NO EXPERT WITNESS TO ASSESS SITUATION	ON BALANCE, SLANTED IN FAVOUR OF TENANTS,	LANDLORD ACCOUNTABILITY WAS THE MAIN FOCUS HERE.. NOT UNREASONABLE, BUT TENANT IGNORANCE ACCEPTED,BUT NOT THAT OF LANDLORD		**A**	0
			**SCORE**					1	0	1				0	0	1		**3**	**P**	
3	86704	2009	C.MINGAY	T	L	MAINTENANCE DEFICIENCY . INTERFERENCE WITH ENJOYMENT	0	CONSIDERED HABITABLE AND REASONABLE ACTIONS BY LANDLORD	INFESTED AT TIME OF MOVE-IN	STRONG BUT NOT USED PROPERLY	0	1		LANDLORD PEST FIRM BUT TESTIMONY WAS INCORRECT FACTUALLY	TENANT DID NOT HAVE EXPERT	INCORRECT EVIDENCE BY FIRM'S TECHNICIAN SAYS DO NOT SPREAD BETWEEN UNITS, BUT MUST BE CARRIED.. ADJUDICATOR CONSIDERED THIS COMMON AND THAT THE LANDLORD DID HIS BEST.. VERY BAD DECISION	FLAWED DECISION		**F**	LPC
			**SCORE**					-1	-1	-1				-1	0	-1		**-5**	**F**	
4	13731	2010	J.VAN DELFT	T	T	HARASSMENT., BED BUG CLAIM WITHDRAWN	UNRELATED TO BED BUGS									PILED ON OTHER CLAIMS, WITHDRAWN				0
REVIEW			**SCORE**					0	0	0				0	0	0		**0**	**N**	
5	13735	2010	G.BITTU	T	L	MAINTENANCE DEFICIENCY, ENJOYMENT OF PROPERTY	0	CASE NOT PROVEN. INSPECTIONS BY EXPERT	7200	Nothing found in spite of inspections, but was treated at least 7 times. Preparation issues, but item about "moving mattress" is flawed.	1	1		YES PEST CONTROL FIRM EXPERT DM	NO DETAILS OF TIME SPENT TREATING UNIT. TREATING SO MANY TIMES WITHOUT EVIDENCE IS ILLEGAL AND POOR PRACTICE	LANDLORD FIRM SEEMED MORE CREDIBLE THAN TENANT, BUT FLAWS IN HOW THIS WAS HANDLED. HOW MANY TIMES WAS UNIT PROPERLY PREPARED?	NOT A GOOD DECISION AS EVIDENCE WAS FLAWED. LANDLORD REPORT MORE CREDIBLE.			LPC
			**SCORE**					0	0	-1				1	-1	1		**0**	**F**	
6	13782	2010	J.McMASTER	T	L	maintenance	0	CASE NOT PROVEN. INSPECTIONS BY EXPERT	no amount mentioned	nothing found in spite of inspections, checked adjacent units				YES PEST CONTROL FIRM EXPERT DM	might have been case of Delusory Parasitosis.. But with bites at move-in.. Curious for sure. PROOF NOT DEFINITIVE BUT SUBSTANTIAL	LANDLORD AND THEIR FIRM WERE CREDIBLE.. PROOF IS NOT DEFINITIVE.	FAIR DECISION	**7**		LPC
			**SCORE**					0	0	-1				1	-1	1		**0**	**F**	
7	13809	2010	V.CHING	T	L	MAINTENANCE	0	NO OTHER REPORTED INFESTATIONS	18500	LANDLORD ACTED PROMPTLY . TENANT DID NOT PREPARE. NO OTHER REQUESTS	1			PEST CONTROL FIRM FOR LANDLORD	NO SOUND BASIS FOR REPORT OF NO OTHER INFESTATIONS. ADJACENT UNITS NOT CHECKED	TENANT CLAIMS WERE VAGUE AND NOT CONSIDERED VALID. SEEM TO BE DEFENSE AGAINST ARREARS	FAIR DECISION			LPC
			**SCORE**					0		1				0	-1	1		**1**	**P**	
8	18631	2010	M.SOO	L	L	RENT ARREARS	RENT ARREARS.	COUNTERCLAIM BY TENANT		LANDLORD ACTED ON REPORT. TENANT DID NOT REQUEST SERVICE	0			NO		TENANT CLAIMS WERE VAGUE AND NOT CONSIDERED VALID. SEEM TO BE DEFENSE AGAINST ARREARS	GOOD DECISION			0
			**SCORE**					0		1				0	0	1		**2**	**P**	
9	37684	2010	S.COLLINS	T	L	MAINTENANCE INTERFERENCE WITH REASONABLE ENJOYMENT	0	NO OTHER REPORTED INFESTATIONS	700	LANDLORD ACTED ON REPORT.	0			NO	THE FACT THAT THERE HAVE NOT BEEN OTHER REQUESTS DOES NOT MEAN THAT THERE IS NO OTHER INFESTATION.. NO REPORT OF ADJACENT UNIT INFESTATIONS	LANDLORD WAS CONSIDERING CHARGING TENANT COSTS OF TREATMENT, BUT THIS WAS REJECTED	GOOD DECISION			0
			**SCORE**					1										**1**	**P**	
10	37783	2010	G.SAVOIE	T	T	MAINTENANCE DEFICIENCY . INTERFERENCE WITH ENJOYMENT. CAUSED TENANT TERMINATION OF LEASE	2160	REPLACEMENT OF DISPOSED FURNITURE.ABATEMENT.COSTS,NO TERMINATION AS NOT CAUSED BY LANDLORD?	INADEQUATE TREATMENT MEASURES CLAIMED (REPEATED TREATMENTS UNTIL SUCCESS). EXTERMINATOR ADVISED TO THROW OUT ITEMS	ADJUDICATOR NOTED A LOT OF DETAILS OF WHAT HAPPENED	0	1		BAD ADVISE BY PEST CONTROL FIRM WITHOUT CONSULTING LANDLORD.. TO THROW OUT ITEMS	FLEAS REPORTED BUT NO MENTION OF ANY PETS	ADJUDICATOR GAVE THIS A LOT OF REFLECTION.. BUT EVIDENCE THAT SECOND PEST CONTROL FIRM SAID IT WAS FREE OF BED BUGS WAS FLAWED. REFUSAL TO ALLOW TERMINATION OF TENANCY WAS FLAWED.. BED BUGS WERE PRESENT AT MOVE IN...			**F**	LPC
								0	-1	1				-1	-1	-1		**-3**	**F**	
11	44231	2010	G.TAYLOR	T	T	INTERFERENCE ENJOYMENT	RENT ABATEMENT AND LOSSES	INTERFERENCE ENJOYMENT	REBATE DAMAGES	WEAK				NO	DETAILS TREATMENT AND PREP					0
			**SCORE**					1	1	0				0	-1	SEEMS FAIR CONSIDERING EVIDENCE WAS		**1**	**P**	
12	44277	2010	J.McMASTER	L	L T	RENT ARREARS	ARREARS PLUS COSTS. 20%R.ABATEMENT	DELAY IN TREATMENT	DELAY IN TREATMENT	2-3 WEEKS FROM REPORTED	1			NO		REASONABLE	GOOD DECISION			0
			**SCORE**					1	1	1				0	0	1		**4**	**P**	
13	48718	2010	S.COLLINS	T	T	PREVIOUSLY INFESTED, MAINTENANCE ISSUES, DAMAGES FROM MICE, DELAY OF TREATMENT	25%R.ABATEMEN $1300)T,DAMAGES, (870) LEASE TERMINATED	DELAY IN TREATMENT	25%R.ABATEMENT,DAMAGES(Mice), LEASE TERMINATED	DELAY IN TREATMENT FROM NOV TO JAN	0	1		NO	NO	POOR RESPONSE TO MICE AND BED BUGS, CLAIMS ADJUSTED	GOOD DECISION, TOUGH ON NEW LANDLORD			0
			**SCORE**					1	1	1				0	0	1		**4**	**P**	
14	48769	2010	L.STEVENS	T	L	SUSPECTED BITES	0	Possible Delusory Parasitosis	BITES.. BED BUGS?	NO BED BUGS FOUND BY HEALTH INSPECTOR, OR PEST CONTROL FIRM. SUGGESTION OF BODY LICE NONSENSE				THE EVIDENCE SUGGESTS THAT NEITHER THE HEALTH INSPECTOR OR PEST CONTROL OPERATOR WERE COMPETENT	PROPER MONITORING INSPECTION. PROPER I.D. OF FOUND INSECTS. ADDRESSING SUSPECTED D.P. WITH HEALTH UNIT	INSECTS SHOULD HAVE BEEN PROPERLY I.D. USE OF CLIMBUPS TO VALIDATE NO BED BUGS. GLUE TRAPS	ADJUDICATOR ACTED AS WELL AS COULD BE EXPECTED WITHOUT UNDERSTANDING THE REAL ISSUE .. DELUSORY PARASITOSIS			LPC LPH
			**SCORE**					0	0	-1				-1	-1	-1		**-4**	**F**	
15	48882	2010	E.SANGMUAH	L	LT	RENT ARREARS	RENT ARREARS LESS 20% ABATEMENT FOR PERIOD BETWEEN REPORTING AND TREATMENT	LANDLORD DELAYED TREATMENT. TENANT DID NOT CO-OPERATE FULLY	ARREARS VS BED BUGS	ISSUE OF NOTICE OF TREATMENT. PREPARATION INSTRUCTIONS.	1	1	1	NO	DETAILS OF PREPARATION	FAIR ASSESSMENT.. DID NOT REIMBURSE TENANT FOR CLAIMS OF DAMAGES TO MATTRESS OR BEDDING AS NO PROPER COST EVIDENCE SHOWN	FAIR DECISION			0
			**SCORE**					0	0	1				0	1	1		**3**	**P**	
16	58936	2010	B.HOMENIUK	T	T	BED BUGS. LANDLORD FAILED TO ACT AND IN BREACH OF MAINTENANCE	50% RENT REBATE FROM FIRST REPORT OF INFESTATION	LANDLORD REFUSED TO ACCEPT UNIT WAS INFESTED. TENANT HARASSED BY LANDLORD AND STAFF.	FAILURE OF PROVIDING SERVICES	TENANT FOUND BED BUG. LANDLORD HIRED FIRM TO INSPECT..THEY DID NOT FIND ANY. TREATMENT REFUSED..TENANT FOUND MORE INFESTATION. TENANT NOTIFIED OTHERS. LANDLORD ASKED TENANT TO MOVE OUT. TENANT HAPPY TO LEAVE	0	1		PEST CONTROL FIRM INCOMPETENT. IF TENANT FOUND ONE, ANDWAS CONFIRMED, THEN SUFFICIENT EVIDENCE.	NO	OVERALL FAIR JUDGEMENT.REFUSAL TO PAY FOR LOTION, STEAMER AND MATTRESS COVERS SEEMS UNFAIR AS LANDLORD REFUSED TO TREAT AT ALL	GENERALLY FAIR, EXCEPT FOR NOT PAYING FOR THE ITEMS TENANT BOUGHT TO DO SELF TREATMENT. THE FACT ITEMS WERE RESUSABLE IS IRRELEVANTIN MY VIEW.		**A**	LPC
			**SCORE**					1	1	1				-1	0	-1		**1**	**P**	
17	58958	2010	V.CHING	T	T	RENT ABATEMENT, CLEANING	AS REQUESTED	DELAY IN TREATMENT, LACK OF CONCERN		3 WEEK DELAY IN ARRANGING TREATMENT		1		NO	SOME FOLLOW-UP FOR SITE	FAIR JUDGEMENT	FAIR DECISION			0
			**SCORE**					1	0	1				0	1	1		**4**	**P**	
18	65536	2010	J.McMASTER	T	T	RENT ABATEMENT, DUMPING OF FURNITURE, LAUNDRY, INSPECTOR	50% RENT ABATEMENT FROM FIRST REPORTED, COSTS OF LAUNDRY,INSPECTOR TO CHECK	SOME DELAY INTREATMENT, ASKED TENANT TO PAY HALF		NO IDENTIFICATION OF INSPECTOR, QUALIFICATIONS, SCOPE OF INSPECTION	1			YES, BUT NOT IDENTIFIED	WERE THERE OTHER INFESTATIONS IN THE SITE, NO APPROPRIATE RECOMMENDATIONS TO PROTECT OTHERS	MOSTLY FAIR JUDGEMENT	LACK OF CONFIRMED EXPERTISE.NO RECOMMENDED FOLLOW-UP TO PROTECT OTHERS			YES BUT UNKNOWN TYPE
			**SCORE**					1	0	0				0	0	1		**2**	**P**	
19	65642	2010	C.LESLIE	T	L	HARASSMENT. WITHHOLDING OF SERVICE, BED BUGS	0	CONFLICT BETWEEN TENANT AND LANDLORD		TENANT EVIDENCE NOT SUBSTANTIATED, LANDLORD COMPANY HAD LEGAL REP PLUS 4 PEOPLE PRESENT	0			NO	NO	OBVIOUS BAD RELATIONS BETWEEN LANDLORD'S AGENTS AND TENANT	BED BUGS NOT THE REAL FOCUS			0
			**SCORE**					1	0	1				0	0	0		**2**	**P**	

20	70677	2010	L.HORTON	T	L	RE-OPENED A MEDIATED AGREEMENT	GIVE ANDTAKE. TENANT LOST	COMPLAINTS ABOUT VARIOUS ISSUES, BED BUGS ONLY A SIDE ISSUE	MAINTENANCE AND INTERFERENCE	ISSUES OF LAUNDRY CARD. T.LOST, PARKING. T.LOST, HANDLING OF GARBAGE, T.LOST, FLOODING, T.WON				NOT REQUIRED		BED BUGS ONLY A SIDE ISSUE	BED BUGS ADDED AS A SIDE ISSUE			0
			**SCORE**					1	1	0				0	0	1		**3**	**P**	
21	76098	2010	K.WALLACE	T	T	LANDLORD FAILED TO MAINTAIN PROPERLY, PRIOR INFESTATION, DAMAGES TO MATTRESS . LEFT BEHIND	50% RENT ABATEMENT, REPLACEMENT OF MATTRESS	LANDLORD KNEW UNIT WAS INFESTED, BUT CLAIMED NO RESPONSIBLITY TO INFORM AS WAS TREATED	DID NOT AWARD 100% ABATEMENT AS TENANT STILL LIVED IN PREMISES	DETAILED EVIDENCE PRESENTED.. LANDLORD CITED OTHER CASES, BUT ADJUDICATOR NOTED UNDER NO OBLIGATION TO FOLLOW THESE AND IN THESE CASES, LANDLORD NOT IN VIOLATION OF MAINTENANCE OBLIGATIONS , MAY HAVE BEEN TENANT		1		LETTERS FROM PEST CONTROL COMPANY INDICATING PROBLEM NOT SOLVED AND OTHER UNITS (ADJACENT) ALSO INFESTED		CONSIDERABLE AMOUNT OF EVIDENCE PRESENTED.. ADJUDICATOR NOTED THAT EVEN IF TENANT BLAMELESS IN BEING UNABLE TO PAY RENT,ARE STILL OBLIGED TO PAY IT, AND IN SIMILAR CONTEXT,EVEN IF LANDLORD IS BLAMELESS IN CAUSE OF INFESTATION, THEY ARE STILL RESPONSIBLE TO PROVIDE AN UNINFESTED UNIT TO NEW TENANT, AND TO SOLVE INFESTATION IN UNITS,.	ADJUDICATOR VERY KNOWLEDGEABLE, AND INTERPRETATED EVIDENCE VERY WELL IN RELATION TO CASE AND THE ACT,		**A**	LPC
			**SCORE**					1	1	1				0	0	1		**4**	**P**	
22	76210	2010	B.WALLACE	T	L	FAILURE OF MAINTENANCE, LOSS OF ENJOYMENT DUE TO BED BUGS	0	NO DIRECT EVIDENCE OF BED BUGS AFTER TREATMENT IN 2010	TENANT CLAIMS TREATMENT IN 2009 DIDN'T WORK , WALLS SHOULD HAVE BEEN DUSTED	SEEMS ONLY ONE TREATMENT EACH TIME.. 7 UNITS INFESTED IN 2010,THEN NO REPORTS	0	1		ONLY PEST CONTROL FIRM.. WORKING FOR LANDLORD.	PRESUMPTION THAT PC INSPECTIONS WOULD ALWAYS VERIFY NO INFESTATION	PROBLEM RECURRED IN 7 UNITS BUT A LONG TIME SINCE ORIGINAL INFESTATION. WAS THE PROBLEM ACTUALLY SOLVED ORIGINALLY? REPORT NO OTHER COMPLAINTS SEEMS REMARKABLE CONSIDERING ONLY ONE TREATMENT EACH TIME	TENANT FIRST THOUGHT SPIDERS, AND SUPER PROVIDED SPRAY.. NOT A SOLUTION, AND NOT QUALIFIED .. THEN SOME WEEKS UNTIL VERIFIED BED BUGS			LPC
			**SCORE**					0	0	-1				0	0	-1		**-2**	**F**	
23	76235	2010	E.FELLMAN	L	LT	RENT ARREARS BY L. TENANT NOTED MAINTENANCE ISSUES, RECOMMENDATION BY HEALTH DEPT FOR SEALING TO PREVENT BED BUGS AND ROACHES DUE TO CHILD'S ASTHMA	15% ABATEMENT TO TENANT. NOTICE OF EVICTION UNLESS RENT PAID	AS NOTED		CAREFULLY DETAILED.	0	1	1	NOT REQUIRED	NO	FAIR DECISION	BED BUGS AND MAINTENANCE AS ISSUES AGAINST RENT ARREARS.. NOT MAIN ISSUE			0
			**SCORE**					1	0	1				0	0	1		**3**	**P**	
24	79677	2010	L.HORTON	T	LT	VARIOUS ISSUES. RENT ARREARS BED BUGS INCIDENTAL.SECOND CLAIM ONSAME ISSUES	SOME ABATEMENT. SCHEDULE TO REPAY RENT	BED BUG ISSUE NOT ACCEPTED. RELATED TO HANDLING OF EXTERIOR GARBAGE DISPOSAL	VARIOUS	NOT CONSIDERED VALID IN RELATION TO BED BUGS				NO	NO	FAIR DECISION	BED BUGS AND MAINTENANCE AS ISSUES AGAINST RENT ARREARS.. NOT MAIN ISSUE			0
			**SCORE**					1	0	1				0	0	1		**3**	**P**	
25	54	2007	J.NOLAN	T	L	BED BUGS. REASONABLE ENJOYMENT AND SUPPLYING NECESSARY SERVICE- SMALL AMOUNT	0	LANDLORD PROVIDED SPRAY AND DUST. ADJUDICATOR CONSIDERED THIS REASONABLE. OFFERED ANOTHER ROOM. TENANT REFUSED	TO FUMIGATE POSSESSIONS, AND FOR ABATEMENT WHEN OUT OF ROOM	OVERWHELMINGLY IN SUPPORT OF TENANT.BROUGHT OTHER TENANTS..KNOWN INFESTATION.	0	1		HEALTH INSPECTOR SAID COULD NOT HELP AS NUISANCE, NOT HEALTH ISSUE		VERY POOR DECISION BY EVERY MEASURE . NO SUGGESTION OF PROFESSIONAL TREATMENT OF SITE. NOTE DECISION WAS IN 2007	SEEMS NEITHER HEALTH INSPECTOR NOR THE ADJUDICATOR WERE COMPETENT IN THIS DECISION			PH
			**SCORE**					-1	-1	-1				-1	0	-1		**-5**	**F**	
26	1714	2008	O.LUFTIG	T	TL	BED BUGS REASONABLE ENJOYMENT, MAINTENANCE	ABATEMENT FOR RENT AND DAMAGES	BED BUGS. INITIALLY IN UNIT.. NOT REPORTED TO TENANT. FAILURE TO ACHIEVE CONTROL WITH PROFESSIONAL SERVICE	FOR RENT. (TERMINATE TENANCY), FOR COST OF SOME INFESTED ITEMS	CLEARLY IN SUPPORT OF TENANT		1		NO		ADJUDICATOR CAREFULLY CONSIDERED THE TERMS OF THE LEASE AND REVIEWED EVERY DETAIL OF CLAIM WITH CARE	VERY FAIR. ONLY QUESTION IS KNOWLEDGE OF LANDLORD THAT THE UNIT WAS INFESTED.. NOT CLEAR			0
			**SCORE**					1	1	1				0	0	1		**4**	**P**	
27	1497	2008	E.SANGMUAH	T	TL	BED BUGS	COST OF TREATMENT	APPEARS TENANT BROUGHT BED BUGS INTO UNIT FROM DISCARDED HEADBOARD	FOR TENANT TREATMENT COSTS.BY HIRING FIRM. FAILURE TO CORRECT A MAINTENANCE ISSUE OF DAMAGES	DETAILED EVIDENCE PRESENTED. HEADBOARD FROM INFESTED UNIT. TENANT'S COMPANY USED PRODUCT NOT REGISTERED FOR BED BUGS.. AMATEURISH TREATMENT	0			NO		ADJUDICATOR REVIEWED EVIDENCE WITH CARE . REBATE OF 250 FOR LANDLORD FAILURE TO RESPOND TO MAINTENANCE ISSUE. NOTHING AWARDED FOR BED BUG ISSUE AS LANDLORD HAD PROVIDED SERVICE, AND TENANT BROUGHT IN INFESTATION, TENANT DID CONTROL ONOWN	VERY FAIR. NOTHING AWARDED FOR BED BUG ISSUE AS LANDLORD HAD PROVIDED SERVICE, AND TENANT BROUGHT IN INFESTATION, TENANT DID CONTROL ONOWN			0
			**SCORE**					1	1	1				0	0	1		**4**	**P**	
28	19858	2009	J.TAYLOR	LT	T	BED BUGS REASONABLE ENJOYMENT AND HARRASSMENT .TENANT; RENT ARREARS, LANDLORD	ABATEMENT FOR HARRASSMEN T,AND COSTS OF TREATMENT BY TENANT	TREATMENT BY CONTRACTOR FAILED.	AS NOTED	LANDLORD'S WITNESS (ANOTHER TENANT) NOTED NEED FOR REPEATEDTREATMENTS THAT SUPPORTED TENANT'SCLAIM OF FAILURE OF CONTROL. CONSIDERED LANDLORD FAILED TO LIMIT SPREAD IN BUILDING	0	1		NO	NO REAL EVALUATION OF STATUS IN THE BUILDING. LANDLORD EVIDENCE SEEMED WEAK..	SOME DISPUTE ABOUT VALIDITY OF RECEIPT,, BUT ADJUDICATOR HAD NO REASON TO QUESTION THIS. FAIR DECISION OVERALL.	FAIR DECISION. BUT NOTHING TO RELATED TO ONGOING PROBLEMS IN BUILDING.. MISSED OPPORTUNITY NOT ONLY THIS CASE		**A**	0
			**SCORE**					1	1	1				0	0	1		**4**	**P**	
29	67622	2010	E.SANGMUAH	T	T	INTERFERENCE WITH ENJOYMENT. FAILURE OF MAINTENANCE	RENT ABATEMENT, HOTEL, COSTS OF SOME MATERIALS	LANDLORD PEST CONTROL CONTRACTOR FAILED TOCONTROL AFTER THREE TREATMENTS, CONFIRMEDBY HEALTH DEPARTMENT. INCOMPETENCY OF CONTRACTOR. INADEQUATE ACTIONS BY LANDLORD IN BUILDING IN GENERAL	AS NOTED	TENANT DOCUMENTATION AND HEALTH DEPARTMENT INSPECTOR	0	1		HEALTH INSPECTOR	ANY FOLLOW UP ACTIONS BY HEALTH INSPECTOR.. NOT APPARENT OR PART OF THIS... BUT AN IMPORTANT ELEMENT TO HELP REDUCE ISSUES.	FAIR ADJUDICATION. DID NOT GRANT TENANT ALL REQUESTS (TIME AWAY FROM WORK DUE TO BITES.. NOT VERIFIED), CLAIM FOR DAMAGED ITEMS (COULD HAVE BEEN TREATED), RENT ABATEMENT VARIED DUE TO TENANT NOT INFORMING LANDLORD OF INTENTION TO VACATE, THEREBY DELAYING HIS ABILITY TO TREAT UNIT	FAIR DECISION. CAREFULLY EVALUATED BY ADJUDICATOR			TPH
			**SCORE**					1	1	1				1	-1	1		**4**	**P**	
30	78053	2009	K.BUGBY	TL	0	MAINTENANCE . T DAMAGES BY TENANT. L	NIL	ADJUDICATOR DISMISSED BOTH CLAIMS. 1. TENANT CLAIM, AS LANDLORD DID TAKE REASONABLE ACTION IN SPITE OF ASKING TENANT TO SIGN AGREEMENT TO PAY FOR TREATMENT. DISMISSED LANDLORD CLAIM FOR DAMAGES (BED BUGS) AS DAMAGES NOT DEFINED IN THIS WAY, BUT LANDLORD FREE TO SUE TENANT FOR THE COSTS IF THEY WISH.	LANDLORD ASKED TENANT TO AGREE TO PAY FOR SERVICES AS NO OTHER INFESTATION NEAR HER UNIT, AND LIKELY BROUGHT IN. TENANT CLAIMED FOR RENT ABATEMENT AND DAMAGES	BASED ON EVIDENCE, NOT A PREXISTING INFESTATION, AND APPARENTLY NOT RELATED TO ADJACENT UNITS.				NO	IN THIS CASE,LANDLORD IS OBLIGATED TO TREAT TO PROTECT OTHERS, BUT PROOF THAT IT IS TENANT'S FAULT ? PERHAPS...	FAIR DECISION OVERALL. ON WHAT BASIS WAS IT DETERMINED THAT ADJACENT UNITS ARE NOT INFESTED? WORD OF LANDLORD? PERHAPS	FAIR DECISION OVERALL.. JUST NEEDED MORE FACTUAL EVIDENCE THAT ADJACENT UNITS NOT INFESTED			0
			**SCORE**					1	-1	1				0	0	1		**2**	**P**	
31	78485	2009	B.GEORGE	T	0	FAILURE OF MAINTENANCE (BED BUGS)	NIL	SITE INFESTED,BUT TENANT ADVISED IN ADVANCE..MAJOR INFESTATION. CONSIDERABLE EFFORTS TO TREAT UNIT. TENANT FAILED TOCO- OPERATE IN MANY ASPECTS. SIGNED AGREEMENT TO ALLOW DISPOSAL AS PART OF PREPARATION. RECEIVED ONTARIO WORKS GRANT	TENANT CLAIMING FAILURE OF CONTROL AND OF DAMAGES AND LOSSES,	107 UNITS INFESTED.. TENANT REQUESTING ALL UNITS IN COMPLEX BE TREATED. (350 UNITS). TENANT WANTED CARPETS REMOVED. LANDLORD COST OF TREATING HALLWAYS IS EXTREMELY EXAGGERATED UNLESS THIS INVOLVES MAJOR RENOVATION.	1	1	0	ONLY EXPERT IS FROM TREATMENT FIRM WHO APPARENTLY ALSO DO PREPARATION. UNIT NOT IDENTIFIED AS CLUTTERED OR HOARDING CASE. CONFLICTS IN TERMS OF POSSESSIONS	SITE HAS MAJOR INFESTATION... ABOUT 30% OF UNITS... REACHING TIPPING POINT.. HOW MANY UNITS ADJACENT TO INFESTED UNITS	COMPLEX CASE.. ADJUDICATOR TOOK BEST JUDGEMENT UNDER CIRCUMSTANCES.., PERHAPS SOME SUGGESTIONS OF MEDIATION MIGHT HAVE BEEN USEFUL RATHER THAN SIMPLE DISMISSAL.	DECISION REALLY DOES NOT MOVE THE SITUATION FORWARD.. ADJUDICATOR COULD MAKE SUGGESTIONS TO FORWARD THE SITUATION TO BENEFIT OF ALL.			LPC
			**SCORE**					1	1	1				-1	-1	0		**1**	**P**	

32	79941	2009	C.KING	TL	TL	RENT ARREARS. L. MAINTENANCE.T	OUTSTANDING RENT. MAINTENANCE ABATEMENT OFFSET AGAINSTCOST FORAPPLICATION	UNIT INFESTED BUT TREATED.. LOW LEVEL OF INFESTATION	AS NOTED	HEALTH INSPECTOR INDICATED LOW LEVEL INFESTATION DUE TO LITTLE EVIDENCE FOUND. DOCTOR SPOKE OF HOW THIS CAN STRESS PEOPLE. (CAN BE FIXATED ON THIS. COMMENT .sb)	0	1	1	HEALTH INSPECTOR	MORE DIRECT PROOF OF LOW OR NO INFESTATION.	OVERALL FAIR..	EVALUATION BY VISUAL INDICATED LOW OR NO.. BUT NEED BETTER EVALUATION			LPH
			**SCORE**					1	1	1				1	-1	1		**4**	**P**	
33	93933	2010	J. BERKOVITS	L	TL	RENT ARREARS VS BED BUG ISSUE	ZERO .ONE OFFSET OTHER	UNIT INFESTED PRIOR TO MOVE IN	AS NOTED	LANDLORD TREATING UNIT. ILLEGAL. TENANT PROVIDED PHOTOS OF BITES. ADJUDICATOR DID NOT ACCEPT DOCTOR NOTE FULLY	0	YES	YES	NO	ADJUDICATOR UNAWARE THAT LANDLORD IS NOT ALLOWED TO TREAT UNIT	OVERALL SEEMS FAIR HOWEVER, ADJUDICATOR NOT KNOWLEDGEABLE OF VIOLATIONS	JUDGEMENT FAIR THOUGH ADJUDICATOR FAILED IN SOME AREAS			
			**SCORE**					1	1	1				0	-1	0		**2**	**P**	
34	13413	2011	J. ROZEHNAL	T	L	FAILURE TO MAINTAIN/ "ILLEGAL" RENT	0	THAT LANDLORD TOOK REASONABLE ACTION/ DID NOT CONSIDER CLAIMS OF LOSS.. NO DETAILS	AS NOTED. NO SPECIFICS OF LOSS OR OF PROOFS IN DECISION	TENANTS SAY WERE BITTEN AT START BUT DIDN'T KNOW FOR 4-5 MONTHS /REPORTED , WERE TREATED, NOTHING MUCH IN WRITING/ REFUSED BED BUG DETECTION DOG	NO	TENANT REFUSED FURTHER TREATMENT DUE TO CONCERNS OF HEALTH OF CHILD	NO	NO	TENANTS DID NOT APPEAR TO BE WELL ADVISED / NO ONE'S FAULT UNDER CURRENT LEGISLATION	IN CIRCUMSTANCES SEEMS LIKE A FAIR ADJUDICATION, BUT QUESTION OF WHETHER UNIT WAS INFESTED AT START. ON THE EVIDENCE SEEMS LANDLORD DIDN'T KNOW. SOME CONFLICT OF WHO IS TELLING TRUTH ABOUT WHO SAID WHAT AND WHEN. SHOULD HAVE BEEN IN WRITING.	NOTWITHSTANDING THE LIKELIHOOD OF UNIT BEING PREVIOUSLY INFESTED, ADJUDICATOR DID BEST THAT COULD BE DONE ON THE EVIDENCE.			
			**SCORE**					1	1	1				0	0	1		**4**	**P**	
35	5939	2011	K. WALLACE	T	T	FAILURE TO MAINTAIN	COST OF TREATMENT	ADJUDICATOR TOOK POSITION THAT IT IS THE LANDLORD'S OBLIGATION EVEN IF NOT THEIR FAULT. NO EVIDENCE THAT IT WAS TENANT'S FAULT (STUDENT HOUSING)	LANDLORD WANTED TENANT TO PAY FOR TREATMENT.. TENANT CONSIDERED THAT LANDLORD SHOULD PAY	TENANT ACTUALLY OFFERED TO SPLIT THE COSTS 50/50 BUT AS LANDLORD REFUSED, THIS WAS NOT CONSIDERED A BINDING AGREEMENT, AND LANDLORD HAD TO PAY THE FULL AMOUNT	NO	NO	NO	NO	NO	THE COST OF TREATMENT WAS CONSIDERABLE $819.25 .. ADJUDICATOR WOULD NOTREQUIRE LANDLORD TO PAY FOR MATTRESS REPLACEMENT AS IT WAS OLD AND NEEDED REPLACEMENT IN ANY CASE.	FAIR DECISION.BUT HIGHLIGHTS NEED FOR EDUCATION, AS THE ISSUE OF FAULT WAS HIGH IN THIS CASE.			
			**SCORE**					1	1	1				0	0	1		**4**	**P**	
36	26964	2011	L. BOURGON	L	L	RENT ARREARS	RENT AND COSTS	LANDLORD TOOK REASONABLE STEPS TO TRY TO TREAT FOR BED BUGS	TENANT REFUSED ENTRY. WANTED HEAT TREATMENT. LANDLORD REFUSED DUE TO COSTS. REFUSED TO ALLOW TECHNICIAN DUE TO PESTICIDES ON CLOTHES	LANDLORD CLAIMED THAT HEALTH DEPARTMENT RECOMMENDS SPRAYING AS STANDARD TREATMENT. DID PAY TENANT TO RENT A STEAMER. NOT CLEAR WHAT KIND	NO	YES	YES	NO	AN ALTERNATIVE COURSE OF TREATMENT , OR SHARING COSTS OF HEAT TREATMENT OR OF MEDICAL REASON FOR AVOIDING PESTICIDE	UNDER THE CIRCUMSTANCES.. SEEMS LIKE A FAIR EVALUATION BY ADJUDICATOR, NOT REALIZING THAT THERE COULD BE GROUNDS FOR ACCOMMODATION	FAIR ADJUDICATION UNDER THE CIRCUMSTANCES			
			**SCORE**					1	1	1				0	-1	1		**3**	**P**	
37	13481	2011	E. FUFFA	T	T	FAILURE TO MAINTAIN	COST OF FILING	LANDLORD DID NOT SOLVE PROBLEM	TENANT DID MAKE CLAIMS OF LOSS, BUT NOT IN EVIDENCE.	ADJUDICATOR REPORT DOES NOT OUTLINE DETAILS. BUT JUST SAYS THAT CLAIMS ARE NOT JUSTIFIED. TENANT CLAIM OF NO RESPONSE BY LANDLORD SINCE 2008 CONSIDERED AN EXAGGERATION BUT NO REASONS WHY.	NO	YES	IN TRUST TO LTB	NO	UNLIKE MOST ADJUDICATORS, DID NOT OUTLINE DETAILS OF REASONS FOR SOME OF THE DECISIONS	ORDER TO CORRECT PROBLEM, BUT THERE IS REALLY VERY LITTLE EVIDENCE PRESENTED ON THE SITUATION.	SEEMS WEAK OTHER THAN ORDER FOR LANDLORD TO CORRECT THE PROBLEM, BUT EVIDENCE IS MISSING.			
			**SCORE**					1	-1	-1				0	-1	-1		**-3**	**F**	
38	13438	2011	M.SOO	L	L	RENT ARREARS	RENT AND COSTS	LANDLORD RESPONDED REASONABLY	LANDLORD TREATED UNIT WITH HEAT TREATMENT AND PROVIDED ABATEMENT FOR THREE MONTHS	TENANT WANTED RENT ABATEMENT FOR ENTIRE TIME UNIT WAS INFESTED AND DAMAGES TO TWO TELEVISIONS, BUT ADMITTED THEY STILL WORKED AND WERE OLD	NO	CLAIMED UNIT INFESTED AT MOVE IN	YES	NO	NO	LANDLORD TOOK CONSIDERABLE STEPS TO TREAT UNIT.. ISSUE OF INFESTATION AT MOVE IN WAS REGRETTABLE BUT LANDLORD PAID ABATEMENT	FAIR EVALUATION			
			**SCORE**					1	1	1				0	0	1		**4**	**P**	
39	27370	2011	K. WALLACE	T	L	"ILLEGAL" RENT	0	REQUESTED ABATEMENT BUT DID NOT FILE CORRECTLY	BED BUGS IN UNIT	ADJUDICATOR FOLLOWING PROCEDURES.... BUT NOT ADDRESSING BED BUG ISSUE AT ALL	NO	YES	NO	NO	NO	DISMISSED ON A TECHNICALITY	TOO BAD ADJUDICATOR DIDN'T FACILITATE CORRECT T6 MAINTENANCE APPLICATION			
			**SCORE**					1	-1	0				0	0	-1		**-1**	**F**	
40	34410	2011	L. BOURGON	LT	L	LANDLORD. RENT ARREARS, TENANT. MAINTENANCE	RENT AND COSTS	TENANT UNCOOPERATIVE AND DID NOT FOLLOW ORDERS OF LTB	TENANT DID NOT PAY RENT ALTHOUGH ORDERED TO DO SO. TENANT LODGED MAINTENANCE CLAIM AFTER LANDLORD CLAIM	REPORT BY HEALTH DEPARTMENT PROVIDED BY TENANT INDICATED UNIT HAD BEEN TREATED AND TENANT ADVISED TO ASK FOR FOLLOW UP.	NO	NOT CLEAR	YES	YES	NO	BASIC ISSUE WAS NON PAYMENT OF RENT. DETAILS OF BED BUG ISSUE WERE REALLY NOT THERE	FAIR UNDER CIRCUMSTANCES			TPH
			**SCORE**					1	1	1				1	0	1		**5**	**P**	
41	13390	2011	P. GUZINA	T	T	MAINTENANCE,DAMAGES, HOTEL	HOTEL ROOM AND COSTS	UNIT INFESTED. FEMALE TENANT PREGNANT AND HAD TO BE OUT OF UNIT	TENANT CLAIMED COMPLAINING FOR MORE THAN A YEAR. CLAIM FOR ALL FURNITURE AND POSSESSIONS. AND HOTEL ROOM	NO DETAILS ON SPECIFICS OF INFESTATION, BUT UNIT WAS TREATED TWICE.	NO	YES	NO	YES	NO	FAIR. COSTS OF HOTEL ROOM. THE RECOMMENDATION FOR PREGNANT WOMEN TO VACATE DOES NOT USUALLY OBLIGATE LANDLORD. ISSUE OF FAULT? AS OPPOSED TO OBLIGATION	FAIR .. DID NOT ORDER PAYMENT FOR FURNITURE AND POSSESSIONS			FAMILY DOCTOR TREATING FOR BED BUG BITES
			**SCORE**					1	1	0				0	0	1		**3**	**P**	
42	5965	2011	R. CAREY	L	0	INTERFERENCE OF LANDLORD OR OTHER TENANT	0	TENANT WOULD NOT PREPARE UNIT FOR TREATMENT	TENANT REFUSED TO PREPARE FOR TREATMENT ON 4 OCCASIONS	NOTICE OF TERMINATION GIVEN, AND TENANT MUST COMPLY, BUT LANDLORD DID NOT ATTEMPT TO TREAT DURING THE PERIOD	YES	YES	NO	NO	NO	THIS FELL TO A LEGAL DETAIL. LANDLORD FAILED TO TRY TO TREAT, THEREFORE NOTICE OF TERMINATION WAS VOIDED.	TECHNICALITY AS PROBLEM IS STILL THERE AND NOTHING IN THE ADJUDICATION PROVIDES SUPPORT TO TENANT OR A PROCESS TO PREPARE, HENCE -1 ON ACTION IN RELATION TO DETAIL CLAIM			
			**SCORE**					1	-1	1				0	0	-1		**0**	**F**	
43	27139	2011	R. CAREY	L	L	INTERFERENCE WITH LANDLORD AND OTHER TENANTS, ILLEGAL ACTIVITY, LACK OF CO-OPERATION	END OF TENANCY, COSTS	BED BUGS ONLY SIDE ISSUE. MAJOR ANTI-SOCIAL BEHAVIOUR. DRUGS, ETC	TENANT REFUSED TREATMENT AND DID NOT CO-OPERATE, BUT WAS LATER CO-OPERATIVE.. THIS WAS THEREFORE NOT THE MAIN REASON FOR THE TERMINATION OF TENANCY	TENANT ON ODSP, BUT MANY ISSUES	YES	NO	NO	TPH	NO	COMPLEX CASE. BED BUG ISSUE ONLY A SMALL PART OF THIS.. WAS HANDLED APPROPRIATELY IN RELATION TO THE EVICTION/TERMINATION ORDER, AND WAS EXCLUDED IN ORDER.	HANDLED AS WELL AS COULD BE EXPECTED CONSIDERING THE COMPLEXITIES UNRELATED TO PEST INFESTATIONS.			PH
			**SCORE**					1	1	1				1	0	1		**5**	**P**	
44	13393	2011	S.COLLINS	L	TL	RENT ARREARS BY LANDLORD. TENANT RAISED ISSUES OF ILLEGAL ENTRY, HARASSMENT, LOSSES	TENANT TO PAY ARREARS AND COSTS OF CLAIM. LANDLORD TO PAY FOR ILLEGAL ENTRY, LOSS DUE TO DEFECTIVE FRIDGE	VARIOUS ITEMS. TENANT CLAIMS INFESTED AT MOVE IN. DISPUTED. LOST COUCH. FOOD LOSSES.	LANDLORD SCHEDULED TREATMENT, BUT UNIT NOT PREPARED.	TENANTS LEFT WITH CHILDREN. UNIT NOT TREATED . LANDLORD CLAIMS NOT PREPARED PROPERLY. BED COVERED IN PLASTIC (NEW?). TENANT SAY DID NOT BRING INFESTATION. REPORTS OF INFESTATION IN BUILDING. REPORTED ROACHES IN COMMON HALLWAY. LANDLORD BAITS CEILINGS IN HALLWAYS. DEAD MICE IN UNIT. LANDLORD GIVES TRAPS TO TENANTS WHO ASK. SOME OF LANDLORD COMMENTS VERY WEAK ("TESTIFIED THAT FEMALE TENANT DIDN'T COMPLAIN ABOUT MICE ONLY THAT SHE FOUND A COUPLE OF DEAD MICE. TENANT REPORTED HEARING BABY MICE IN COUCH. LANDLORD ADVISED TO "VACUUM".	YES	YES	YES	NO		LOSS CLAIMS HANDLED FAIRLY, BUT CONSIDERING THAT THERE WAS INDICATION OF ROACH AND MOUSE INFESTATIONS, IT IS NOT UNLIKELY THAT THE TENANT'S CLAIMS OF INFESTATION AT MOVE IN WERE REASONABLE. THE FACT THAT A NEWBORN WAS BEING BITTEN, THE ABATEMENT AWARDED TO TENANT WAS MEAGRE.	THE ADJUDICATOR CONSIDERED THAT THE LANDLORD'S TREATMENT EFFORTS WERE REASONABLE, AND ACTIONS WERE PROMPT REASONABLE AND PROFESSIONAL. THE FACT TENANT WAS NOT PREPARED FOR THE SCHEDULED TREATMENT WAS A MAJOR DETERMINING FACTOR IN THIS, HOWEVER, THE FACT OF THE EXISTENCE OF THREE PEST INFESTATIONS AND LANDLORD ONLY ACTING ON TENANT REQUESTS PAINTS QUITE A DIFFERENT PICTURE AS WELL AS VERY LOW QUALITY OF RESPONSE TO MOUSE ISSUES.			
			**SCORE**					1	1	-1				0	0	-1		**0**	**F**	
